# A Modular Synthesis of Teraryl‐Based α‐Helix Mimetics, Part 5: A Complete Set of Pyridine Boronic Acid Pinacol Esters Featuring Side Chains of Proteinogenic Amino Acids

**DOI:** 10.1002/ejoc.202101280

**Published:** 2022-02-24

**Authors:** Melanie Trobe, Till Schreiner, Martin Vareka, Sebastian Grimm, Bernhard Wölfl, Rolf Breinbauer

**Affiliations:** ^1^ Institute of Organic Chemistry Graz University of Technology Stremayrgasse 9 8010 Graz Austria

**Keywords:** Borylation, Cross-coupling, Peptide mimetics, Protein-protein interactions, Pyridine boronic acid pinacol ester

## Abstract

Teraryl‐based α‐helix mimetics have proven to be useful compounds for the inhibition of protein‐protein interactions (PPI). We have developed a modular and flexible approach for the synthesis of teraryl‐based α‐helix mimetics using pyridine containing boronic acid building blocks to increase the water solubility. Following our initial publication in which we have introduced the methodology in combination with sequential Pd‐catalyzed cross‐coupling for teraryl assembly, we can now report a complete set of pyridine based boronic acid building blocks decorated with side chains of all proteinogenic amino acids relevant for PPI (Ala, Arg, Asn, Asp, Cys, Gln, Glu, His, Ile, Leu, Lys, Met, Phe, Ser, Thr, Trp, Tyr, Val) to complement the core fragment set. For a representative set of teraryls we have studied the influence of the pyridine rings on the solubility of the assembled oligoarenes.

## Graphical abstract

A set of 5‐substituted 3‐pyridine boronic acid pinacol esters decorated with the side chains of all proteinogenic amino acids relevant in protein‐protein interactions is reported. This work completes our efforts towards a library of building blocks for the modular assembly of teraryl‐based α‐helix mimetics via sequential Pd cross‐coupling.

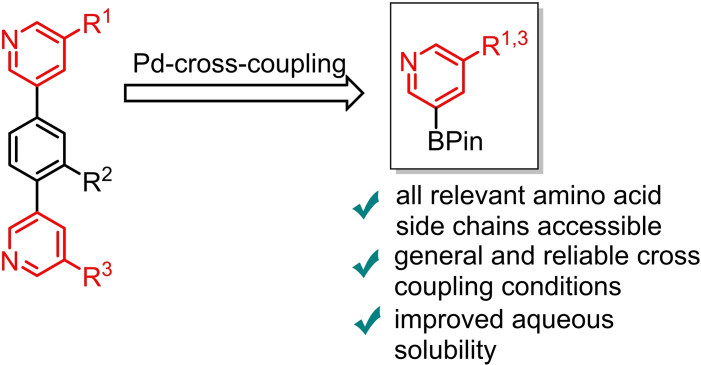

## Introduction

**Figure 1:**
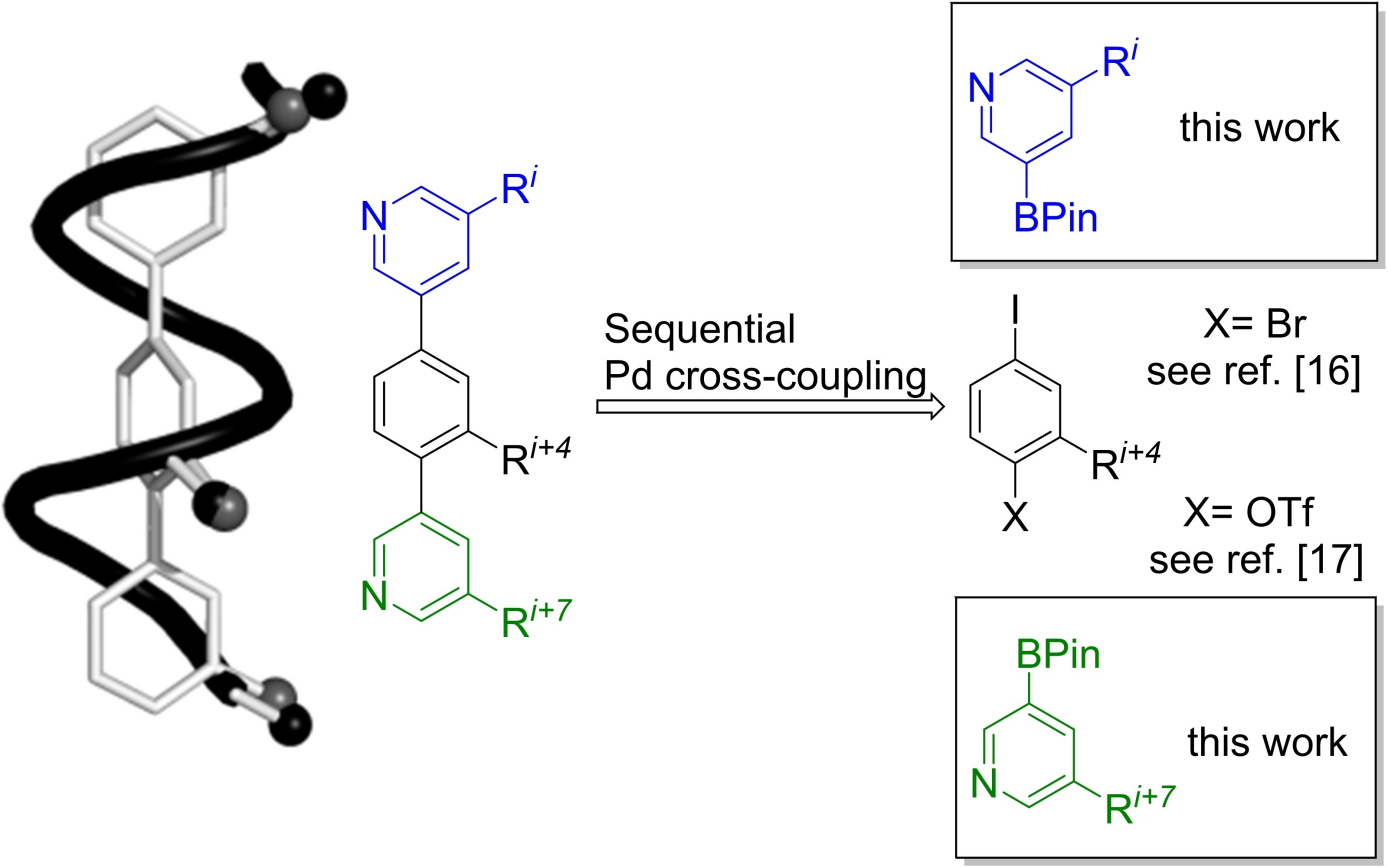
Design principle of teraryl‐based alpha‐helix mimetics (BPin: boronic acid pinacol ester).

Protein–protein interactions (PPIs) are recognized as one of the main factors in controlling protein function in living cells.^[1]^ The number of different PPIs is estimated to be beyond the validated 110,000.[^1^, ^2^, ^3^] From a structural perspective, α‐helices play the most prominent role in the interaction sites, offering a huge opportunity for Chemical Biology and Medicinal Chemistry.[^1^, ^3^] PPIs are intrinsically difficult to inhibit with small molecules as the interfaces of PPIs are rather large with many residues contributing to the binding energy,^[4]^ but Hamilton and co‐workers have presented a quite general approach of mimicking α‐helices by suitable positioning of amino acid side chains around a terarylic scaffold.^[5]^ As many of these terphenyls show poor solubility in aqueous solvents, more polar heteroaryl‐based helical mimetics have been developed, such as diphenylacetylene‐,^[6]^ imidazole‐phenyl‐thiazole‐,^[7]^ oligobenzamide‐,^[8]^ oligopyridylamide‐,^[9]^ piperazine‐,^[10]^ pyridazine‐,^[11]^ pyrrolopyrimidine‐,^[12]^ or triazolo‐based scaffolds.^[13]^ Pyridine‐based teraryls have been reported in which the pyridine nitrogen atoms are mostly positioned on the same site as the proteinogenic side chains.^[14]^ Our group has introduced a modified design, in which the pyridine nitrogen atoms of the teraryl‐based α‐helix mimetics are positioned to the water‐exposed face distal to the protein binding site,^[15]^ which should reduce the entropic cost of binding. In addition, our design builds on an efficient synthetic approach to amino acid surrogate pyridine boronic acid building blocks, which in combination with core fragments containing two differentiated leaving groups[^16^, ^17^] for sequential cross‐coupling,^[18]^ represents a universal and flexible approach for the synthesis of pyridine‐based α‐helix mimetics (Figure 1).[^19^, ^20^]

In our original report we had presented the synthesis of a few representative pyridine boronic acid building blocks (Phe, Leu, Ile, Lys, Asp, Asn) using methodology relying on transmetalation with Knochel‐Grignard reagents and electrophilic quench with aldehyde electrophiles.^[15]^ In this report, we now present a complete synthetic tool kit to access all pyridine boronic acid building blocks mimicking amino acid side chains relevant in PPI using improved and extended methodology.

## Results and Discussion

**Scheme 1:**
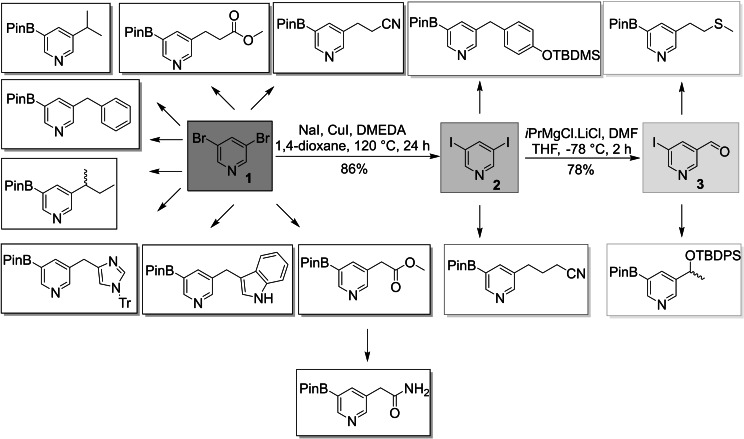
Overview of the synthetic routes towards the pyridine boronic acid building blocks (DMEDA: *N,N’*‐dimethylethylene diamine; TBDMS: *tert*‐butyldimethyl silyl; TBDPS: *tert*‐butyldiphenyl silyl; Tr: trityl).

We aimed to establish short, high yielding and scalable reaction sequences preferably without the use of protecting groups. Furthermore, the use of general intermediates was desirable, which would allow for a more efficient and rapid generation of a building block library. Proline and glycine were not included in our synthetic efforts as these amino acids are not of relevance as hot‐spot residues of α‐helices.^[21]^ Gratifyingly, we were able to use three universal intermediates for the synthesis of 13 out of 18 pyridine building blocks mimicking the side chains of the relevant amino acids. The first central intermediate was commercially available 3,5‐dibromopyridine (**1**), which was converted to 3,5‐diiodopyridine (**2**) via a Cu‐catalyzed Buchwald‐Finkelstein reaction.[^15^, ^22^] In the next step, by using a Knochel‐Grignard reaction, one iodide underwent metal‐halogen exchange, which was quenched with DMF and upon hydrolysis yielded 5‐ iodonicotinaldehyde (**3**) in 67 % yield over two steps (Scheme 1).^[23]^

**Scheme 2:**
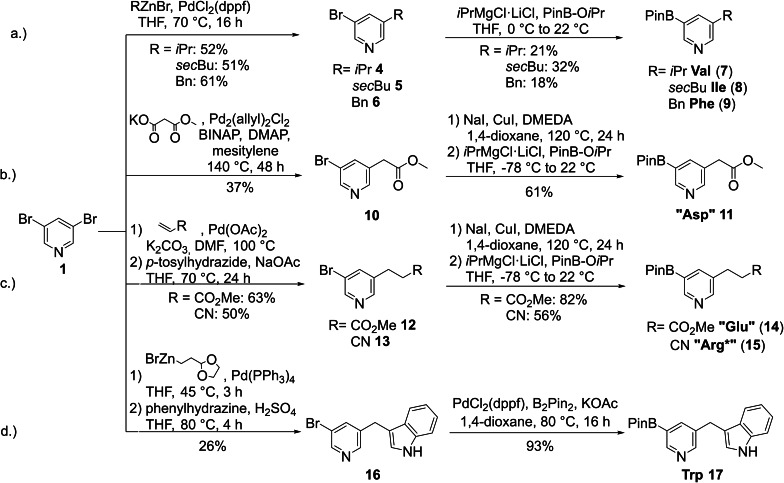
Synthesis of the Val, Ile, Phe, “Asp”, “Glu”, “Arg*” and Trp building blocks (dppf: 1,1’‐bis(diphenylphosphino)ferrocene; BINAP: 2,2′‐bis(diphenylphosphino)‐1,1′‐binaphthyl; DMAP: 4‐(dimethylamino)pyridine).

The majority of building blocks was synthesized utilizing 3,5‐dibromopyridine (**1**) as starting material. In most cases, one bromide was selectively converted to the desired side chain via a Pd‐catalyzed cross‐coupling reaction, followed by introduction of the boronic acid pinacol ester replacing the second halide either via a Knochel‐Grignard reaction or Miyaura borylation (Scheme 2).

In our previously reported route to synthesize the Ile, Phe and also Leu building blocks a twofold Knochel‐Grignard reaction sequence was used.^[15]^ The first metal‐halogen exchange intermediate was reacted with various aldehydes to introduce the side chains and the second metal halogen exchange was reacted with PinB‐O*i*Pr. The big drawback of this reaction sequence was the fact that it required several reaction steps to remove the hydroxyl function formed during the first Grignard reaction without destroying the boronic acid group. Moderate yields and long reaction sequences turned out to be problematic for scale up. In order to streamline this process, we present here an alternative synthetic strategy in which the amino acid alkyl side chain is introduced via a single Negishi‐coupling of 3,5‐dibromopyridine with various Zn‐organyls. A procedure established by Knochel was used to activate commercially available zinc dust for the preparation of the Zn organyls.^[24]^ Since the case of the Val, Ile and Phe side chains no sensitive functional groups are present, it was possible to convert the bromide leaving group in **4**–**6** directly to the boronic acid pinacol ester via metal‐halogen exchange at elevated temperatures (Scheme 2a). Although the overall yields could not be improved compared to the original route,^[15]^ we regard this route as more attractive since the longest linear sequence was reduced from 5 reaction steps to only 2.

For the synthesis of pyridine building blocks with polar side chains, a more elaborate strategy had to be chosen. The “Asp” (Please note that building blocks marked with “ “ were synthesized in a protected form.) side chain was introduced via a decarboxylative cross‐coupling procedure^[25]^ starting from 3,5‐dibromopyridine (**1**) and monopotassium methyl malonate. For building blocks with electrophilic side chains, it was instrumental that the remaining bromide on the pyridine is converted to the more reactive iodide via a Cu‐catalyzed Buchwald‐Finkelstein reaction. The higher reactivity of the iodine leaving group is needed for a successful metal‐halogen exchange allowing the introduction of the boronic ester group at low temperatures. When this reaction was performed at higher temperatures as necessary for bromides to undergo metal halogen exchange, the sensitive ester group also reacted with the Knochel‐Grignard reagent. In contrast, with iodine the metal‐halogen exchange already selectively occurred at −78 °C and the functional group remained intact (Scheme 2b).^[26]^ However, during this reaction isopropanolate was formed within the electrophilic quench with PinB‐O*i*Pr, which caused a partial transesterification, resulting in an inseparable mixture of desired methyl ester (Me) and isopropyl ester (*i*Pr) of “Asp” building block **11**. The “Asp” building block was used as a mixture in all following reactions. This was only a minor nuisance as in the final stages of the teraryl synthesis the ester side chains of “Asp” and “Glu” have to be saponified to the free carboxylic acids anyway.

The “Glu” and “Arg*” (Please note that building blocks marked with “*” were synthesized in a latent form and have to be converted into the desired functional group after cross coupling.) side chains were introduced via a Heck reaction of 3,5‐dibromopyridine (**1**) with methyl acrylate or acrylonitrile, followed by a diimide reduction of the formed double bond, delivering **11** and **12**, respectively. Our original plan to use the more reactive 3,5‐diiodopyridine (**2**) for the Heck reaction was not pursued further as a significant amount of dicoupling product from the reaction at both iodides was observed. Therefore, again a bromide‐iodide exchange was indispensable as only with iodine the metal‐halogen exchange in the following step selectively occurred at −78 °C and the functional groups (−CO_2_Me and −CN) remained intact in both cases (Scheme 2c). As observed for the “Asp” building block isopropanolate formation resulted in an inseparable mixture of desired methyl ester (Me) and isopropyl ester (*i*Pr) of “Glu” building block **12**.

**Scheme 3:**
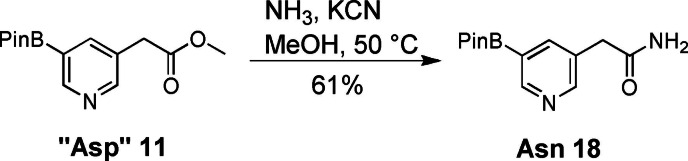
Synthesis of the Asn building block.

“Asp” **11** was converted to Asn (**18**) in 61 % yield via aminolysis with NH_3_ in MeOH in the presence of the installed pinacol boronic ester (Scheme 3). To our surprise, we could not find conditions to convert “Glu” to Gln in the same manner due to incomplete conversion and formation of side products. We therefore recommend using either “Glu” or “Arg” building blocks **14** and **15** as Gln surrogates. The free amide can be generated from these side chains after cross coupling either via aminolysis of an ester or hydrolysis of a nitrile, which should be compatible with the other two functional groups of the teraryl.

A formidable challenge was the synthesis of the Trp building block as all attempts to synthesize this compound by directly attaching an indole group to the pyridine core were unsuccessful. Therefore, a different strategy was pursued in which the indole ring was synthesized from scratch. First, a masked aldehyde was attached to 3,5‐dibromopyridine via Negishi coupling. It was found that for optimal yields the deprotection occurred best in situ under the acidic conditions during Fischer indole synthesis yielding **14** in 26 % yield over two steps. In the last step the bromide was efficiently converted to the corresponding boronic acid pinacol ester via Miyaura borylation delivering the Trp building block **17** in 24 % overall yield (Scheme 2d).

**Scheme 4:**
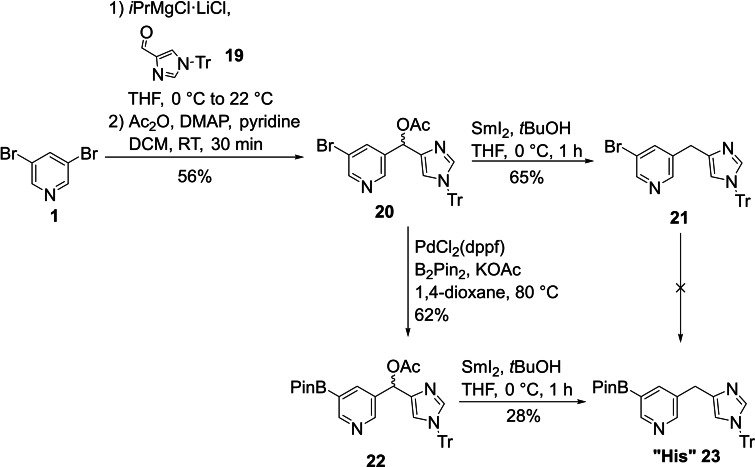
Synthesis of the “His” building block.

Even more difficult was the synthesis of the His building block, for which several routes had to be explored. In our original synthesis plan, we aimed to introduce the imidazole side chain of the “His” building block via Grignard reaction of metalated 3,5‐dibromopyridine with aldehyde **19**.^[27]^ The resulting racemic alcohol should then be reduced directly in a one‐step deoxygenation. However, upon treatment with reducing agents such as Et_3_SiH/TFA only decomposition of the imidazole heterocycle was observed. Therefore, the alcohol was converted into acetyl derivative **20**, which was subjected to SmI_2_‐mediated reduction of the benzylic acetate, yielding **21**.^[28]^ Surprisingly, it was impossible to introduce the boronic ester moiety at this stage of the synthesis. Even though seemingly no reactive functional groups are present in **21**, all attempts converting **21** to **23** via either Miyaura borylation or metal‐halide exchange only led to decomposition of the starting material. However, the boronic ester group could be introduced by performing the Miyaura borylation with acetyl derivative **20**, in which case no decomposition was observed and the product **22** could be isolated in 62 % yield. To our delight, SmI_2_‐mediated reduction smoothly proceeded also with the boronic ester present in the molecule. While the reaction proceeded in high conversion, the purification of the “His” building block **23** was difficult, leading to a rather low isolated yield (28 %) in this reaction (Scheme 4).

**Scheme 5:**
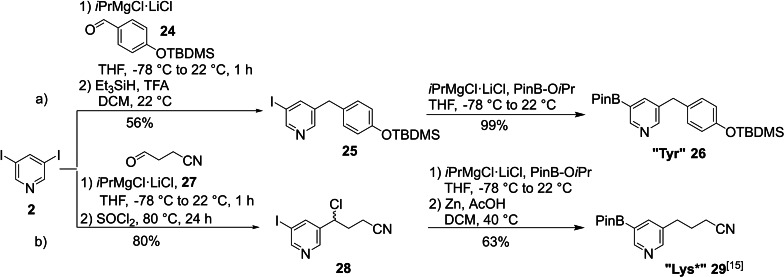
Synthesis of the “Tyr” and “Lys*” building blocks (TFA: trifluoroacetic acid).

The “Tyr” building block **26** was synthesized by a two‐fold Knochel‐Grignard route by adding protected aldehyde **24** to metalated 3,5‐diiodopyridine (**2**) under the formation of a secondary alcohol. The electron rich phenolether moiety in this molecule facilitated the deoxygenation using Et_3_SiH and TFA (56 % yield), by stabilizing the formed carbocation intermediate. After Knochel‐Grignard mediated borylation “Tyr” (**26**) could be isolated in 99 % yield (Scheme 5a). The synthesis of the “Lys*” building block **29** from 3,5‐ diiodopyridine was performed as described in our original report (Scheme 5b).^[15]^

**Scheme 6:**
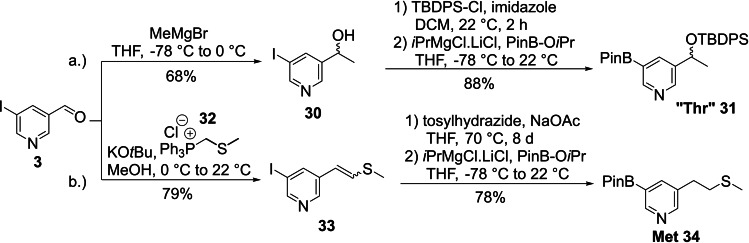
Synthesis of the “Thr” and Met building blocks.

The “Thr” and Met building blocks were synthesized starting from the general intermediate 5‐iodonicotinaldehyde (**3**). To access the “Thr” side chain the aldehyde was reacted with MeMgBr yielding the racemic secondary alcohol **30**. The reaction temperature was kept at −78 °C to avoid any metal‐halogen exchange with the iodine. In the next step the secondary alcohol was protected with *tert*‐butyldiphenylchlorosilane (TBDPSCl). The boronic acid pinacol ester was introduced via the standard Knochel‐Grignard metal‐halogen exchange, followed by electrophilic quench with PinB‐O*i*Pr to generate the “Thr” building block **31** (Scheme 6a).

The Met side chain was introduced via a Wittig reaction and subsequent diimide reduction of the resulting olefin. To achieve full conversion 12 eq of diimide precursor were necessary, which were added over the course of 8 d to provide sufficient amounts of reactive diimide during the whole reaction time. In the last step the boronic acid pinacol ester was introduced via the standard Knochel‐Grignard procedure, furnishing Met building block **34** in 78 % yield (Scheme 6b).

**Scheme 7:**
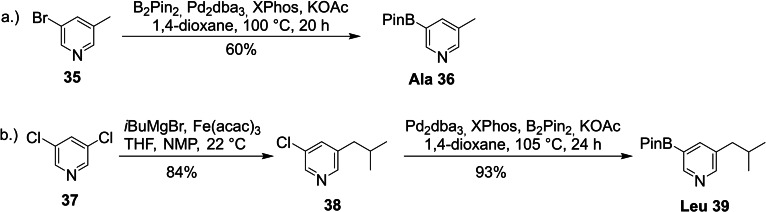
Synthesis of the Ala and Leu building blocks (acac: acetylacetonate; dba: dibenzylideneacetone; *i*Bu: isobutyl; NMP: *N*‐methyl 2‐pyrrolidone).

For the remaining building blocks a diverse set of starting materials was used to allow the most straightforward and shortest sequence to the desired final products. Commercially available 3‐bromo‐5‐methylpyridine (**35**) was converted to the Ala building block **36** via Miyaura borylation in 60 % yield (Scheme 7a).

The Leu building block **39** was not accessible via the same Negishi coupling strategy as described above for the other aliphatic amino acid side chains since this substrate is prone to β‐hydride elimination. Therefore, a Fe‐catalyzed Kochi‐Fürstner‐coupling was performed with 3,5‐dichloropyridine.^[29]^ As the remaining chloride was unsuitable for a Knochel‐metal halogen exchange, we used a Miyaura borylation to introduce the boronic acid pinacol ester (Scheme 7b).

**Scheme 8:**
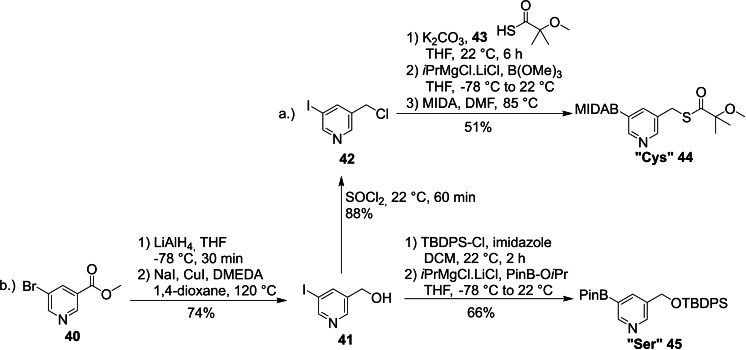
Synthesis of the “Cys” and “Ser” building blocks (MIDA: *N*‐methyliminodiacetic acid).

Methyl 5‐bromonicotinate (**40**) was reduced with LiAlH_4_ as precursor for “Cys” and “Ser” building blocks. To facilitate the metal‐halogen exchange in the last step of the reaction sequence the bromide was then converted to the corresponding iodide (74 % over two steps). It was not possible to perform these two reaction steps in the reversed order since the iodide was not stable under LiAlH_4_ reduction conditions. For the “Cys” side chain the alcohol was then chlorinated with SOCl_2_ and a thioester was introduced via nucleophilic substitution, presenting the protected “Cys” side chain. The used protecting group was chosen as it has been reported to provide good stability during Pd‐catalyzed cross‐coupling reactions.^[30]^ It was not possible to purify the pinacol ester derivative of this building block. Therefore, the boronic acid was protected by a MIDA group,^[31]^ which allowed isolation of corresponding compound **44** in 33 % overall yield after purification via column chromatography (Scheme 8a).

The LiAlH_4_ reduction of **40** described above had already provided the correct side chain of the “Ser” building block **45**, which was TBDPS‐protected as already practiced for the “Thr” building block. In the last step the iodine was converted to the corresponding boronic acid pinacol ester via the Knochel‐Grignard reaction sequence, delivering “Ser” (**45**) in 66 % yield (Scheme 8b).

With the set of pyridine building blocks completed, the assembly of teraryls was investigated. We synthesized 5 teraryls featuring various side chains by connecting two different pyridine boronic acid building blocks to a core fragment bearing two differentiated leaving groups‐either −I/−OTf^[19]^ or −I/−Br^[16]^. For all coupling reactions, two general procedures could be applied.

**Scheme 9:**
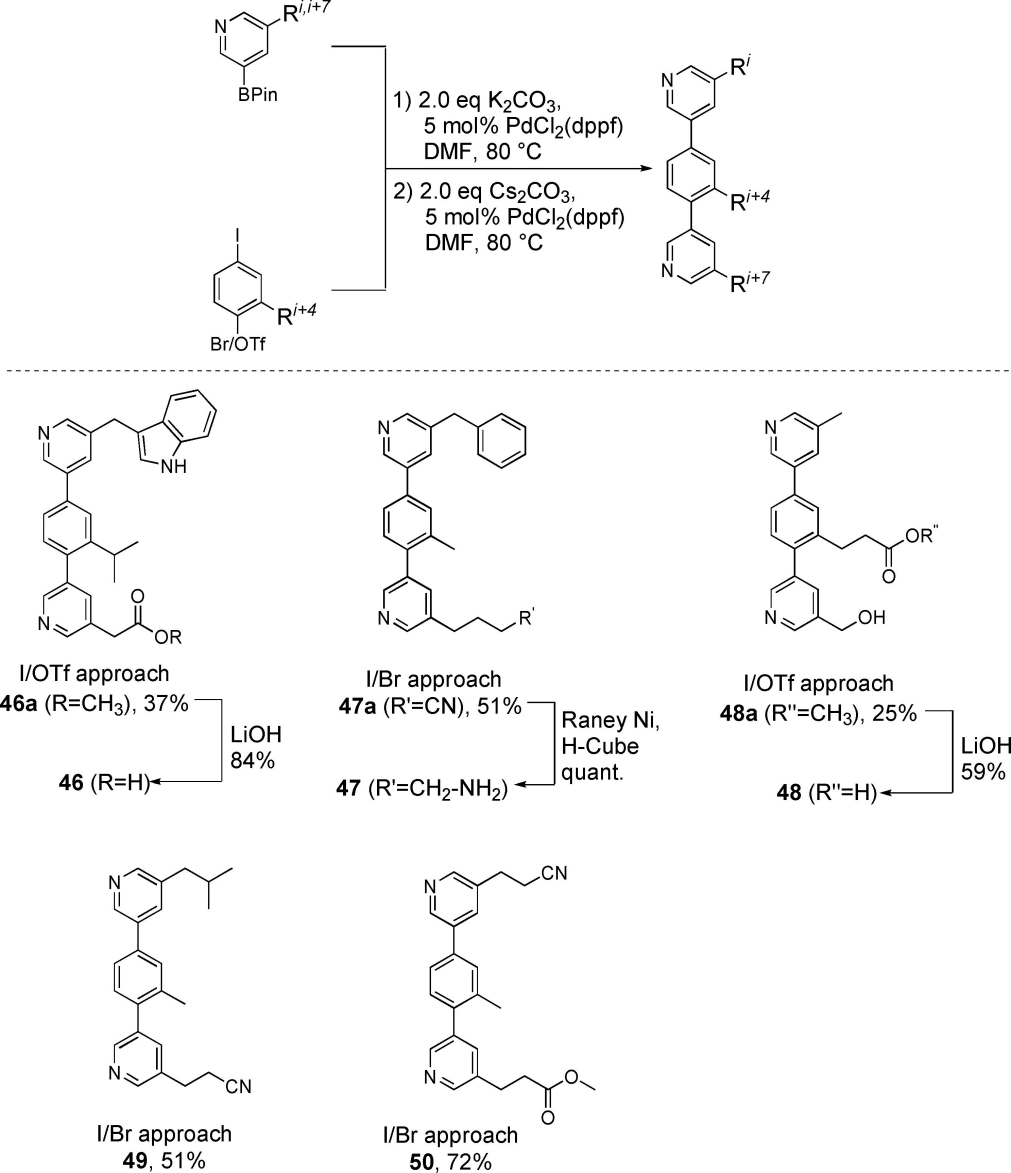
Teraryl assembly and deprotection steps.

Selective coupling of the more reactive iodine leaving group at the core unit with the first pyridine building block was achieved by using PdCl_2_(dppf) as catalyst and K_2_CO_3_ as base. Switching to the stronger base Cs_2_CO_3_ allowed for coupling of the remaining less reactive leaving group (Br or OTf) with the second pyridine building block. Deprotection of the “Ser” side chain in **48a** occurred already during the cross‐coupling step. While the coupling reactions proceeded chemoselectively in all cases, a side reaction was the hydrolysis of aryl triflate intermediates. Therefore, higher yields were achieved when commercially available 2‐bromo‐5‐iodo toluene was used as starting material instead of the −I/−OTf core fragments. After the coupling reactions protected functional groups were deprotected. The free acid functionalities Asp and Glu in **46a** and **48a** were exposed after saponification, delivering Trp‐Val‐Asp (**46**; 31 % over three steps) and Ala‐Glu‐Ser (**48**; 15 % over three steps) mimetics, respectively. The nitrile function of the latent *Lys*” side chain in **47a** was reduced to the desired amine, delivering Phe‐Ala‐Lys (**47**) in 51 % overall yield (Scheme 9).

**Scheme 10:**
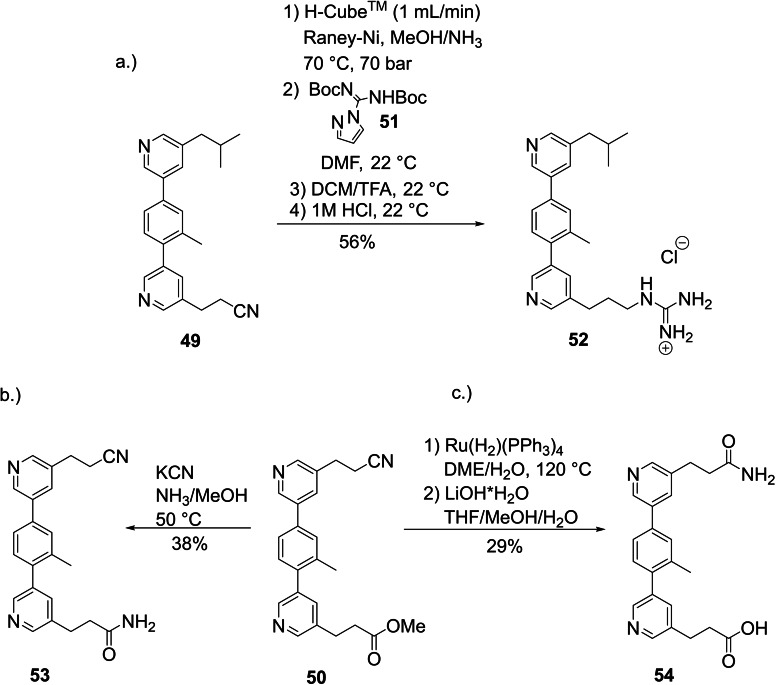
Introduction of the natural Arg and Gln side chains.

In a similar fashion, **49** was reduced with H_2_/Raney‐Ni to the corresponding free amine, which was then treated with guanylating agent **51** and subsequently deprotected to present the natural Arg side chain in the Leu‐Ala‐Arg teraryl **52** in 56 % yield over four steps (Scheme 10a).^[32]^ The free amide moiety of the Asn side chain could be selectively introduced at either functional group in teraryl **50**. Aminolysis of the ester group yielded compound **53** (Scheme 10b), while Ru‐catalyzed hydration of the nitrile function,^[33]^ followed by saponification of the untouched ester group, delivered Gln‐Ala‐Glu mimetic **54** in 29 % yield over two steps (Scheme 10c). These examples demonstrate that our modular assembly approach allows the synthesis of any desired substitution pattern of α‐helix mimetics.

**Figure 2:**
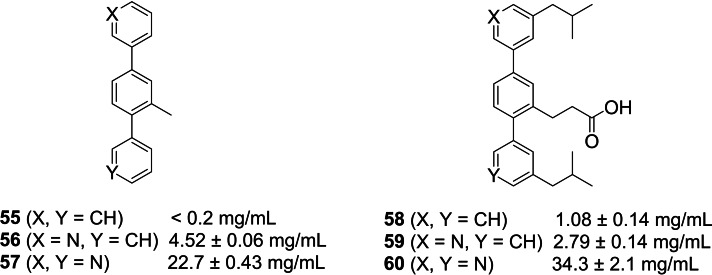
Solubility studies.

Finally, in order to demonstrate the influence of the backbone nitrogen on the water solubility, we prepared two series of teraryls containing either none, one or two pyridine rings. The first series consist of unsubstituted teraryls to evaluate how much the nitrogen atoms in the backbone contribute to increased water solubility. The second series looks at the additional influence of polar and nonpolar side chains (Figure 2). The kinetic solubility of the two teraryl series was measured by HPLC in a phosphate buffer (pH=7.4). The results confirm our initial hypothesis that the solubility of teraryls can be significantly increased by the introduction of pyridine moieties. The solubility of compound **55**, bearing only phenyl groups and an aliphatic side chain was below the limit of detection.^[34]^ In comparison, the solubility of terphenyl **58**, bearing two hydrophobic Leu side chains, was significantly higher (1.08 mg/mL), most likely because of the presence of the polar carboxylic acid moiety in the Glu side chain. Compared to the underlying terphenyl analogues, the compounds bearing one pyridine group (**56** and **59**) exhibited significantly higher kinetic solubility (4.52 resp. 2.79 mg/mL). Interestingly, **59** was unexpectedly found to be less soluble than **56**. This might be due to hydrophobic repulsion and steric shielding of the two Leu side chains.

Introduction of a second pyridine ring improved the solubility of the teraryls **57** and **60** even further (22.7 resp. 34.3 mg/mL). Compound **60** exhibits the highest kinetic solubility, showing that polar side chains can also have a positive influence on the solubility of our teraryls. While the kinetic solubility was measured in a buffer with neutral pH, it should be noted that protonation of pyridine moieties under acidic pH‐for instance in the stomach‐should further improve the solubility of our compounds.

## Conclusion

**Figure 3:**
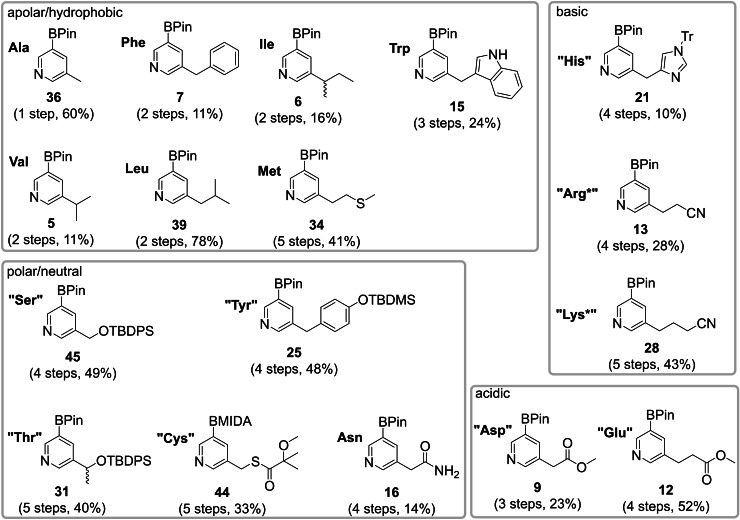
Complete set of pyridine boronic acid building blocks.

In conclusion, we can now present a set of pyridine boronic acid building blocks featuring all relevant proteinogenic amino acids for the modular synthesis of teraryl‐based α‐helix mimetics (Figure 3). For several building blocks it was necessary to prepare them in a protected or latent form to ensure reagent stability and high yields in sequential Suzuki coupling assembly. In an example we could show that both “Arg*” and “Glu” building blocks can be converted to the natural Gln side chain after cross coupling.

For most of these building blocks, the boronic ester group was introduced via the corresponding aryl bromides or iodides either via Miyaura borylation or via Knochel‐Grignard metal‐halogen exchange reaction followed by electrophilic quench with PinBO*i*Pr. In general, reactions utilizing metal‐halogen exchange of aryl iodides proceeded very smoothly and often, the product was obtained in pure form after a simple extraction step. In contrast, Miyaura borylation was found to be more effective starting from aryl bromides rather than iodides. This reaction was more tolerant towards sensitive, i. e., functionalized side chains, but also usually made purification of the products more challenging. As the pyridine boronic esters turned out to be unstable on silica gel, we used Kugelrohr distillation, sublimation, or recrystallization as purification methods. However, in some cases, none of these methods seemed to work appropriately and only very low yields of the final building blocks could be isolated.

A possible solution for this problem could be using MIDA boronates for these problematic building blocks.^[31]^ Burke has shown that MIDA boronates are extraordinarily stable, even in combination with heterocycles that are unstable as boronic acid and therefore hard to cross‐couple.^[35]^ The free boronic acid is readily released under mild aqueous basic conditions or slowly during coupling itself.^[36]^ Furthermore, MIDA boronates tend to be crystalline and often recrystallization is possible, or they can be purified by catch‐and‐release chromatography.

Together with the complementary set of core fragments, containing two leaving groups of differentiated reactivity in Pd‐catalyzed cross coupling reactions, this work represents a significant step forward towards the modular synthesis of teraryl based α‐helix mimetics and allows comprehensive coverage of the protein sequence space. Ongoing collaborations with biology groups have resulted in first promising biologically active PPI inhibitors and will be reported in due course.

## Experimental Section

### Representative procedure for Cu‐catalyzed Buchwald‐Finkelstein reaction

A Schlenk‐flask was charged with the corresponding pyridine‐derivative (1.0 eq) which was dissolved in abs., degassed 1,4‐dioxane (0.6 M). *N,N*'‐Dimethylethylenediamine (0.1 eq), NaI (4.0 eq) and CuI (10 mol %) were added. The green suspension was stirred at 120 °C until full conversion was detected via GC‐MS (24 h). Then the reaction was cooled to RT and quenched by the addition of satd. NH_4_Cl solution. A light brown precipitate was formed, which was removed by filtration through a pad of Celite® (eluted with DCM). The phases were separated, and the dark blue aqueous phase was extracted with DCM. The combined organic layers were dried over Na_2_SO_4_, filtered and the solvent was removed under reduced pressure. The crude product was purified via flash column chromatography.

### Representative procedure for Knochel‐Grignard reaction

A Schlenk‐flask was charged with the corresponding pyridine‐derivative (1.0 eq) dissolved in abs. THF (0.3 M). The reaction mixture was cooled to −78 °C (0 °C when a bromide is used as starting material) and *i*PrMgCl.LiCl solution (1.5 M in THF, 1.2 eq) was added dropwise. When complete metal‐halogen exchange was detected by GC‐MS (2 h), PinBO*i*Pr (1.5 eq) were added to the reaction mixture. The reaction was allowed to warm up in the cooling bath overnight and full conversion was detected via GC‐MS (24 h). The reaction mixture was quenched by the addition of satd. NH_4_Cl solution. The phases were separated, and the aqueous layer was extracted with DCM. The combined organic layers were washed with brine, dried over Na_2_SO_4_ and concentrated in vacuum. If necessary, the product was further purified via Kugelrohr distillation, sublimation or recrystallization.

### Representative procedure for the synthesis of teraryls by consecutive double Suzuki‐Coupling (1^st^ step)

A flame dried Schlenk‐flask was charged with 1.0 eq of the corresponding boronic acid derivative, 2.0–3.0 eq K_2_CO_3_, and 5 mol % PdCl_2_(dppf). After drying in vacuo, a solution of 1.0 eq core unit fragment in abs., degassed DMF (∼0.2 M) was added. The reaction mixture was stirred at 80 °C until full conversion was detected by GC‐MS or TLC. The typically brown suspension was filtered through a pad of SiO_2_ (eluted with MeOH) and the filtrate was concentrated to dryness using a rotary evaporator. The crude product was purified via flash column chromatography or used in the next step without further purification.

### Representative procedure for the synthesis of teraryls by consecutive double Suzuki‐Coupling (2^nd^ step)

Another flame dried Schlenk‐flask was charged with 1.0–1.2 eq of the second boronic acid pinacol ester, 2.0–3.0 eq Cs_2_CO_3_, and 5 mol % PdCl_2_(dppf). After drying in vacuo, a solution of the previously prepared intermediate in abs., degassed DMF (∼0.2 M) was added. The reaction mixture was stirred at 80 °C overnight. The typically black suspension was filtered through a pad of SiO_2_ (eluted with MeOH) and after concentrating to dryness, the crude product was purified via flash column chromatography.

### Representative procedure for water solubility tests^[34]^

The kinetic solubility of teraryls was measured by HPLC using individual calibration curves. For the preparation of the solubility samples and the calibration standards a DMSO stock solution for each compound was prepared. For this, 10 μmol of the corresponding teraryl were dissolved in 1 mL DMSO (10 mM stock solution). In the next step 10 μL of the 10 mM DMSO stock solutions were added to 990 μL of PBS buffer (pH 7.4). The created 100 μM stock solutions were mixed for 5 s on a Grant‐bio vortex mixer and agitated for 120 min at RT (500 rpm, Bohdan MiniBlock shaker). Then the solutions were centrifuged for 15 min at 14000 rpm with an Eppendorf 5415 C centrifuge. 200 μL were decanted from the top of the 100 μM stock solutions, added to 50 μL DMSO and vortexed for 5 s to obtain the solubility samples. The transfer of the centrifuged stock solution into a vial with DMSO was necessary to avoid precipitation from the saturated solution.

The 100 μM calibration standards were produced by mixing 10 μL of the 10 mM DMSO stock solution with 990 μL DMSO for 5 s on the vortex mixer. The individual calibration curves were prepared by injecting 0.5, 2.5 and 5 μL of the calibration standards and the measured areas under the curves were used to calculate a linear function. At last, the obtained areas of the solubility samples by injecting 5 or 50 μL were inserted in the linear equation to determine the kinetic solubility values. Each volume of the calibration standards and solubility samples was injected and measured three times.

## Conflict of interest

The authors declare no conflict of interest.

## Supporting information

As a service to our authors and readers, this journal provides supporting information supplied by the authors. Such materials are peer reviewed and may be re‐organized for online delivery, but are not copy‐edited or typeset. Technical support issues arising from supporting information (other than missing files) should be addressed to the authors.

Supporting Information
