# Supplementary material for: A Modular Synthesis of Teraryl‐Based α‐Helix Mimetics, Part 5: A Complete Set of Pyridine Boronic Acid Pinacol Esters Featuring Side Chains of Proteinogenic Amino Acids
Source: European J Org Chem. 2022 Feb 24;2022(17):e202101280. doi: 10.1002/ejoc.202101280 (PMC9304165; doi:10.1002/ejoc.202101280)

European Journal of Organic Chemistry

Supporting Information

A Modular Synthesis of Teraryl-Based α-Helix Mimetics, Part 5: A Complete Set of Pyridine Boronic Acid Pinacol Esters Featuring Side Chains of Proteinogenic Amino Acids

Melanie Trobe, Till Schreiner, Martin Vareka, Sebastian Grimm, Bernhard Wölfl, and Rolf Breinbauer*

1 General Experimental Aspects, Materials and Methods

NMR spectra were recorded on a Bruker Avance III 300 MHz FT NMR spectrometer (300.36 MHz (^{1}H), 75.53 MHz (^{13}C)), or on a Varian Unity Inova 500 MHz NB high resolution FT NMR spectrometer (499.76 MHz (^{1}H), 125.67 MHz (^{13}C)) at 27 °C. Chemical shifts δ [ppm] are referenced to residual protonated solvent signals as internal standard [D6]DMSO: δ = 2.50 ppm (^{1}H), 39.52 ppm (^{13}C) and $CDCl_3$: δ = 7.26 ppm (^{1}H), 77.16 ppm (^{13}C).[1] Signal multiplicities are abbreviated as s (singlet), d (doublet), dd (doublet of doublet), t (triplet), dt (doublet of triplet), q (quadruplet), dq (doublet of quadruplet), sept (septet), m (multiplet) with the prefix b in case of broad signals. Superscript abbreviations are used as follows: H^{Ar} (aromatic protons); abbreviation C_q is used for quaternary carbon atoms. ^{13}C NMR resonances were assigned by APT or 2D-HSQC and -HMBC experiments. GC-MS measurements were performed on an Agilent Technologies 7890A (G3440A) GC system equipped with an Agilent Technologies J&W GC-column HP-5MS ((5%-phenyl)-methylpolysiloxane; length: 30 m; inner-diameter: 0.250 mm; film: 0.25 µm) at a constant helium flow rate (He 5.0; Air Liquide; "Alphagaz"; 1.085 mL/min; average velocity 41.6 cm/sec) in split mode 1/175 (inlet temperature: 250°C; injection volume: 2.0 µL; sample concentration: ~0.5 mg/mL in ethyl acetate (EtOAc), methanol (MeOH), dichloromethane (DCM), or diethyl ether (Et_2O)). The GC was coupled to a 5975C inert mass sensitive detector with triple-axis detector (MSD, EI, 70 eV; transfer line: 300 °C; MS source: 240 °C; MS quad: 180 °C), with a solvent delay of 2.60 min. Two general gradients MT_50_S (initial temperature: 50 °C, 1.0 min; linear ramp: 40 °C/min; final temperature: 300 °C; final time: 5.0 min; post run 1.0 min; detecting range: 50.0 to 550.0 amu) and MT_50_XS (initial temperature: 50 °C, 1.0 min; linear ramp: 10 °C/min; 150 °C 2.0 min; linear ramp: 40 °C/min; final temperature: 300 °C; final time: 5.0 min; post run 1.0 min; detecting range: 50.0 to 550.0 amu) were applied. When reactions were monitored by GC-MS, the samples were prepared using a microscale workup. This means an aliquot was taken from the reaction mixture, quenched by the addition of ~1 mL aqueous solution and ~1 mL DCM, EtOAc, or Et_2O. After proper mixing and phase separation, the organic layer was collected, dried over $MgSO_4$ and filtered through cotton in a Pasteur-pipette. Reaction mixtures containing transition metals were additionally filtered through a short pad of silica gel (~1 cm) over cotton in a Pasteur-pipette (eluted with EtOAc or MeOH). Analytical thin layer chromatography (TLC) was performed on Merck silica gel 60-F254 and spots were visualized by UV-light (λ = 254 and/or 366 nm), and by treatment with cerium ammonium molybdate solution (CAM) (CAM: 2.0 g $Ce(IV)SO_4$, 50 g $(NH_4)_2MoO_4$, 50 mL concentrated H_2SO_4 in 400 mL water), vanillin solution (15

g vanillin in 250 mL ethanol and 2.5 mL concentrated sulphuric acid), ninhydrin solution (1.5 g ninhydrin in 100 mL n-butanol and 3.0 mL acetic acid) or $FeCl_3$ solution (5 g $FeCl_3$ in 100 mL 0.1M HCl), followed by warming with a heat gun. Flash column chromatography was performed using silica gel 60 Å (35-70 μ m particle size) from Acros Organics at an air pressure of ~1.5 bar. A 20 to 100-fold excess of silica gel was used with respect to the amount of dry raw material (exact values are given in experimental procedures). The stationary phase was filled in an appropriately sized column resulting in a pad of 15-25 cm silica gel. The column was equilibrated with the solvent or solvent mixture, and the sample was loaded onto the pad by diluting the crude product with the eluent. If the crude product was not sufficiently soluble in the eluent, the sample was dissolved in a proper solvent (MeOH or EtOAc), and the double amount of silica gel (or Celite®545, particle size 0.02-0.1 mm) was added, followed by removing the solvent using a rotary evaporator and drying in vacuo. The mobile phase was forced through the column by means of a rubber bulb pump. Analytical HPLC analysis was performed on a Shimadzu Nexera Liquid Chromatograph with a tempered column oven. The separation was performed on a C-18-Reversed-Phase column of the type „Poroshell® 120 SB-C18, 3.0 x 100 mm, 2.7 µm" by Agilent Technologies. For detection a Shimadzu SPD-M20A Prominence Diode Array Detector at a wavelength of λ = 210 nm and a mass selective detector Shimadzu LCMS-2020 Liquid Chromatograph Mass Spectrometer in ESI positive and ESI negative mode were used. The following gradient was applied for reaction control: MV_general (0.0 – 0.1 min, isocratic, 2% MeCN (98% H_2O + 0.05% TFA); 0.1 – 8.0 min, linear, 2% to 100% MeCN; 8.0 – 11.1 min, isocratic, 100% MeCN; 11.1 – 11.3 min, linear, 100% to 2% MeCN; 11.3 – 12.0 min, isocratic, 2% MeCN). For the solubility studies, the general gradient SOL_10_100 (0 – 5 min, linear, 10% CH_3CN to 100% CH_3CN (90% to 0% H_2O + 0.1% HCOOH); 5 – 8 min, isocratic, 100% CH_3CN, T = 30 °C, flow rate: 0.7 mL/min) was applied. Reversed phase preparative HPLC purifications were performed on a Thermo Scientific UltiMate 3000 system. Detection was accomplished with a Dionex UltiMate Diode Array Detector. The separations were carried out on a Macherey Nagel 125/21 Nucleodur® 100-5 C18EC (125 x 21 mm, 5 µm) column. Acetonitrile and water with 0.05% HCOOH were used as eluents for the purification of all compounds. The following methods were applied: MV_NucleodurC18_001HCOOH_2to30 (0.0 – 13.0 min, linear, 2% CH_3CN to 30% CH_3CN, 13.0 – 15.0 min, isocratic, 30% CH_3CN, flow rate: 15 mL/min, 15.0 – 16.0 min, linear, 30% CH_3CN to 2% CH_3CN, 16 – 18 min, isocratic, 2% CH_3CN, T = 30 °C, flow rate: 15 mL/min) MV_NucleodurC18_001HCOOH_30to100 (0.0 – 13.0 min, linear, 30% CH_3CN to 100% CH_3CN, 13.0 – 15.0 min, isocratic, 100% CH_3CN, flow rate: 12

mL/min, 15.0 – 16.0 min, linear, 100% CH_3CN to 30% CH_3CN, 16 – 18 min, isocratic, 30% CH_3CN, T = 30 °C, flow rate: 12 mL/min), MV_NucleodurC18_001HCOOH_50to100 (0.0 – 13.0 min, linear, 50% CH_3CN to 100% CH_3CN, 13.0 – 15.0 min, isocratic, 100% CH_3CN, flow rate: 12 mL/min, 15.0 – 16.0 min, linear, 100% CH_3CN to 50% CH_3CN, 16 – 18 min, isocratic, 50% CH_3CN, T = 30 °C, flow rate: 12 mL/min) and MV_NucleodurC18_001HCOOH_70to100 (0.0 – 13.0 min, linear, 70% CH_3CN to 100% CH_3CN, 13.0 – 15.0 min, isocratic, 100% CH_3CN, flow rate: 12 mL/min, 15.0 – 16.0 min, linear, 100% CH_3CN to 70% CH_3CN, 16 – 18 min, isocratic, 70% CH_3CN, T = 30 °C, flow rate: 12 mL/min). High Resolution Mass Spectrometry (HRMS) was performed on a Waters GCT Premier Micromass (Direct Inlet (DI-EI)). Melting points were determined on a "Mel-Temp" melting-point apparatus (Electrothermal) and are given uncorrected. Chemicals were purchased from Sigma-Aldrich, Fisher Scientific, Merck, or Alfa Aesar. All compounds were used without further purification unless otherwise noted. For determination of concentration of the alkyl-lithium solution in n-hexane a procedure according to Kofron and Baclawski was used.[2] For this, a flame dried Schlenk-flask was charged with 2.0 mL absolute THF and 300 mg diphenyl acetic acid. The alkyl-Li solution was added dropwise under inert conditions. The equivalence point was indicated by a color change from colorless to yellow. To ensure a precise titration a triple determination was performed. The concentration of the Grignard solution was determined according to a procedure by Watson and Eastham.[3] A flame dried Schlenk-flask was charged with 200 mL absolute, degassed toluene and 20 mL abs. 2-butanol. This stock solution was stored under an atmosphere of argon over 3Å molecular sieves (stable over months). The concentration of this stock solution (c = 0.86M) was used as reference for the titration of Grignard-reagent solutions and was determined by NMR. For the titration, a flame dried and argon flushed 10 mL Schlenk-tube was charged with 1 mg ortho-phenantroline and 2 mL anhydrous toluene. 500 µL of the Grignard-reagent to be titrated were added under inert conditions and the solution was titrated with the standard butan-2-ol in toluene solution under inert conditions. The equivalence point was indicated by a color change from purple to yellow. The added moles of butan-2-ol are equal to the moles Grignard-reagent in the given aliquot. To ensure a precise titration a triple determination was performed. The concentration of alkyl zinc solutions was determined according to a procedure from Krasovskiy and Knochel.[4] A 10-mL round-bottom flask was charged with accurately weighed I_2 (1 mmol), fitted with a rubber septum and flushed with argon. A satd. LiCl-solution in THF (5 mL) was added. The resulting brown solution was cooled to 0 °C in an ice bath and the organometallic reagent was added dropwise via a syringe (1.0 mL, 0.01 mL

graduations) until the brown color disappeared. The amount consumed contains 1.0 eq of the RZnX reagent relative to iodine. DCM (EtOH stabilized) was first dried over P_2O_5, distilled, then dried over CaH_2 and distilled under an argon atmosphere before use. THF was dried by heating under reflux under an atmosphere of argon over Na, until benzophenone indicated dryness by a deep blue color and stored over 4Å molecular sieves in an amber glass Schlenk-flask under an argon atmosphere. Molecular sieves were activated by filling a 500 mL round-bottomed flask to one third of its volume with molecular sieves (Sigma-Aldrich, beads, 8-12 mesh) and heating the flask in a heating mantle (~150 °C) under oil pump vacuum for ~3 d, followed by cooling to room temperature under an atmosphere of argon. When working at a temperature of 0 °C, an ice-water bath served as the cooling agent, and -78 °C was achieved by a dry ice/acetone mixture.

2 General Procedures

2.1 Representative procedure for Negishi coupling

A two-neck round-bottom flask was charged with Zn-powder (2.0-3.0 eq). Subsequently the flask was evacuated, heated with a heat gun and after cooling to RT back flushed with argon three times. The Zn was suspended in abs. THF (6M) and 1,2-dibromoethane (5 mol%) was added. The mixture was heated to reflux temperature and cooled again to RT for three times. After the third cycle, TMSCl (5 mol%) was added and stirred at RT for 10 min. A solution of a corresponding halide (1.2-2.2 eq) in abs. THF (6M) was added slowly via a dropping funnel. A water bath was used to keep the reaction at RT. When addition was finished the reaction was stirred at RT for another 2 h. The conversion of halide was measured by GC-FID. For the GC-sample an aliquot of the reaction mixture was quenched with satd. NH_4Cl solution and extracted with DCM. The concentration of RZnX was determined as described in part 1.

A flame dried and argon flushed Schlenk-flask was charged with 3,5-dibromopyridine (**1**) (1.0 eq), $PdCl_2$(dppf) (1 mol%) and abs. THF (1M). The previously prepared organozinc solution (1.0 eq) was added to this orange suspension and the brown solution was stirred at 70 °C overnight (16 h). The reaction was in general stopped at about 95% conversion since the amount of dialkylation was rising. The catalyst was removed by filtration through a pad of silica gel and the product was eluted with EtOAc. The solvent was removed under reduced pressure and the crude product was purified via flash column chromatography.

2.2 Representative procedure for Cu-catalyzed Buchwald-Finkelstein reaction

A Schlenk-flask was charged with the corresponding pyridine-derivative (1.0 eq) which was dissolved in abs., degassed 1,4-dioxane (0.6M). *N*,*N*´-Dimethylethylenediamine (10 mol%), NaI (4.0 eq) and CuI (10 mol%) were added. The green suspension was stirred at 120 °C until full conversion was detected by GC-MS (24 h). Then the reaction was cooled to RT and quenched by the addition of satd. NH_4Cl solution. A light brown precipitate was formed, which was removed by filtration through a pad of Celite® (eluted with DCM). The phases were separated and the dark blue aqueous phase was extracted with DCM. The combined organic layers were dried over Na_2SO_4, filtered and the solvent was removed under reduced pressure. The crude product was purified via flash column chromatography.

2.3 Representative procedure for Knochel-Grignard reaction

A Schlenk-flask was charged with the corresponding pyridine-derivative (1.0 eq) dissolved in abs. THF (0.3M). The reaction mixture was cooled to -78 °C (0 °C when a bromide is used as starting material) and *i*PrMgCl.LiCl solution (1.5M in THF, 1.2 eq) was added dropwise. When complete metal-halogen exchange was detected by GC-MS (an aliquot of the reaction mixture was quenched with satd. NH_4Cl solution and extracted with DCM after 2 h), PinBO*i*Pr (1.15 eq) was added to the reaction mixture. The reaction was allowed to warm up in the cooling bath overnight and full conversion was detected by GC-MS (24 h). The reaction mixture was quenched by the addition of satd. NH_4Cl solution. The phases were separated, and the aqueous layer extracted with DCM. The combined organic layers were washed with brine, dried over Na_2SO_4 and concentrated in vacuo. If necessary, the crude product was purified via Kugelrohr distillation, sublimation or recrystallization.

2.4 Representative procedure for the synthesis of teraryls by consecutive double Suzuki-Coupling (1st step)

A flame dried Schlenk-flask was charged with 1.0 eq of the corresponding boronic acid derivative, 2.0 eq K_2CO_3, and 5 mol% $PdCl_2$(dppf). After drying in vacuo, a solution of 1.0 eq I/OTf or I/Br core fragment in abs., degassed DMF (~0.2M) was added. The reaction mixture was stirred at 80 °C until full conversion was detected by GC-MS or TLC. The typically brown suspension was filtered through a pad of SiO_2 (eluted with MeOH) and the filtrate was concentrated to dryness using a rotary evaporator. The crude product was purified via flash column chromatography or used in the next step without further purification.

2.5 Representative procedure for the synthesis of teraryls by consecutive double Suzuki-Coupling (2nd step)

Another flame dried Schlenk-flask was charged with 1.0-1.2 eq of the second boronic acid pinacol ester, 2.0 eq cesium carbonate (Cs_2CO_3), and 5 mol% $PdCl_2$(dppf). After drying in vacuo, a solution of the previously prepared intermediate in abs., degassed DMF (~0.2M) was added. The reaction mixture was stirred at 80 °C overnight. The typically black suspension was filtered through a pad of SiO_2 (eluted with MeOH) and after concentrating to dryness, the crude product was purified via flash column chromatography.

3 Experimental Procedures and Analytical Data for Building Block Synthesis

3.1 Synthesis of the Valine, Isoleucine and Phenylalanine building blocks

3.1.1 3-Bromo-5-isopropylpyridine

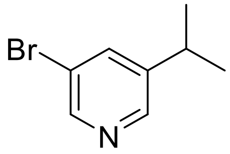

4

4 was prepared according to general procedure 2.1 from 4.62 g Zn-powder (70.6 mmol, 3.1 eq) in 10 mL abs. THF, 3.50 mL 2-iodopropane (6.0 g, 35.3 mmol, 1.6 eq) in 10 mL abs. THF, 5.33 g 3,5-dibromopyridine (**1**) (22.5 mmol, 1.0 eq) and 181 mg $PdCl_2$(dppf) (225 µmol, 1 mol%) in 20 mL abs. THF. As at 95% conversion (16 h) already 10% dialkylation was detected, the reaction was cooled down to RT, and the catalyst was removed by filtration through a pad of silica gel and the product was eluted with EtOAc (3 x 250 mL). The solvent was removed under reduced pressure and the brown oil was purified via flash column chromatography (200 g SiO_2, 5.0 x 22 cm, eluent: cyclohexane/EtOAc = 9/1, R_f = 0.30, UV and CAM).

Yield: 2.34 g (52%), yellow oil, $C_8H_{10}BrN$ [200.08 g/mol].

TLC: R_f = 0.30 (cyclohexane/EtOAc = 9/1, UV and CAM); **^{1}H NMR** (300 MHz, $CDCl_3$): δ = 8.63 (s, 1H; H^{Ar}), 8.42 (d, $^3J_{H,H}$ = 5.0 Hz, 1H; H^{Ar}), 7.18 (d, $^3J_{H,H}$ = 5.0 Hz, 1H; H^{Ar}), 3.30 (h, $^3J_{H,H}$ = 6.8 Hz, 1H; CH), 1.24 (d, $^3J_{H,H}$ = 6.9 Hz, 6H; CH_3) ppm; **^{13}C NMR** (76 MHz, $CDCl_3$, APT): δ = 156.1 (C_q; C^{Ar}), 152.0 (C^{Ar}), 148.7 (C^{Ar}), 123.0 (C_q; C^{Ar}), 121.9 (C^{Ar}), 32.7 (CH), 22.0 (CH_3) ppm;

GC-MS (EI, 70 eV; MT_50_S): t_R = 4.72 min; *m/z* (%): 203 (76) [M^+], 201 (77) [M^+], 186 (91) [$M^+{-}CH_3$], 184 (93) [$M^+{-}CH_3$], 120 (19) [M^+−Br], 104 (100) [$M^+{-}CH_3Br$]; **HRMS** (EI): calcd for [M^+]: 198.9997; found: 199.0001.

3.1.2 3-Isopropyl-5-(4,4,5,5-tetramethyl-1,3,2-dioxaborolan-2-yl)pyridine

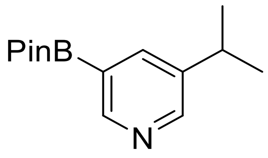

7

7 was prepared according to general procedure 2.3 from 1.67 g 3-bromo-5-isopropylpyridine (**4**) (8.35 mmol, 1.0 eq) in 20 mL abs. THF, 6.6 mL *i*PrMgCl.LiCl (1.51M in THF) (10.0 mmol, 1.2 eq) and 2.3 mL PinBO*i*Pr (2.10 g, 11.3 mmol, 1.4 eq). The crude product was purified via recrystallization from pentane.

Yield: 482 mg (21%), colorless powder, $C_{14}H_{22}BNO_2$ [247.15 g/mol].

m.p.$^{exp.}$ = 81-82 °C; **^{1}H NMR** (300 MHz, $CDCl_3$): δ = 8.82 (s, 1H; H^{Ar}), 8.54 (d, $^3J_{H,H}$ = 5.2 Hz, 1H; H^{Ar}), 7.18 (d, $^3J_{H,H}$ = 5.2 Hz, 1H; H^{Ar}), 3.62 (h, $^3J_{H,H}$ = 6.8 Hz, 1H; CH), 1.35 (s, 12H; CH_3), 1.21 (d, $^3J_{H,H}$ = 6.9 Hz, 6H; CH_3) ppm; **^{13}C NMR** (76 MHz, $CDCl_3$, APT): δ = 164.4 (C_q; C^{Ar}), 156.3 (C^{Ar}), 151.9 (C^{Ar}), 83.9 (C_q), 31.5 (CH), 24.9 (CH_3), 23.6 (CH_3) ppm;[1] **GC-MS** (EI, 70 eV; MT_50_XS): t_R = 14.91 min; *m/z* (%): 274 (16) [M^+], 232 (16) [$M^+{-}CH_3$], 147 (100) [$M^+{-}C_6H_{12}O$], 132 (29) [$M^+{-}C_6H_{13}O_2$]; **HRMS** (EI): calcd (*m/z*) for [M^+]: 247.1746; found: 247.1751.

3.1.3 3-Bromo-5-(*sec*-butyl)pyridine

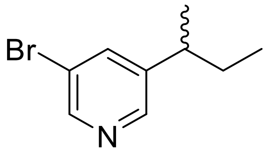

5

5 was prepared according to general procedure 2.1 from 4.26 g Zn-powder (65.2 mmol, 2.4 eq), in 10 mL abs. THF, 3.80 mL 2-iodobutane (6.0 g, 32.2 mmol, 1.2 eq) in 8 mL abs. THF, 6.44 g 3,5-dibromopyridine (**1**) (27.2 mmol, 1.0 eq) and 219 mg $PdCl_2$(dppf) (272 µmol, 1 mol%) in 20 mL abs. THF. The reaction was stopped at 98% conversion (24 h) by removal of the catalyst since

[1] Signal for the quaternary *ipso*-pyridine carbon (C_q; C^{Ar}) at the boronic acid pinacol ester function was not observed.

already by-products were detected. The catalyst was removed by filtration through a pad of silica gel and the product was eluted with EtOAc (3 x 250 mL). The solvent was removed under reduced pressure and the brown crude product was purified via flash column chromatography (250 g SiO_2, 7.0 x 17 cm, eluent: cyclohexane/EtOAc = 9/1, R_f = 0.30, UV and CAM).
Yield: 3.00 g (51%), yellow oil (contains ~10% *n*-isomer), $C_9H_{12}BrN$ [214.11 g/mol].

TLC: R_f = 0.30 (cyclohexane/EtOAc = 9/1, UV and CAM); **^{1}H NMR** (300 MHz, $CDCl_3$): δ = 8.65 (s, 1H; H^{Ar}), 8.41 (d, $^3J_{H,H}$ = 4.6 Hz, 1H; H^{Ar}), 7.13 (d, $^3J_{H,H}$ = 5.0 Hz, 1H; H^{Ar}), 3.17-3.05 (m, 1H; CH), 1.71-1.53 (m, 2H; CH_2), 1.21 (d, $^3J_{H,H}$ = 6.9 Hz, 3H; CH_3), 0.87 (t, $^3J_{H,H}$ = 7.4 Hz, 3H; CH_3) ppm; **^{13}C NMR** (76 MHz, $CDCl_3$, APT): δ = 155.3 (C_q; C^{Ar}), 152.1 (C^{Ar}), 148.5 (C^{Ar}), 123.6 (C_q; C^{Ar}), 122.5 (C^{Ar}), 39.5 (CH), 29.5 (CH_2), 20.0 (CH_3), 11.9 (CH_3) ppm; **GC-MS** (EI, 70 eV; MT_50_S): t_R = 5.04 min; *m/z* (%): 215 (71) [M^+], 201 (73) [M^+], 186 (96) [$M^+-C_2H_5$], 184 (97) [$M^+-C_2H_5$], 134 (21) [M^+−Br], 104 (100) [$M^+-C_2H_5Br$].

Analytical data are in accordance with those reported.[5]

3.1.4 3-(*sec*-Butyl)-5-(4,4,5,5-tetramethyl-1,3,2-dioxaborolan-2-yl)pyridine

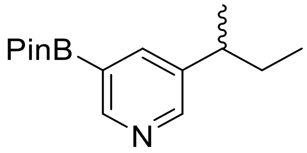

8

8 was prepared according to general procedure 2.3 from 2.39 g 3-bromo-5-(*sec*-butyl) pyridine (**5**) (11.2 mmol, 1.0 eq) in 20 mL abs. THF, 9.0 mL *i*PrMgCl.LiCl (1.51M in THF) (13.6 mmol, 1.2 eq) and 2.7 mL PinBO*i*Pr (2.46 g, 13.2 mmol, 1.2 eq). The crude product was purified via recrystallization from pentane.
Yield: 967 mg (32%), colorless powder, $C_{15}H_{24}BNO_2$ [261.17 g/mol].

m.p.$^{exp.}$ = 83-84 °C; **^{1}H NMR** (300 MHz, $CDCl_3$): δ = 8.82 (s, 1H; H^{Ar}), 8.53 (d, $^3J_{H,H}$ = 5.3 Hz, 1H; H^{Ar}), 7.14 (d, $^3J_{H,H}$ = 5.2 Hz, 1H; H^{Ar}), 3.46-3.34 (m, 1H; CH), 1.64-1.47 (m, 2H; CH_2), 1.34 (s, 12H; CH_3), 1.20 (d, $^3J_{H,H}$ = 6.9 Hz, 3H; CH_3), 0.82 (t, $^3J_{H,H}$ = 7.4 Hz, 3H; CH_3) ppm; **^{13}C NMR** (76 MHz, $CDCl_3$, APT): δ = 163.5 (C_q; C^{Ar}), 156.3 (C^{Ar}), 151.7 (C^{Ar}), 120.5

(C^{Ar}), 83.9 (C_q), 38.2 (CH), 31.3 (CH_2), 25.0 (CH_3), 24.9 (CH_3), 20.9 (CH_3), 12.1 (CH_3) ppm;[2] **GC-MS** (EI, 70 eV; MT_50_S): t_R = 6.29 min; *m/z* (%): 246 (14) [M^+], 177 (17) [M^+−C_6H_{12}], 161 (100) [M^+− $C_6H_{12}O$**HRMS** (EI): calcd (*m/z*) for [M^+]: 261.1903; found: 261.1914.

Analytical data are in accordance with those reported.[5]

3.1.5 3-Benzyl-5-bromopyridine

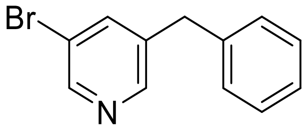

6

6 was prepared according to general procedure 2.1 from 1.15 g Zn-powder (17.5 mmol, 4.4 eq) in 3 mL abs. THF, 1.00 mL benzylbromide (1.50 g, 8.77 mmol, 2.2 eq) in 2 mL abs. THF, 950 mg 3,5-dibromopyridine (**1**) (4.01 mmol, 1.0 eq) and 32.3 mg $PdCl_2$(dppf) (40.1 µmol, 1 mol%) in 5 mL abs. THF. At 93% conversion (2 h) already 5% dibenzylation was detected, the reaction was cooled to RT, and the catalyst was removed by filtration through a pad of silica gel and the product was eluted with 100 mL EtOAc. The solvent was removed under reduced pressure and the brown oil was purified via flash column chromatography (100 g SiO_2, 2.5 x 20 cm, eluent: cyclohexane/EtOAc = 10/1 changing to cyclohexane/EtOAc = 5/1 when the product starts eluting, R_f = 0.40, UV and CAM).
Yield: 620 mg (61%), colorless oil, $C_{12}H_{10}BrN$ [248.12 g/mol].

TLC: R_f = 0.40 (cyclohexane/EtOAc = 5/1, UV and CAM); **^{1}H NMR** (300 MHz, $CDCl_3$): δ = 8.44 (d, $^4J_{H,H}$ = 1.9 Hz, 1H; H^{Ar}), 8.34 (s, 1H; H^{Ar}), 7.53 (s, 1H; H^{Ar}), 7.25-7.15 (m, 3H; H^{Ar}), 7.10 (d, $^3J_{H,H}$ = 7.0 Hz, 2H; H^{Ar}), 3.88 (s, 2H; CH_2) ppm; **^{13}C NMR** (76 MHz, $CDCl_3$, APT): δ = 148.9 (C^{Ar}), 148.4 (C^{Ar}), 139.0 (C_q; C^{Ar}) 138.9 (C^{Ar}), 138.5 (C_q; C^{Ar}), 129.0 (C^{Ar}), 126.9 (C^{Ar}), 120.9 (C_q; C^{Ar}), 38.8 (CH_2) ppm; **GC-MS** (EI, 70 eV; MT_50_S): t_R = 6.46 min; *m/z* (%): 247 (100) [M^+], 167 (100) [M^+−Br], 91 (36) [M^+−C_6H_5Br].

[2] Signal for the quaternary *ipso*-pyridine carbon (C_q; C^{Ar}) at the boronic acid pinacol ester function was not observed.

3.1.6 3-Benzyl-5-(4,4,5,5-tetramethyl-1,3,2-dioxaborolan-2-yl)pyridine

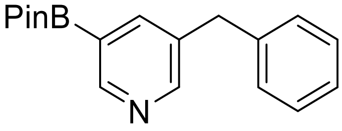

9

9 was prepared according to general procedure 2.3 from 860 mg 3-benzyl-5-bromopyridine (**6**) (3.47 mmol, 1.0 eq) in 10 mL absolute THF, 1.80 mL *i*PrMgCl.LiCl (1.5M in THF) (3.81 mmol, 1.1 eq) and 780 µL PinBO*i*Pr (3.81 mmol, 1.1 eq). The crude product was purified via recrystallization from pentane.

Yield: 187 mg (18%), colorless powder, $C_{18}H1_{22}BNO_2$ [295.19 g/mol].

m.p.$^{exp.}$ = 95-97 °C; **^{1}H NMR** (300 MHz, $CDCl_3$): δ = 8.80 (d, $^{4}J_{H,H}$ = 1.3 Hz, 1H; H^{Ar}), 8.53 (d, $^{4}J_{H,H}$ = 2.3 Hz, 1H; H^{Ar}), 7.99 (bs, 1H; H^{Ar}), 7.33-7.16 (m, 5H; H^{Ar}), 3.99 (s, 2H; CH_2), 1.34 (s, 12H; CH_3) ppm; **^{13}C NMR** (76 MHz, $CDCl_3$): δ = 152.0 (C^{Ar}), 150.9 (C^{Ar}), 144.0 (C^{Ar}), 139.6 (C_q; C^{Ar}), 136.6 (C_q; C^{Ar}), 129.0 (C^{Ar}), 128.9 (C^{Ar}), 126.8 (C^{Ar}), 84.6 (C_q), 39.2 (CH_2), 25.0 (CH_3) ppm;[3] **GC-MS** (EI, 70 eV; MT_50_S): t_R = 7.53 min; *m/z* (%): 295 (97) [M^+], 280 (100) [$M^+ - CH_3$], 238 (55) [$M^+ - C_4H_{13}$], 194 (80) [$M^+ - C_6H_{13}O$]; **HRMS** (EI): calcd (*m/z*) for [M^+]: 295.1747; found: 295.1749.

Analytical data are in accordance with those reported.[5]

3.2 Synthesis of the Aspartate building block

3.2.1 Methyl 2-(5-bromopyridin-3-yl)acetate

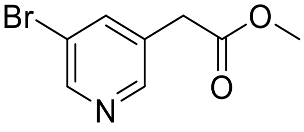

10

A flame dried, nitrogen flushed Schlenk-flask was charged with 91.7 mg $Pd_2(allyl)_2Cl_2$ (250 µmol, 2 mol%), 480 mg BINAP (770 µmol, 6 mol%), 2.97 g potassium 3-methoxy-3-oxopropanoate (19.0 mmol, 1.5 eq), 3.0 g 3,5-dibromopyridine (**1**) (12.7 mmol, 1.0 eq) and 153 mg DMAP

[3] Signal for the quaternary *ipso*-pyridine carbon (C_q; C^{Ar}) at the boronic acid pinacol ester function was not observed.

(1.25 mmol, 10 mol%) under nitrogen counter flow. The Schlenk-flask containing the reagents was evacuated and then nitrogen flushed. Subsequent to repeating this procedure two more times, 30 mL degassed mesitylene were added under inert conditions and the reaction mixture was stirred for 10 min at RT. Then the reaction mixture was heated to 140 °C and kept stirring until quantitative conversion of the starting material was detected by GC-MS. After full conversion (4 d) the brown suspension was brought to RT and directly purified via flash column chromatography (300 g SiO_2, 8.5 x 15 cm, eluent cyclohexane/EtOAc = 5/2, R_f = 0.26, UV and CAM)
Yield: 1.04 g (37%), yellow oil, $C_8H_8BrNO_2$ [230.06 g/mol].

TLC: R_f = 0.26 (cyclohexane//EtOAc = 5/2, UV and CAM); **^{1}H NMR** (300 MHz, $CDCl_3$): δ = 8.59 (d, $^4J_{H,H}$ = 2.0 Hz, 1H; H^{Ar}), 8.43 (d, $^4J_{H,H}$ = 1.5 Hz, 1H; H^{Ar}), 7.80 (s, 1H; H^{Ar}), 3.72 (s, 3H; CH_3), 3.62 (s, 2H; CH_2) ppm; **^{13}C NMR** (76 MHz, $CDCl_3$, APT): δ = 170.6 (C_q; CO), 149.9 (C^{Ar}), 148.6 (C^{Ar}), 139.6 (C^{Ar}), 131.4 (C_q; C^{Ar}), 120.8 (C_q; C^{Ar}), 52.6 (CH_3), 37.8 (CH_2); **GC-MS** (EI, 70 eV; MT_50_S): t_R = 5.50 min; *m/z* (%): 231 (90) [M^+], 229 (93) [M^+], 172 (100) [M^+−$COOCH_3$], 170 (100) [M^+−$COOCH_3$], 91 (44) [M^+−$COOCH_3$Br].

Analytical data are in accordance with those reported.[6]

3.2.2 Methyl 2-(5-iodopyridin-3-yl)acetate

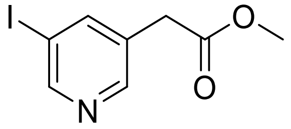

11a

11a was prepared according to general procedure 2.2 from 1.0 g **10** (4.35 mmol, 1.0 eq) in 10 mL abs., degassed 1,4-dioxane, 50 µL *N,N´*-dimethylethylenediamine (38.2 mg, 435 µmol, 10 mol%), 2.61 g NaI (17.4 mmol, 4.0 eq) and 82.8 mg CuI (435 µmol, 10 mol%). The yellow solid crude was purified via flash column chromatography (100 g SiO_2, 4.5 x 13 cm, eluent: cyclohexane//EtOAc = 4/1, R_f = 0.28, UV and CAM).
Yield: 743 mg (62%), colorless powder, $C_8H_8INO_2$ [277.06 g/mol].

TLC: R_f = 0.28 (cyclohexane//EtOAc = 4/1, UV and CAM); **m.p.**$^{exp.}$ = 41-43 °C; **^{1}H NMR** (300 MHz, $CDCl_3$): δ = 8.73 (d, $^4J_{H,H}$ = 1.4 Hz, 1H; H^{Ar}), 8.44 (bs, 1H; H^{Ar}), 7.98 (bs, 1H; H^{Ar}), 3.72 (s, 3H; CH_3), 3.58 (s, 2H; CH_2) ppm; **^{13}C NMR** (76 MHz, $CDCl_3$, APT): δ = 170.6 (C_q; CO),

154.7 (C^{Ar}), 149.0 (C^{Ar}), 145.1 (C^{Ar}), 131.7 (C_q; C^{Ar}), 93.4 (C_q; C^{Ar}), 52.6 (CH_3), 37.8 (CH_2) ppm; **GC-MS** (EI, 70 eV; MT_50_S): t_R = 5.95 min; *m/z* (%): 277 (100) [M^+], 218 (57) [M^+−COOCH].

3.2.3 Methyl 2-(5-(4,4,5,5-tetramethyl-1,3,2-dioxaborolan-2-yl)pyridin-3-yl)acetate

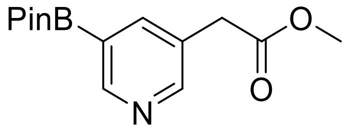

11

11 was prepared according to general procedure from 2.3 from 500 mg pyridine-derivative **11a** (1.80 mmol, 1.0 eq) in 10 mL abs., 1.32 mL *i*PrMgCl.LiCl solution (1.5M in THF) (288 mg, 1.99 mmol, 1.2 eq) and 425 µL PinBO*i*Pr (386 mg, 2.08 mmol, 1.2 eq). Due to the formation of isopropanolate during the reaction, partial trans-esterification occurred, resulting in an inseparable mixture of desired methyl ester (Me) and the isopropyl ester (*i*Pr) (17/3).

Yield: 498 mg (99%), colorless oil, $C_{14}H_{20}BNO_4$ [277.13 g/mol].

^{1}H NMR (300 MHz, $CDCl_3$): δ = 8.83 (s, 1H; H^{Ar}), 8.56 (d, $^4J_{H,H}$ = 1.8 Hz, 1H; H^{Ar}), 7.98 (s, 1H; H^{Ar}), 5.00 (sept, $^3J_{H,H}$ = 6.3 Hz, 0.2H; CH^{iPr}), 3.69 (s, 3H; CH_3^{Me}), 3.62 (s, 2H; CH_2^{Me}), 3.57 (s, 0.4H; CH_2^{iPr}), 1.24 (s, 1.3H; CH_3^{iPr}), 1.22 (s, 12H; CH_3) ppm; **^{13}C NMR** (76 MHz, $CDCl_3$, APT): δ = 171.3 (C_q; CO^{Me}), 170.3 (C_q; CO^{iPr}), 154.0 ($C^{Ar(Me)}$), 153.9 ($C^{Ar(iPr)}$), 152.4 ($C^{Ar(Me)}$), 152.4 ($C^{Ar(iPr)}$), 143.4 ($C^{Ar(iPr)}$), 143.4 ($C^{Ar(Me)}$), 129.5 (C_q; $C^{Ar(iPr)}$), 129.2 (C_q; $C^{Ar(Me)}$), 84.4 (C_q^{Me}), 82.9 (C_q^{iPr}), 68.8 (CH^{iPr}), 52.4 (CH_3^{Me}), 38.9 (CH_2^{iPr}), 38.4 (CH_2^{Me}), 25.0 (CH_3^{Me}), 24.7 (CH_3^{iPr}), 21.9 (CH_3^{iPr}) ppm;[4] **GC-MS** (EI, 70 eV; MT_50_S): $t_{R(Me)}$ = 6.82 min; *m/z* (%): 277 (50) [M^+], 262 (64) [M^+−CH_3], 218 (53) [M^+−$COOCH_3$], 178 (100) [M^+−$C_6H_{12}O$], 118 (47)) [M^+−$COOCH_3$]; $t_{R(iPr)}$ = 7.00 min; *m/z* (%): 305 (24) [M^+], 262 (14) [M^+−C_3H_7], 218 (100) [M^+−$COOC_3H_7$].

3.3 Synthesis of the Glutamate and Arginine building blocks

3.3.1 Methyl (*E*)-3-(5-bromopyridin-3-yl)acrylate

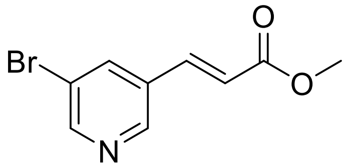

[4] Signal for the quaternary *ipso*-pyridine carbon (C_q; C^{Ar}) at the boronic acid pinacol ester function was not observed.

12a

A flame dried and argon flushed Schlenk-flask was charged with 2.0 g 3,5-dibromopyridine (**1**) (8.44 mmol, 1.0 eq), 760 µL methyl acrylate (727 mg, 8.44 mmol, 1.0 eq) and 30 mL degassed DMF. 190 mg $Pd(OAc)_2$ (844 µmol, 10 mol%) and 1.75 g K_2CO_3 (12.7 mmol, 1.5 eq) were added to this pale yellow solution. The yellow suspension was heated to 100 °C and stirred until full conversion was detected by GC-MS (48 h). The reaction mixture darkened during heating and turned dark brown after 24 h. When the starting material was fully converted the reaction was cooled to RT and diluted with 100 mL EtOAc. 100 mL H_2O were added and the dark brown emulsion was stirred for 20 min. A fine dark brown precipitate was formed, which was removed by filtration through a thin pad of Celite®. The phases of the filtrate were separated and the aqueous phase was extracted with EtOAc (2 x 50 mL). The combined organic layers were washed with brine (1 x 100 mL), dried over Na_2SO_4, filtered and the solvent was removed under reduced pressure. The brown solid crude was purified via flash column chromatography (250 g SiO_2, 6.0 x 15 cm, eluent: cyclohexane/EtOAc = 4/1, Rf = 0.31, UV and $KMnO_4$).

Yield: 1.30 g (71%), colorless powder, $C_9H_8BrNO_2$ [242.07 g/mol].

TLC: R_f = 0.31 (cyclohexane/EtOAc = 4/1, UV and $KMnO_4$); **m.p.**$^{exp.}$ = 120-121 °C; **^{1}H NMR** (300 MHz, $CDCl_3$): δ = 8.65 (d, $^4J_{H,H}$ = 4.8 Hz, 2H; H^{Ar}), 7.97 (s, 1H; H^{Ar}), 7.60 (d, $^3J_{H,H}$ = 16.1 Hz, 1H; CH), 6.50 (d, $^3J_{H,H}$ = 16.1 Hz, 1H; CH), 3.81 (s, 3H; CH_3) ppm; **^{13}C NMR** (76 MHz, $CDCl_3$, APT): δ = 166.4 (C_q; CO), 152.0 (CH), 147.7 (CH), 139.6 (C^{Ar}), 136.7 (C^{Ar}), 131.9 (C_q; C^{Ar}), 121.6 (C^{Ar}), 121.3 (C_q; C^{Ar}), 52.2 (CH_3) ppm; **GC-MS** (EI, 70 eV; MT_50_S): t_R = 6.10 min; *m/z* (%): 243 (34) [M^+], 241 (34) [M^+], 212 (100) [M^+−CH_3O], 212 (100) [M^+−CH_3O], 210 (100) [M^+−CH_3O], 184 (31) [M^+−$C_2H_3O_2$], 182 (31) [M^+−$C_2H_3O_2$], 103 (70) [M^+− $C_2H_3O_2Br$].

Analytical data are in accordance with those reported.[7]

3.3.2 Methyl 3-(5-bromopyridin-3-yl)propanoate

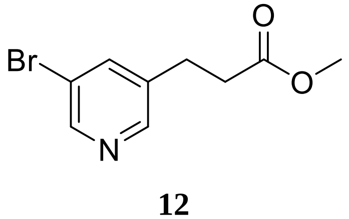

12

A 50 mL round-bottom flask was charged with 1.10 g pyridine derivative **12a** (4.54 mmol, 1.0 eq), 5.08 g p-tosyl hydrazide (27.3 mmol, 6.0 eq) and 3.71 g NaOAc.3H_2O (27.3 mmol, 6.0 eq). 30 mL THF were added to give a pale yellow suspension. The suspension was warmed to 70 °C and after 24 h an orange solution was formed. When quantitative conversion was detected by GC-MS the reaction mixture was cooled to RT and diluted with 100 mL DCM. The organic phase was washed with satd. $NaHCO_3$ solution (1 x 60 mL) and the aqueous phase was re-extracted with DCM (3 x 50 mL). The combined organic layers were washed with brine (1 x 100 mL), dried over Na_2SO_4, filtered and the solvent was removed under reduced pressure. The yellow solid crude was purified via flash column chromatography (100 g SiO_2, 5.0 x 20 cm, eluent: cyclohexane/EtOAc = 3/1, Rf = 0.27, UV and CAM).
Yield: 988 mg (89%), colorless powder, $C_9H_{10}BrNO_2$ [244.09 g/mol].

TLC: R_f = 0.27 (cyclohexane/EtOAc = 3/1, UV and CAM); **m.p.**$^{exp.}$ = 32-34 °C, (m.p.$^{lit.}$ = 33 °C);[8] **^{1}H NMR** (300 MHz, $CDCl_3$): δ = 8.54 (d, $^4J_{H,H}$ = 1.3 Hz, 1H; H^{Ar}), 8.41 (s, 1H; H^{Ar}), 7.74 (s, 1H; H^{Ar}), 3.67 (s, 3H; CH_3), 2.93 (t, $^3J_{H,H}$ = 7.5 Hz, 2H; CH_2), 2.64 (t, $^3J_{H,H}$ = 7.5 Hz, 2H; CH_2) ppm; **^{13}C NMR** (76 MHz, $CDCl_3$, APT): δ = 172.5 (C_q; CO), 148.4 (C^{Ar}), 147.5 (C^{Ar}), 139.4 (C^{Ar}), 138.2 (C_q; C^{Ar}), 120.9 (C_q; C^{Ar}), 52.0 (CH_3), 34.8 (CH_2), 27.7 (CH_2) ppm; **GC-MS** (EI, 70 eV; MT_50_S): t_R = 5.90 min; *m/z* (%): 245 (20) [M^+], 243 (20) [M^+], 230 (26) [M^+−CH_3], 228 (26) [M^+−CH_3], 215 (93) [M^+−OCH_3], 213 (100) [M^+−OCH_3], 185 (100) [M^+−$COOCH_3$], 183 (100) [M^+−$COOCH_3$].

Analytical data are in accordance with those reported.[8]

3.3.3 Methyl 3-(5-iodopyridin-3-yl)propanoate

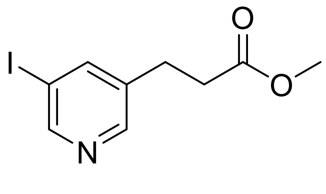

14a

14a was prepared according to general procedure 2.2 from 675 mg pyridine-derivative **12** (2.77 mmol, 1.0 eq) in 6 mL degassed 1,4-dioxane, 50 µL *N,N´*-dimethylethylenediamine (41 mg, 464 µmol, 20 mol%), 1.91 g NaI (12.7 mmol, 4.6 eq) and 66 mg CuI (347 µmol, 10 mol%). The

yellow oily crude was purified via flash column chromatography (75 g SiO_2, 4.0 x 18 cm, eluent: cyclohexane/EtOAc = 3/1, R_f = 0.32, UV and CAM).

Yield: 666 mg (83%), yellow powder, $C_9H_{10}INO_2$ [291.09 g/mol].

TLC: R_f = 0.32 (cyclohexane/EtOAc = 3/1, UV and CAM); **m.p.**$^{exp.}$ = 60-61 °C; **^{1}H NMR** (300 MHz, $CDCl_3$): δ = 8.68 (s, 1H; H^{Ar}), 8.42 (s, 1H; H^{Ar}), 7.92 (s, 1H; H^{Ar}), 3.67 (s, 3H; CH_3), 2.93 (t, $^3J_{H,H}$ = 7.5 Hz, 2H; CH_2), 2.64 (t, $^3J_{H,H}$ = 7.5 Hz, 2H; CH_2) ppm; **^{13}C NMR** (76 MHz, $CDCl_3$, APT): δ = 172.5 (C_q; CO), 153.3 (C^{Ar}), 147.8 (C^{Ar}), 145.0 (C^{Ar}), 138.4 (C_q; C^{Ar}), 93.5 (C_q; C^{Ar}), 52.0 (CH_3), 34.9 (CH_2), 27.7 (CH_2) ppm; **GC-MS** (EI, 70 eV; MT_50_S): t_R = 6.27 min; *m/z* (%): 291 (53) [M^+], 261 (100) [M^+−OCH_3], 231 (67) [M^+−$COOCH_3$]; **HRMS** (EI): calcd (*m*/*z*) for [M^+]: 290.9756; found: 290.9775.

3.3.4 Methyl 3-(5-(4,4,5,5-tetramethyl-1,3,2-dioxaborolan-2-yl)pyridin-3 yl)propanoate

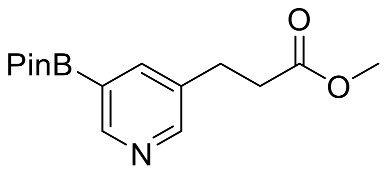

14

14 was prepared according to general procedure 2.3 from 576 mg **14a** (1.98 mmol, 1.0 eq) dissolved in 8 mL abs. THF, 1.9 mL *i*PrMgCl.LiCl solution (1.26M in THF) (2.2 mmol, 1.1 eq) and 500 µL PinBO*i*Pr (2.45 mmol, 1.2 eq). During the reaction isopropanolate was formed and caused a partial transesterification, resulting in an inseparable mixture of desired methyl ester (Me) and isopropyl ester (*i*Pr) (77/23).

Yield: 1.85 g (99%), pale yellow oil, $C_{15}H_{22}BNO_4$ [291.15 g/mol].

^{1}H NMR (300 MHz, $CDCl_3$): δ = 8.77 (bs, 1H; H^{Ar}), 8.51 (bs, 1H; H^{Ar}), 7.90 (bs, 1H; H^{Ar}), 4.98 (sept, $^3J_{H,H}$ = 6.2 Hz, 0.5H; CH^{iPr}), 3.66 (s, 1.8H; CH_3^{Me}), 2.95-2.91 (m, 2H, CH_2), 2.66-2.57 (m, 2H; CH_2), 1.34 (s, 12H; CH_3), 1.19 (d, $^3J_{H,H}$ = 6.2 Hz, 3.6H; CH_3^{iPr}) ppm; **^{13}C NMR** (76 MHz, $CDCl_3$): 172.9 (C_q, $CO^{(Me)}$), 172.0 (C_q, $CO^{(iPr)}$), 153.5 ($C^{Ar(Me)}$), 153.4 ($C^{Ar(iPr)}$), 152.1 ($C^{Ar(Me)}$), 152.0 ($C^{Ar(iPr)}$), 142.3 ($C^{Ar(Me)}$), 142.2 ($C^{Ar(iPr)}$), 135.2 ($C^{Ar(Me)}$), 135.2 ($C^{Ar(iPr)}$), 84.4 (C_q), 68.1 (CH^{iPr}), 51.9 (CH_3^{Me}), 35.9 (CH_2^{iPr}), 35.3 (CH_2^{Me}), 28.3 (CH_2^{iPr}), 28.2 (CH_2^{Me}), 25.0 (CH_3), 21.9 (CH_3^{iPr}) ppm;[5] **GC-MS** (EI, 70 eV; MT_50_S): $t_{R\ Me}$ = 7.07 min; *m/z* (%): 291 (50) [M^+], 232 (89)

[5] Signal for the quaternary *ipso*-pyridine carbon (C_q; C^{Ar}) at the boronic acid pinacol ester function was not observed.

[M^+−$COOCH_3$], 192 (100) [M^+−$C_6H_{12}O$], 132 (66) [M^+−$C_8H_{15}O_3$]; $t_{R\ iPr}$ = 7.29 min; *m/z* (%): 319 (11) [M^+], 277 (36) [M^+−C_3H_7], 232 (100) [M^+−$COOCH_3$], 132 (47) [M^+−$C_{10}H_{19}O_3$]; **HRMS** (EI): calcd (*m/z*) for [M_{Me}^+]: 291.1645; found: 291.1647; [M_{iPr}^+]: 319.1958; found: 319.1977.

3.3.5 (*E*,*Z*)-3-(5-Bromopyridin-3-yl)acrylonitrile

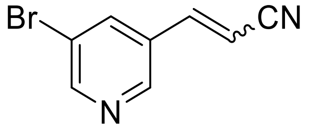

13a

A flame dried and argon flushed Schlenk-flask was charged with 2.37 g 3,5-dibromopyridine **1** (10.0 mmol, 1.0 eq), 2.07 g K_2CO_3 (15.0 mmol, 1.5 eq), 48.9 mg $Pd(OAc)_2$ (200 µmol, 2 mol%) and 35 mL degassed DMF. 660 µL acrylonitrile (531 mg, 10.0 mmol, 1.0 eq) were added to the pale yellow suspension. The reaction mixture was stirred at 90 °C under argon atmosphere for 16 h. The reaction was cooled to RT and diluted with 100 mL H_2O. The resulting solution was extracted with DCM (3 x 70 mL). The organic phase was dried over Na_2SO_4, filtered and the solvent was removed under reduced pressure. The crude product was purified via flash column chromatography (75 g SiO_2, 3.0 x 20 cm, eluent: cyclohexane/EtOAc = 4/1 → 2/1). The received mixture of stereoisomers was directly used in the subsequent reduction.
Yield: 1.08 g (58%), colorless powder, $C_8H_5BrN_2$ [209.05 g/mol].

(E): **TLC:** R_f = 0.43 (cyclohexane/EtOAc = 2/1, UV); **m.p.**$^{exp.}$ = 131-133 °C; **^{1}H NMR** (300 MHz, $CDCl_3$): δ = 8.71 (s, 1H; H^{Ar}), 8.60 (s, 1H; H^{Ar}), 7.92 (s, 1H; H^{Ar}), 7.35 (d, $^3J_{H,H}$ = 16.7 Hz, 1H; CH), 5.99 (d, $^3J_{H,H}$ = 16.7 Hz, 1H; CH) ppm; **^{13}C NMR** (76 MHz, $CDCl_3$): δ = 153.0 (C^{Ar}), 147.0 (C^{Ar}), 145.5 (CH), 136.0 (C^{Ar}), 130.9 (C_q; C^{Ar}), 121.4 (C_q; C^{Ar}), 116.9 (C_q; CN), 100.6 (CH) ppm; **GC-MS**: (EI, 70 eV; MT_50_S): t_R = 5.80 min; *m/z* (%): 210 (100) [M^+], 208 (100) [M^+], 184 (18) [M^+-CN], 182 (18) [M^+-CN], 129 (47) [M^+-Br]; **HRMS** (DI-EI) calcd (m/z) for [M^+]: 207.9628; found: 207.9639.

(Z): **TLC:** R_f = 0.26 (cyclohexane/EtOAc = 2/1, UV); **m.p.**$^{exp.}$ = 96-99 °C; **^{1}H NMR** (300 MHz, $CDCl_3$): δ = 8.82-8.65 (m, 2H; H^{Ar}), 8.43 (s, 1H; H^{Ar}), 7.10 (d, $^3J_{H,H}$ = 12.1 Hz, 1H; CH), 5.67 (d, $^3J_{H,H}$ = 12.1 Hz, 1H; CH) ppm; **^{13}C NMR** (76 MHz, $CDCl_3$): δ = 152.8 (C^{Ar}), 148.7 (C^{Ar}), 143.6 (CH), 137.4 (C^{Ar}), 130.9 (C_q; C^{Ar}), 121.2 (C_q; C^{Ar}), 116.2 (C_q; CN), 99.5 (CH) ppm; **GC-MS**: (EI, 70 eV; MT_50_S): t_R = 5.80 min; *m/z* (%): 210 (100) [M^+], 208 (100) [M^+], 184 (17) [M^+-CN],

182 (17) [M^+-CN], 129 (44) [M^+-Br]; **HRMS** (DI-EI) calcd (*m/z*) for [M^+]: 207.9636; found: 207.9639.

3.3.6 3-(5-Bromopyridin-3-yl)propanenitrile

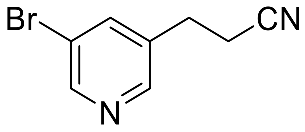

13

In a 250 mL round-bottom 2.83 g **13a** (13.5 mmol, 1.0 eq), 15.1 g *p*-tosyl hydrazide (81.2 mmol, 6.0 eq) and 6.68 g $NaOAc \cdot 3H_2O$ (81.2 mmol, 6.0 eq) were suspended in 80 mL THF. The suspension was stirred at 70 °C for 24 h. The solvent was removed under reduced pressure and the residue was diluted with 100 mL DCM and extracted with 150 mL satd. $NaHCO_3$. The aqueous phase was extracted with DCM (2 x 50 mL). The collected organic phase was dried over Na_2SO_4 and the solvent removed under reduced pressure. The crude product was purified via flash column chromatography (350 g SiO_2, 5.0 x 35 cm, eluent: cyclohexane/EtOAc = 3/1 → 2/1).
Yield: 2.46 g (86%), colorless oil, $C_8H_7BrN_2$ [211.06 g/mol].

TLC: R_f = 0.29 (cyclohexane/EtOAc = 1/1, UV and CAM); **^{1}H NMR** (300 MHz, $CDCl_3$): δ = 8.62 (d, $^4J_{H,H}$ = 1.5 Hz, 1H; H^{Ar}), 8.48 (s, 1H; H^{Ar}), 7.80 (s, 1H; H^{Ar}), 2.98 (t, $^3J_{H,H}$ = 7.2 Hz, 2H; CH_2), 2.68 (t, $^3J_{H,H}$ = 7.2 Hz, 2H; CH_2); **^{13}C NMR** (76 MHz, $CDCl_3$): δ = 149.5 (C^{Ar}), 147.4 (C^{Ar}), 139.3 (C^{Ar}), 135.5 (C_q; C^{Ar}), 121.2 (C_q; C^{Ar}), 118.2 (C_q; CN), 28.5 (CH_2), 19.0 (CH_2) ppm; **GC-MS**: (EI, 70 eV; MT_50_S): t_R = 5.82 min; *m/z* (%): 212 (46) [M^+], 210 (46) [M^+], 172 (100) [M^+-CH_2CN], 170 (100) [M^+-CH_2CN]; **HRMS** (DI-EI) calcd (*m/z*) for [M^+]: 209.9793; found: 209.9777.

3.3.7 3-(5-Iodopyridin-3-yl)propanenitrile

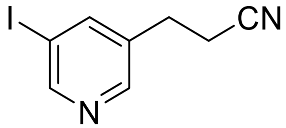

15a

15a was prepared according to general procedure 2.2 from 351 mg **13** (1.66 mmol, 1.0 eq) in 10 mL abs., degassed 1,4-dioxane, 35 µL *N*,*N*-dimethylethylenediamine (29.2 mg, 332 µmol, 20 mol%), 998 mg NaI (6.65 mmol, 4.0 eq) and 31.5 mg CuI (166 µmol, 10 mol%) The crude product was

purified via flash column chromatography (15 g SiO_2, 1.5 x 20 cm, eluent: cyclohexane/EtOAc = 2/1 → 6/5).
Yield: 386 mg (90%), pale yellow oil, $C_8H_7BrIN_2$ [257.97 g/mol].

TLC: R_f = 0.38 (cyclohexane/EtOAc = 1/1, UV); **^{1}H NMR** (300 MHz, $CDCl_3$): δ = 8.76 (s, 1H; H^{Ar}), 8.45 (s, 1H; H^{Ar}), 7.93 (s, 1H; H^{Ar}), 2.92 (t, $^3J_{H,H}$ = 7.2 Hz, 2H; CH_2), 2.65 (t, $^3J_{H,H}$ = 7.2 Hz, 2H; CH_2) ppm; **^{13}C NMR** (76 MHz, $CDCl_3$): δ = 155.0 (C^{Ar}), 148.2 (C^{Ar}), 144.2 (C^{Ar}), 135.5 (C_q; C^{Ar}), 118.2 (C_q; CN), 93.7 (C_q; C^{Ar}), 28.4 (CH_2), 19.0 (CH_2) ppm; **GC-MS**: (EI, 70 eV; MT_50_S): t_R = 6.20; *m/z* (%): 258 (100) [M^+], 218 (82) [M^+-CH_2CN], 131 (14) [M^+-I]; **HRMS** (DI-EI) calcd (*m/z*) for [M^+]: 257.9654; found: 257.9662.

3.3.8 3-(5-(4,4,5,5-Tetramethyl-1,3,2-dioxaborolan-2-yl)pyridin-3-yl)propanenitrile

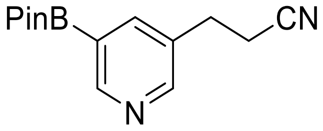

15

15 was prepared according to general procedure 2.3 from 386 mg **15a** (1.50 mmol, 1.0 eq) in 5 mL abs. THF, 1.33 mL *i*PrMgCl·LiCl solution (1.24M in THF) (1.65 mmol, 1.1 eq) and 333 µL PinBO*i*Pr (307 mg, 1.65 mmol, 1.1 eq). The residue was purified via Kugelrohr distillation (150 °C, $8 \cdot 10^{-2}$ mbar).
Yield: 240 mg (62%), colorless solid, $C_{14}H_{19}BN_2O_2$ [258.13 g/mol].

^{1}H NMR (300 MHz, $CDCl_3$): δ = 8.86 (s, 1H; H^{Ar}), 8.57 (d, $^4J_{H,H}$ = 1.8 Hz, 1H; H^{Ar}), 7.94 (s, 1H; H^{Ar}), 2.97 (t, $^3J_{H,H}$ = 7.4 Hz, 2H; CH_2), 2.65 (t, $^3J_{H,H}$ = 7.4 Hz, 2H; CH_2), 1.34 (s, 12H; CH_3) ppm; **^{13}C NMR** (76 MHz, $CDCl_3$, APT): δ = 154.2 (C^{Ar}), 151.6 (C^{Ar}), 142.3 (C^{Ar}), 132.9 (C_q; C^{Ar}), 118.5 (C_q; CN), 84.5 (C_q), 28.9 (CH_2), 25.0 (CH_3), 19.1 (CH_2) ppm;[6] **GC-MS**: (EI, 70 eV; MT_50_S): t_R = 6.90; *m/z* (%): 257 (97) [M^+], 243 (45) [M^+-CH_3], 173 (32) [M^+-C_6H_{12}], 159 (100) [M^+-C_6H_{11}O]; **HRMS** (DI-EI) calcd (*m/z*) for [M^+-H]: 257.1464; found: 257.1469.

[6] Signal for the quaternary *ipso*-pyridine carbon (C_q; C^{Ar}) at the boronic acid pinacol ester function was not observed.

3.4 Synthesis of the Tryptophan building block

3.4.1 3-(2-(1,3-Dioxolan-2-yl)ethyl)-5-bromopyridine

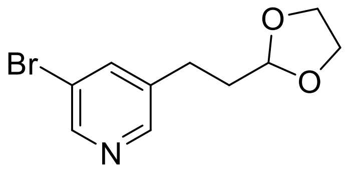

16a

A Schlenk flask was charged with 2.94 g Zn-powder (45.0 mmol, 1.5 eq). The flask was evacuated and heated to 70 °C for 30 min. After cooling to RT the flask was flushed with argon and evacuated again for three times. The Zn-powder was suspended in 20 mL abs., degassed DMA and 189 mg I_2 (750 µmol, 3 mol%) were added. The resulting mixture was heated to 70 °C and stirred until discoloration (5 min) and 3.60 mL 2-(2-bromoethyl)-1,3-dioxolane (5.43 g, 30 mmol, 1.0 eq) were added. The reaction mixture was stirred at 70 °C for another 16 h under Ar atmosphere. After cooling to RT the suspension was filtered into another flame dried Schlenk flask under argon. The conversion was determined (c = 0.80M) by titration as described in part 1.

A flame dried Schlenk flask was charged with 2.97 g 3,5-dibromopyridine (**1**) (16.8 mmol, 1.0 eq) and 387 mg $Pd(PPh_3)_4$ (336 µmol, 2 mol%) and dissolved in 20 mL abs., degassed THF. Then 21 mL previously prepared (2-(1,3-dioxolan-2-yl)ethyl)zinc(II) bromide solution in DMA (0.80M; 16.8 mmol, 1.0 eq) were added. The reaction mixture was stirred at 45 °C until full conversion was detected by GC-MS (3 h). The reaction was quenched by the addition of 100 mL satd. NH_4Cl solution. The mixture was extracted with DCM (2 x 150 mL). The combined organic layers were dried over Na_2SO_4, filtered and the solvent was removed under reduced pressure. The crude product was purified via flash column chromatography (120 g SiO_2, 4 x 18 cm, eluent: cyclohexane/EtOAc = 7/2 → 5/2).

Yield: 2.15 g (50%) colorless crystals, $C_{10}H_{12}BrNO_2$ [258.12 g/mol].

TLC R_f = 0.37 (cyclohexane/EtOAc = 2/1, UV and CAM); **m.p.**$^{exp.}$ = 39-40 °C; **^{1}H NMR** (300 MHz, $CDCl_3$): δ = 8.50 (d, $^4J_{H,H}$ = 1.8 Hz, 1H; H^{Ar}), 8.38 (s, 1H; H^{Ar}), 7.68 (s, 1H; H^{Ar}), 4.89 (t, $^3J_{H,H}$ = 4.4 Hz, 1H; CH), 4.05-3.78 (m, 4H; CH_2), 2.73 (t, $^3J_{H,H}$ = 16.1 Hz, 2H; CH_2), 2.03-1.91 (m, 2H; CH_2) ppm; **^{13}C NMR** (75.53 MHz, $CDCl_3$, APT): δ = 148.7 (C^{Ar}), 148.2 (C^{Ar}), 139.0 (C_q; C^{Ar}), 138.6 (C^{Ar}), 120.7 (C_q; C^{Ar}), 103.3 (CH), 65.2 (CH_2), 34.9 (CH_2), 26.8 (CH_2) ppm; **GC-MS**

(EI, 70 eV: MT_50_S): t_R = 6.39 min; *m/z* (%): 257 [M^+], 259 [M^+], 184 [M^+–$C_3H_5O_2$]; **HRMS** (EI): calcd (*m/z*) for [M^+–H]: 257.0051; found: 257.0054.

3.4.2 3-((5-Bromopyridin-3-yl)methyl)-1*H*-indole

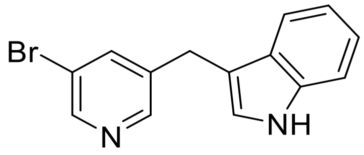

16

In a 250 mL round-bottom flask 4.00 g **16a** (15.5 mmol, 1.0 eq) were dissolved in a mixture of 3.04 mL phenylhydrazine (3.55 g, 31.0 mmol, 2.0 eq), 12 mL H_2SO_4 (96% w/w) and 120 mL THF. The orange solution was stirred at 80 °C for 4 h. The reaction mixture was cooled to RT and neutralized with 200 mL satd. Na_2CO_3 solution. The neutral solution was extracted with DCM (1 x 100 mL, 2 x 50 mL). The combined organic layers were dried over Na_2SO_4 and concentrated under reduced pressure. The orange oil was purified via flash column chromatography (90 g SiO_2, 4 x 18 cm, eluent: cyclohexane/EtOAc = 4/1 → 3/1), followed by recrystallization from cyclohexane/EtOAc = 8/1 (90 mL).

Yield: 2.25 g (51%), pale orange powder, $C_{14}H_{11}BrN_2$ [287.15 g/mol]

TLC R_f = 0.38 (cyclohexane/EtOAc = 2/1, UV and CAM); **m.p.**$^{exp.}$ = 157-158 °C; **^{1}H NMR** (300 MHz, $CDCl_3$): δ = 8.52 (s, 2H; H^{Ar}), 8.24 (bs, 1H; NH), 7.70 (s, 1H; H^{Ar}), 7.47 (d, $^3J_{H,H}$ = 7.9 Hz, 1H; H^{Ar}), 7.38 (d, $^3J_{H,H}$ = 8.1 Hz, 1H; H^{Ar}), 7.22 (t, $^3J_{H,H}$ = 7.5 Hz, 1H; H^{Ar}), 7.11 (t, $^3J_{H,H}$ = 7.4 Hz, 1H; H^{Ar}), 6.97 (s, 1H; H^{Ar}), 4.10 (s, 2H; CH_2) ppm; **^{13}C NMR** (75.53 MHz, $CDCl_3$, APT): δ = 148.6 (C^{Ar}), 148.2 (C^{Ar}), 138.9 (C^{Ar}), 138.8 (C_q; C^{Ar}), 136.6 (C_q; C^{Ar}), 127.0 (C_q; C^{Ar}), 122.7 (C^{Ar}), 122.6 (C^{Ar}), 120.9 (C_q; C^{Ar}), 119.9 (C^{Ar}), 118.8 (C^{Ar}), 113.7 (C_q; C^{Ar}), 111.5 (C^{Ar}), 28.6 (CH_2) ppm; **GC-MS** (EI, 70 eV: MT_50_S): t_R = 8.29 min; *m/z* (%): 288 [M^+], 286 [M^+], 207 [M^+–Br], 155 [M^+–C_5H_3BrN], 130 [M^+–C_9H_8N]; **HRMS** (EI): calcd (*m/z*) for [M^+–H]: 286.0106; found: 286.0106.

3.4.3 3-((5-(4,4,5,5-Tetramethyl-1,3,2-dioxaborolan-2-yl)pyridin-3-yl)methyl)-1*H*-indole

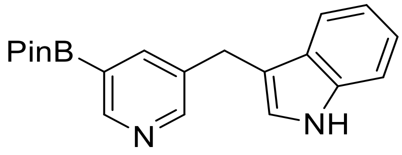

17

A flame dried Schlenk flask was charged with 1.86 g B_2Pin_2 (7.34 mmol, 1.1 eq), 285 mg Pd(dppf)Cl_2 (50.0 µmol, 5 mol%), 891 mg KOAc (9.09 mmol, 1.3 eq) and 2.00 g 3-((5-bromopyridin-3-yl)methyl)-1*H*-indole **16** (6.99 mmol, 1.0 eq). After drying in vacuum for 30 min, 22 mL abs., degassed 1,4-dioxane were added and the reaction mixture was stirred for 16 h at 80 °C. The black suspension was cooled to RT, diluted with 10 mL MeOH and 100 mL satd. NH_4Cl solution. The resulting mixture was extracted with EtOAc (1 x 100 mL, 2 x 50 mL). The combined organic layers were dried over Na_2SO_4, filtered and concentrated under reduced pressure to dryness. The residue was suspended in 100 mL EtOAc, filtered and the filter cake was washed with 50 mL EtOAc. The filtrate was concentrated to dryness using a rotary evaporator. The residue was dissolved in 8 mL DCM and slowly dropped into vigorously stirred 400 mL *n*-pentane. The resulting suspension was filtered and the collected solid was dried under reduced pressure.

Yield: 2.16 g (93%), pale grey powder, $C_{20}H_{23}BN_2O_2$ [334.23 g/mol]

m.p.$^{exp.}$ = 53-55°C; **^{1}H NMR** (300 MHz, $CDCl_3$): δ = 8.81 (s, 1H; H^{Ar}), 8.64 (s, 1H; H^{Ar}), 8.34 (bs, 1H; NH), 8.00 (s, 1H; H^{Ar}), 7.51 (d, $^3J_{H,H}$ = 7.7 Hz, 1H; H^{Ar}), 7.35 (d, $^3J_{H,H}$ = 8.0 Hz, 1H; H^{Ar}), 7.18 (t, $^3J_{H,H}$ = 7.4 Hz, 1H; H^{Ar}), 7.08 (t, $^3J_{H,H}$ = 7.3 Hz, 1H; H^{Ar}), 6.88 (s, 1H; H^{Ar}), 4.09 (s, 2H; CH_2) 1.34 (s, 12H; CH_3) ppm; **^{13}C NMR** (75.53 MHz, $CDCl_3$, APT): δ = 153.2 (C^{Ar}), 152.4 (C^{Ar}), 142.7 (C^{Ar}), 136.6 (C_q; C^{Ar}), 136.0 (C_q; C^{Ar}), 127.2 (C_q; C^{Ar}), 122.7 (C^{Ar}), 122.3 (C^{Ar}), 119.6 (C^{Ar}), 119.0 (C^{Ar}), 114.8 (C_q; C^{Ar}), 111.3 (C^{Ar}), 84.3 (C_q), 29.1 (CH_2), 25.0 (CH_3) ppm;[7] **GC-MS** (EI, 70 eV: MT_50_S): t_R = 9.91 min; *m/z* (%): 334 [M^+], 207 [M^+–$C_6H_{12}BO_2$], 130 [M^+–$C_{11}H_{15}BO_2$]; **HRMS** (MALDI): calcd for [M^++H]: 335.1935; found: 335.1934.

3.5 Synthesis of the Asparagine building block

3.5.1 2-(5-(4,4,5,5-Tetramethyl-1,3,2-dioxaborolan-2-yl)pyridin-3-yl)acetamide

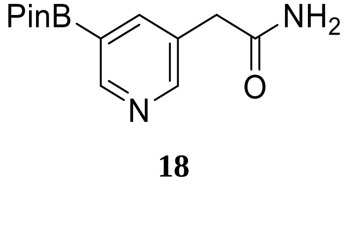

18

[7] Signal for the quaternary *ipso*-pyridine carbon (C_q; C^{Ar}) at the boronic acid pinacol ester function was not observed.

A 15 mL "Ace pressure tube®, front seal" (Aldrich Z181099) with a "Duro-Silicone O-ring" was charged with 100 mg compound mixture **11** (361 µmol, 1.0 eq), 7.1 mg KCN (108 µmol, 30 mol%) and 5 mL ammonia solution (7M in MeOH). The flask was sealed, and the mixture was stirred at 50 °C for 9 d. The solvent was evaporated in vacuo and the brown residue was purified via sublimation (210 °C, $1.4 \cdot 10^{-2}$ mbar).

Yield: 55 mg (61%), colorless solid, $C_{13}H_{19}BN_2O_3$ [262.11 g/mol].

^{1}H NMR (300 MHz, $CDCl_3$): δ = 8.85 (s, 1H; H^{Ar}), 8.61 (s, 1H; H^{Ar}), 8.03 (s, 1H; H^{Ar}), 5.82 (bs, 1H; $CONH_2$), 5.75 (bs, 1H; $CONH_2$), 3.57 (s, 2H; CH_2), 1.34 (s, 12H; CH_3) ppm; **^{13}C NMR** (76 MHz, $CDCl_3$): δ = 172.0 (C_q; CO), 153.6 (C^{Ar}), 151.9 (C^{Ar}), 143.9 (C^{Ar}), 130.4 (C_q; C^{Ar}), 84.6 (C_q), 40.2 (CH_2), 25.0 (CH_3) ppm;[8] **GC-MS** (EI, 70 eV; MT_50_S): t_R = 7.63 min; *m/z* (%): 262 (43) [M^+], 247 (70) [$M^+{-}CH_3$], 203 (80) [$M^+{-}C_2H_5NO$], 163 (100) [$C_8H_{10}BNO_2^+$], 146 (9) [$C_7H_5BNO_2^+$], 119 (50) [$C_7H_5NO^+$]; **m.p.**$^{exp.}$ = 174-179 °C; **HRMS** (EI): calcd (*m/z*) for [M^+]: 262.1491; found: 262.1501.

Analytical data are in accordance with those reported.[5]

3.6 Synthesis of the Histidine building block

3.6.1 1-Trityl-1*H*-imidazole-4-carbaldehyde

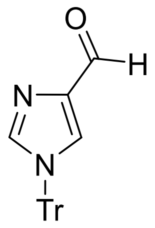

19

In a 100 mL round-bottom flask equipped with stirring bar 2.35 g 1*H*-imidazole-4-carbaldehyde (24.4 mmol, 1.0 eq) and 7.50 g trityl chloride (26.9 mmol, 1.1 eq) were suspended in 60 mL MeCN. 5.7 mL Et_3N (41.1 mmol, 1.7 eq) were added upon which the suspension turned yellow. The reaction mixture was stirred at RT for 12 h and full conversion was detected via TLC. 60 mL H_2O and 10 mL cyclohexane were added, the reaction mixture was filtered and the filter cake was rinsed

[8] Signal for the quaternary *ipso*-pyridine carbon (C_q; C^{Py}) at the boronic acid pinacol ester function was not observed.

with H_2O (3x 20 mL). The beige solid so obtained was purified via silica gel filtration (75 g SiO_2, 20 x 5 cm, eluent: cyclohexane/EtOAc = 2/1 → 1/1).
Yield: 7.33 g (89%), colourless solid, $C_{23}H_{18}N_2O$ [338.41 g/mol].

TLC: R_f = 0.29 (cyclohexane/EtOAc = 2/1, UV and CAM); **m.p.**$^{exp.}$ = 184-186 °C; **^{1}H NMR** (300 MHz, $CDCl_3$): δ = 9.87 (s, 1H; COH), 7.61 (d, $^4J_{H,H}$ = 1.1 Hz, 1H; H^{Ar}), 7.53 (d, $^4J_{H,H}$ = 1.1 Hz, 1H; H^{Ar}), 7.37-7.35 (m, 9H; 9x H^{Ar}), 7.12-7.09 (m, 6H; 6x H^{Ar}) ppm; **^{13}C NMR** (76 MHz, $CDCl_3$, APT): δ = 186.6 (C_q; CO), 141.7 (C_q; 3x C^{Ar}), 141.0 (C_q; C^{Ar}), 140.8 (C^{Ar}), 129.8 (6x C_{Ar}), 128.7 (3x C_{Ar}), 128.5 (6x C_{Ar}), 127.0 (C^{Ar}), 76.5 (C_q) ppm.
Analytical data are in accordance with those reported.[9]

3.6.2 (5-Bromopyridin-3-yl)(1-trityl-1*H*-imidazol-4-yl)methanol

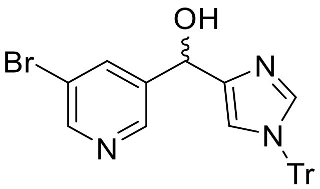

20a

In a flame-dried, Ar flushed Schlenk flask equipped with stirring bar 3.15 g 3,5-dibromopyridine (**1**) (13.3 mmol, 1.5 eq) were dissolved in 40 mL abs. THF. The colourless solution was cooled to 0 °C via an ice-water bath and 11 mL *i*PrMgCl·LiCl solution (1.3M in THF) (14.3 mmol, 1.6 eq) were added dropwise upon which the reaction mixture turned orange. The solution was stirred at 0 °C until full conversion of the metal-halogen exchange was detected via GC-MS (30 min). The reaction mixture was then cooled to -78 °C via an acetone-dry ice bath. 3.01 g **19** (8.89 mmol, 1.0 eq) were added and the resulting colourless suspension was allowed to warm to RT overnight. Full conversion was detected via TLC and the reaction mixture was poured onto 50 mL NH_4Cl. The aqueous phase was extracted with DCM (3x 50 mL) and the combined organic phases were dried over Na_2SO_4 and filtered. The filter cake was rinsed with DCM (3x 5 mL) and the filtrate was concentrated under reduced pressure to give a yellow oil. The crude product was purified via flash column chromatography (250 g SiO_2, 25 x 5 cm, eluent: EtOAc → MeOH).
Yield: 2.97 g (68%), orange solid, $C_{28}H_{23}BrN_3O$ [496.41 g/mol].

TLC: R_f = 0.09 (EtOAc, UV and CAM); **^{1}H NMR** (300 MHz, [D6]DMSO): δ = 8.56-8.53 (m, 2H; 2x H^{Ar}), 7.92 (m, 1H; H^{Ar}), 7.41-7.39 (m, 9H; 9x H^{Ar}), 7.32 (s, 1H; H^{Ar}), 7.10-7.08 (m, 6H; 6x

H^{Ar}), 6.85 (s, 1H; H^{Ar}), 5.98 (d, $^3J_{H,H}$ = 5.1 Hz, 1H; OH), 5.63 (d, $^3J_{H,H}$ = 5.1 Hz, 1H; CH) ppm; **^{13}C NMR** (76 MHz, [D6]DMSO, APT): δ = 148.5 (C^{Ar}), 146.8 (C^{Ar}), 143.5 (C^{Ar}), 142.2 (C^{Ar}), 142.2 (C_q; 3x C^{Ar}), 138.3 (C_q; C^{Ar}), 136.7 (C_q; C^{Ar}), 129.2 (6x C^{Ar}), 128.2 (6x C^{Ar}), 128.0 (3x C^{Ar}), 119.7 (C_q; C^{Ar}), 118.2 (C^{Ar}), 74.6 (C_q), 67.1 (CH) ppm.

3.6.3 **(5-Bromopyridin-3-yl)(1-trityl-1*H*-imidazol-4-yl)methyl acetate**

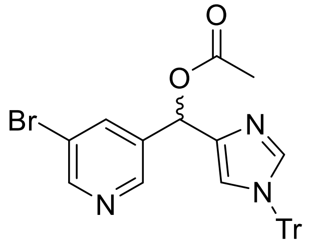

18

In a 100 mL round-bottom flask equipped with stirring bar 2.6 g **20a** (5.24 mmol, 1.0 eq) were suspended in 40 mL DCM. 550 µL Pyridine (6.81 mmol, 1.3 eq), 600 µL Ac_2O (6.35 mmol, 1.2 eq) and 65 mg DMAP (0.53 mmol, 10 mol%) were added and the resulting yellow solution was stirred at RT for 30 min. Full conversion was detected via TLC. 40 mL sat. $NaHCO_3$ were added, the reaction mixture was transferred to a separatory funnel and the aqueous phase was extracted with DCM (2x 40 mL). The combined organic phases were dried over Na_2SO_4 and filtered. The filter cake was rinsed with DCM (3x 10 mL) and the filtrate was concentrated under reduced pressure. The crude product was purified via flash column chromatography (150 g SiO_2, 30 x 5 cm, eluent: cyclohexane/EtOAc = 2/1 → 1/1).

Yield: 2.34 g (83%), colourless solid, $C_{30}H_{24}BrN_3O_2$ [538.45 g/mol].

TLC: R_f = 0.29 (cyclohexane/EtOAc = 1/1, UV and CAM); **m.p.**$^{exp.}$ = 43-47 °C; **^{1}H NMR (**300 MHz, $CDCl_3$): δ = 8.59-8.55 (m, 2H; 2x H^{Ar}), 7.97 (m, 1H; H^{Ar}), 7.58 (s, 1H; H^{Ar}), 7.36-7.35 (m, 9H; 9x H^{Ar}), 7.11-7.08 (m, 6H; 6x H^{Ar}), 6.82 (m, 2H; H^{Ar} and CH), 2.14 (s, 3H; CH_3) ppm; **^{13}C NMR (**76 MHz, $CDCl_3$, APT): δ = 169.9 (C_q; CO), 150.7 (C^{Ar}), 147.1 (C^{Ar}), 141.6 (C_q; 3x C^{Ar}), 139.3 (C^{Ar}), 137.9 (C^{Ar}), 137.4 (C_q; C^{Ar}), 136.3 (C_q; C^{Ar}), 129.7 (6x C^{Ar}), 128.6 (3x C^{Ar}), 128.5 (6x C^{Ar}), 120.8 (C^{Ar}), 120.7 (C_q; C^{Ar}), 76.5 (C_q), 68.7 (CH), 21.3 (CH_3) ppm; **HRMS (**MALDI): calcd (*m*/*z*) for [M^+]: 537.1052; found: 537.1055.

3.6.4 3-Bromo-5-((1-trityl-1*H*-imidazol-4-yl)methyl)pyridine

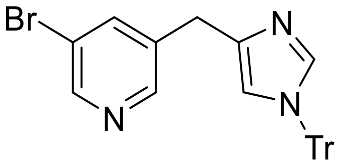

21

A flame-dried, Ar flushed Schlenk flask equipped with stirring bar was charged with 250 mg **20** (0.46 mmol, 1.0 eq), 70 µL *t*-BuOH (2.75 mmol, 1.6 eq) and 5 mL abs. THF. The resulting colourless solution was cooled to 0 °C in an ice-water bath and 14 mL freshly prepared SmI_2 solution (0.1M in THF) (1.40 mmol, 3.0 eq) were added over 10 min. Upon addition, the reaction mixture immediately turned yellow and after 10 mL the deep blue colour persisted. The reaction mixture was stirred at RT for 1 h and full conversion was detected via TLC. 20 mL H_2O were added, the aqueous phase was extracted with DCM (2x 30 mL) and the combined organic phases were dried over Na_2SO_4 and filtered. The filter cake was rinsed with DCM (3x 15 mL) and the solvent was removed under reduced pressure. The crude product was purified via flash column chromatography (13 g SiO_2, 10 x 2.5 cm, eluent: cyclohexane/EtOAc = 1/1).

Yield: 144 mg (65%), orange solid, $C_{28}H_{22}BrN_3$ [480.41 g/mol].

TLC: R_f = 0.17 (cyclohexane/EtOAc = 1/1, UV and CAM); **m.p.**$^{exp.}$ = 160-165 °C; **^{1}H NMR (**300 MHz, $CDCl_3$): δ = 8.49 (d, $^4J_{H,H}$ = 1.7 Hz, 1H; H^{Ar}), 8.39 (d, $^4J_{H,H}$ = 1.4 Hz, 1H; H^{Ar}), 7.71 (m, 1H; H^{Ar}), 7.51 (s, 1H; H^{Ar}), 7.36-7.34 (m, 9H; 9x H^{Ar}), 7.13-7.10 (m, 6H; 6x H^{Ar}), 6.60 (s, 1H; H^{Ar}), 3.92 (s, 2H; CH_2) ppm; **^{13}C NMR (**76 MHz, $CDCl_3$, APT): δ = 148.9 (C^{Ar}), 148.2 (C^{Ar}), 142.0 (C_q; 3x C^{Ar}), 139.0 (C^{Ar}), 138.8 (C^{Ar}), 138.1 (C_q; C^{Ar}), 137.3 (C_q; C^{Ar}), 129.8 (6x C^{Ar}), 128.4 (3x C^{Ar}), 128.4 (6x C^{Ar}), 120.8 (C_q; C^{Ar}), 119.5 (C^{Ar}), 76.0 (C_q), 31.2 (CH_2) ppm; **HRMS** (MALDI): calcd (*m*/*z*) for [M^+]: 479.0997; found: 479.1001.

3.6.5 (5-(4,4,5,5-Tetramethyl-1,3,2-dioxaborolan-2-yl)pyridin-3-yl)(1-trityl-1*H*-imidazol-4-yl)methyl acetate

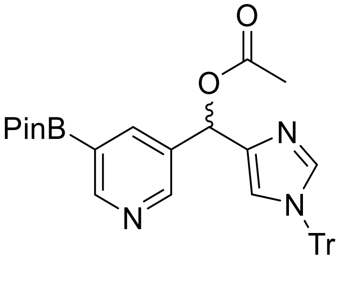

22

In a flame-dried, Ar flushed Schlenk flask equipped with stirring bar 1.00 g **20** (1.86 mmol, 1.0 eq), 708 mg B_2Pin_2 (2.79 mmol, 1.5 eq), 364 mg KOAc (3.71 mmol, 2.0 eq) and 66 mg $PdCl_2$(dppf) (90.2 µmol, 5 mol%) were suspended in 20 mL abs., degassed 1,4-dioxane. The reaction mixture was stirred at 80 °C for 15 h and full conversion was detected via TLC. The reaction mixture was filtered through a pad of celite and the filter cake was rinsed with EtOAc (3x 10 mL). The solvent was removed under reduced pressure to give a brown oil. The crude product was dissolved in a minimum of EtOAc (4 mL) and a colourless solid was precipitated by the addition of pentane (20 mL). The solid was collected by filtration, washed with pentane (3x 5 mL) and dried under vacuum. The filtrate was concentrated under reduced pressure and the procedure was repeated two more times, yielding a total of 666 mg desired product.

Yield: 666 mg (62%), colourless solid, $C_{36}H_{36}BN_3O_4$ [585.51 g/mol].

TLC: R_f = 0.05 (DCM/MeOH = 5/1, UV and CAM); **m.p.**$^{exp.}$ = 56-60 °C; **^{1}H NMR (**300 MHz, $CDCl_3$): δ = 8.87 (s, 1H; H^{Ar}), 8.78 (s, 1H; H^{Ar}), 8.32 (s, 1H; H^{Ar}), 7.43 (s, 1H; H^{Ar}), 7.38-7.28 (m, 9H; 9x H^{Ar}), 7.13-7.05 (m, 6H; 6x H^{Ar}), 6.83-6.81 (m, 2H; H^{Ar} and CH), 2.12 (s, 3H; CH_3), 1.34 (s, 12H; 4x CH_3) ppm; **^{13}C NMR (**76 MHz, $CDCl_3$, APT): δ = 170.0 (C_q; CO), 151.7 (C^{Ar}), 148.0 (C^{Ar}), 144.1 (C^{Ar}), 142.1 (C_q; 3x C^{Ar}), 139.9 (C^{Ar}), 138.2 (C_q; C^{Ar}), 135.9 (C_q; C^{Ar}), 129.8 (6x C^{Ar}), 128.4 (3x C^{Ar}), 128.3 (6x C^{Ar}), 120.7 (C^{Ar}), 84.8 (2x C_q), 75.9 (C_q), 69.9 (CH), 25.0 (2x CH_3), 25.0 (2x CH_3), 21.3 (CH_3) ppm; **HRMS (**MALDI): calcd (*m*/*z*) for [M^+]: 585.2799; found: 585.2802.

3.6.6 3-(4,4,5,5-Tetramethyl-1,3,2-dioxaborolan-2-yl)-5-((1-trityl-1*H*-imidazol-4-yl)methyl)pyridine

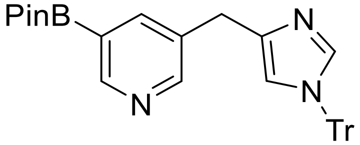

23

A flame-dried, Ar flushed Schlenk flask equipped with stirring bar was charged with 166 mg **22** (0.28 mmol, 1.0 eq), 40 µL *t*-BuOH (0.43 mmol, 1.5 eq) and 5 mL abs. THF. The resulting yellow solution was cooled to 0 °C in an ice-water bath and 10.6 mL freshly prepared SmI_2 solution (0.08M in THF) (0.85 mmol, 3.0 eq) were added over 10 min. The deep blue reaction mixture was stirred at 0 °C for 1 h and the reaction was quenched by addition of 20 mL EtOAc upon which the colour changed from deep blue to orange. 20 mL sat. Rochelle-salt solution were added, and the resulting

emulsion was stirred vigorously until the organic layer was clear orange (4 h). The two phases were separated in a separatory funnel and the organic phase was dried over Na_2SO_4 and filtered. The filter cake was rinsed with EtOAc (3x 10 mL) and the solvent was removed under reduced pressure to give an orange oil. The crude product was purified via recrystallization from 3 mL EtOAc.
Yield: 41 mg (28%), orange solid, $C_{34}H_{34}BN_3O_2$ [527.48 g/mol].

TLC: R_f = 0.05 (DCM/MeOH = 5/1, UV and CAM); **m.p.**$^{exp.}$ = 93-96 °C; **^{1}H NMR (**300 MHz, $CDCl_3$): δ = 8.74 (s, 1H; H^{Ar}), 8.52 (s, 1H; H^{Ar}), 7.95 (s, 1H; H^{Ar}), 7.43 (s, 1H; H^{Ar}), 7.39-7.27 (m, 9H; 9x H^{Ar}), 7.15-7.10 (m, 6H; 6x H^{Ar}), 6.55 (s, 1H; H^{Ar}), 3.91 (s, 2H; CH_2), 1.33 (s, 12H, 4x CH_3) ppm; **^{13}C NMR (**76 MHz, $CDCl_3$, APT): δ = 152.8 (C^{Ar}), 151.8 (C^{Ar}), 143.0 (C^{Ar}), 142.3 (C_q; 3x C^{Ar}), 139.2 (C_q; C^{Ar}), 138.8 (C^{Ar}), 135.0 (C_q; C^{Ar}), 129.8 (6x C^{Ar}), 128.2 (9x C^{Ar}), 128.4 (6x C^{Ar}), 119.3 (C^{Ar}), 84.3 (2x C_q), 75.7 (C_q), 32.0 (CH_2), 25.0 (4x CH_3) ppm;[9] **HRMS** (MALDI): calcd (*m*/*z*) for [M^+]: 527.2744; found: 527.2749.

3.7 Synthesis of the Lysine and Tyrosine building blocks

3.7.1 3,5-Diiodopyridine

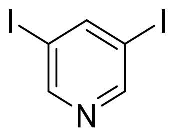

2

2 was prepared according to general procedure 2.2 from 7.27 g 3,5-dibromopyridine (**1**) (30.7 mmol, 1.0 eq), 585 mg copper(I) iodide (3.1 mmol, 10 mol%) and 18.4 g sodium iodide (130 mmol, 4.0 eq) and 330 µL *N*,*N*'-dimethylethylenediamine (270 mg, 3.07 mmol, 10 mol%) in 50 mL abs., degassed 1,4-dioxane. The crude product was recrystallized from 235 mL EtOH.
Yield: 8.72 g (86%), pale golden shavings, $C_5H_3I_2N$ [330.89 g/mol].

TLC: R_f = 0.68 (cyclohexane/EtOAc = 9/1); **m.p.**$^{exp.}$ = 166-168 °C (m.p.$^{lit.}$ = 170-172 °C);[10] **^{1}H NMR** (300 MHz, $CDCl_3$): δ = 8.75 (d, $^4J_{H,H}$ = 1.3 Hz, 2H; H^{Ar}), 8.35 (t, $^4J_{H,H}$ = 1.8 Hz, 1H;

[9] Signal for the quaternary *ipso*-pyridine carbon (C_q; C^{Ar}) at the boronic acid pinacol ester function was not observed.

H^{Ar}) ppm; **^{13}C NMR** (76 MHz, $CDCl_3$): δ = 154.3 (C^{Ar}), 151.7 (C^{Ar}), 94.0 (C_q; C^{Ar}) ppm; **GC-MS** (EI, 70 eV; MP_50_S): t_R = 5.66 min; *m/z* (%): 331 (100) [M^+], 204 (46) [M^+−I], 77 (17) [M^+−I_2].

Analytical data are in accordance with those reported.[10]

3.7.2 4-((*tert*-Butyldimethylsilyl)oxy)benzaldehyde

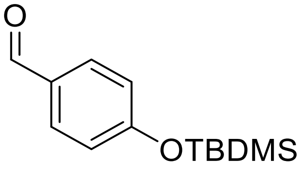

24

In a 100 mL round-bottom flask 2.00 g 4-hydroxybenzaldehyde (16.4 mmol, 1.0 eq) and 2.79 g imidazole (40.9 mmol, 2.5 eq) were dissolved in 80 mL DCM. 2.96 g *tert*-Butylchlorodimethylsilane (19.7 mmol, 1.2 eq) were added to the pale yellow solution and immediately a white precipitate was formed. After the reaction was stirred for 6 h at RT full conversion was detected by GC-MS. The suspension was diluted with 70 mL DCM and washed with H_2O (2 x 100 mL) and satd. NaCl solution (1 x 100 mL). The organic layer was dried over Na_2SO_4, filtered and the solvent was removed under reduced pressure. The orange oily crude product was purified via silica gel filtration (25 g SiO_2, 7.5 x 5.0 cm, eluent: cyclohexane/EtOAc = 20/1, R_f = 0.29, UV and CAM).

Yield: 3.47 g (90%), yellow oil, $C_{13}H_{20}O_2Si$ [236.39 g/mol].

TLC: R_f = 0.29 (cyclohexane/EtOAc = 20/1, UV and CAM); **^{1}H NMR** (300 MHz , $CDCl_3$): δ = 9.88 (s, 1H; CHO), 7.78 (d, $^3J_{H,H}$ = 8.6 Hz, 2H; H^{Ar}), 6.94 (d, $^3J_{H,H}$ = 8.5 Hz, 2H; H^{Ar}), 0.99 (s, 9H; CH_3), 0.24 (s, 6H; CH_3) ppm; **^{13}C NMR** (75.53 MHz, $CDCl_3$, APT): δ = 191.0 (CO), 161.6 (C_q; C^{Ar}), 132.0 (C^{Ar}), 130.6 (C_q; C^{Ar}), 120.6 (C^{Ar}), 25.7 (CH_3), 18.4 (C_q), 4.2 (CH_3) ppm; **GC-MS** (EI, 70 eV; MT_50_S): t_R = 5.94 min; *m/z* (%): 236 (11) [M^+], 179 (100) [M^+-C_4H_9], 151 (43) [M^+-C_6H_{15}].

Analytical data are in accordance with those reported.[11]

3.7.3 (4-((*tert*-Butyldimethylsilyl)oxy)phenyl)(5-iodopyridin-3-yl)methanol

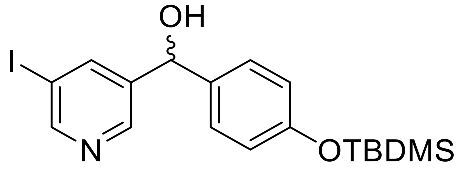

25a

A flame dried and nitrogen-flushed Schlenk-flask was charged with 200 mg 3,5-diiodopyridine (**2**) (604 µmol, 1.0 eq), which was dissolved in 3 mL abs. THF. The yellow solution was then cooled to -78 °C in an acetone/dry ice bath. Under stirring at -78 °C 300 µL *i*PrMgCl.LiCl solution (2.1M in THF) (635 µmol, 1.1 eq) were added via a syringe under N_2-flow. The reaction mixture was stirred at -78 °C until quantitative conversion of the metal-halide exchange was detected via GC-MS. After quantitative metal-halide exchange (4 h) 150 mg aldehyde **24** (635 µmol, 1.1 eq) were added at -78 °C under N_2-flow. The reaction mixture was then warmed to RT and was kept stirring until full conversion (overnight) was detected by GC-MS. The reaction mixture was quenched by the addition of 20 mL satd. NH_4Cl solution. Subsequently, the aqueous phase was extracted with DCM (3 x 20 mL). The combined organic layers were dried over Na_2SO_4, filtered and then the solvent was removed under reduced pressure to give a pale yellow oily crude product. The crude product was purified via flash column chromatography (15 g SiO_2, 2.0 x 16 cm, eluent: cyclohexane/EtOAc = 3/1, R_f = 0.30, UV and CAM).

Yield: 198 mg (74%), pale yellow oil, $C_{18}H_{24}INO_2Si$ [441.38 g/mol].

TLC: R_f = 0.13 (cyclohexane/EtOAc = 4/1, UV and CAM); **^{1}H NMR** (300 MHz, $CDCl_3$): δ = 8.64 (d, $^4J_{H,H}$ = 1.7 Hz, 1H; H^{Ar}), 8.45 (d, $^4J_{H,H}$ = 1.3 Hz, 1H; H^{Ar}), 8.06 (bs, 1H; H^{Ar}), 7.17 (d, $^3J_{H,H}$ = 8.5 Hz, 2H; H^{Ar}), 6.82 (d, $^3J_{H,H}$ = 8.5 Hz, 2H; H^{Ar}), 5.73 (s, 1H; CH), 2.96 (bs, 1H; OH), 0.97 (s, 9H; CH_3), 0.19 (s, 6H; CH_3) ppm; **^{13}C NMR** (76 MHz, $CDCl_3$): δ = 155.9 (C_q; C^{Ar}), 154.5 (C^{Ar}), 146.7 (C^{Ar}), 142.3 (C^{Ar}), 141.7 (C_q; C^{Ar}), 135.4 (C_q; C^{Ar}), 128.1 (C^{Ar}), 120.6 (C^{Ar}), 93.5 (C_q; C^{Ar}), 73.3 (CH), 25.8 (CH_3), 18.3 (C_q), -4.3 (CH_3) ppm; **GC-MS** (EI, 70 eV; MT_50_S): t_R = 9.01 min; *m/z* (%): 441 (27) [M^+], 384 (100) [M^+-C_4H_9], 234 (40) [M^+-$C_{12}H_{19}OSi$]; **HRMS** (EI): calcd (*m*/*z*) for [M^+]: 441.0621; found: 441.0633.

3.7.4 3-(4-((*tert*-Butyldimethylsilyl)oxy)benzyl)-5-iodopyridine

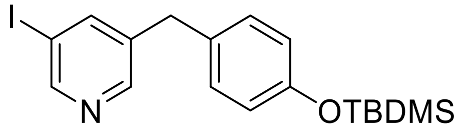

25

A 25 mL round-bottom-flask equipped with a Schlenk adapter was flushed with nitrogen and charged with 200 mg pyridine derivative **25a** (453 µmol, 1.0 eq) dissolved in 5 mL abs. DCM. After 615 µL triethylsilane (3.85 mmol, 8.5 eq) and 1.1 mL trifluoroacetic acid (14.5 mmol, 32 eq) were added, the yellow solution was stirred at RT overnight. The mixture was quenched by the addition of 150 mL satd. $NaHCO_3$ solution and stirred vigorously for 1 h. The phases were separated and the aqueous layer was extracted with DCM (5 x 50 mL). The combined organic layers were dried over Na_2SO_4, filtered and the solvent was removed under reduced pressure. The crude product was purified via flash column chromatography (15 g SiO_2, 2.0 x 16 cm, eluent: cyclohexane/EtOAc = 10/1, R_f = 0.34, UV and CAM).

Yield: 145 mg (75%), yellow oil, $C_{18}H_{24}INOSi$ [425.39 g/mol].

TLC: R_f = 0.34 (cyclohexane/EtOAc = 10/1, UV and CAM); **^{1}H NMR** (300 MHz, $CDCl_3$): δ = 8.66 (bs, 1H; H^{Ar}), 8.40 (bs, 1H; H^{Ar}), 7.78 (bs, 1H; H^{Ar}), 7.01 (d, $^3J_{H,H}$ = 8.4 Hz, 2H; H^{Ar}), 6.78 (d, $^3J_{H,H}$ = 8.4 Hz, 2H; H^{Ar}), 3.85 (s, 2H; CH_2), 0.98 (s, 9H; CH_3), 0.19 (s, 6H; CH_3) ppm; **^{13}C NMR** (76 MHz, $CDCl_3$): δ = 154.6 (C_q; C^{Ar}), 153.6 (C^{Ar}), 148.7 (C^{Ar}), 144.6 (C^{Ar}), 139.3 (C_q; C^{Ar}), 131.6 (C_q; C^{Ar}), 130.0 (C^{Ar}), 120.5 (C^{Ar}), 93.7 (C_q; C^{Ar}), 38.0 (CH_2), 25.8 (CH_3), 18.3 (C_q), -4.3 (CH_3) ppm; **GC-MS** (EI, 70 eV; MT_50_S): t_R = 8.58 min; *m/z* (%): 425 (24) [M^+], 368 (100) [M^+-C_4H_9]; **HRMS** (EI): calcd (*m*/*z*) for [M^+]: 425.0672; found: 425.0680.

3.7.5 3-(4-((*tert*-Butyldimethylsilyl)oxy)benzyl)-5-(4,4,5,5-tetramethyl-1,3,2-dioxaborolan-2-yl)pyridine

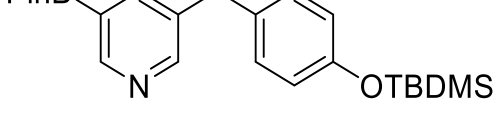

26

26 was prepared according to general procedure 2.3 from 150 mg pyridine derivative **25** (353 µmol, 1.0 eq) in 1 mL abs. THF, 230 µL *i*PrMgCl.LiCl solution (1.7M in THF) (391 µmol, 1.1 eq) and 100 µL PinBO*i*Pr (490 µmol, 1.4 eq).

Yield: 149 mg (99%), pale yellow solid, $C_{24}H_{36}BNO_3Si$ [425.45 g/mol].

m.p.$^{exp.}$ = 98-99 °C; **^{1}H NMR** (300 MHz, $CDCl_3$): δ = 8.78 (bs, 1H; H^{Ar}), 8.51 (d, ^4J = 2.0 Hz, 1H; H^{Ar}), 7.86 (bs, 1H; H^{Ar}), 7.01 (d, ^3J = 8.4 Hz, 2H; H^{Ar}), 6.76 (d, ^3J = 8.4 Hz, 2H; H^{Ar}), 3.89 (s, 2H; CH_2), 1.34 (s, 12H; CH_3), 0.97 (s, 9H; CH_3), 0.17 (s, 6H; CH_3) ppm; **^{13}C NMR** (76 MHz, $CDCl_3$): δ = 154.3 (C_q; C^{Ar}), 153.3 (C^{Ar}), 152.5 (C^{Ar}), 142.7 (C^{Ar}), 136.2 (C_q; C^{Ar}), 132.7 (C_q; C^{Ar}), 129.9 (C^{Ar}), 120.3 (C^{Ar}), 84.3 (C_q), 38.4 (CH_2), 25.8 (CH_3), 25.0 (CH_3), 18.3 (C_q), -4.3 (CH_3) ppm;[10] **GC-MS** (EI, 70 eV; MT_50_S): t_R = 9.59 min; *m/z* (%): 425 (28) [M^+], 368 (49) [M^+-C_4H_9], 268 (100) [M^+-$C_{10}H_{21}O$]; **HRMS** (EI): calcd (*m/z*) for [M^+]: 425.2562; found: 425.2570.

3.7.6 Oxobutanenitrile

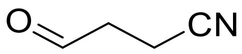

27

In a 250 mL two-neck round-bottom flask with argon-inlet 4.88 g 4,4-diethoxybutanenitrile (31.04 mmol, 1.0 eq) were mixed in degassed acetone (154 mL) and 6M HCl (62 mL). The colourless solution was stirred at -4 °C overnight. After the reaction was completed (detected by GC-MS), the acetone was removed in vacuo using a rotary evaporator at 25 °C. The aqueous residue (~60 mL) was extracted with DCM (4× 40 mL), and the combined organic layers were dried over Na_2SO_4 and filtered. The solvent was removed in vacuo using a rotary evaporator to give crude product as a colourless oil. After distillation (b.p.$^{1.7}$ = 56-58°C), compound **26** was obtained as a colourless liquid.

Yield: 1.92 g (74%), colourless liquid, C_4H_5NO [83.09 g/mol].

^{1}H NMR (300 MHz, $CDCl_3$): δ = 9.80 (s, 1H; CHO), 2.91 (t, $^3J_{H,H}$ = 7.1 Hz, 2H; CH_2), 2.64 (t, $^3J_{H,H}$ = 7.1 Hz, 2H; CH_2) ppm; **b.p.**$^{exp.}$ = 56-58 °C, 1.7 torr (b.p.$^{lit.}$ = 66-68 °C, 2 torr); **GC-MS** (EI, 70 eV; MT_50_S): t_R = 3.17 min; *m/z* (%): 82 (4) [M^+−H], 54 (100) [M^+−CHO].

The spectroscopic data are in accordance with those reported.[12]

[10] Signal for the quaternary *ipso*-pyridine carbon (C_q; C^{Ar}) at the boronic acid pinacol ester function was not observed.

3.7.7 4-Hydroxy-4-(5-iodopyridin-3-yl)butanenitrile

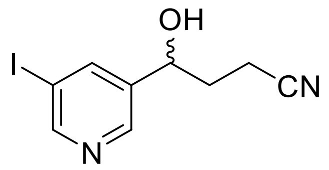

28a

In a flame-dried, Ar flushed Schlenk flask equipped with stirring bar 2.81 g 3,5-diiodopyridine (**2**) (8.49 mmol, 1.0 eq) were dissolved in 30 mL absolute, degassed THF. The colourless solution was cooled to -78 °C in an ice-water bath and 7.83 mL *i*PrMgCl·LiCl solution (8.93 mmol, 1.14M, 1.1 eq) were added. The reaction mixture was stirred at -78 °C for 2 h and full metal-halide exchange was detected via GC-MS. 810 µL 4-oxobutanenitrile (**27**) (843 mg, 10.1 mmol, 1.2 eq) were added and the reaction mixture was allowed to warm to RT overnight. 30 mL satd NH_4Cl were added and the aqueous phase was extracted with DCM (5x 20 mL). The combined organic phases were dried over Na_2SO_4, filtered and washed with DCM. The solvent was removed under reduced pressure and the crude product was purified via flash column chromatography (cyclohexane/EtOAc = 1/1, R_f = 0.20, CAM).

Yield: 2.04 g (83%), pale yellow, highly viscous oil, C_9H_9INO [288.09 g/mol].

^{1}H NMR (300 MHz, $CDCl_3$): δ = 8.71 (d, $^4J_{H,H}$ = 1.9 Hz, 1H; H^{Ar}), 8.49 (d, $^4J_{H,H}$ = 1.7 Hz, 1H; H^{Ar}), 8.09 (t, $^4J_{H,H}$ = 1.7 Hz, 1H; H^{Ar}), 4.84 (t, $^3J_{H,H}$ = 6.6 Hz, 1H; CH), 3.15 (bs, 1H; OH), 2.70-2.43 (m, 2H; CH_2), 2.05-1.98 (m, 2H; CH_2) ppm; **^{13}C NMR** (76 MHz, $CDCl_3$): δ = 155.0 (C^{Ar}), 145.7 (C^{Ar}), 142.4 (C^{Ar}), 141.2 (C_q; C^{Ar}), 119.3 (C_q; CN), 93.8 (C_q; C^{Ar}), 69.2 (CH), 34.3 (CH_2), 13.9 (CH_2) ppm; **GC-MS** (EI, 70 eV; MT_50_S): t_R = 7.31 min; *m/z* (%): 288 (20) [M^+], 234 (100) [$M^+-C_3H_4N$]; **HRMS** (EI): calcd (*m*/*z*) for [M^+]: 287.9760; found: 287.9776.

3.7.8 4-Chloro-4-(5-iodopyridin-3-yl)butanenitrile

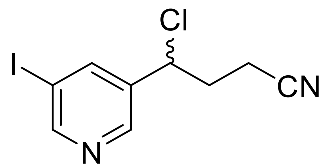

28

A 50 mL round-bottom flask with reflux condenser was charged with 1.79 g 4-hydroxy-4-(5-iodopyridin-3-yl)butanenitrile (**28a**) (6.21 mmol, 1.0 eq). After cooling to -12 °C, 20 mL $SOCl_2$ and 10 mL DCM were added and the reaction mixture was stirred at RT for 21 h. After quantitative conversion was detected via GC-MS, the $SOCl_2$ was distilled off, and the crude product was quenched with saturated Na_2CO_3 solution. The aqueous phase (pH ~8) was extracted with DCM (4x 20 mL), and the combined organic layers were washed with brine (1x 15 mL). The pale yellow combined organic phases were dried over Na_2SO_4, and after filtration, the solvent was removed under reduced pressure. The crude product was purified via flash column chromatography (37 g SiO_2, 8 x 3.5 cm, cyclohexane/EtOAc = 6/4, R_f = 0.38, UV and CAM).

Yield: 1.85 g (97%), reddish brown oil, $C_9H_8ClIN_2$ [306.53 g/mol].

^{1}H NMR (300 MHz, $CDCl_3$): δ = 8.82 (d, $^4J_{H,H}$ = 1.7 Hz, 1H; H^{Ar}), 8.59 (d, $^4J_{H,H}$ = 1.7 Hz, 1H; H^{Ar}), 8.10 (t, $^4J_{H,H}$ = 1.8 Hz, 1H; H^{Ar}), 4.95 (dd, $^3J_{H,H}$ = 9.1 Hz, $^4J_{H,H}$ = 5.3 Hz, 1H; CH), 2.73-2.57 (m, 2H; CH_2), 2.40-2.32 (m, 2H; CH_2) ppm; **^{13}C NMR** (76 MHz, $CDCl_3$): δ = 156.1 (C^{Ar}), 146.4 (C^{Ar}), 143.1 (C^{Ar}), 137.8 (C_q; C^{Ar}), 118.0 (C_q; CN), 93.6 (C_q; C^{Ar}), 57.5 (CH), 35.3 (CH_2), 15.4 (CH_2) ppm; **GC-MS** (EI, 70 eV; MT_50_S): t_R = 7.09 min; *m/z* (%): 306 (97) [M^+], 271 (100) [M^+−Cl], 252 (58) [M^+−C_3H_4N]; **HRMS** (EI): calcd (*m/z*) for [M^+]: 305.9421; found: 305.9422.

3.7.9 4-Chloro-4-(5-(4,4,5,5-tetramethyl-1,3,2-dioxaborolan-2-yl)pyridin-3-yl)butanenitrile

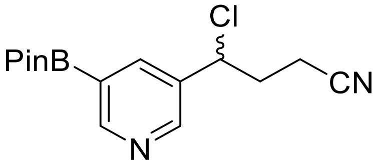

29a

Compound **29a** was prepared according to procedure 2.3 from 1.44 g 4-chloro-4-(5-iodopyridin-3-yl)butanenitrile (**28**) (4.70 mmol, 1.0 eq) in 20 mL absolute, degassed THF, 4.40 mL *i*PrMgCl·LiCl solution (5.10 mmol, 1.16 M, 1.1 eq), and 1.10 mL PinBO*i*Pr (1.00 g, 5.39 mmol, 1.1 eq). The crude product was used in the next step without further purification.

Yield: 1.37 g (95%, crude), pale yellow oil, $C_{15}H_{20}BClN_2O_2$ [306.60 g/mol].

TLC: R_f = 0.09 (cyclohexane/EtOAc = 3/7, UV and CAM); **GC-MS** (EI, 70 eV; MT_50_S): t_R = 7.74 min; *m/z* (%): 306 (9) [M^+], 291 (48) [M^+−CH3], 271 (100) [M^+−Cl], 221 (30) [*M*+−C_6H_{13}], 207 (68) [M^+−C_5H_9NO]; **HRMS** (EI): calcd (*m/z*) for [M^+−H]: 304.1264; found: 304.1273.

3.7.10 4-(5-(4,4,5,5-Tetramethyl-1,3,2-dioxaborolan-2-yl)pyridin-3-yl)butanenitrile

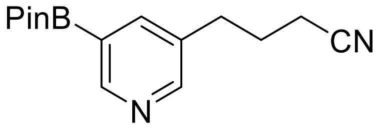

29

In a 250 mL round-bottom flask 1.36 g 4-chloro-4-(5-(4,4,5,5-tetramethyl-1,3,2-dioxaborolan-2-yl)pyridin-3-yl)butanenitrile (**29a**) (4.44 mmol, 1.0 eq) were dissolved in 90 mL DCM and 5.8 mL AcOH. After addition of 867 mg zinc dust (13.25 mmol, 3.0 eq), the green suspension was stirred at RT for 5 h. After quantitative conversion was detected via GC-MS the reaction mixture was quenched with saturated Na_2CO_3 solution, extracted with DCM (4× 50 mL) and washed with brine (1x 50 mL). The combined organic layers were dried over Na_2SO_4 and filtered. The solvent was removed under reduced pressure. After Kugelrohr-distillation (150 °C, $1\cdot10^{-3}$ mbar), compound **29** was isolated as a colourless solid.

Yield: 809 mg (63% over two steps), colourless solid, $C_{15}H_{21}BN_2O_2$ [272.15 g/mol].

TLC: R_f = 0.11 (EtOAc/MeOH = 4/1, UV and CAM); **^{1}H NMR** (300 MHz, $CDCl_3$): δ = 8.82 (d, $^4J_{H,H}$ = 1.3 Hz, 1H; H^{Ar}), 8.53 (d, $^4J_{H,H}$ = 2.3 Hz, 1H; H^{Ar}), 7.93 (bs, 1H; H^{Ar}), 2.81-2.76 (m, 2H; CH_2), 2.36 (t, $^3J_{H,H}$ = 7.1 Hz, 2H; CH_2), 2.05-1.96 (m, 2H; CH_2), 1.35 (s, 12H; CH_3) ppm; **^{13}C NMR** (76 MHz, $CDCl_3$): δ = 153.2 (C^{Ar}), 151.4 C^{Ar}), 142.7 (C^{Ar}), 134.8 (C_q; C^{Ar}), 119.1 (C_q; CN), 84.5 (C_q; C^{BPin}), 31.7 (CH_2), 26.8 (CH_2), 25.0 (CH_3), 16.7 (CH_2) ppm;[11] **GC-MS** (EI, 70 eV; MT_50_S): t_R = 7.44 min; *m/z* (%): 272 (28) [M^+], 257 (59) [M^+−CH_3], 187 (41) [M^+−C_6H_{13}], 173 (100) [M^+−C_5H_9NO]; **m.p.**$^{exp.}$ = 55-57 °C; **b.p.**^{KRD} = 150 °C, $1\cdot10^{-3}$ mbar; **HRMS** (EI): calcd (*m*/*z*) for [M^+−H]: 271.1620; found: 271.1633.

[11] Signal for the quaternary *ipso*-pyridine carbon (C_q; C^{Ar}) at the boronic acid pinacol ester function was not observed.

3.8 Synthesis of the Threonine and Methionine building blocks

3.8.1 5-Iodonicotinaldehyde

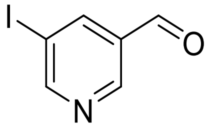

3

In a flame dried, nitrogen flushed 100 mL Schlenk-flask 1.0 g 3,5-diiodopyridine (**2**) (3.0 mmol, 1.0 eq) was dissolved in 10 mL abs. THF. After cooling to -78 °C in a dry ice/acetone bath 2.5 mL isopropylmagnesium chloride lithium chloride solution (1.27M in THF) (3.2 mmol, 1.1 eq) were added under N_2 counter flow and kept stirring at -78 °C until full conversion of the metal-halogen exchange was detected by GC-MS. After full conversion (3.5 h) 460 µL abs. DMF (5.9 mmol, 2.0 eq) were added at -78 °C under inert conditions. Subsequently the reaction mixture was brought to RT and stirred overnight (12 h). After quantitative conversion the reaction mixture was quenched by the addition of 10 mL satd. NH_4Cl solution and the aqueous layer was extracted with Et_2O (3 x 20 mL). The combined organic layers were dried over Na_2SO_4, filtered and the solvent was removed under reduced pressure to give a pale orange solid. The crude product was purified via flash column chromatography (100 g SiO_2, 4.5 x 15 cm, eluent: cyclohexane/EtOAc = 4/1, R_f = 0.21, UV and CAM).

Yield: 526 mg (78%), colorless powder, C_6H_4INO [233.01 g/mol].

TLC: R_f = 0.21 (cyclohexane/EtOAc = 4/1, UV and CAM); **m.p.**$^{exp.}$ = 143-145 °C; ^{1}**H NMR** (300 MHz, $CDCl_3$): δ = 10.03 (s, 1H; CHO), 9.05 (d, $^{4}J_{H,H}$ = 2.0 Hz, 1H; H^{Ar}), 9.00 (t, $^{4}J_{H,H}$ = 1.6 Hz, 1H; H^{Ar}), 8.49-8.48 (m, 1H; H^{Ar}) ppm; ^{13}**C NMR** (76 MHz, $CDCl_3$, APT): δ = 189.4 (CO), 160.8 (C^{Ar}), 150.31 (C^{Ar}), 144.0 (C^{Ar}), 132.9 (C_q; C^{Ar}), 94.0 (C_q; C^{Ar}) ppm; **GC-MS** (EI, 70 eV; MT_50_S): t_R = 5.28 min; *m/z* (%): 233 (100) [M^+], 204 (16) [M^+-CHO].

3.8.2 1-(5-Iodopyridin-3-yl)ethan-1-ol

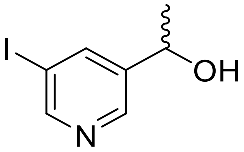

30

A flame dried and nitrogen flushed 250 mL two-neck-round-bottom flask equipped with a dropping funnel and N_2-inlet was charged with 1.54 g 5-iodonicotinaldehyde (**3**) (6.54 mmol, 1.0 eq) dissolved in 50 mL abs. THF. After cooling the reaction mixture to -78 °C 2.52 mL MeMgBr (2.86M in Et_2O) (7.20 mmol, 1.1 eq) were added dropwise to the reaction mixture over a period of 10 min. After stirring for 60 min the reaction was quenched by the addition of 5 mL EtOAc and allowed to warm up to 0 °C. HCl solution (6M) was added dropwise until the dark oily residue in the mixture was dissolved. 50 mL satd. $NaHCO_3$ solution were added and the aqueous phase was extracted with EtOAc (3 x 50 mL). The combined organic layers were washed with satd. NaCl solution, dried over Na_2SO_4, and concentrated in vacuum. The crude product was purified via flash column chromatography (20 g SiO_2, 2 x 15 cm, cyclohexane/EtOAc = 1/1, R_f = 0.32, UV).
Yield: 1.11 g (68%), yellow oil, C_7H_8INO [249.05 g/mol].

TLC: R_f = 0.32 (cyclohexane/EtOAc = 1/1, UV and CAM); **^{1}H NMR** (300 MHz, $CDCl_3$): δ = 8.68 (d, $^4J_{H,H}$ = 1.8 Hz, 1H; H^{Ar}), 8.47 (d, $^4J_{H,H}$ = 1.4 Hz, 1H; H^{Ar}), 8.09 (m, 1H; H^{Ar}), 4.89 (q, $^3J_{H,H}$ = 6.4 Hz, 1H; CH), 2.85 (bs, 1H; OH), 1.50 (d, $^3J_{H,H}$ = 6.5 Hz, 3H; CH_3) ppm; **^{13}C NMR** (76 MHz, $CDCl_3$, APT): δ = 154.5 (C^{Ar}), 145.7 (C^{Ar}), 143.5 (C_q, C^{Ar}), 142.0 (C^{Ar}), 93.7 (C_q, C^{Ar}), 67.5 (CH), 25.4 (CH_3). **GC-MS** (EI, 70 eV; MT_50_S): t_R = 5.917 min; *m/z* (%): 249 (58) [M^+], 234 (100) [M^+-CH_3]; **HRMS** (EI): calcd (*m/z*) for [M^+]: 248.9651; found: 248.9662.

3.8.3 3-(1-((*tert*-Butyldiphenylsilyl)oxy)ethyl)-5-iodopyridine

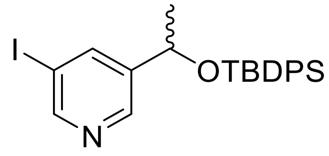

31a

In a one-neck-round-bottom-flask 960 mg **30** (3.87 mmol, 1.0 eq) and 660 mg imidazole (9.7 mmol, 2.5 eq) were dissolved in 18 mL DCM. After the addition of 1.05 mL TBDPSCl (4.06 mmol, 1.1 eq) a white precipitate started forming. The reaction mixture was stirred for 7 h and transferred into a separation funnel. The organic layer was washed with 3M NaOH (2 x 40 mL) and satd. NaCl solution (1 x 40 mL). The organic phase was dried over Na_2SO_4 and concentrated in vacuum. The crude product was purified via flash column chromatography (60 g, SiO_2, cyclohexane/EtOAc = 30/1, R_f = 0.21, UV).
Yield: 1.79 g (95%), colorless oil, $C_{23}H_{26}INOSi$ [487.46 g/mol].

TLC: R_f = 0.21 (cyclohexane/EtOAc = 30/1, UV); **^{1}H NMR** (300 MHz, [D6]DMSO): δ = 8.64 (d, $^4J_{H,H}$ = 1.7 Hz, 1H; H^{Ar}), 8.44 (bs, 1H; H^{Ar}), 7.99 (bs, 1H; H^{Ar}), 7.62-7.60 (m, 2H; H^{Ar}), 7.49-7.30 (m, 8H; H^{Ar}), 4.90 (q, $^3J_{H,H}$ = 6.3 Hz, 1H; CH), 1.34 (d, $^3J_{H,H}$ = 6.3 Hz, CH_3), 1.01 (s, 9H; CH_3) ppm; **^{13}C NMR** (76 MHz, [D6]DMSO): δ = 153.7 (C^{Ar}), 145.6 (C^{Ar}), 143.1 (C_q, C^{Ar}), 141.0 (C^{Ar}), 135.2 (C^{Ar}), 135.2 (C^{Ar}), 133.1 (C_q, C^{Ar}), 132.6 (C_q, C^{Ar}), 130.0 (C^{Ar}), 129.9 (C^{Ar}), 127.8 (C^{Ar}), 127.7 (C^{Ar}) 93.9 (C_q, C^{Ar}), 68.8 (CH), 26.7 (CH_3), 26.3 (CH_3), 18.7 (C_q) ppm; **GC-MS** (EI, 70 eV; MT_50_S): t_R = 9.94 min; *m/z* (%): 430 (62) [M^+-*t*Bu]; **HRMS** (EI): calcd (*m*/*z*) for [M^+]: 487.0829; found: 487.0853.

3.8.4 3-(1-((*tert*-Butyldiphenylsilyl)oxy)ethyl)-5-(4,4,5,5-tetramethyl-1,3,2-dioxaborolan-2-yl)pyridine

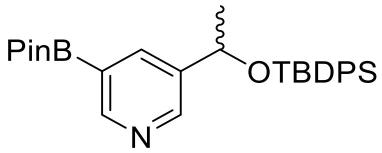

31

31 was prepared according to general procedure 2.3 from 1.39 g **31a** (2.85 mmol, 1.0 eq) in 15 mL abs. THF, 2.0 mL iPrMgCl·LiCl solution (1.7M in THF) (3.4 mmol, 1.2 eq) and 0.87 mL PinBOiPr (4.27 mmol, 1.5 eq). The crude product was purified via sublimation (165 °C, $8\cdot10^{-3}$ mbar).
Yield: 1.29 g (93%), colorless wax like solid, $C_{29}H_{38}BNO_3Si$ [487.52 g/mol].

^{1}H NMR (300 MHz, $CDCl_3$): δ = 8.71 (bs, 1H; H^{Ar}), 8.52 (d, $^4J_{H,H}$ = 2.2 Hz, 1H; H^{Ar}), 7.78 (bs, 1H; H^{Ar}), 7.62-7.60 (m, 2H; C^{Ar}), 7.42-7.15 (m, 8H; H^{Ar}), 4.77 (m, 1H; CH), 1.28 (s, 12H; CH_3), 1.19 (d, $^3J_{H,H}$ = 8.2 Hz, 3H; CH_3), 0.99 (s, 9H; CH_3) ppm; **^{13}C NMR** (76 MHz, $CDCl_3$, APT): δ = 153.9 (C^{Ar}), 149.7 (C^{Ar}), 140.5 (C^{Ar}), 140.5 (C^{Ar}), 139.5 (C_q, C^{Ar}), 135.8 (C^{Ar}), 135.8 (C^{Ar}), 134.0 (C_q, C^{Ar}), 129.7 (C^{Ar}), 129.6 (C^{Ar}), 127.6 (C^{Ar}), 127.5 (C^{Ar}), 84.2 (C_q), 70.1 (CH), 27.0 (CH_3), 26.7 (CH_3), 24.9 (CH_3), 24.8 (CH_3), 24.6 (CH_3), 19.2 (C_q) ppm;[12] **GC-MS** (EI, 70 eV; MT_50_S): t_R = 11.06 min; *m/z* (%): 430 (100) [M^+-*t*Bu]; **HRMS** (EI): calcd (*m*/*z*) for [M^+-*H*]: 486.2641; found: 486.2692.

[12] Signal for the quaternary *ipso*-pyridine carbon (C_q; C^{Ar}) at the boronic acid pinacol ester function was not observed.

3.8.5 ((Methylthio)methyl)triphenylphosphonium chloride

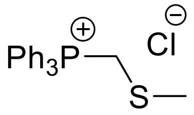

32

In a flame dried and argon flushed Schlenk-flask 20.0 g PPh_3 (76.3 mmol, 1.0 eq) were dissolved in 40 mL abs. toluene. 6.40 mL Chloromethyl methyl sulfide (7.36 g, 76.3 mmol, 1.0 eq) were added to the colorless solution. The solution turned yellow. The reaction mixture was stirred at 100 °C and within 24 h a colorless precipitate was formed. The suspension was cooled to 0 °C and the precipitate was collected by filtration and washed with toluene (3 x 5 mL).
Yield: 18.7 g (68%), colorless powder, $C_{20}H_{20}ClPS$ [358.86 g/mol].

m.p.$^{exp.}$ = 225-227 °C (m.p.$^{lit.}$ = 213-214 °C);[13] **^{1}H NMR** (300 MHz, [D6]DMSO): δ = 7.92-7.75 (m, 15H; H^{Ar}), 5.11 (d, $^2J_{H,P}$ = 9.0 Hz, 2H; CH_2), 1.90 (s, 3H; CH_3) ppm; **^{13}C NMR** (76 MHz, [D6]DMSO, APT): δ = 135.1 (d, $^4J_{C,P}$ = 3 Hz; C^{Ar}), 134.0 (d, $^3J_{C,P}$ = 10 Hz; C^{Ar}), 130.1 (d, $^2J_{C,P}$ = 12 Hz; C^{Ar}), 118.2 (d, $^1J_{C,P}$ = 87 Hz; C_q; C^{Ar}), 23.8 (d, $^1J_{C,P}$ = 51 Hz; CH_2), 17.2 (d, $^3J_{C,P}$ = 3 Hz; CH_3) ppm.

Analytical data are in accordance with those reported.[13]

3.8.6 *(E/Z)*-3-Iodo-5-(2-(methylthio)vinyl)pyridine

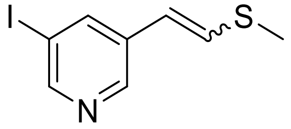

33

A flame dried and nitrogen flushed 250 mL two-neck round-bottom flask equipped with an N_2-inlet was charged with 4.85 g **32** (13.5 mmol, 1.1 eq) suspended in 96 mL abs. methanol and cooled to 0 °C. After adding 1.52 g KO*t*Bu (13.5 mmol, 1.1 eq) and stirring for 50 min 3.00 g 5-iodonicotinaldehyde (**3**) (12.9 mmol, 1.0 eq) were added. The reaction mixture was allowed to warm up to RT and stirred overnight. After full conversion was detected by GC-MS, the solvent removed under vacuum. The crude product was purified via flash column chromatography (155 g SiO_2, 7 x 12 cm, cyclohexane/EtOAc = 8/1, R_f = 0.20, UV).
Yield: 3.28 g (E/Z = 5.7/1, 79%), colorless solid, C_8H_8INS [277.12 g/mol].

(E): **m.p.**$^{exp.}$ = 45-55°C; **^{1}H NMR** (300 MHz, $CDCl_3$): δ = 8.60 (d, $^{4}J_{H,H}$ = 1.7 Hz, 1H; H^{Ar}), 8.44 (d, $^{4}J_{H,H}$ = 1.7 Hz, 1H; H^{Ar}), 7.93 (bs, 1H; H^{Ar}), 6.92 (d, $^{3}J_{H,H}$ = 15.5 Hz, 1H; CH), 6.11 (d, $^{3}J_{H,H}$ = 15.5 Hz, 1H; CH), 2.40 (s, 3H; CH_3) ppm; **^{13}C NMR** (76 MHz, $CDCl_3$): 153.2 (C^{Ar}), 145.6 (C^{Ar}), 139.8 (C^{Ar}), 134.8 (C_q, C^{Ar}), 130.6 (CH), 118.7 (CH), 93.7 (C_q, C^{Ar}), 18.9 (CH_3) ppm; **GC-MS** (EI, 70 eV; MT_50_S): t_R = 6.84 min; *m/z* (%): 277 (100) [M^+]; **HRMS** (EI): calcd (*m/z*) for [M^+]: 276.9422; found: 276.9413.

(Z): **m.p.**$^{exp.}$ = 45-55°C; **^{1}H NMR** (300 MHz, $CDCl_3$): δ = 8.63 (d, $^{4}J_{H,H}$ = 1.7 Hz, 1H; H^{Ar}), 8.58 (d, $^{4}J_{H,H}$ = 1.8 Hz, 1H; H^{Ar}), 8.18 (bs, 1H; H^{Ar}), 6.45 (d, $^{3}J_{H,H}$ = 11.0 Hz, 1H; CH), 6.27 (d, $^{3}J_{H,H}$ = 11.0 Hz, 1H; CH), 2.45 (s, 3H; CH_3) ppm; **^{13}C NMR** (76 MHz, $CDCl_3$): 153.0 (C^{Ar}), 184.4 (C^{Ar}), 142.9 (C^{Ar}), 134.8 (C_q, C^{Ar}), 133.8 (CH), 120.0 (CH), 93.7 (C_q, C^{Ar}), 18.9 (CH_3) ppm; **GC-MS** (EI, 70 eV; MT_50_S): t_R = 6.759 min; *m/z* (%): 277 (100) [M^+]; **HRMS** (EI): calcd (*m/z*) for [M^+]: 276.9422; found: 276.9413.

3.8.7 3-Iodo-5-(2-(methylthio)ethyl)pyridine

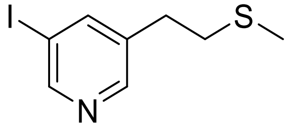

34a

A 100 mL round-bottom flask equipped with a reflux condenser was charged with 2.76 g **33** (10.0 mmol, 1.0 eq), 5.56 g *p*-tosylhydrazide (29.9 mmol, 3.0 eq) and 4.07 g NaOAc·3H_2O (29.9 mmol, 3.0 eq) suspended in 56 mL THF. The reaction mixture was stirred at 70 °C for 8 d. During this time additional p-tosylhydrazide and NaOAc·3H_2O were added in 4 equal portions to a total of 12 eq. After complete conversion was detected by GC-MS, 170 mL half-satd. $NaHCO_3$ solution and 250 mL DCM were added to the reaction mixture. The phases were separated and the aqueous layer was extracted with DCM (2 x 210 mL). The combined organic layers were washed with brine (1 x 400 mL), dried over Na_2SO_4 and the solvent was removed in vacuum. The crude product was purified via flash column chromatography (80 g SiO_2, 3.5 x 30 cm) cyclohexane/EtOAc = 5/1, R_f = 0.31, UV).

Yield: 5.16 g (89%), yellow oil, $C_8H_{10}INS$ [279.14 g/mol].

^{1}H NMR (300 MHz, $CDCl_3$): δ = 8.69 (d, $^{4}J_{H,H}$ = 1.8 Hz, 1H; H^{Ar}), 8.41 (d, $^{4}J_{H,H}$ = 1.6 Hz, 1H; H^{Ar}), 7.88 (bs, 1H; H^{Ar}), 2.87-2.82 (m, 2H; CH_2), 2.76-2.70 (m, 2H; CH_2), 2.12 (s, 3H; CH_3) ppm;

^{13}C NMR (76 MHz, $CDCl_3$): 154.2 (C^{Ar}), 148.8 (C^{Ar}), 144.6 (C^{Ar}), 138.2 (C_q, C^{Ar}), 93.8 (C_q, C^{Ar}), 35.5 (CH_2), 32.8 (CH_2), 16.1 (CH_3) ppm; **GC-MS** (EI, 70 eV; MT_50_S): t_R = 6.56 min; *m/z* (%): 279 (54) [M^+]; **HRMS** (EI): calcd (*m*/*z*) for [M^+]: 278.9579; found: 278.9576.

3.8.8 3-(2-(Methylthio)ethyl)-5-(4,4,5,5-tetramethyl-1,3,2-dioxaborolan-2-yl)pyridine

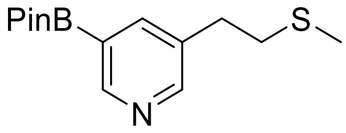

34

34 was prepared according to general procedure 2.3 from 2.33 g **34a** (8.36 mmol, 1.0 eq) dissolved in 30 mL abs. THF, 5.48 mL iPrMgCl·LiCl solution (1.83M in THF) (10.03 mmol, 1.2 eq) and 2.6 mL PinBO*i*Pr (12.5 mmol, 1.5 eq). The crude product was purified via Kugelrohr-distillation (165 °C, $8\cdot10^{-3}$ mbar).

Yield: 2.04 g (88%), colorless oil, $C_{14}H_{22}BNO_2$ [279.20 g/mol].

^{1}H NMR (300 MHz, $CDCl_3$): δ = 8.80 (d, $^4J_{H,H}$ = 1.3 Hz, 1H; H^{Ar}), 8.53 (d, $^4J_{H,H}$ = 2.2 Hz, 1H; H^{Ar}), 7.89 (bs, 1H; H^{Ar}), 2.91-2.86 (m, 2H; CH_2), 2.77-2.72 (m, 2H; CH_2),), 2.12 (s, 3H; CH_3), 1.35 (s, 12H; CH_3) ppm; **^{13}C NMR** (76 MHz, $CDCl_3$, APT): 153.7 (C^{Ar}), 152.4 (C^{Ar}), 142.2 (C^{Ar}), 135.1 (C_q, C^{Ar}), 84.4 (C_q), 35.5 (CH_2), 33.1 (CH_2), 25.0 (CH_3), 15.8 (CH_3) ppm;[13] **GC-MS** (EI, 70 eV; MT_50_S): t_R = 7.25 min; *m/z* (%): 279 (75) [M^+]; **HRMS** (EI): calcd (*m*/*z*) for [M^+]: 279.1467; found: 279.1474.

3.9 Synthesis of the Alanine and Leucine building blocks

3.9.1 3-Methyl-5-(4,4,5,5-tetramethyl-1,3,2-dioxaborolan-2-yl)pyridine

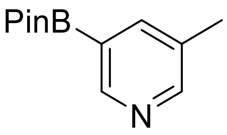

36

[13] Signal for the quaternary *ipso*-pyridine carbon (C_q; C^{Ar}) at the boronic acid pinacol ester function was not observed.

A flame dried and argon flushed Schlenk-flask was charged with 1.4 g B_2Pin_2 (5.5 mmol, 1.1 eq), 630 mg KOAc (6.5 mmol, 1.3 eq) 25 mg Pd_2dba_3 (27 µmol, 0.6 mol%) and 40 mg XPhos (84 µmol, 1.7 mol%). A solution of 575 mg 3-bromo-5-methyl pyridine (**35**) (5.0 mmol, 1.0 eq) in 7.5 mL absolute, degassed 1,4-dioxane was added and the purple suspension was stirred at 110 °C for 20 h. When full conversion was detected by GC-MS (20 h) the catalyst was removed by filtration through a pad of silica gel (eluted with 3 x 90 mL EtOAc) and the solvent was removed under reduced pressure. The product was purified via recrystallization from pentane and washed with cold ethyl acetate.

Yield: 657 mg (60%), colorless powder, $C_{12}H_{18}BNO_2$ [219.09 g/mol].

m.p.$^{exp.}$ = 121-123 °C; ^{1}**H NMR** (300 MHz, $CDCl_3$): δ = 8.73 (s, 1H; H^{Ar}), 8.48 (s, 1H; H^{Ar}), 7.88 (s, 1H; H^{Ar}), 2.31 (s, 3H; CH_3), 1.32 (s, 12H; CH_3) ppm; ^{13}**C NMR** (76 MHz, $CDCl_3$, APT): δ = 152.1 (C^{Ar}), 151.8 (C^{Ar}), 143.2 (C^{Ar}), 132.6 (C_q; C^{Ar}), 84.2 (C_q; C^{Ar}), 25.0 (CH_3), 18.4 (CH_3) ppm;[14] **GC-MS** (EI, 70 eV; MT_50_S): t_R = 5.71 min; *m/z* (%): 219 (34) [M^+], 204 (59) [M^+-CH_3], 162 (26) [$M^+-C_4H_{12}$], 120 (100) [$M^+-C_6H_{12}O$].

3.9.2 3-Chloro-5-isobutylpyridine

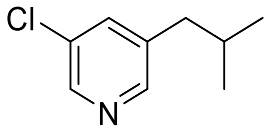

38

A three-neck round-bottom flask equipped with a reflux condenser, inline oil bubbler (after flame drying), dropping funnel and vacuum adapter with stopcock was charged with 2.00 g Mg turnings (82.3 mmol, 1.0 eq). Then the setup was flame dried and flushed with argon. Under inert conditions 10 mL abs. THF were added and the Mg was activated by adding I_2. A solution of 8.90 mL isobutyl bromide (11.3 g, 82.3 mmol, 1.0 eq) in 20 mL abs. THF were added dropwise via the dropping funnel. After stirring the grey, turbid reaction mixture for 2 h at 70 °C the isobutyl magnesium chloride solution was transferred to a flame dried and argon flushed Schlenk-flask. For determination of the actual concentration of the Grignard solution, a titration was performed as described in 1.

[14] Signal for the quaternary *ipso*-pyridine carbon (C_q; C^{Ar}) at the boronic acid pinacol ester function was not observed.

A flame dried and argon flushed three-neck round-bottom flask equipped with dropping funnel and vacuum adapter with stopcock was charged with 3.0 g 3,5-dichloropyridine (**37**) (20.3 mmol, 1.0 eq) and 358 mg $Fe(acac)_2$ (1.01 mmol, 5 mol%). 120 mL abs. THF and 6.5 mL abs. NMP were added and a red-orange solution was formed. The solution was cooled to 0 °C in an ice bath and 6.9 mL previously prepared isobutylmagnesium bromide solution (2.95M in THF) (3.27 g, 20.3 mmol, 1.0 eq) were added dropwise via the dropping funnel. The reaction mixture turned dark brownish purple and was stirred overnight at RT. When quantitative conversion was detected via GC-MS (16 h) the catalyst was removed by filtration through a pad of silica gel (eluted with 150 mL EtOAc) and the solvent of the collected filtrate was removed under reduced pressure to yield a brown, oily crude product, which was purified via flash column chromatography (250 g SiO_2, 6.0 x 16 cm, eluent: cyclohexane/EtOAc = 5/1, R_f = 0.52, UV).
Yield: 2.88 g (84%), pale yellow oil, $C_9H_{12}ClN$ [169.65 g/mol].

TLC: R_f = 0.52 (cyclohexane/EtOAc = 5/1, UV and CAM); **^{1}H NMR** (300 MHz, $CDCl_3$): δ = 8.38 (d, $^4J_{H,H}$ = 2.1 Hz, 1H; H^{Ar}), 8.25 (d, $^4J_{H,H}$ = 1.3 Hz, 1H; H^{Ar}), 7.43 (bs, 1H; CH), 2.44 (d, $^3J_{H,H}$ = 7.2 Hz, 2H; CH_2), 1.84 (h, $^3J_{H,H}$ = 6.8 Hz, 1H; CH), 0.89 (d, $^3J_{H,H}$ = 6.6 Hz, 6H; CH_3) ppm; **^{13}C NMR** (76 MHz, $CDCl_3$, APT): δ = 148.4 (C^{Ar}), 146.3 (C^{Ar}), 138.2 (C_q; C^{Ar}), 136.2 (C^{Ar}), 131.7 (C_q; C^{Ar}), 41.9 (CH_2), 30.0 (CH), 22.2 (CH_3) ppm; **GC-MS** (EI, 70 eV; MT_50_XS): t_R = 9.70 min; *m/z* (%): 169 (40) [M^+], 127 (100) [$M^+-C_3H_7$], 92 (16) [$M^+-C_3H_7Cl$].

Analytical data are in accordance with those reported.[14]

3.9.3 3-Isobutyl-5-(4,4,5,5-tetramethyl-1,3,2-dioxaborolan-2-yl)pyridine

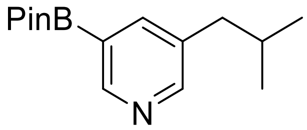

39

A flame dried round bottom flask with Schlenk adapter was charged with 3.01 g 3-chloro-5-isobutylpyridine (**38**) (17.7 mmol, 1.0 eq), 4.96 g bis(pinacolato)diboron (19.5 mmol, 1.1 eq), 98.0 mg Pd_2dba_3 (0.11 mmol, 0.6 mol%) and 143 mg XPhos (0.30 mmol, 1.7 mol%). The round bottom flask was evacuated and flushed with argon (3x). Then, 30 mL abs., degassed 1,4-dioxane were added. The obtained brown-red suspension was stirred at 105 °C for 24 h. After full conversion was detected by GC-MS, the solvent was removed under reduced pressure. The crude

product was dissolved in 100 mL EtOAc, washed with H_2O (100 mL) and the aqueous layer was extracted with EtOAc (2 x 100 mL). The combined organic layers were dried over Na_2SO_4, the solvent was removed under reduced pressure and the crude product was further purified via Kugelrohr-distillation (125 °C, 0.1 mbar).
Yield: 4.31 g (93%) colourless solid, $C_{15}H_{24}BNO_2$ [261.17 g/mol].

m.p.$^{exp.}$ = 71-73 °C, (m.p.$^{lit.}$ = 75-77 °C);[5] **^{1}H NMR** (300 MHz, $CDCl_3$): δ = 8.77 (d, $^4J_{H,H}$ = 1.3 Hz, 1H; H^{Ar}), 8.46 (d, $^4J_{H,H}$ = 2.2 Hz, 1H; H^{Ar}), 7.88 (bs, 1H; H^{Ar}), 2.48 (d, $^3J_{H,H}$ = 7.2 Hz, 2H; CH_2), 1.95-1.82 (m, 1H; CH), 1.35 (s, 12H; CH_3), 0.91 (d, $^3J_{H,H}$ = 6.6 Hz, 6H; CH_3) ppm; **^{13}C NMR** (76 MHz, $CDCl_3$): δ = 152.3 (C^{Ar}), 151.8 (C^{Ar}), 143.4 (C^{Ar}), 136.5 (C_q; C^{Ar}), 84.4 (C_q; C^{Ar}), 42.4 (CH_2), 30.2 (CH), 25.0 (CH_3), 22.4 (CH_3) ppm;[15] **GC-MS** (EI, 70 eV; MT_50_S): t_R = 6.53 min; *m/z* (%): 261 (64) [M^+], 246 (100) [M^+-CH_3], 218 (46) [$M^+-C_3H_7$], 162 (69) [$M^+-C_6H_{12}O$]; **HRMS** (EI): calcd (*m/z*) for [M^+]: 261.1903; found: 261.1884.

Analytical data are in accordance with those reported.[5]

3.10 Synthesis of the Cysteine and Serine building blocks

3.10.1 (5-Bromopyridin-3-yl)methanol

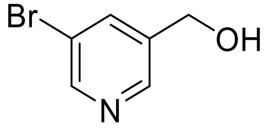

41a

In a flame dried and nitrogen flushed 500 mL two-neck round-bottom flask, equipped with an addition funnel and N_2-inlet 3.53 g $LiAlH_4$ (46.54 mmol, 2.0 eq) were suspended in 84 mL abs. THF and cooled to -78 °C in a dry ice/acetone bath. Through the addition funnel a solution of 10.00 g methyl 5-bromonicotinate (**40**) (46.54 mmol, 1.0 eq) dissolved in 84 mL abs. THF was added dropwise to the reaction mixture over a period of 20 min. After stirring the suspension at -78 °C for 60 min the reaction was quenched by adding 12.5 mL EtOAc. After the consecutive addition of 3.53 mL H_2O, 3.53 mL 15% NaOH and 10.59 mL H_2O the mixture was allowed to warm up to RT and was stirred vigorously for 60 min. The reaction was dried over anhydrous $MgSO_4$ and

[15] Signal for the quaternary *ipso*-pyridine carbon (C_q; C^{Ar}) at the boronic acid pinacol ester function was not observed.

filtrated through a glass frit. The solvent was removed under reduced pressure to give a brown oil. The crude product was used without further purification. An analytical sample was purified via flash column chromatography (cyclohexane/EtOAc = 4/1, R_f = 0.17, CAM).

Yield: 8.26 g (94%), brown oil, C_6H_6BrNO [188.02 g/mol].

TLC: R_f = 0.17 (cyclohexane/EtOAc = 4/1, UV and CAM); **^{1}H NMR** (300 MHz, $CDCl_3$): δ = 8.57 (d, $^4J_{H,H}$ = 1.8 Hz, 1H; H^{Ar}), 8.46 (bs, 1H; H^{Ar}), 7.89 (bs, 1H; H^{Ar}), 4.72 (s, 2H; CH_2), 2.70 (bs, 1H; OH) ppm; **^{13}C NMR** (76 MHz, $CDCl_3$, APT): δ = 150.0 (C^{Ar}), 145.7 (C^{Ar}), 138.2 (C_q, C^{Ar}), 137.5 (C^{Ar}), 121.1 (C_q, C^{Ar}), 61.9 (CH_2) ppm; **GC-MS** (EI, 70 eV; MT_50_S): t_R = 5.44 min; *m/z* (%): 189 (80) [M^+], 187 (82) [M^+].

Analytical data are in accordance with those reported.[15]

3.10.2 **(5-Iodopyridin-3-yl)methanol**

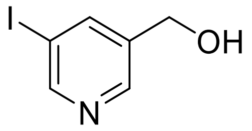

41

41 was prepared according to general procedure 2.2 from 4.02 g **41a** (21.39 mmol, 1.0 eq), 6.41 g NaI (42.78 mmol, 2.0 eq), 407 mg CuI (2.14 mmol, 10 mol%) and 230 µL *N,N'*-dimethylethylenediamine (2.14 mmol, 10 mol%) in 36 mL abs. 1,4-dioxane. The crude product was purified via flash column chromatography (130 g SiO2, 6.5 x 9 cm, cyclohexane/EtOAc = 2/1, R_f = 0.24, CAM).

Yield: 3.14 g (79%), colorless solid, C_6H_6INO [235.02 g/mol].

TLC: R_f = 0.24 (cyclohexane/EtOAc = 2/1, UV and CAM); **m.p.**$^{exp.}$ = 63-65 °C; **^{1}H NMR** (300 MHz, $CDCl_3$): δ = 8.69 (d, $^4J_{H,H}$ = 1.4 Hz, 1H; H^{Ar}), 8.46 (s, 1H; H^{Ar}), 8.07 (s, 1H; H^{Ar}), 4.67 (s, 2H; CH_2), 2.78 (bs, 1H; OH) ppm; **^{13}C NMR** (76 MHz, $CDCl_3$, APT): δ = 154.8 (C^{Ar}), 146.7 (C^{Ar}), 143.2 (C^{Ar}), 138.6 (C_q; C^{Ar}), 93.6 (C_q; C^{Ar}), 61.9 (CH_2) ppm; **GC-MS** (EI, 70 eV; MT_50_S): t_R = 5.60 min; *m/z* (%): 235 (100) [M^+], 206 (36) [M^+−CH_2OH], 108 (14) [M^+−I].

3.10.3 3-(Chloromethyl)-5-iodopyridine

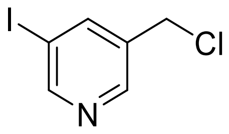

42

In a 50 mL round-bottom-flask 887 mg (5-iodopyridin-3-yl)methanol (**41**) (3.7 mmol, 1.0 eq) were dissolved in 15 mL thionylchloride and the reaction was stirred for 60 min at RT. Evolving acidic gases were neutralized by bubbling through a satd. $NaHCO_3$ solution. After quantitative conversion was detected by TLC excess thionylchloride was evaporated in vacuum and collected by a cooling trap cooled in liquid N_2. The remaining oily residue was dissolved in 50 mL DCM and washed with satd. $NaHCO_3$ solution (1 x 50 mL). The phases were separated and the aqueous phase extracted with DCM (2 x 50 mL). The combined organic phases were washed with brine (1 x 100 mL), dried over Na_2SO_4 and concentrated in vacuum to yield a brown oil. The crude product was purified via flash column chromatography (100 g SiO_2, 3.0 x 27 cm, eluent: cyclohexane/EtOAc = 14/1, R_f = 0.17, UV).

Yield: 839 mg (88%), colorless powder, C_6H_5ClIN [253.47 g/mol].

^{1}H NMR (300 MHz, $CDCl_3$): δ = 8.74 (d, $^4J_{H,H}$ = 1.6 Hz, 1H; H^{Ar}), 8.51 (d, $^4J_{H,H}$ = 1.3 Hz, 1H; H^{Ar}), 8.04 (bs, 1H; H^{Ar}), 4.48 (s, 2H; CH_2) ppm; **^{13}C NMR** (76 MHz, $CDCl_3$, APT): δ = 155.8 (C^{Ar}), 148.1 (C^{Ar}), 144.4 (C^{Ar}), 135.2 (C_q; C^{Ar}), 93.4 (C_q; C^{Ar}), 42.3 (CH_2) ppm; **GC-MS** (EI, 70 eV; MT_50_S): t_R = 5.46 min; *m/z* (%): 253 (100) [M^+], 218 (86) [M^{++}−Cl], 126 (29) [M^+−I], 91 (30) [M^+−ClI]; **m.p.**$^{exp.}$ = 63-64 °C; **HRMS** (EI): calcd (*m*/*z*) for [M^+]: 252.9155; found: 252.9160.

3.10.4 2-Methoxy-2-methylpropanoic acid

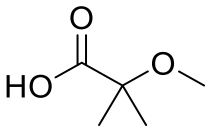

43a

A flame dried and nitrogen flushed 500 mL three-neck round-bottom flask equipped with an addition funnel, a reflux condenser and a mechanical stirrer was charged with 24.08 g KOH (428.6 mmol, 4.0 eq) dissolved in a mixture of 54 mL methanol and 12.4 mL H_2O. After cooling

the reaction mixture to 0 °C a solution of 20.00 g 1,1,1-trichloro-2-methyl-2-propanol hemihydrate (107.3 mmol, 1.0 eq) in 38 mL methanol was added dropwise within 30 min. Then the ice bath was removed and the suspension was stirred for 1 h at RT. Afterwards, the reaction mixture was heated under reflux for 2 h and filtered after cooling to RT. The filtrate was concentrated in vacuum, the resulting residue was cooled to 0 °C and 42 mL H_2SO_4 (1M) were added. The colorless precipitate was removed via filtration and washed with DCM (5 x 30 mL). The phases were separated and the aqueous layer extracted with DCM (4 x 50 mL). The combined organic layers were dried over Na_2SO_4 and concentrated in vacuum. The crude product was purified via fractionated distillation (114-116 °C, 57 mbar).
Yield: 7.76 g (61%), colorless liquid, $C_5H_{10}O_3$ [118.13 g/mol].

^{1}H NMR (300 MHz, $CDCl_3$): δ = 3.33 (s, 3H; OCH_3), 1.46 (s, 6H; CH_3) ppm; **^{13}C NMR** (76 MHz, $CDCl_3$): δ = 179.4 (C_q, CO), 77.7 (C_q), 52.0 (OCH_3), 23.7 (CH_3) ppm; **b.p.**$^{exp.}$ = 114-116 °C, 57 mbar, (**b.p.**$^{lit.}$ = 98-99 °C, 20 torr).[16]

Analytical data are in accordance with those reported.[16]

3.10.5 2-Methoxy-2-methylpropanethioic *S*-acid

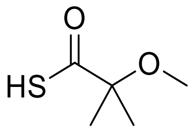

43

A 25 mL round-bottom flask equipped with a reflux condenser was charged with 3.00 g 2-methoxy-2-methylpropanoic acid (**43a**) (25.4 mmol, 1.0 eq). Upon the addition of 5.2 mL oxalyl chloride (60 mmol, 2.4 eq) the reaction mixture was heated to reflux for 5 min, which caused rapid development of gas. This gas was directed into two gas-washing bottles consecutively. The first gas-washing bottle was empty and served as a safety bottle. The second gas-washing bottle was filled with 200 mL satd. $NaHCO_3$ solution. After the yellow suspension had been stirred for 1 h at RT the excess oxalyl chloride was removed in vacuum by using a liquid N_2 cooled cold trap.
Meanwhile, in a second round-bottom flask 4.74 g sodium hydrosulfide hydrate (50.82 mmol, 2.0 eq) were mixed with 16 mL methanol and cooled to 0 °C. The previously prepared acid chloride was added to this suspension via a syringe dropwise over a period of 1.5 h. After the addition was completed the orange suspension was stirred for 1 h at 0 °C and 1 h at RT consecutively. Then the reaction mixture was poured into 100 mL H_2O and extracted with DCM (4 x 50 mL). The organic

layer was discarded while the aqueous layer was transferred into a 500 mL three-neck round-bottom flask with nitrogen inlet and acidified by the addition of 25 mL 20% HCl causing a rapid generation of gas, which was directed into two gas-washing bottles consecutively. The first gas-washing bottle was empty and served as a safety bottle. The second gas-washing bottle was filled with 100 mL 15% NaOCl solution. The reaction mixture was stirred overnight and flushed with a gentle stream of nitrogen. Afterwards, the yellow solution was transferred into a separation funnel and extracted with DCM (4 x 50 mL). The combined organic layers were dried over Na_2SO_4 and concentrated in vacuum. The crude product was purified via fractionated distillation (62 °C, 49 mbar).

Yield: 704 mg (21%), colorless liquid, $C_5H_{10}O_2S$ [134.19 g/mol].

^{1}H NMR (300 MHz, $CDCl_3$): δ = 4.63 (s, 1H; COSH), 3.32 (s, 3H; OCH_3), 1.36 (s, 6H; CH_3) ppm; **^{13}C NMR** (76 MHz, $CDCl_3$, APT): δ = 205.9 (C_q, CO), 83.7 (C_q), 52.4 (OCH_3), 23.5 (CH_3) ppm; **b.p.**$^{exp.}$ = 62 °C, 49 mbar, (**b.p.**$^{lit.}$ = 40-55 °C, 5-10 torr).

3.10.6 ***S*-((5-Iodopyridin-3-yl)methyl) 2-methoxy-2-methylpropanethioate**

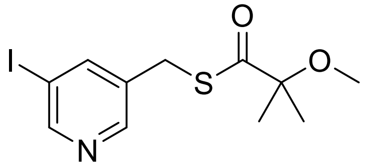

44a

A 5 mL one-neck-round-bottom-flask was charged with 127 mg 3-(chloromethyl)-5-iodopyridine (**42**) (0.501 mmol, 1.0 eq) and a solution of 81 mg 2-methoxy-2-methylpropanethioic *S*-acid (**43**) (0.60 mmol, 1.2 eq) in 1.5 mL THF. After the addition of 151 mg K_2CO_3 (1.09 mmol, 2.2 eq) the yellow suspension was stirred for 5 h at RT. The reaction mixture was quenched by the addition of 10 mL satd. $NaHCO_3$ solution and extracted with DCM (4 x 20 mL). The combined organic layers were dried over Na_2SO_4 and concentrated in vacuum to yield a yellow oil. The crude product was used for the following reaction without further purification.

Yield: 176 mg (quantitative yield), yellow oil, $C_{11}H_{14}INO_2S$ [351.20 g/mol].

^{1}H NMR (300 MHz, $CDCl_3$): δ = 8.69 (d, $^4J_{H,H}$ = 1.7 Hz, 1H; H^{Ar}), 8.49 (d, $^4J_{H,H}$ = 1.5 Hz, 1H; H^{Ar}), 7.95 (bs, 1H; H^{Ar}), 3.95 (s, 2H; CH_2), 3.29 (s, 3H; OCH_3), 1.38 (s, 6H; CH_3) ppm; **^{13}C NMR** (76 MHz, $CDCl_3$): δ = 205.1 (C_q, CO), 154.6 (C^{Ar}), 148.6 (C^{Ar}), 144.6 (C^{Ar}), 136.2 (C_q, C^{Ar}), 93.4

(C_q, C^{Ar}), 83.4 (C_q), 52.3 (OCH_3), 29.3 (CH_2), 23.9 (CH_3) ppm; **GC-MS** (EI, 70 eV; MT_50_S): t_R = 7.34 min; *m/z* (%): 251 (18) [$M^+{-}C_5H_9O_2$], 73 (100) [$M^+{-}C_7H_5INOS$].

3.10.7 ***S*-((5-(6-methyl-4,8-dioxo-1,3,6,2-dioxazaborocan-2-yl)pyridin-3-yl)methyl) 2-methoxy-2-methylpropanethioate**

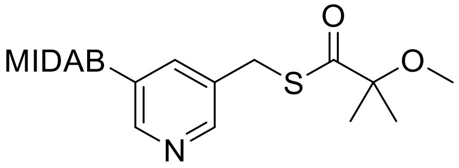

44

A flame dried and nitrogen flushed Schlenk flask was charged with 200 mg *S*-((5-iodopyridin-3-yl)methyl) 2-methoxy-2-methylpropanethioate (**44a**) (570 µmol, 1.0 eq) and dissolved in 1.8 mL absolute THF. After cooling the solution to -78 °C 370 µL *i*PrMgCl·LiCl solution (627 µmol, 1.7 M, 1.1 eq) were added dropwise. After 1 h complete metal-halogen exchange was detected by GC-MS. After adding 108 µL trimethyl borate (969 µmol, 1.7 eq) the reaction mixture was allowed to warm up in the cooling bath overnight. Afterwards the colorless suspension was cooled to -20 °C and 1.14 mL 1M HCl were added, causing the formation of a biphasic mixture, which was stirred for 1 h. Then the cooling bath was removed and the phases were separated. The aqueous phase was neutralized by addition of 5M NaOH, saturated with NaCl and extracted with THF (5 x 2 mL). The combined organic layers were dried over Na_2SO_4 and concentrated in vacuum to yield a yellow solid, which was transferred into a nitrogen flushed 25 mL round-bottom-flask equipped with a Schlenk adapter. After the addition of 126 mg methyliminodiacetic acid (0.856 mmol, 1.5 eq) and 2 mL abs. DMF the reaction mixture was heated to 85 °C overnight. Then the solvent was removed in vacuum and the crude product was purified via flash column chromatography (25 g SiO_2, 8 x 3 cm, EtOAc/MeCN = 100/0 → 0/100).

Yield: 110 mg (51%), colorless solid, $C_{16}H_{21}BN_2O_6S$ [380.22 g/mol].

TLC: R_f = 0.58 (CH_3CN, UV and CAM), **^{1}H NMR** (300 MHz, [D6]DMSO): δ = 8.50 (bs, 1H; H^{Ar}), 8.46 (bs, 1H; H^{Ar}), 7.74 (bs, 1H; H^{Ar}), 4.37 (d, $^2J_{H,H}$ = 17.2 Hz, 2H; CH_2), 4.15 (d, $^2J_{H,H}$ = 17.2 Hz, 2H; CH_2), 4.07 (s, 2H; CH_2), 3.19 (s, 3H; OCH_3), 2.54 (s, 3H; NCH_3), 1.28 (s, 6H; CH_3) ppm; **^{13}C NMR** (76 MHz, [D6]DMSO, APT): δ = 204.9 (C_q, CO), 169.2 (C_q, CO), 151.8

(C^{Ar}), 150.1 (C^{Ar}), 140.5 (C^{Ar}), 132.9 (C_q, C^{Ar}), 82.7 (C_q), 61.9 (CH_2), 51.7 (OCH_3), 47.7 (CH_3), 29.0 (CH_2), 23.5 (CH_3) ppm.[16]

3.10.8 3-(((*tert*-Butyldiphenylsilyl)oxy)methyl)-5-iodopyridine

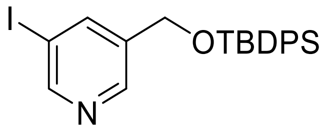

45a

In a 50 mL one-neck round-bottom-flask 1.00 g (5-iodopyridin-3-yl)methanol (**41**) (4.17 mmol, 1.0 eq) and 710 mg imidazole (10.42 mmol, 2.5 eq) were dissolved in 19 mL DCM. After addition of 1.0 mL TBDPSCl (3.75 mmol, 0.9 eq) a white precipitate started forming. The reaction mixture was stirred overnight and transferred into a separation funnel. After addition of 30 mL NaOH (3M) the phases were separated and the aqueous phase extracted with DCM (1 x 30 mL). The combined organic phases were washed with satd. NaCl solution (1 x 40 mL), dried over Na_2SO_4 and concentrated in vacuum. The crude product was purified via flash column chromatography (100 g SiO_2, 6 x 9 cm, cyclohexane/EtOAc = 20/1, R_f = 0.17, UV).

Yield: 1.47 g (83%), colorless oil, $C_{22}H_{24}INOSi$ [473.43 g/mol].

^{1}H NMR (300 MHz, $CDCl_3$): δ = 8.72 (d, $^4J_{H,H}$ = 1.6 Hz, 1H; H^{Ar}), 8.47 (bs, 1H; H^{Ar}), 7.94 (bs, 1H; H^{Ar}), 7.67-7.65 (m, 4H; H^{Ar}), 7.48-7.37 (m, 6H; H^{Ar}), 4.70 (s, 2H; CH_2), 1.10 (s, 9H; CH_3) ppm; **^{13}C NMR** (76 MHz, $CDCl_3$): δ = 154.4 (C^{Ar}), 146.5 (C^{Ar}), 142.2 (C^{Ar}), 138.3 (C_q, C^{Ar}), 135.6 (C^{Ar}), 132.8 (C_q, C^{Ar}), 130.0 (C^{Ar}), 127.9 (C^{Ar}), 93.3 (C_q, C^{Ar}), 62.8 (CH_2), 26.8 (CH_3), 19.3 (C_q) ppm; **GC-MS** (EI, 70 eV; MT_50_S): t_R = 9.97 min; *m/z* (%): 416 (100) [M^+−*t*Bu]; **HRMS** (EI): calcd (*m*/*z*) for [M^+]: 473.0672; found: 473.0697.

3.10.9 3-(((*tert*-Butyldiphenylsilyl)oxy)methyl)-5-(4,4,5,5-tetramethyl-1,3,2-dioxaborolan-2-yl)pyridine

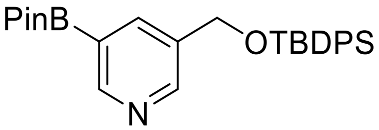

45

[16] Signal for the quaternary *ipso*-pyridine carbon (C_q; C^{Ar}) at the boronic acid MIDA ester function was not observed.

45 was prepared according to general procedure 2.3 from 1.19 g **45a** (2.51 mmol, 1.0 eq) in 13 mL abs. THF, 1.8 mL iPrMgCl·LiCl solution (1.71M in THF) (3.0 mmol, 1.2 eq) and 0.77 mL PinBO*i*Pr (3.8 mmol, 1.5 eq). The crude product was purified via sublimation (160 °C, $3{\cdot}10^{-3}$ mbar).

Yield: 0.94 g (79%), colorless wax like solid, $C_{28}H_{36}BNO_3Si$ [473.50 g/mol].

^{1}H NMR (300 MHz, $CDCl_3$): δ = 8.84 (bs, 1H; H^{Ar}), 8.70 (bs, 1H; H^{Ar}), 7.92 (bs, 1H; H^{Ar}), 7.69-7.67 (m, 4H; H^{Ar}), 7.44-7.36 (m, 6H; H^{Ar}), 4.75 (s, 2H; CH_2), 1.35 (s, 12H; CH_3), 1.09 (s, 9H; CH_3) ppm; **^{13}C NMR** (76 MHz, $CDCl_3$): δ = 154.3 (C^{Ar}), 150.7 (C^{Ar}), 140.5 (C^{Ar}), 135.7 (C^{Ar}) 135.4 (C_q, C^{Ar}), 133.3 (C_q, C^{Ar}), 130.0 (C^{Ar}), 127.9 (C^{Ar}); 84.3 (C_q), 63.8 (CH_2), 27.0 (CH_3), 25.0 (CH_3), 19.4 (C_q) ppm;[17] **GC-MS** (EI, 70 eV; MT_50_S): t_R = 11.48 min; *m/z* (%): 416 (100) [M^+−*t*Bu]; **HRMS** (EI): calcd (*m*/*z*) for [M-H^+]: 472.2485; found: 472.2533.

4 Experimental Procedures and Analytical Data for Teraryl Synthesis

4.1 Methyl 2-(5-(4-(5-((1*H*-indol-3-yl)methyl)pyridin-3-yl)-2-isopropylphenyl)pyridin-3-yl)acetate

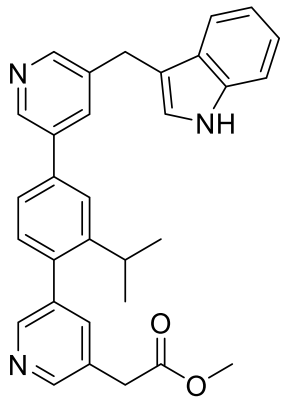

46a

46a was prepared according to general procedure 2.4 from 200 mg pyridine building block **17** (599 µmol, 1.0 eq), 166 mg K_2CO_3 (1.20 mmol, 2.0 eq), 19.5 mg $PdCl_2$(dppf) (24.0 µmol, 4 mol%) and 236 mg 4-iodo-2-isopropylphenyl trifluoromethanesulfonate[17] (599 µmol, 1.0 eq) in 3 mL abs., degassed DMF. The brown crude product was purified via flash column chromatography

[17] Signal for the quaternary *ipso*-pyridine carbon (C_q; C^{Ar}) at the boronic acid pinacol ester function was not observed.

(15 g SiO_2, 2.5 x 12 cm, eluent: cyclohexane/EtOAc = 3/1 → 4/3, R_f = 0.38 (cyclohexane/EtOAc = 1/1, UV)) to give 195 mg (410 µmol, 69%) diaryl intermediate as a brown oil.

The second coupling was performed according to general procedure 2.5 from 131 mg pyridine building block **11** (473 µmol, 1.2 eq), 266 mg Cs_2CO_3 (823 µmol, 2.0 eq), 13.4 mg $PdCl_2$(dppf) (16.4 µmol, 4 mol%) and the previously prepared intermediate in 2.5 mL abs., degassed DMF. The crude product was purified via flash column chromatography (25 g SiO_2, 2.5 x 20 cm, eluent: cyclohexane/EtOAc = 1/1 → 1/9). The product was obtained as a mixture of methyl (Me) and isopropyl (*i*Pr) ester and was used in the following step without further purification.

Yield: 101 mg (37% over 2 steps), pale brown oil, $C_{31}H_{29}N_3O_2$ [475.58 g/mol].

TLC: R_f = 0.37 (methyl ester), 0.51 (isopropyl ester), EtOAc, UV); **^{1}H NMR** (300 MHz, $CDCl_3$): δ = 8.73 (s, 1 H; H^{Ar}), 8.61 (s, 1 H; H^{Ar}), 8.53 (s, 1 H; H^{Ar}), 8.50 (s, 1 H; H^{Ar}), 8.40 (bs, 1 H; NH), 7.80 (s, 1 H; H^{Ar}), 7.64-7.51 (m, 3 H; 2 x H^{Pyr}, H^{Ar}), 7.44-7.34 (m, 2 H; H^{Ar}, H^{Ar}), 7.29-7.16 (m, 2 H; H^{Ar}, H^{Ar}), 7.11 (t, ^3J (H,H) = 7.3 Hz, 1 H; H^{Ar}), 6.99 (s, 1 H; H^{Ar}), 5.12-4.97 (m, 0.6 H; CH^{iPr}), 4.21 (s, 2 H; CH_2^{Ar}), 3.77-3.62 (m, 3.2 H; CH_3^{Me}, CH_2), 3.08-2.96 (m, 1 H; CH), 1.36-1.10 (m, 9.6 H; CH_3^{Me}, CH_3^{iPr}) ppm; **^{13}C NMR** (75.53 MHz, $CDCl_3$, APT): δ = 171.1 (C_q; $C{=}O^{Me}$), 170.2 (C_q; $C{=}O^{iPr}$), 149.1 (C^{Ar}), 148.9 (C^{Ar}), 148.7 (C^{Ar}), 148.6 (C^{Ar}), 147.6 (2 x C_q; C^{Ar}), 146.1 (C^{Ar}), 138.4 (C_q; C^{Ar}), 138.3 (C_q; C^{Ar}), 137.6 (2 x C^{Ar}), 136.9 (2 x C_q; C^{Ar}), 136.8 (C_q; C^{Ar}), 136.7 (2 x C_q; C^{Ar}), 136.3 (C_q; C^{Ar}), 134.9 (C^{Ar}), 130.9 (C^{Ar}), 129.6 (C_q; C^{Ar}), 129.3 (C_q; C^{Ar}), 127.3 (C_q; C^{Ar}), 124.9 (C^{Ar}), 124.8 (C^{Ar}), 124.7 (C^{Ar}), 122.7 (C^{Ar}), 122.4 (C^{Ar}), 119.7 (C^{Ar}), 119.0 (C^{Ar}), 114.6 (C_q; C^{Ar}), 111.4 (C^{Ar}), 68.9 (CH^{iPr}), 52.4 (CH_3^{Me}), 38.9 ($CH_2^{Asp,\,iPr}$), 38.3 ($CH_2^{Asp,\,Me}$), 29.7 (CH^{Val}), 29.1 (CH_2^{Ind}), 24.3 (CH_3^{Val}), 21.9 (CH_3^{iPr}) ppm; **HPLC-MS** (Poroshell, ESI^+, MV_general): t_R = 7.17 min; m/z: 476 [M+H^+].

4.2 Trp-Val-Asp

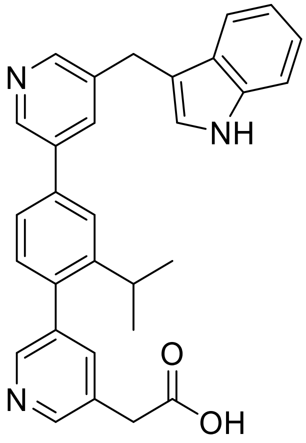

46

In a 10 mL round-bottom flask 74 mg previously prepared intermediate **46a** (0.16 mmol, 1.0 eq) was dissolved in a 10 mL round-bottom flask in 3 mL THF. A solution of 131 mg $LiOH \cdot H_2O$ (3.12 mmol, 20.0 eq) in 3 mL H_2O was added and the resulting heterogenic mixture was stirred at RT. After full conversion was detected via TLC. 3.1 mL aq. HCl (1M) were added into the cold (0 ˚C) reaction mixture until a pH of 7 was reached. The solution was diluted with 20 mL satd. NH_4Cl and extracted with EtOAc (3 x 20 mL). The organic layer was dried over Na_2SO_4, filtered and evaporated under reduced pressure. The crude product was purified via silica gel filtration (10 g SiO_2, 8 x 2.5 cm, eluent: EtOAc/MeOH = 1/0 → 1/1).

Yield: 60 mg (84%), pale orange powder, $C_{30}H_{27}N_3O_2$ [461.55 g/mol].

TLC: R_f = 0.23 (EtOAc/MeOH = 3/2, UV); **^{1}H NMR** (300 MHz, [D6]DMSO): δ = 10.99 (s, 1H; NH), 8.74 (s, 1H; H^{Ar}), 8.55 (s, 1H; H^{Ar}), 8.48 (s, 1H; H^{Ar}), 8.38 (s, 1H; H^{Ar}), 8.02 (s, 1H; H^{Ar}), 7.70 (s, 1H; H^{Ar}), 7.64 (s, 1H; H^{Ar}), 7.60-7.48 (m, 2H; H^{Ar}), 7.36 (d, $^3J_{H,H}$ = 7.9 Hz, 1H; H^{Ar}), 7.30-7.21 (m, 2H; H^{Ar}), 7.06 (t, $^3J_{H,H}$ = 7.3 Hz, 1H; H^{Ar}), 6.96 (t, $^3J_{H,H}$ = 7.2 Hz, 1H; H^{Ar}), 4.16 (s, 2H; CH_2), 3.64 (s, 2H; CH_2), 3.03-2.89 (m, 1H; CH), 1.17 (d, $^3J_{H,H}$ = 6.5 Hz, 6H; CH_3) ppm; **^{13}C NMR** (75.53 MHz, [D6]DMSO, APT): δ = 172.8 (C_q; CO), 149.0 (C^{Ar}), 148.6 (C^{Ar}), 147.0 (C_q; C^{Ar}), 145.2 (C^{Ar}), 137.4 (C_q; C^{Ar}), 137.3 (C^{Ar}, C_q; C^{Ar}), 136.6 (C_q; C^{Ar}), 136.4 (C_q; C^{Ar}), 135.7 (C_q; C^{Ar}), 135.0 (C_q; C^{Ar}), 134.0 (C^{Ar}), 131.5 (C_q; C^{Ar}), 130.7 (C^{Ar}), 126.8 (C_q; C^{Ar}), 124.3 (C^{Ar}), 124.2 (C^{Ar}), 123.4 (C^{Ar}), 121.1 (C^{Ar}), 118.4 (2 x C^{Ar}), 113.0 (C_q; C^{Ar}), 111.5 (C^{Ar}), 38.8 (CH_2), 29.2 (CH), 28.1 (CH_2), 23.9 (CH_3) ppm; **HPLC-MS** (Poroshell, ESI^+, MV_general): t_R = 5.97 min; *m/z*: 462 [M+H^+]; **HRMS** (MALDI): calcd (*m/z*) for [M^++H]: 462.2181; found: 462.2195.

4.3 4-(5-(4-(5-Benzylpyridin-3-yl)-2-methylphenyl)pyridin-3-yl)butanenitrile

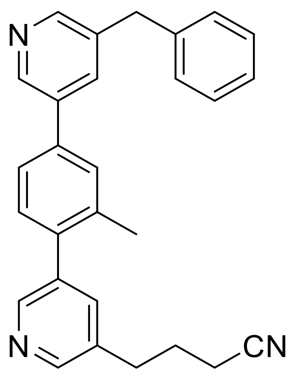

47a

47a was prepared according to general procedure 2.4 from 329 mg pyridine building block **9** (1.89 mmol, 1.1 eq), 744 mg K_2CO_3 (5.40 mmol, 3.0 eq), 73.5 mg $PdCl_2$(dppf) (90.0 µmol, 5 mol%) and 528 mg 1-bromo-4-iodo-2-methylbenzene (1.80 mmol, 1.0 eq) in 9 mL abs., degassed DMF. The crude product was purified via flash column chromatography (35 g SiO_2, 3.5 x 15 cm, eluent: cyclohexane/EtOAc = 6/1 → 4/1, R_f = 0.34 (cyclohexane/EtOAc = 3/1, UV)) to give 403 mg (67%) diaryl intermediate as a brown oil.

The second coupling was performed according to general procedure 2.5 from 105 mg pyridine building block **29** (387 µmol, 1.2 eq), 266 mg Cs_2CO_3 (966 µmol, 3.0 eq), 13.1 mg $PdCl_2$(dppf) (16.1 µmol, 5 mol%), 109 mg previously prepared intermediate (322 µmol, 1.0 eq) and 3 mL abs., degassed DMF. The crude product was purified via flash column chromatography (25 g SiO_2, 2.5 x 20 cm, eluent: cyclohexane/EtOAc = 2/1 → 1/4).

Yield: 99 mg (51% over 2 steps), colorless powder, $C_{28}H_{25}N_3$ [403.53 g/mol].

TLC: R_f = 0.23 (cyclohexane/EtOAc = 1/1, UV); **^{1}H NMR** (300 MHz, $CDCl_3$): δ = 8.73 (s, 1H; H^{Ar}), 8.61-8.39 (m, 3H; 3x H^{Ar}), 7.69 (s, 1H; H^{Ar}), 7.57-7.40 (m, 3H; 3x H^{Ar}), 7.38-7.17 (m, 6H; 6x H^{Ar}), 4.07 (s, 2H; CH_2), 2.87 (t, $^3J_{H,H}$ = 7.5 Hz, 2H; CH_2), 2.42 (t, $^3J_{H,H}$ = 6.9 Hz, 2H; CH_2), 2.34 (s, 3H; CH_3), 2.14-1.95 (m, 2H; CH_2) ppm; **^{13}C NMR** (76 MHz, $CDCl_3$): δ = 148.9 (C^{Ar}), 148.4 (C^{Ar}), 148.3 (C^{Ar}), 146.0 (C^{Ar}), 139.7 (C_q; C^{Ar}), 137.8 (C_q; C^{Ar}), 137.7 (C_q; C^{Ar}), 136.9 (C_q; C^{Ar}), 136.8 (C_q; C^{Ar}), 136.6 (C^{Ar}), 136.5 (C_q; C^{Ar}), 136.2 (C_q; C^{Ar}), 135.2 (C^{Ar}), 134.8 (C_q; C^{Ar}), 130.7 (C^{Ar}), 129.5 (C^{Ar}), 129.0 (C^{Ar}), 128.9 (C^{Ar}), 126.8 (C^{Ar}), 125.1 (C^{Ar}), 119.1 (C_q; CN), 39.2 (CH_2), 31.7 (CH_2), 26.8 (CH_2), 20.7 (CH_3), 16.8 (CH_2) ppm; **m.p.^{exp}** = 83 °C; **HRMS** (MALDI): calcd for [M^++H]: 404.2127; found: 404.2109.

4.4 Phe-Ala-Lys

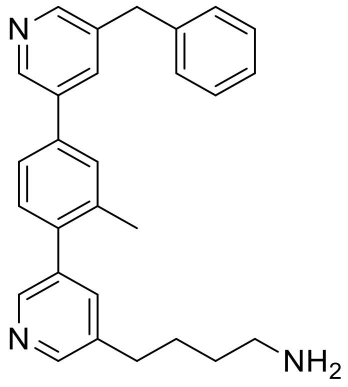

47

A solution of 30 mg nitrile **47a** (84.3 µmol, 1.0 eq) in 7.5 mL MeOH/NH_3 (aq.) (20/1) was subjected to hydrogenation with a flow reactor H-Cube^{TM} with a Raney nickel cartridge (THS 01112) at 70 °C and 70 bar in continuous flow mode of 0.5 mL/min. After full conversion was detected via HPLC-MS, the solvent was removed under reduced pressure.

Yield: 30 mg (quantitative), colorless oil, $C_{25}H_{29}N_3$ [407.56 g/mol].

^{1}H NMR (300 MHz, $CDCl_3$): δ = 8.65 (s, 1H; H^{Ar}), 8.43 (s, 1H; H^{Ar}), 8.41-8.30 (m, 2H; H^{Ar}), 7.61 (s, 1H; H^{Ar}), 7.46-7.30 (m, 3H; H^{Ar}), 7.30-7.05 (m, 6H; H^{Ar}), 3.99 (s, 2H; CH_2), 2.79-2.52 (m, 6H; CH_2, NH_2), 2.26 (s, 3H; CH_3), 1.73-1.41 (m, 4H; CH_2) ppm; **^{13}C NMR** (75.53 MHz, $CDCl_3$, APT): δ = 149.2 (C^{Ar}), 148.6 (C^{Ar}), 147.5 (C^{Ar}), 146.3 (C^{Ar}), 139.8 (C_q; C^{Ar}), 138.2 (C_q; C^{Ar}), 137.7 (C_q; C^{Ar}), 137.0 (C_q; C^{Ar}), 136.6 (C_q; C^{Ar}), 136.5 (C_q; C^{Ar}), 136.4 (C^{Ar}), 136.1 (C_q; C^{Ar}), 134.8 (C^{Ar}), 130.7 (C^{Ar}), 129.4 (C^{Ar}), 129.0 (C^{Ar}), 128.9 (C^{Ar}), 126.7 (C^{Ar}), 125.0 (C^{Ar}), 41.7 (CH_2), 39.2 (CH_3), 32.8 (CH_2), 32.5 (CH_2), 28.5 (CH_2), 20.7 (CH_2) ppm; **HRMS** (MALDI): calcd (*m/z*) for [M^++H]: 407.2361; found: 407.2365.

4.5 Methyl 3-(2-(5-(hydroxymethyl)pyridin-3-yl)-5-(5-methylpyridin-3-yl)phenyl)propanoate

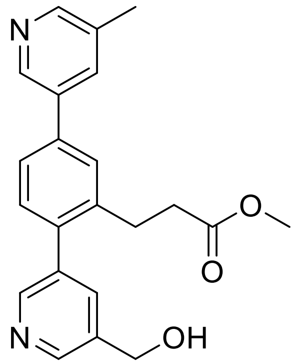

48a

48a was prepared according to general procedure 2.4 from 277 mg pyridine building block **36** (1.26 mmol, 1.1 eq), 316 mg K_2CO_3 (2.29 mmol, 2.0 eq), 43 mg $PdCl_2$(dppf) (58.8 µmol, 5 mol%) and 500 mg methyl 3-(5-iodo-2-(((trifluoromethyl)sulfonyl)oxy)phenyl)propanoate[18] (1.14 mmol, 1.0 eq) in 10 mL abs., degassed DMF. The brown crude product was purified via flash column chromatography (40 g SiO_2, 3 x 20 cm, eluent: cyclohexane/EtOAc = 1/1, R_f = 0.21) to give 200 mg (43%) diaryl intermediate as an orange oil.

The second coupling was performed according to general procedure 2.5 from 226 mg pyridine building block **45** (477 µmol, 1.1 eq), 281 mg Cs_2CO_3 (862 µmol, 2.0 eq), 16.1 mg $PdCl_2$(dppf) (22.0 µmol, 5 mol%) and 175 mg previously prepared intermediate (434 µmol, 1.0 eq) in 4 mL abs., degassed DMF. Deprotection of the serine residue occurred already under coupling conditions. The crude product was purified via flash column chromatography (8 g SiO_2, 1 x 12 cm, eluent: DCM/MeOH = 25/1 → 15/1).

Yield: 92 mg (25% over 2 steps), pale orange oil, $C_{22}H_{22}N_2O_3$ [362.43 g/mol].

TLC: R_f = 0.23 (DCM/MeOH = 15/1, UV and CAM); **^{1}H NMR** (300 MHz, $CDCl_3$): δ = 8.64-8.61 (m, 2H; 2x H^{Ar}), 8.51 (s, 1H; H^{Ar}), 8.42 (s, 1H; H^{Ar}), 7.75 (s, 1H; H^{Ar}), 7.70 (s, 1H; H^{Ar}), 7.51-7.46 (m, 2H; H^{Ar}), 7.28 (d, $^3J_{H,H}$ = 8.0 Hz, 1H; H^{Ar}), 4.83 (s, 2H; CH_2), 3.59 (s, 3H; CH_3), 3.10-2.85 (m, 3H; CH_2, OH), 2.50 (t, $^3J_{H,H}$ = 7.8 Hz, 2H; CH_2), 2.42 (s, 3H; CH_3) ppm; **^{13}C NMR** (75.53 MHz, $CDCl_3$, APT): δ = 173.0 (C_q; CO), 149.2 (C^{Ar}), 148.7 (C^{Ar}), 147.4 (C^{Ar}), 145.4 (C^{Ar}), 139.2 (C_q; C^{Ar}), 138.2 (C^{Ar}, C_q; C^{Ar}), 137.8 (C_q; C^{Ar}), 136.5 (C_q; C^{Ar}), 136.4 (C_q; C^{Ar}), 135.7 (C_q; C^{Ar}), 135.4 (C^{Ar}), 135.2 (C^{Ar}), 133.4 (C_q; C^{Ar}), 131.3 (C^{Ar}), 128.1 (C^{Ar}), 125.5 (C^{Ar}), 62.5 (CH_2), 51.9

(CH_3), 35.2 (CH_2), 28.4 (CH_2), 18.6 (CH_3) ppm; **HPLC-MS** (Poroshell, ESI$^+$, MV_general): t_R = 3.21 min; *m/z*: 363 [*M*+H$^+$]; **HRMS** (MALDI): calcd (*m/z*) for [*M*$^+$]: 362.1630; found: 362.1628.

4.6 Ala-Glu-Ser

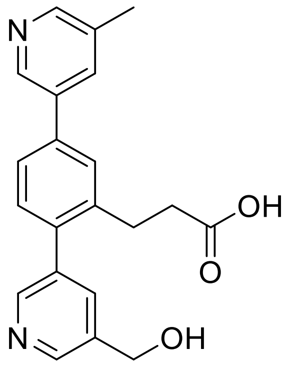

48

In a 10 mL round-bottom flask equipped with stirring bar 40 mg previously prepared intermediate **48a** (110 µmol, 1.0 eq) were dissolved in 2 mL THF. A solution of 46.0 mg LiOH•H_2O (1.10 mmol, 10.0 eq) in 1 mL H_2O was added and the orange solution was stirred at RT. After full conversion was detected by TLC 1.1 mL aq. HCl (1M) was added into the cold (0 ˚C) reaction mixture until a pH of 7 was reached. Workup via extraction was not possible as the compound remained in the aqueous layer. Therefore, the reaction mixture was directly concentrated under reduced pressure and subsequently purified via semi-preparative HPLC (MV_NucleodurC18_001HCOOH_2to30).

Yield: 22.0 mg (58%), colourless powder, $C_{21}H_{20}N_2O_3$ [348.40 g/mol].

TLC: R_f = 0.09 (DCM/MeOH = 5/1, UV and CAM); **^{1}H NMR** (300 MHz, [D6]DMSO): δ = 8.75 (s, 1H; H^{Ar}), 8.56 (s, 1H; H^{Ar}), 8.47-8.43 (m, 2H; 2x H^{Ar}), 7.97 (s, 1H; H^{Ar}), 7.75-7.72 (m, 2H; 2x H^{Ar}), 7.65 (dd, $^3J_{H,H}$ = 7.8, $^4J_{H,H}$ = 1.5 Hz, 1H; H^{Ar}), 7.32 (d, $^3J_{H,H}$ = 7.8 Hz, 1H; H^{Ar}), 4.62 (s, 2H; CH_2), 2.85 (t, $^3J_{H,H}$ = 7.7 Hz, 2H; CH_2), 2.46 (t, $^3J_{H,H}$ = 7.7 Hz, 2H; CH_2 (overlaps with solvent peak)), 2.39 (s, 3H; CH_3) ppm; **^{13}C NMR** (75.53 MHz, [D6]DMSO, APT): δ = 173.9 (C_q; CO), 149.0 (C^{Ar}), 147.7 (C^{Ar}), 146.9 (C^{Ar}), 144.8 (C^{Ar}), 139.4 (C_q; C^{Ar}), 137.6 (C_q; C^{Ar}), 137.3 (C_q; C^{Ar}), 136.9 (C_q; C^{Ar}), 135.7 (C_q; C^{Ar}), 134.6 (C_q; C^{Ar}), 134.6 (C^{Ar}), 134.2 (C^{Ar}), 133.1 (C_q; C^{Ar}), 130.8 (C^{Ar}), 127.6 (C^{Ar}), 124.7 (C^{Ar}), 60.5 (CH_2), 34.9 (CH_2), 27.8 (CH_2), 17.9 (CH_3) ppm; **HPLC-MS** (Poroshell, ESI$^+$, MV_general): t_R = 2.92 min; *m/z*: 349 [*M*+H$^+$]; **HRMS** (MALDI): calcd (*m/z*) for [*M*$^+$+H]: 349.1544; found: 349.1544.

4.7 3-(5-(4-(5-Isobutylpyridin-3-yl)-2-methylphenyl)pyridin-3-yl)propanenitrile

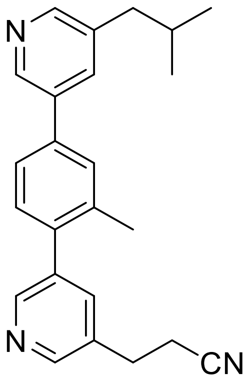

49

49 was prepared according to general procedure 2.4 from 329 mg pyridine building block **39** (1.26 mmol, 1.1 eq), 496 mg K_2CO_3 (3.60 mmol, 3.0 eq), 49.0 mg $PdCl_2$(dppf) (60.0 µmol, 5 mol%) and 352 mg 1-bromo-4-iodo-2-methylbenzene (1.20 mmol, 1.0 eq) in 6 mL abs., degassed DMF. The crude product was purified via flash column chromatography (25 g SiO_2, 2.0 x 16 cm, eluent: cyclohexane/EtOAc = 9/1 → 5/1, R_f = 0.37 (cyclohexane/EtOAc = 3/1, UV)) to give 263 mg (73%) diaryl intermediate as a brown oil.

The second coupling was performed according to general procedure 2.5 from 83.4 mg pyridine building block **15** (323 µmol, 1.1 eq), 282 mg Cs_2CO_3 (870 µmol, 3.0 eq), 11.8 mg $PdCl_2$(dppf) (14.5 µmol, 5 mol%), 89.4 mg previously prepared intermediate (290 µmol, 1.0 eq) and 3 mL abs., degassed DMF. The crude product was purified via flash column chromatography (10 g SiO_2, 2 x 10 cm, eluent: cyclohexane/EtOAc = 1/1 → EtOAc/MeOH = 99/1).

Yield: 73 mg (51% over 2 steps), colorless powder, $C_{24}H_{25}N_3$ [355.49 g/mol].

TLC: R_f = 0.21 (EtOAc, UV); **^{1}H NMR** (300 MHz, $CDCl_3$): δ = 8.71 (s, 1H; H^{Ar}), 8.57 (s, 1H; H^{Ar}), 8.52 (s, 1H; H^{Ar}), 8.41 (s, 1H; H^{Ar}), 7.68 (s, 1H; H^{Ar}), 7.61 (s, 1H; H^{Ar}), 7.56-7.43 (m, 2H; 2x H^{Ar}), 7.33 (d, $^3J_{H,H}$ = 7.7 Hz, 1H; H^{Ar}), 3.05 (t, $^3J_{H,H}$ = 7.0 Hz, 2H; CH_2), 2.72 (t, $^3J_{H,H}$ = 7.1 Hz, 2H; CH_2), 2.56 (d, $^3J_{H,H}$ = 7.1 Hz, 2H; CH_2), 2.37 (s, 3H; CH_3^{Ala}), 2.05-1.82 (m, 1H; CH), 0.96 (d, $^3J_{H,H}$ = 6.5 Hz, 6H; 2 x CH_3) ppm; **^{13}C NMR** (76 MHz, $CDCl_3$): δ = 149.6 (C^{Ar}), 149.1 (C^{Ar}), 148.3 (C^{Ar}), 145.9 (C^{Ar}), 138.2 (C_q; C^{Ar}), 137.4 (C_q; C^{Ar}), 137.1 (C_q; C^{Ar}), 136.8 (C_q; C^{Ar}), 136.6 (C_q; C^{Ar}), 136.5 (C^{Ar}), 135.6 (C_q; C^{Ar}), 135.0 (C^{Ar}), 133.0 (C_q; C^{Ar}), 130.7 (C^{Ar}), 129.5 (C^{Ar}), 125.1 (C^{Ar}), 118.5 (CN), 42.5 (CH_2), 30.2 (CH), 28.9 (CH_2), 22.4 (CH_3), 20.7 (CH_2), 19.3 (CH_2)

ppm; **HPLC-MS** (Poroshell, ESI$^+$, MV_general): t_R = 6.63 min; m/z: 356 [M+H$^+$]; **m.p.^{exp}** = 115-116 °C; **HRMS** (DI-EI): calcd (*m/z*) for [M^+]: 355.2048; found: 355.2050.

4.8 Leu-Ala-Arg

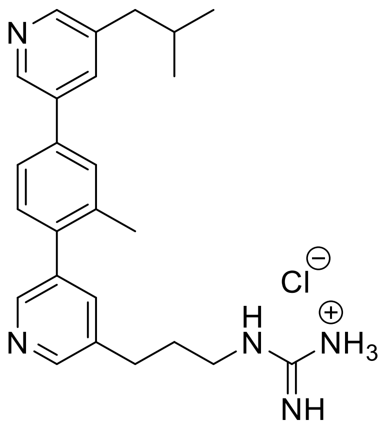

52

A solution of 35.5 mg nitrile **49** (100 µmol, 1.0 eq) in 5 mL MeOH/NH_3 (aq.) (20/1) was subjected to hydrogenation with a flow reactor H-Cube^{TM} with a Raney nickel cartridge (THS 01112) at 70 °C and 70 bar in a continuous flow mode of 1.0 mL/min. After full conversion was detected via HPLC-MS, the solvent was removed under reduced pressure. The residue was dissolved in 2 mL DMF in a 10 mL round-bottom flask and 31 mg *N*,*N'*-di-Boc-1*H*-pyrazole-1-carboxamidine **51** (100 µmol, 1.0 eq) were added. The reaction mixture was stirred at RT for 16 h. The solution was diluted with 10 mL DCM and washed with H_2O (2 x 10 mL). The organic phase was dried over Na_2SO_4 and the solvent was removed under reduced pressure. The crude product was purified via flash column chromatography (10 g SiO_2, 2 x 10 cm, eluent: cyclohexane/EtOAc = 2/1 → 1/1).
In the last deprotection step, the previously prepared di-boc protected intermediate was dissolved in 3 mL DCM/TFA (4/1). The reaction mixture was stirred at RT for 4 h. After detection of full conversion via TLC the solvent was removed under reduced pressure and the crude product was purified via semi-preparative HPLC (MV_NucleodurC18_001HCOOH_2to100). To the collected product fractions 75 µL 1M HCl were added and the solvent was removed under reduced pressure.
Yield: 24.0 mg (56% over 4 steps), colorless solid, $C_{25}H_{32}ClN_5$ [438.02 g/mol].

^{1}H NMR (300 MHz, D_2O): δ = 8.95 (s, 1H; H^{Ar}), 8.77 (s, 1H; H^{Ar}), 8.76-8.69 (m, 2H; H^{Ar}), 8.63 (s, 1H; H^{Ar}), 8.58 (s, 1H; H^{Ar}), 7.79 (s, 1H; H^{Ar}), 7.74 (d, $^3J_{H,H}$ = 8.0 Hz, 1H; H^{Ar}), 7.54 (d, $^3J_{H,H}$ = 7.9 Hz, 1H; H^{Ar}), 3.32 (d, $^3J_{H,H}$ = 6.8 Hz, 2H; CH_2), 3.03 (t, $^3J_{H,H}$ = 7.5 Hz, 2H; CH_2), 2.81 (d,

$^3J_{H,H}$ = 7.1 Hz, 2H; CH_2), 2.38 (s, 3H; CH_3), 2.17-1.92 (m, 3H; CH_2, CH), 0.95 (d, $^3J_{H,H}$ = 6.6 Hz, 6H; CH_3) ppm; **^{13}C NMR** (75.53 MHz, D_2O): δ = 156.8 (C_q; CNH), 147.2 (C^{Ar}), 145.2 (C^{Ar}), 142.5 (C_q; C^{Ar}), 141.8 (C_q; C^{Ar}), 140.1 (C_q; C^{Ar}), 139.5 (C^{Ar}), 139.3 (C^{Ar}), 139.2 (C_q; C^{Ar}), 138.8 (C^{Ar}), 137.5 (C_q; C^{Ar}), 136.8 (C^{Ar}), 135.6 (C_q; C^{Ar}), 135.0 (C_q; C^{Ar}), 130.9 (C^{Ar}), 129.8 (C^{Ar}), 125.3 (C^{Ar}), 41.0 (CH_2), 40.4 (CH_2), 29.3 (CH), 29.1 (CH_2), 28.6 (CH_2), 21.1 (CH_3), 19.3 (CH_3) ppm; **HRMS** (MALDI): calcd (*m*/*z*) for [M^+-HCl]: 401.2579; found: 401.2580.

4.9 Methyl 3-(5-(4-(5-(2-cyanoethyl)pyridin-3-yl)-2-methylphenyl)pyridin-3-yl)propanoate

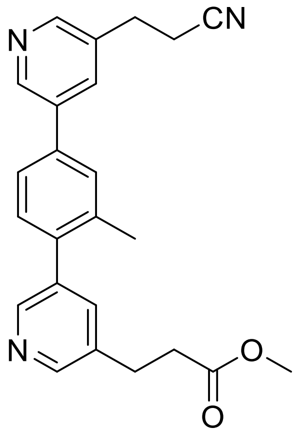

50

50 was prepared according to general procedure 2.4 from 287 mg pyridine building block **15** (1.11 mmol, 1.1 eq), 279 mg K_2CO_3 (2.02 mmol, 2.0 eq), 37.0 mg $PdCl_2$(dppf) (50.0 µmol, 5 mol%) and 300 mg 1-bromo-4-iodo-2-methylbenzene (1.01 mmol, 1.0 eq) in 4 mL abs., degassed DMF. The crude product was purified via flash column chromatography (25 g SiO_2, 2.0 x 16 cm, eluent: cyclohexane/EtOAc = 3/1 → 1/1, R_f = 0.23 (cyclohexane/EtOAc = 1/1, UV and CAM)) to give 290 mg (95%) diaryl intermediate as a brown solid.

The second coupling was performed according to general procedure 2.5 from 294 mg pyridine building block **14** (980 µmol, 1.0 eq), 627 mg Cs_2CO_3 (1.92 mmol, 2.0 eq), 35.0 mg $PdCl_2$(dppf) (47.8 µmol, 5 mol%), 290 mg previously prepared intermediate (960 µmol, 1.0 eq) and 5 mL abs., degassed DMF. The crude product was purified via flash column chromatography (10 g SiO_2, 2 x 10 cm, eluent: EtOAc → EtOAc/MeOH = 10/1). The product was obtained as a mixture of methyl (Me) and isopropyl ester (*i*Pr) (2.7/1), which was used for the following steps without further purification.

Yield: 292 mg (72% over 2 steps), brown oil, $C_{24}H_{23}N_3O_2$ [385.47 g/mol].

TLC: R_f = 0.46 (EtOAc/MeOH = 10/1, UV and CAM); **HPLC-MS** (Poroshell, ESI^+, MV_general): t_R (Me) = 3.641 min; m/z: 386 [M+H^+], t_R (*i*Pr) = 4.242 min; m/z: 414 [M+H^+]; **HRMS** (MALDI): calcd (*m/z*) for [M^++H]: 386.1869; found: 386.1860.

4.10 3-(5-(4-(5-(2-Cyanoethyl)pyridin-3-yl)-2-methylphenyl)pyridin-3-yl)propanamide

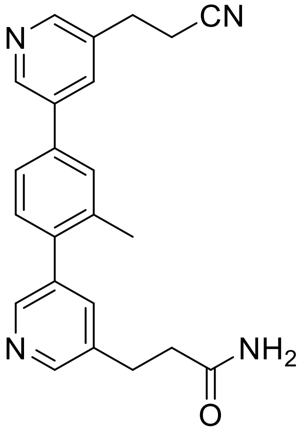

53

A 5 mL "Ace pressure tube®, front seal" (Aldrich Z181099) with a "Duro-Silicone O-ring" was charged with 47 mg compound mixture **50** (120 µmol, 1.0 eq), 6.8 mg KCN (100 µmol, 80 mol%) and 2 mL ammonia solution (7M in MeOH). The flask was sealed, and the mixture was stirred at 50 °C for 2 d. Full conversion was detected via HPLC-MS and the solvent was removed under reduced pressure. The remaining brown residue was triturated with 5 mL Et_2O and the resulting solid was filtered, washed with Et_2O (5 mL) and H_2O (5 mL) and then dried *in vacuo*.

Yield: 17.0 mg (38%), beige solid, $C_{23}H_{22}N_4O$ [370.46 g/mol].

^{1}H NMR (500 MHz, MeOD): δ = 8.81 (d, $^4J_{H,H}$ = 1.9 Hz, 1H; H^{Ar}), 8.55 (d, $^4J_{H,H}$ = 1.6 Hz, 1H; H^{Ar}), 8.51 (d, $^4J_{H,H}$ = 1.6 Hz, 1H; H^{Ar}), 8.44 (d, $^4J_{H,H}$ = 1.7 Hz, 1H; H^{Ar}), 8.15 (s, 1H; H^{Ar}), 7.81 (s, 1H; H^{Ar}), 7.71 (s, 1H; H^{Ar}), 7.66 (d, $^3J_{H,H}$ = 7.8 Hz, 1H; H^{Ar}), 7.42 (d, $^3J_{H,H}$ = 7.9 Hz, 1H; H^{Ar}), 3.15 (t, $^3J_{H,H}$ = 7.1 Hz, 2H; CH_2), 3.10 (t, $^3J_{H,H}$ = 7.4 Hz, 2H; CH_2), 2.93 (t, $^3J_{H,H}$ = 7.1 Hz, 2H; CH_2), 2.66 (t, $^3J_{H,H}$ = 7.4 Hz, 2H; CH_2), 2.41 (s, 3H; CH_3) ppm; **^{13}C NMR** (126 MHz, MeOD): δ = 175.8 (C_q; $CONH_2$), 147.6 (C^{Ar}), 147.4 (C^{Ar}), 146.5 (C^{Ar}), 145.7 (C^{Ar}), 137.8 (C_q; C^{Ar}), 137.4 (C^{Ar}), 137.2 (C_q; C^{Ar}), 136.9 (C_q; C^{Ar}), 136.8 (C_q; C^{Ar}), 136.5 (C_q; C^{Ar}), 136.5 (C_q; C^{Ar}), 135.2 (C_q; C^{Ar}), 135.2 (C^{Ar}), 130.3 (C^{Ar}), 129.0 (C^{Ar}), 124.6 (C^{Ar}), 118.9 (C_q; CN), 36.0 (CH_2), 28.0 (CH_2), 28.0 (CH), 19.2 (CH_3), 17.8 (CH_2) ppm; **HPLC-MS** (Poroshell, ESI^+, MV_general): t_R =

3.142 min; m/z: 371 [M+H$^+$]; **m.p.^{exp}** = 180 °C (decomposition); **HRMS** (MALDI): calcd (*m*/*z*) for [M^++H]: 371.1872; found: 371.1866.

4.11 Gln-Ala-Glu

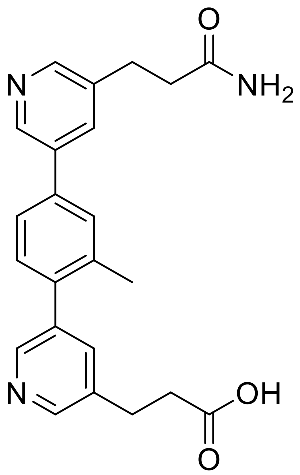

54

A 5 mL "Ace pressure tube®, front seal" (Aldrich Z181099) with a "Duro-Silicone O-ring" was charged with 60 mg compound mixture **50** (160 µmol, 1.0 eq), 180 mg $Ru(H_2)(PPh_3)_4$ (160 µmol, 1.0 eq) and 1.5 mL 1,2-DME/H_2O = 2/1. The tube was sealed and the black suspension was stirred at 120 °C for 24 h. Full conversion was detected via HPLC-MS and the reaction mixture was filtered through a short pad of celite and washed extensively with EtOAc. The filtrate was concentrated under reduced pressure to give intermediate Gln-Ala-Glu ester as a brown solid (47 mg). The intermediate was transferred to a 10 mL round-bottom flask equipped with stirring bar and dissolved in 2 mL THF/MeOH/H_2O = 5/4/1. 53.0 mg LiOH·H_2O (1.26 mmol, 7.9 eq) were added and the orange solution was stirred at RT. After 1.5 h full conversion was detected via TLC and 210 µL 6M HCl were added into the cold (0 ˚C) reaction mixture until a pH of 7 was reached. Workup via extraction was not possible as the compound remained in the aqueous layer. Therefore, the reaction mixture was directly concentrated under reduced pressure and subsequently purified via flash column chromatography (3 g SiO_2, 1.2 x 24 cm, eluent: EtOAc → MeOH).

Yield: 18.0 mg (29%), orange oil, $C_{23}H_{23}N_3O_3$ [389.46 g/mol].

TLC: R_f = 0.62 (MeOH, UV and CAM); **^{1}H NMR** (300 MHz, MeOD): δ = 9.11 (s, 1H; H^{Ar}), 8.90-8.78 (m, 4H; H^{Ar}), 8.66 (s, 1H; H^{Ar}), 7.90 (s, 1H; H^{Ar}), 7.84 (d, $^3J_{H,H}$ = 7.8, 1H; H^{Ar}), 7.58 (d, ^3J

$_{H,H}$ = 7.8 Hz, 1H; H^{Ar}), 3.32-3.22 (m, 4H; CH_2 (overlaps with solvent peak), 2.85 (t, $^3J_{H,H}$ = 7.0 Hz, 2H; CH_2), 2.75 (t, $^3J_{H,H}$ = 7.0 Hz, 2H, CH_2), 2.44 (s, 3H; CH_3) ppm; **^{13}C NMR** (75.53 MHz, MeOD, APT): δ = 176.4 (C_q; CO), 175.3 (C_q; CO), 148.7 (C^{Ar}), 146.0 (C^{Ar}), 143.8 (C_q; C^{Ar}), 143.3 (C_q; C^{Ar}), 141.6 (C^{Ar}), 141.4 (C_q; C^{Ar}), 141.2 (C^{Ar}), 140.7 (C_q; C^{Ar}), 140.3 (C^{Ar}), 138.9 (C_q; C^{Ar}), 138.8 (C^{Ar}), 137.4 (C_q; C^{Ar}), 136.4 (C_q; C^{Ar}), 132.4 (C^{Ar}), 131.2 (C^{Ar}), 126.8 (C^{Ar}), 36.1 (CH_2), 34.7 (CH_2), 29.2 (CH_2), 28.6 (CH_2), 20.3 (CH_3) ppm; **HPLC-MS** (Poroshell, ESI^+, MV_general): t_R = 2.945 min; *m/z*: 349 [M+H^+]; **HRMS** (MALDI): calcd (*m/z*) for [M^++H]: 390.1818; found: 390.1815.

5 Solubility studies

5.1 2'-Methyl-1,1':4',1''-terphenyl

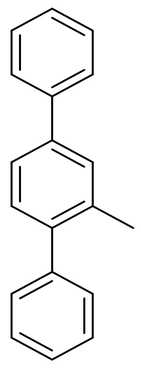

55

Compound **55** was prepared according to procedure 2.5 from 725 mg phenylboronic acid (3.55 mmol, 2.1 eq), 1.65 g Cs_2CO_3 (5.11 mmol, 3.0 eq) and 61.8 mg $PdCl_2$(dppf) (86 µmol, 5 mol%) in 14 mL abs. CH_3CN and 520 mg 1-bromo-4-iodo-2-methylbenzene (1.75 mmol, 1.0 eq). After quantitative conversion was detected by GC-MS (20 h) the catalyst was removed by filtration through a pad of silica gel (eluted with 150 mL EtOAc). The solvent was removed under reduced pressure and the crude product was purified via flash column chromatography (25 g SiO_2, 3.0 x 12 cm, eluent: cyclohexane → cyclohexane/EtOAc = 100/1).
Yield: 200 mg (47%), colorless solid, $C_{19}H_{16}$ [244.34 g/mol].

TLC: R_f = 0.31 (cyclohexane, UV and CAM); **^{1}H NMR** (300 MHz, $CDCl_3$): δ = 7.69-7.66 (m, 2H; H^{Ar}), 7.54-7.34 (m, 11H; H^{Ar}), 2.39 (s, 3H; CH_3) ppm; **^{13}C NMR** (76 MHz, $CDCl_3$, APT): δ =

141.7 (C_q; C^{Ar}), 141.1 (C_q; C^{Ar}), 141.1 (C_q; C^{Ar}), 140.3 (C_q; C^{Ar}), 135.9 (C_q; C^{Ar}), 130.4 (C^{Ar}), 129.4 (C^{Ar}), 129.3 (C^{Ar}), 128.9 (C^{Ar}), 128.3 (C^{Ar}), 127.4 (C^{Ar}), 127.3 (C^{Ar}), 127.0 (C^{Ar}), 124.7 (C^{Ar}), 20.8 (CH_3) ppm; **GC-MS** (EI, 70 eV; MT_50_S): t_R = 8.16 min; *m/z* (%): 246 (100) [M^+].

5.2 3-(2-Methyl-[1,1'-biphenyl]-4-yl)pyridine

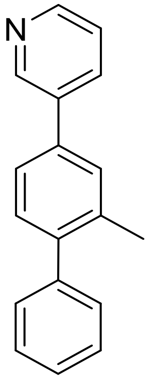

56

56 was prepared according to general procedure 2.4 from 0.24 mL 1-bromo-4-iodo-2-methylbenzene (1.68 mmol, 1.0 eq), 385 mg 3-pyridineboronic acid pinacolester (1.88 mmol, 1.2 eq), 469 mg K_2CO_3 (3.40 mmol, 2.0 eq) and 62 mg $PdCl_2$(dppf) (84.5 µmol, 5 mol%) in 8 mL degassed DMF. The brown crude product was purified via flash column chromatography (20 g SiO_2, 15 x 3 cm, eluent: cyclohexane/EtOAc = 3/1 → 1/2) to give the intermediate diaryl as a brown oil (411 mg, 98%).

The second coupling step was performed according to procedure 2.5 from 174 mg previously prepared intermediate (0.70 mmol, 1.0 eq), 95 mg phenylboronic acid (0.78 mmol, 1.1 eq), 466 mg Cs_2CO_3 (1.43 mmol, 2.0 eq) and 26 mg $PdCl_2$(dppf) (35.8 µmol, 5 mol%) in 3 mL abs., degassed DMF. The brown crude product was purified via flash column chromatography (10 g SiO_2, 15 x 2.5 cm, eluent: cyclohexane/EtOAc = 5/1 → 3/1. For the solubility studies, a small sample was additionally purified via semi-preparative HPLC (MV_NucleodurC18_001HCOOH_50to100) (t_R = 12.8 min) with 70% recovery.

Yield: 127 mg (74% over 2 steps), colorless solid, $C_{18}H_{15}N$ [245.33 g/mol].

TLC: R_f = 0.17 (cyclohexane/EtOAc = 5/1, UV and CAM); **^{1}H NMR** (300 MHz, $CDCl_3$): δ = 8.92 (s, 1H; H^{Ar}), 8.61 (m, 1H; H^{Ar}), 7.96 (d, $^3J_{H,H}$ = 7.8 Hz, 1H; H^{Ar}), 7.50-7.35 (m, 9H; 9 x H^{Ar}), 2.37 (s, 3H; CH_3) ppm; **^{13}C NMR** (76 MHz, $CDCl_3$, APT): δ = 147.9 (C^{Ar}), 147.8 (C^{Ar}), 142.1 (C_q; C^{Ar}), 141.3 (C_q; C^{Ar}), 136.7 (C_q; C^{Ar}), 136.5 (C_q; C^{Ar}), 136.4 (C_q; C^{Ar}), 134.9 (C^{Ar}), 130.7 (C^{Ar}), 129.2

(2x C^{Ar}), 129.2 (C^{Ar}), 128.3 (2x C^{Ar}), 127.2 (C^{Ar}), 124.6 (C^{Ar}), 123.8 (C^{Ar}), 20.7 (CH_3) ppm; **GC-MS** (EI, 70 EV; MT_50_S): t_R = 7.845 min, *m/z* (%) = 245 (100%) [M^+]; **HRMS** (MALDI): calcd (*m/z*) for [M^+]: 245.1205, found: 245.1204.

5.3 Gly-Ala-Gly

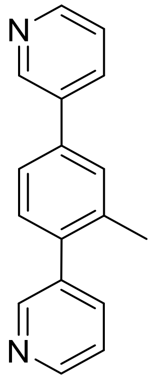

57

Compound **57** was prepared according to general procedure 2.5 from 0.24 mL 1-bromo-4-iodo-2-metthylbenzene (1.68 mmol, 1.0 eq), 760 mg 3-pyridineboronic acid pinacolester (3.71 mmol, 2.2 eq), 220 mg Cs_2CO_3 (6.75 mmol, 4.0 eq) and 64 mg $PdCl_2$(dppf) (87.9 µmol, 5 mol%) in 8 mL abs., degassed DMF. The dark-green crude product was purified via flash column chromatography (20 g SiO_2, 15 x 3 cm, eluent: cyclohexane/EtOAc = 1/3 → EtOAc). For the solubility studies, a small sample was additionally purified via semi-preparative HPLC (MV_NucleodurC18_001HCOOH_10to100) (t_R = 7.8 min) with 29% recovery.
Yield: 390 mg (94%), colorless solid, $C_{17}H_{14}N_2$ [246.31 g/mol].

TLC: R_f = 0.20 (cyclohexane/EtOAc = 1/4, UV and CAM); **m.p.**$^{exp.}$ = 83-85 °C; **^{1}H NMR** (300 MHz, $CDCl_3$): δ = 8.87 (s, 1H; H^{Ar}), 8.60 (m, 3H; 3x H^{Ar}), 7.91 (d, $^3J_{H,H}$ = 7.7 Hz, 1H; H^{Ar}), 7.68 (d, $^3J_{H,H}$ = 7.6 Hz, 1H; H^{Ar}), 7.54-7.43 (m, 2H; 2x H^{Ar}), 7.42-7.27 (m, 3H; 3x H^{Ar}), 2.33 (s, 3H; CH_3) ppm; **^{13}C NMR** (76 MHz, $CDCl_3$, APT): δ = 149.6 (C^{Ar}), 148.3 (C^{Ar}), 148.2 (C^{Ar}), 148.0 (C^{Ar}), 138.0 (C_q; C^{Ar}), 137.6 (C_q; C^{Ar}), 137.0 (C_q; C^{Ar}), 136.7 (C^{Ar}), 136.6 (C_q; C^{Ar}), 136.3 (C_q; C^{Ar}), 134.7 (C^{Ar}), 130.7 (C^{Ar}), 129.4 (C^{Ar}), 124.9 (C^{Ar}), 123.8 (C^{Ar}), 123.3 (C^{Ar}), 20.6 (CH_3) ppm**;** **GC-MS** (EI, 70 EV; MT_50_S): t_R = 8.141 min, *m/z* (%) = 246 (100) [M^+]; **HRMS** (MALDI): calcd *(m/z*) for [M^+]: 246.1157, found: 246.1158.

5.4 2-Bromo-5-iodobenzaldehyde

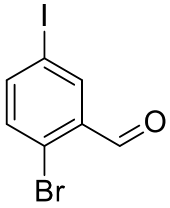

58a

In a 250 mL flame dried round-bottom flask equipped with a Schlenk adapter 2.40 g (7.67 mmol, 1.0 eq) (2-bromo-5-iodophenyl)-methanol were dissolved in 75 mL CH_2Cl_2. 1.5 g anhydrous 4Å MS and 4.0 g (46.0 mmol, 6.0 eq) MnO_2 were added under inert atmosphere. The black reaction mixture was stirred overnight. After full conversion of the starting material was indicated by TLC and GC-MS (15 h) MnO_2 was removed via filtration through a pad of silica (20 g SiO_2, eluent: EtOAc, fraction size: 50 mL). The filtrate was collected and the solvent was removed under reduced pressure. The crude product was used without further purification.

Yield: 2.14 g (6.90 mmol, 90%), colorless solid, C_7H_4BrIO [309.85 g/mol]

TLC: R_f = 0.67 (cyclohexane/EtOAc = 3/1, UV and CAM); **mp**$^{exp.}$ = 103-107 °C (**mp**$^{lit.}$ = 112 – 114 °C)[19]; **^{1}H NMR** (300 MHz, $CDCl_3$): δ = 10.24 (s, 1H; COH), 8.19 (d, $^4J_{H,H}$ = 2.1 Hz, 1H; H^{Ar}), 7.74 (dd, $^3J_{H,H}$ = 8.3, $^4J_{H,H}$ = 2.1 Hz, 1H; H^{Ar}), 7.38 (d, $^3J_{H,H}$ = 8.4 Hz, 1H; H^{Ar}) ppm; **^{13}C NMR** (75.53 MHz, $CDCl_3$): δ = 190.5 (CO), 144.0 (C^{Ar}), 138.8 (C_q; C^{Ar}), 135.6 (C^{Ar}), 134.9 (C^{Ar}), 126.7 (C_q; C^{Ar}), 93.0 (C_q; C^{Ar}) ppm; **GC-MS** (EI, 70 eV; MT_50_S): t_R = 5.83 min, *m/z* = 310 (100), 283 (12), 202 (6), 157 (8), 127 (10), 75 (34).

Analytical data are in accordance with those reported.[19]

5.4.1 Ethyl 3-(2-bromo-5-iodophenyl)acrylate

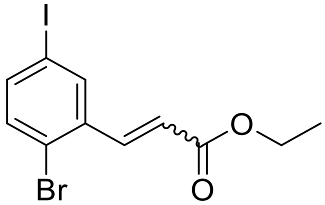

58b

In a flame dried Schlenk-flask 4.98 g (11.6 mmol, 1.2 eq) (2-ethoxy-2-oxoethyl)triphenylphosphonium bromide and 1.42 g (12.8 mmol, 1.3 eq) KO*t*Bu were dried in vacuum for 1 h. Then 40 mL abs., degassed THF were added. The yellowish suspension was stirred at RT for 1 h. Then 2.94 g (9.49 mmol, 1.0 eq) 2-bromo-5-iodobenzaldehyde (**58a**) were added to the ylide solution. The orange/yellow suspension was stirred overnight (16 h) at 50 °C until complete conversion was detected by TLC. The solvent was removed under reduced pressure. The yellow-brown crude product was purified via flash column chromatography (500 g SiO_2, 5.5 x 19.0 cm, eluent: cyclohexane/EtOAc = 75/1 → 50/1, fraction size: 50 mL).

Yield: 3.38 g (8.90 mmol, 94%), *E/Z* mixture = 4/1, colorless solid, $C_{11}H_{10}BrIO_2$ [379.89 g/mol].

TLC: R_f = 0.53 (cyclohexane/EtOAc = 10/1, UV and $KMnO_4$); **mp**$^{exp.}$ = 65-72 °C; **^{1}H NMR** (300 MHz, $CDCl_3$): δ = 7.92-7.87 (m, 2H; H^{Ar} & CH), 7.74 (d, $^4J_{H,H}$ = 1.2 Hz; 0.2H, H^{Ar}), 7.51 (dd, $^3J_{H,H}$ = 8.4 Hz, $^4J_{H,H}$ = 1.8 Hz; 1H, H^{Ar}), 7.46 (d, $^4J_{H,H}$ = 1.6 Hz, 0.1H; H^{Ar}), 7.32 (d, $^3J_{H,H}$ = 8.4 Hz, 1H; H^{Ar}), 7.27 (s, 0.1H; H^{Ar}), 6.96 (d, $^3J_{H,H}$ = 12.1 Hz, 0.2H; CH), 6.37 (d, $^3J_{H,H}$ = 15.9 Hz, 1H; CH), 6.07 (d, $^3J_{H,H}$ = 12.1 Hz, 0.2H; CH), 4.28 (q, $^3J_{H,H}$ = 7.1 Hz, 2H; CH_2), 4.12 (q, $^3J_{H,H}$ = 7.1 Hz, 0.5H; CH_2), 1.34 (t, $^3J_{H,H}$ = 7.1 Hz, 3H; CH_3), 1.19 (t, $^3J_{H,H}$ = 7.1 Hz, 0.7H; CH_3) ppm; **^{13}C-NMR** (75.53 MHz, $CDCl_3$): δ = 166.1 (C_q; CO), 141.5 (CH), 140.7 (CH), 139.9 (C^{Ar}), 139.4 (C^{Ar}), 138.6 (C^{Ar}), 136.9 (C_q: C^{Ar}), 136.7 (C^{Ar}), 135.0 (C^{Ar}), 133.9 (C^{Ar}), 125.0 (C_q, C^{Ar}), 123.0 (CH), 122.5 (CH), 92.7 (C_q; C^{Ar}), 91.5 (C_q; C^{Ar}), 61.0 (CH_2), 60.7 (CH_2), 14.4 (CH_3), 14.2 (CH_3) ppm; **HRMS** (DI-EI): calcd *(m/z)* for [M^+]: 379.8909; found: 379.8921.

5.4.2 Ethyl 3-(2-bromo-5-iodophenyl)propanoate

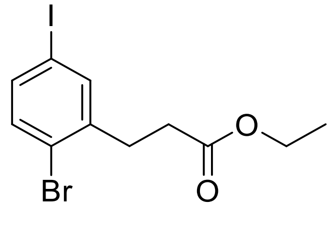

58c

A 250 mL one-neck round-bottom flask, equipped with reflux-condenser, was charged with 3.30 g (8.69 mmol, 1.0 eq) ethyl 3-(2-bromo-5-iodophenyl)acrylate (**58b**) which was then dissolved in 70 mL THF. First 9.71 g (52.1 mmol, 6.0 eq) p-tosylhydrazide and afterwards 7.10 g (52.1 mmol, 6.0 eq) NaOAc·3H_2O were added and the pale yellow suspension was stirred at 70 °C until quantitative

conversion (50 h) was detected by GC-MS. The reaction mixture was cooled to RT and 75 mL satd. $NaHCO_3$-solution were added. The phases were separated and the aqueous layer was extracted with CH_2Cl_2 (3 x 100 mL). The combined organic layers were dried over Na_2SO_4, filtered and the solvent was removed under reduced pressure. The crude product was purified via flash column chromatography (500 g SiO_2, 5.5 x 19.0 cm, eluent: cyclohexane/EtOAc = 75/1).

Yield: 3.05 g (7.99 mmol, 92%), colorless solid, $C_{11}H_{12}BrIO_2$ [381.91 g/mol].

TLC: R_f = 0.17 (cyclohexane/EtOAc = 75/1, UV and $KMnO_4$); **mp**$^{exp.}$ = 42-46 °C; **^{1}H NMR** (300 MHz, $CDCl_3$): δ = 7.58 (d, $^4J_{H,H}$ = 1.6 Hz, 1H; H^{Ar}), 7.38 (dd, $^3J_{H,H}$ = 8.3 Hz, $^4J_{H,H}$ = 1.8 Hz, 1H; H^{Ar}), 7.25 (d, $^3J_{H,H}$ = 8.5 Hz, 1H; H^{Ar}), 4.15 (q, $^3J_{H,H}$ = 7.1 Hz, 2H; CH_2), 3.00 (t, $^3J_{H,H}$ = 7.7 Hz, 2H; CH_2), 2.62 (t, $^3J_{H,H}$ = 7.7 Hz, 2H; CH_2), 1.25 (t, $^3J_{H,H}$ = 7.1 Hz, 3H; CH_3) ppm; **^{13}C NMR** (75.53 MHz, $CDCl_3$, APT): δ = 172.4 (C_q: CO), 142.3 (C_q; C^{Ar}), 139.4 (C^{Ar}), 137.2 (C^{Ar}), 134.6 (C^{Ar}), 124.4 (C_q; C^{Ar}), 92.6 (C_q; C^{Ar}), 60.8 (CH_2), 34.0 (CH_2), 31.2 (CH_2), 14.4 (CH_3) ppm; **HRMS** (DI-EI): calcd *(m/z)* for [M^+]: 381.9065; found: 381.8986.

5.5 3-(3,3''-Diisobutyl-[1,1':4',1''-terphenyl]-2'-yl)propanoic acid

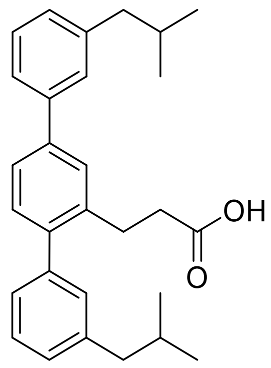

58

Compound **58** was prepared according to general procedure 2.5 from 206 mg ethyl 3-(2-bromo-5-iodophenyl)propanoate (**58c**) (0.54 mmol, 1.0 eq), 298 mg 3-isobutylbenzeneboronic acid pinacol ester[17] (1.15 mmol, 2.1 eq), 685 mg Cs_2CO_3 (2.10 mmol, 3.9 eq) and 20.5 mg $PdCl_2$(dppf) (28.0 µmol, 5 mol%) in 3 mL abs., degassed DMF. The orange crude product was purified via flash column chromatography (13 g SiO_2, 30 x 1.5 cm, eluent: cyclohexane/EtOAc = 100/1 → 50:1).
For the deprotection step, the previously prepared intermediate was dissolved in 4 mL THF and 211 mg $LiOH\cdot H_2O$ (5.03 mmol, 9.3 eq) in 2 mL H_2O were added. The reaction mixture was stirred for 21 h and full conversion was detected via TLC. 5 mL 1M HCl and 10 mL H_2O were added and

the reaction mixture was transferred to a separatory funnel. The aqueous phase was extracted with DCM (3 x 10 mL) and the combined organic phases were dried over Na_2SO_4 and filtered. The filter cake was rinsed with DCM and the solvent was removed under reduced pressure. For the solubility studies, a small amount of the product was additionally purified via semi-preparative HPLC (MV_NucleodurC18_001HCOOH_80to100) (t_R = 12 min) with 28% recovery.
Yield: 120 mg (54% over 2 steps), colorless solid, $C_{29}H_{34}O_2$ [414.16 g/mol].

TLC: R_f = 0.17 (cyclohexane/EtOAc = 10/1 + 1% AcOH, UV and $KMnO_4$); **^{1}H NMR** (300 MHz, $CDCl_3$): δ = 8.96 (bs, 1H; COOH), 7.56-7.28 (m, 7H; 7x H_{Ar}), 7.21-7.09 (m, 4H; 4x H_{Ar}), 3.03 (t, $^3J_{H,H}$ = 7.0 Hz, 2H; CH_2), 2.61-2.40 (m, 6H; 3x CH_2), 2.03-1.84 (m, 2H; 2x CH), 1.03-0.85 (m, 12H, 4x CH_3) ppm; **^{13}C NMR** (76 MHz, $CDCl_3$, APT): δ = 179.0 (C_q; CO), 142.4 (C_q; C^{Ar}), 141.8 (C_q; C^{Ar}), 141.3 (C_q; C^{Ar}), 140.9 (C_q; C^{Ar}), 140.8 (C_q; C^{Ar}), 140.7 (C_q; C^{Ar}), 138.1 (C_q; C^{Ar}), 130.9 (C^{Ar}), 130.0 (C^{Ar}), 128.7 (C^{Ar}), 128.3 (C^{Ar}), 128.2 (C^{Ar}), 128.1 (2x C^{Ar}), 128.0 (C^{Ar}), 126.5 (C^{Ar}), 125.3 (C^{Ar}), 124.6 (C^{Ar}), 45.7 (CH_2), 45.6 (CH_2), 35.2 (CH_2), 30.4 (2x CH), 28.5 (CH_2), 22.6 (2x CH_3), 22.5 (2x CH_3) ppm; **HRMS** (MALDI): calcd (*m/z*) for [M^+]: 414.2559, found: 414.2558.

5.6 3-(3'-Isobutyl-4-(5-isobutylpyridin-3-yl)-[1,1'-biphenyl]-2-yl)propanoic acid

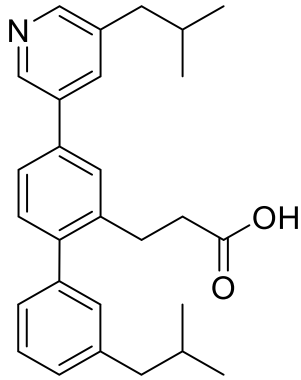

59

Compound **59** was prepared according to general procedure 2.4 from 206 mg ethyl 3-(2-bromo-5-iodophenyl)propanoate (**58c**) (0.54 mmol, 1.0 eq), 152 mg pyridine building block **39** (0.58 mmol, 1.1 eq), 145 mg K_2CO_3 (1.05 mmol, 2.0 eq) and 22.3 mg $PdCl_2$(dppf) (30.5 µmol, 5 mol%) in 3 mL abs., degassed DMF. The brown crude product was purified via flash column chromatography (10 g SiO_2, 11 x 2 cm, eluent: cyclohexane/EtOAc = 10/1 → 2/1).
The second coupling step was performed according to general procedure 2.5 from 100 mg 3-isobutylbenzeneboronic acid pinacol ester[17] (0.39 mmol, 1.1 eq), 282 mg Cs_2CO_3 (0.87 mmol,

2.4 eq), 18.2 mg $PdCl_2$(dppf) (24.9 µmol, 7 mol%) and 139 mg previously prepared intermediate (0.36 mmol, 1.0 eq) in 3 mL abs., degassed DMF. The crude product was purified via flash column chromatography (10 g SiO_2, 15 x 2.5 cm, eluent: cyclohexane/EtOAc = 5/1).

In the last deprotection step 110 mg previously prepared intermediate (0.25 mmol, 1.0 eq) were dissolved in 4 mL THF. 109 mg $LiOH \cdot H_2O$ (2.61 mmol, 10.5 eq) in 2 mL H_2O were added and the reaction mixture was stirred for 21 h. Full conversion was detected via TLC. 2.5 mL 1M HCl and 10 mL H_2O were added and the reaction mixture was transferred to a separatory funnel. The aqueous phase was extracted with DCM (5 x 10 mL) and the combined organic phases were dried over Na_2SO_4 and filtered. The filter cake was rinsed with DCM and the solvent was removed under reduced pressure. For the solubility studies, a small amount of the product was additionally purified via semi-preparative HPLC (MV_NucleodurC18_001HCOOH_70to100) (t_R = 12.0 min) with 36% recovery.

Yield: 105 mg (46% over 3 steps), colorless solid, $C_{28}H_{33}NO_2$ [415.63 g/mol].

TLC: R_f = 0.22 (cyclohexane/EtOAc = 2/1 + 1% AcOH, UV and CAM); **m.p.**$^{exp.}$ = 50 °C; **^{1}H NMR** (300 MHz $CDCl_3$): δ = 9.39 (bs, 1H; COOH), 8.79 (s, 1H; H^{Ar}), 8.41 (s, 1H; H^{Ar}), 7.94 (s, 1H; H^{Py}), 7.59 (s, 1H; H^{Ar}), 7.49-7.43 (m, 1H; H^{Ar}), 7.37-7.30 (m, 2H; 2x H^{Ar}), 7.19 -7.09 (m, 3H; 3x H^{Ar}), 3.05 (t, $^3J_{H,H}$ = 7.4 Hz, 2H; CH_2), 2.62 (d, $^3J_{H,H}$ = 7.0 Hz, 2H; CH_2), 2.56-2.47 (m, 4H; 2x CH_2), 1.99-1.84 (m, 2H; 2x CH), 0.98-0.88 (m, 12H; 4x CH_3) ppm; **^{13}C NMR** (76 MHz, $CDCl_3$, APT): δ = 176.4 (C_q; CO), 144.8 (C^{Ar}), 143.2 (C_q; C^{Ar}), 142.1 (C^{Ar}), 142.0 (C_q; C^{Ar}), 140.4 (C_q; C^{Ar}), 139.6 (C_q; C^{Ar}), 138.9 (C_q; C^{Ar}), 138.4 (C^{Ar}), 137.8 (C_q; C^{Ar}), 135.3 (C_q; C^{Ar}), 131.4 (C^{Ar}), 129.9 (C^{Ar}), 128.4 (C^{Ar}), 128.3 (C^{Ar}), 128.2 (C^{Ar}), 126.4 (C^{Ar}), 125.0 (C^{Ar}), 45.6 (CH_2), 42.4 (CH_2), 35.3 (CH_2), 30.4 (CH), 30.1 (CH), 28.6 (CH_2), 22.5 (2x CH_3), 22.3 (2x CH_3) ppm; **HRMS** (MALDI): calcd (*m/z*) for [M^+]: 415.2511, found: 415.2509.

5.7 Leu-Glu-Leu

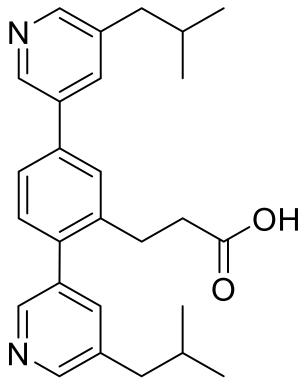

60

Compound **60** was prepared according to general procedure 2.5 from 206 mg ethyl 3-(2-bromo-5-iodophenyl)propanoate (**58c**) (0.54 mmol, 1.0 eq), 306 mg pyridine building block **39** (1.17 mmol, 2.2 eq), 684 mg Cs_2CO_3 (2.1 mmol, 3.9 eq) and 21.9 mg $PdCl_2$(dppf) (29.9 µmol, 5 mol%) in 3 mL abs., degassed DMF. The brown crude product was purified via flash column chromatography (13 g SiO_2, 11 x 3 cm, eluent: cyclohexane/EtOAc = 1/1 → 1/2).

For the deprotection step the previously prepared intermediate was dissolved in 4 mL THF and 150 mg $LiOH{\cdot}H_2O$ (3.57 mmol, 6.6 eq) in 2 mL H_2O were added. The reaction mixture was stirred for 21 h and full conversion was detected via TLC. 3.5 mL 1M HCl and 10 mL H_2O were added and the reaction mixture was transferred to a separatory funnel. The aqueous phase was extracted with DCM (5 x 10 mL) and the combined organic phases were dried over Na_2SO_4 and filtered. The filter cake was rinsed with DCM and the solvent was removed under reduced pressure. For the solubility studies, a small amount of the product was additionally purified via semi-preparative HPLC (MV_NucleodurC18_001HCOOH_30to100) (t_R = 13.2 min) with 52% recovery.

Yield: 168 mg (75% over 2 steps), colorless solid, $C_{27}H_{32}N_2O_2$ [416.57 g/mol].

TLC: R_f = 0.17 (cyclohexane/EtOAc = 1/3 + 1% AcOH, UV and CAM); **m.p.**$^{exp.}$ = 55 °C; **^{1}H NMR** (300 MHz, $CDCl_3$): δ = 11.78 (bs, 1H; COOH), 8.66 (s, 1H; H^{Ar}), 8.51-8.25 (m, 3H; 3x H^{Ar}), 7.75 (s, 1H; H^{Ar}), 7.62-7.51 (m, 2H; 2x H^{Ar}), 7.44 (d, $^3J_{H,H}$ = 6.8 Hz, 1H; H^{Ar}), 7.26-7.21 (m, 1H; H^{Ar}), 2.97 (t, $^3J_{H,H}$ = 7.3 Hz, 2H; CH_2), 2.59-2.44 (m, 6H; 3x CH_2), 1.95-1.80 (m, 2H; 2x CH), 0.93-0.82 (m, 12H; 4x CH_3) ppm; **^{13}C NMR** (76 MHz, $CDCl_3$, APT): δ = 175.3 (C_q; CO), 147.5 (C^{Ar}), 147.3 (C^{Ar}), 145.6 (C^{Ar}), 143.9 (C^{Ar}), 140.0 (C_q; C^{Ar}), 138.8 (C^{Ar}), 137.9 (C_q; 2x C^{Ar}), 137.5 (C_q; C^{Ar}), 137.3 (C_q; C^{Ar}), 136.9 (C_q; C^{Ar}), 136.7 (C^{Ar}), 136.5 (C_q; C^{Ar}), 131.2 (C^{Ar}), 128.2 (C^{Ar}),

125.3 (C^{Ar}), 42.4 (CH_2), 42.3 (CH_2), 35.7 (CH_2), 30.1 (2x CH), 28.4 (CH_2), 22.3 (4x CH_3)*;* **HRMS** (MALDI): calcd (*m/z*) for [M^+]: 416.2464, found: 416.2463.

5.8 Representative procedure for water solubility tests[20]

The kinetic solubility of teraryls was measured by HPLC using individual calibration curves. For the preparation of the solubility samples and the calibration standards a DMSO stock solution for each compound was prepared. For this, 10 µmol of the corresponding teraryl were dissolved in 1 mL DMSO (10 mM stock solution). In the next step 10 µL of the 10 mM DMSO stock solutions were added to 990 µL of PBS buffer (pH 7.4). The created 100 µM stock solutions were mixed for 5 s on a Grant-bio vortex mixer and agitated for 120 min at RT (500 rpm, Bohdan MiniBlock shaker). Then the solutions were centrifuged for 15 min at 14000 rpm with an Eppendorf 5415C centrifuge. 200 µL were decanted from the top of the 100 µM stock solutions, added to 50 µL DMSO and vortexed for 5 s to obtain the solubility samples. The transfer of the centrifuged stock solution into a vial with DMSO was necessary to avoid precipitation from the saturated solution.
The 100 µM calibration standards were produced by mixing 10 µL of the 10 mM DMSO stock solution with 990 µL DMSO for 5 s on the vortex mixer. The individual calibration curves were prepared by injecting 0.5, 2.5 and 5 µL of the calibration standards and the measured areas under the curves were used to calculate a linear function. At last, the obtained areas of the solubility samples by injecting 5 or 50 µL were inserted in the linear equation to determine the kinetic solubility values. All solubility samples and calibration standards were measured using the general gradient SOL_10_100 (described in section 1). Each volume of the calibration standards and solubility samples was injected and measured three times.

6 Literature:

[1] G. R. Fulmer, A. J. M. Miller, N. H. Sherden, H. E. Gottlieb, A. Nudelman, B. M. Stoltz, J. E. Bercaw, K. I. Goldberg, *Organometallics* **2010**, *29*, 2176–2179.

[2] W. G. Kofron, L. M. Baclawski, *J. Org. Chem.* **1976**, *41*, 1879–1880.

[3] S. C. Watson, J. F. Eastham, *J. Organomet. Chem.* **1967**, *9*, 165–168.

[4] A. Krasovskiy, P. Knochel, *Synthesis* **2006**, *2006*, 890–891.

[5] M. Peters, M. Trobe, R. Breinbauer, *Chem. Eur. J.* **2013**, *19*, 2450–2456.

[6] J. Zhang, C. Huitema, C. Niu, J. Yin, M. N. G. James, L. D. Eltis, J. C. Vederas, *Bioorg. Chem.* **2008**, *36*, 229–240.

[7] D. Wang, Y. Wang, J. Zhao, M. Shen, J. Hu, Z. Liu, L. Li, F. Xue, P. Yu, *Org. Lett.* **2017**, *19*, 984–987.

[8] T. Kauffmann, H. Fischer, *Chem. Ber.* **1973**, *106*, 220–227.

[9] M. Yamashita, K. Shimizu, Y. Koizumi, T. Wakimoto, Y. Hamashima, T. Asakawa, M. Inai, T. Kan, *Synlett* **2016**, *27*, 2734–2736.

[10] N. T. T. Chau, M. Meyer, S. Komagawa, F. Chevallier, Y. Fort, M. Uchiyama, F. Mongin, P. C. Gros, *Chem. Eur. J.* **2010**, *16*, 12425–12433.

[11] K. C. Lee, B. S. Moon, J. H. Lee, K.-H. Chung, J. A. Katzenellenbogen, D. Y. Chi, *Bioorg. Med. Chem.* **2003**, *11*, 3649–3658.

[12] H. Yuan, R. B. Silverman, *Bioorg. Med. Chem.* **2006**, *14*, 1331–1338.

[13] M. C. Caserio, R. E. Pratt, R. J. Holland, *J. Am. Chem. Soc.* **1966**, *88*, 5747–5753.

[14] O. Tsuge, S. Kanemasa, T. Naritomi, J. Tanaka, *Bull. Chem. Soc. Jpn.* **1987**, *60*, 1497–1504.

[15] R. Heim, S. Lucas, C. M. Grombein, C. Ries, K. E. Schewe, M. Negri, U. Müller-Vieira, B. Birk, R. W. Hartmann, *J. Med. Chem.* **2008**, *51*, 5064–5074.

[16] C. Weizmann, M. Sulzbacher, E. Bergmann, *J. Am. Chem. Soc.* **1948**, *70*, 1153–1158.

[17] M. Peters, M. Trobe, H. Tan, R. Kleineweischede, R. Breinbauer, *Chem. Eur. J.* **2013**, *19*, 2442–2449.

[18] M. Trobe, R. Breinbauer, *Monatsh. Chem.* **2016**, *147*, 509–521.

[19] S. Luliński, J. Serwatowski, M. Szczerbińska, *Eur. J. Org. Chem.* **2008**, *2008*, 1797–1801.

[20] C. Saal, A. C. Petereit, *Eur. J. Pharm. Sci.* **2012**, *47*, 589–595.

7 NMR Spectra

3-Bromo-5-isopropylpyridine **(4)**

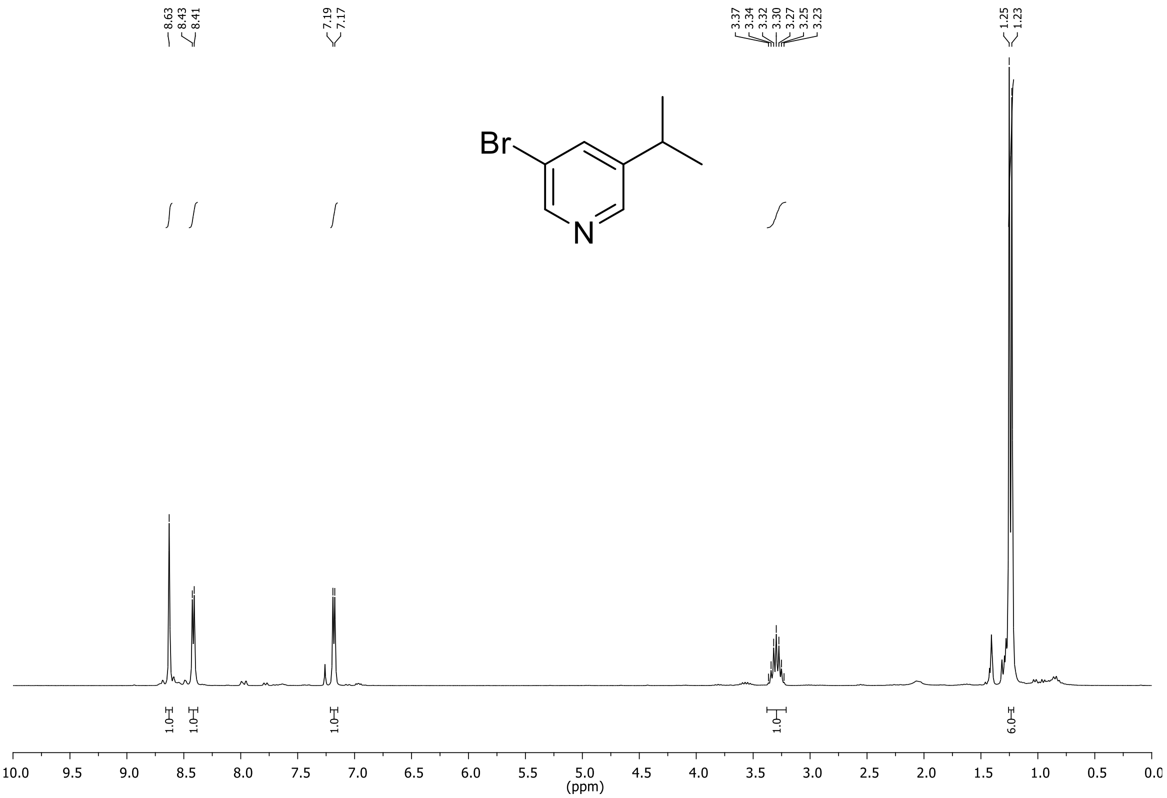

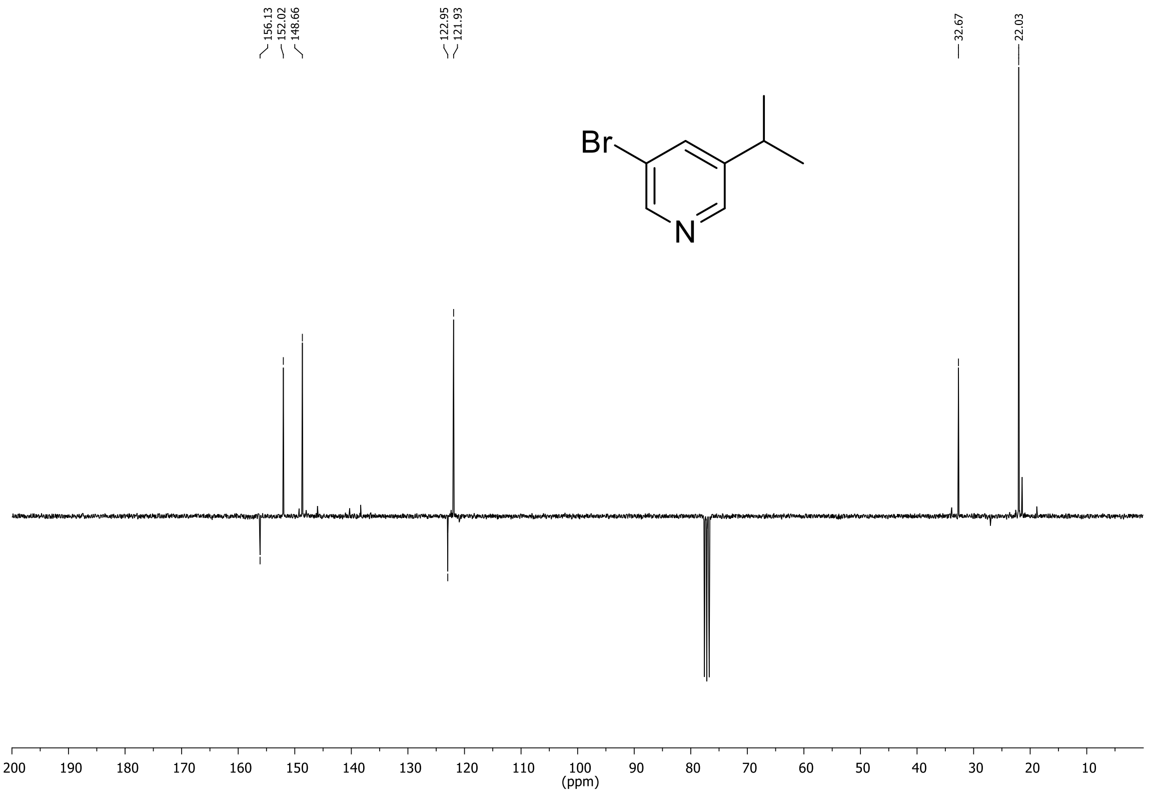

3-Isopropyl-5-(4,4,5,5-tetramethyl-1,3,2-dioxaborolan-2-yl)pyridine (**7**)

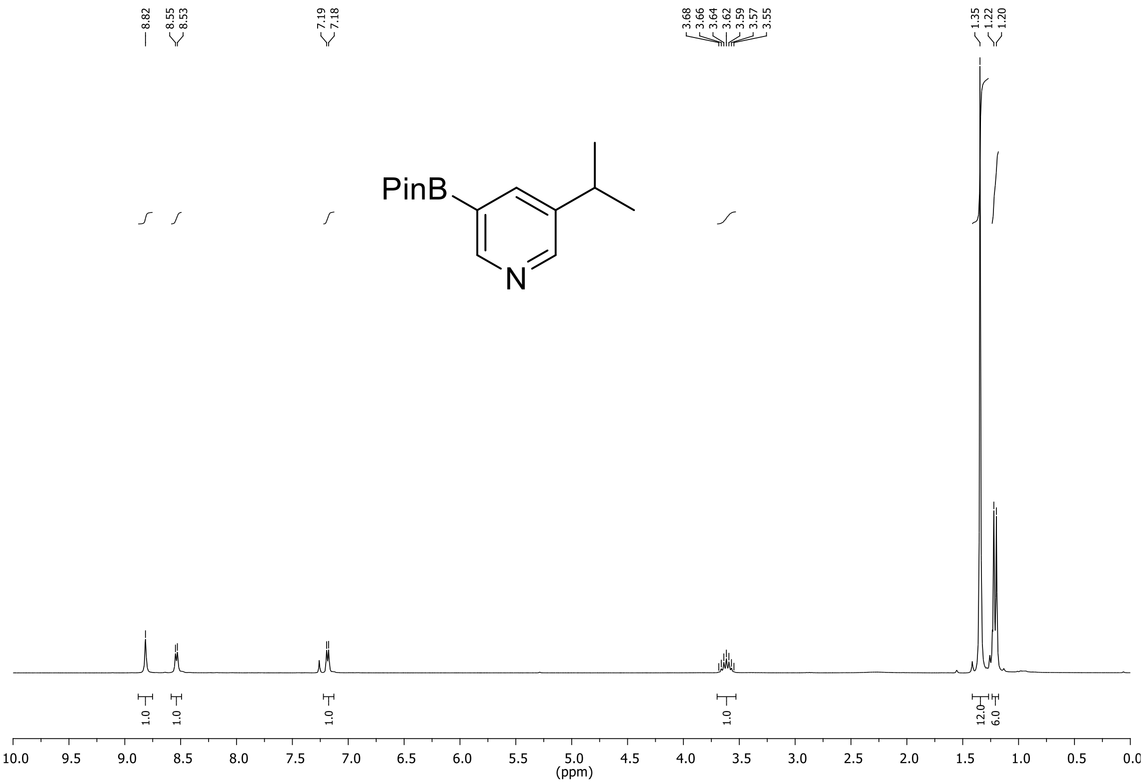

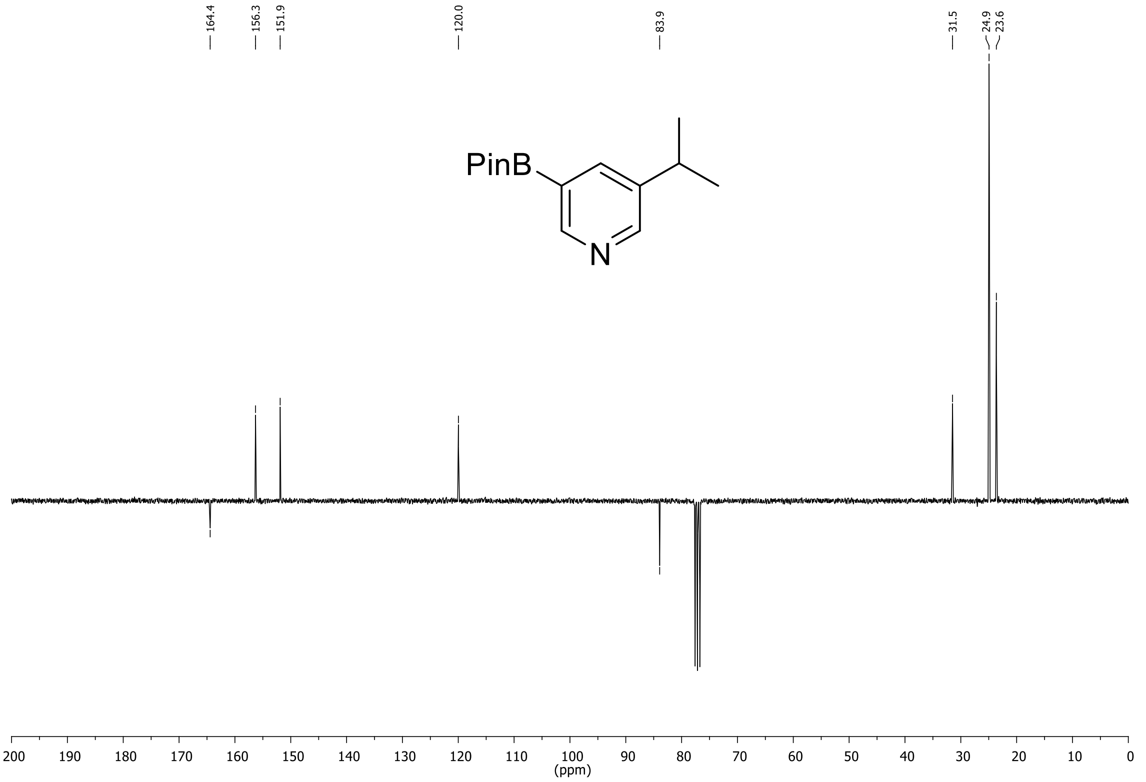

3-Bromo-5-(*sec*-butyl)pyridine (**5**)

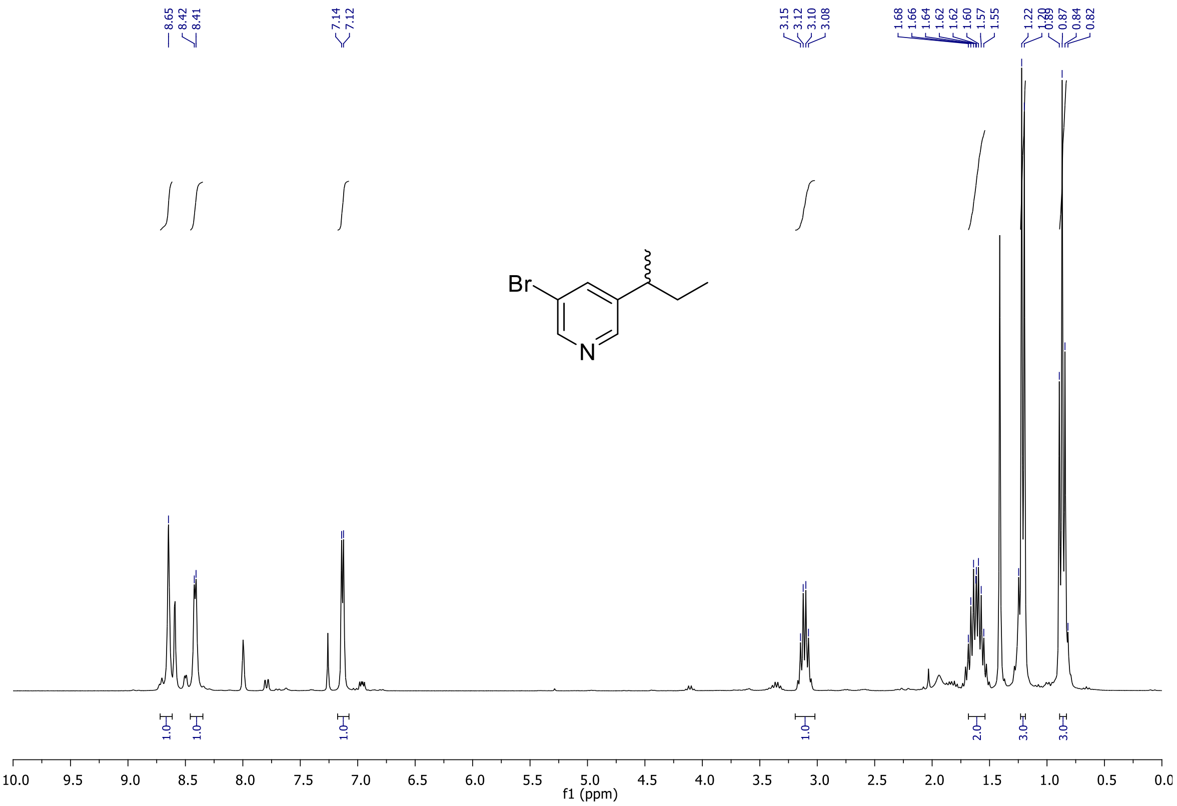

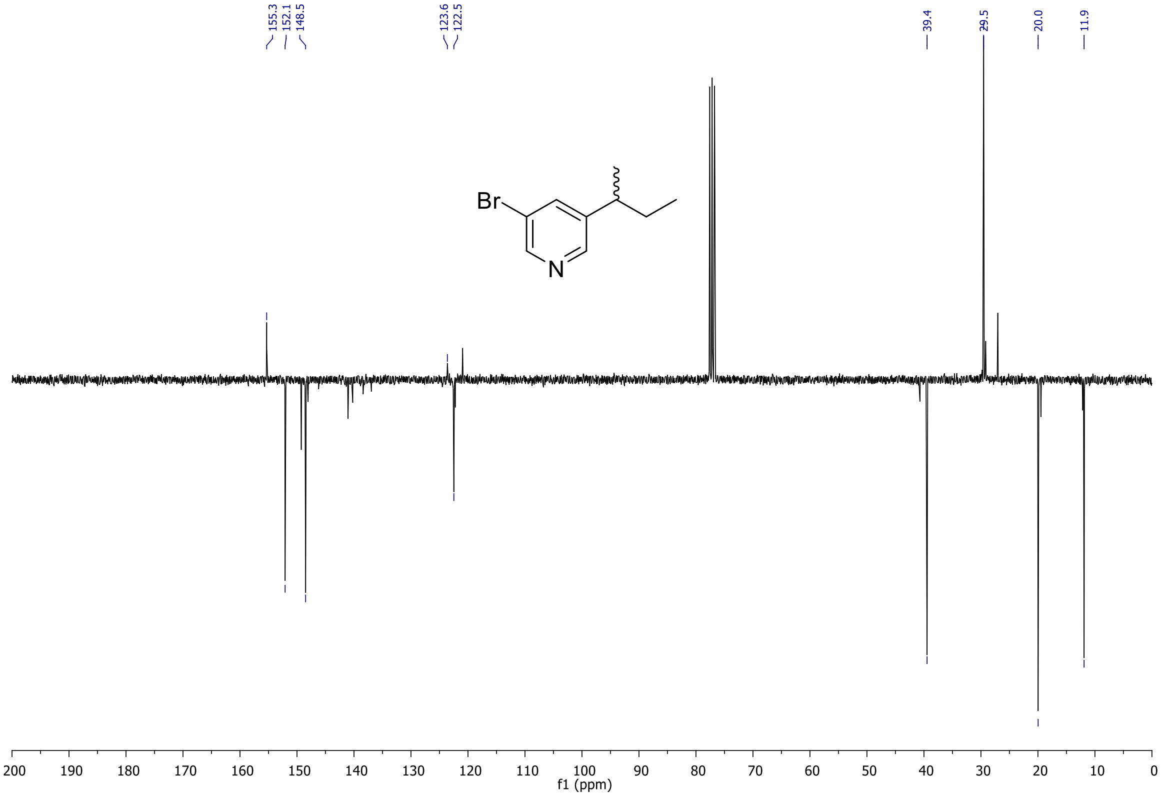

3-(*sec*-Butyl)-5-(4,4,5,5-tetramethyl-1,3,2-dioxaborolan-2-yl)pyridine (**8**)

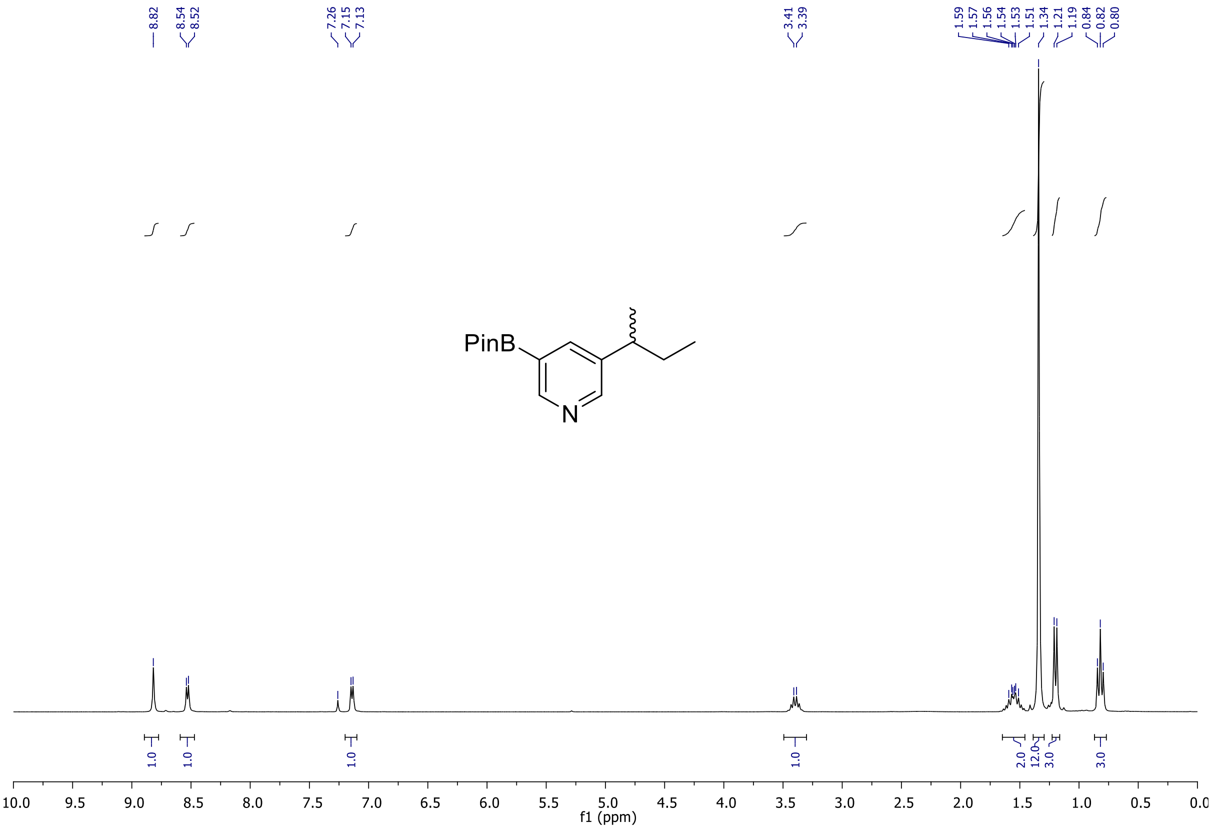

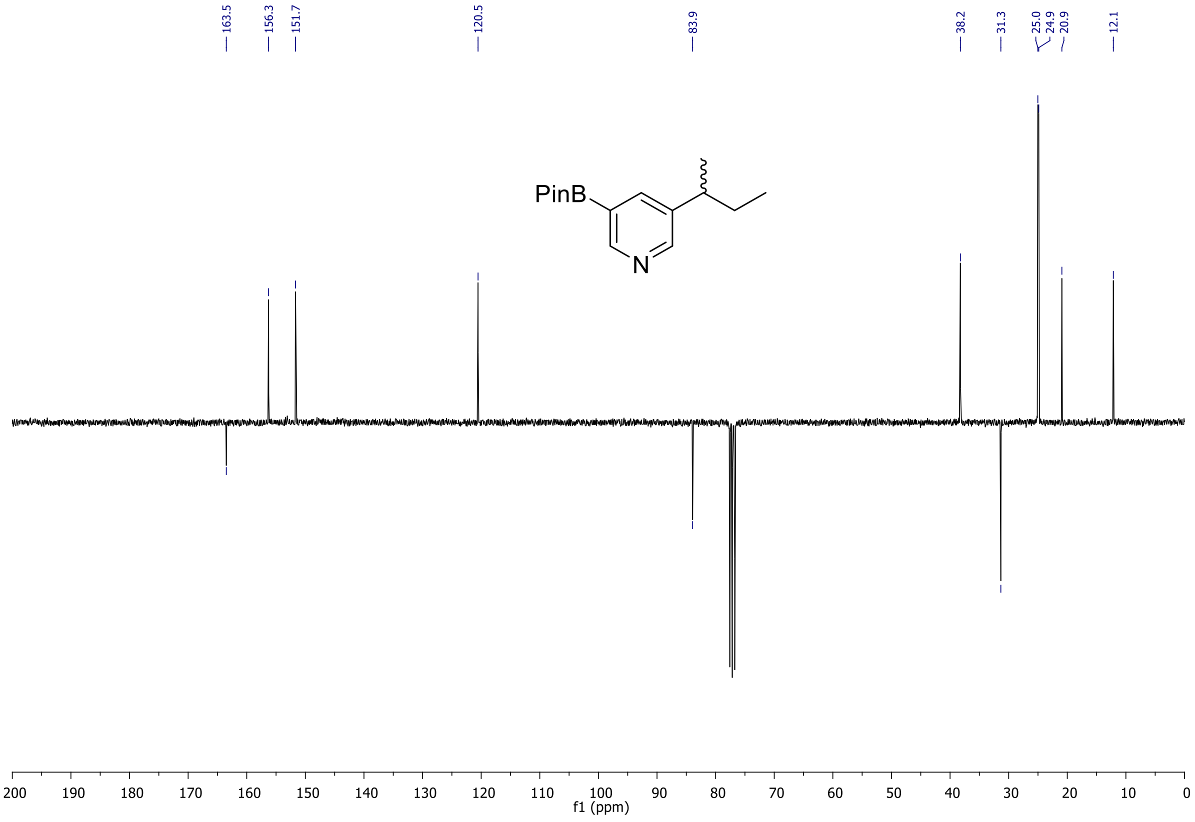

3-Benzyl-5-bromopyridine (**6**)

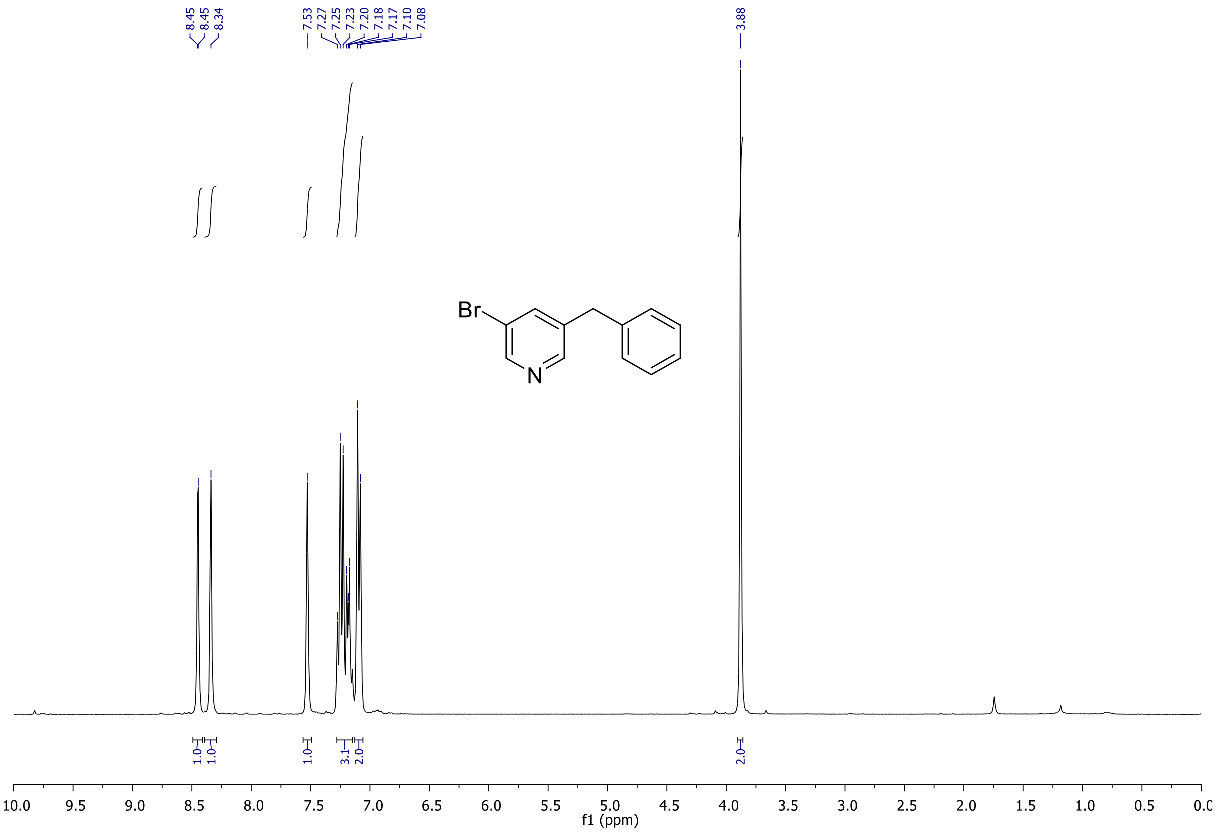

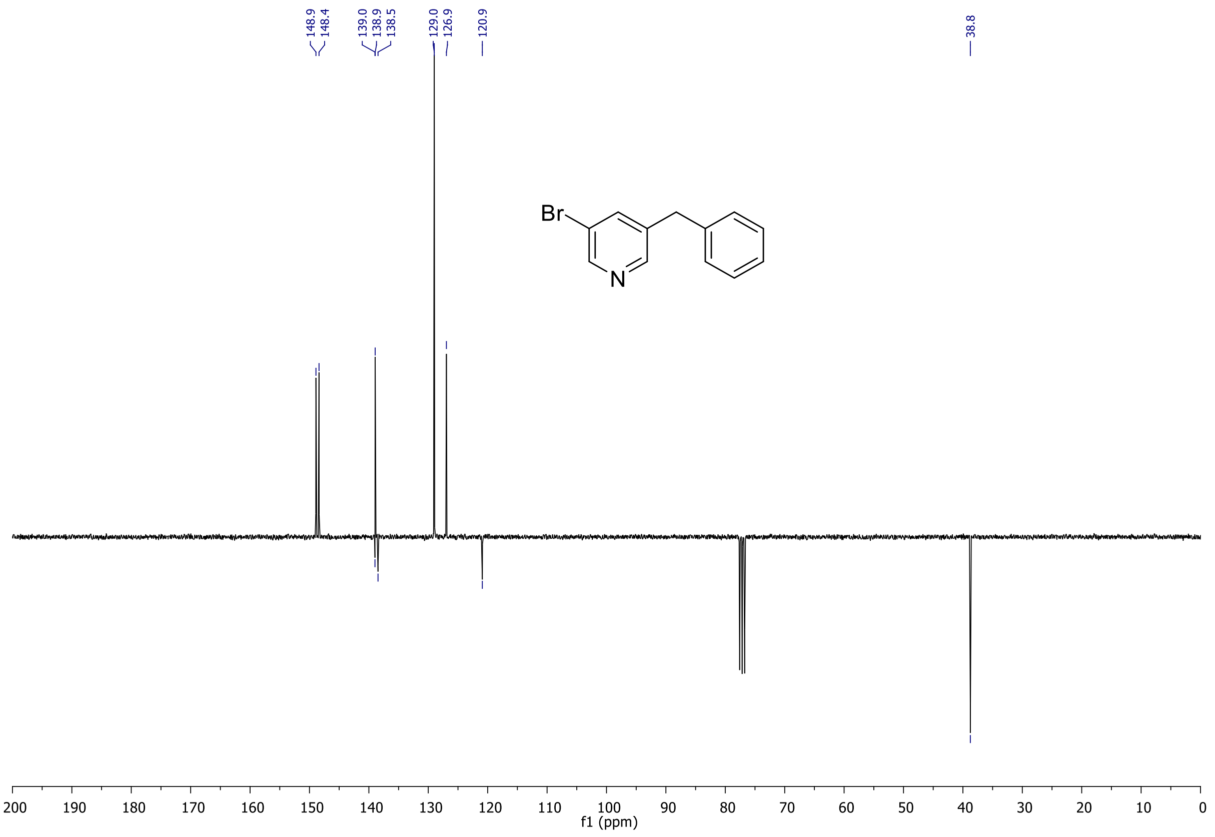

3-Benzyl-5-(4,4,5,5-tetramethyl-1,3,2-dioxaborolan-2-yl)pyridine (**9**)

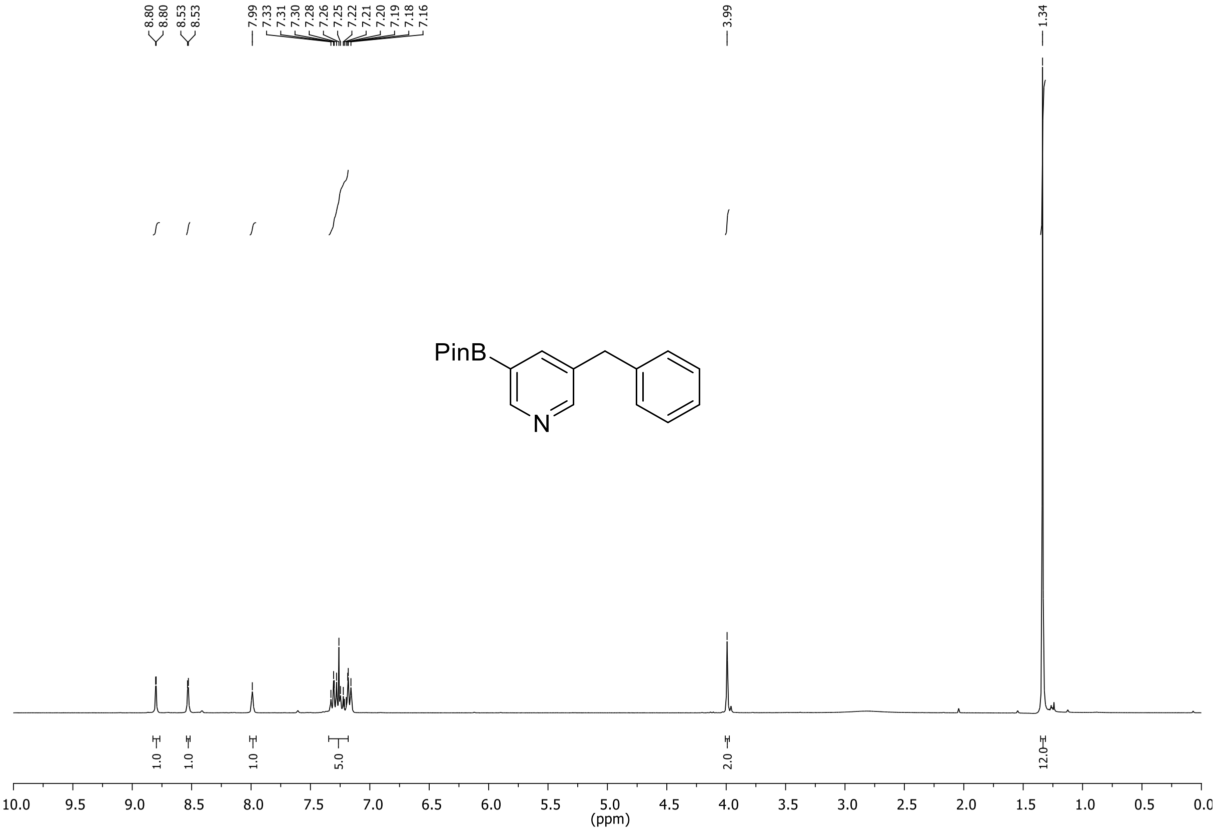

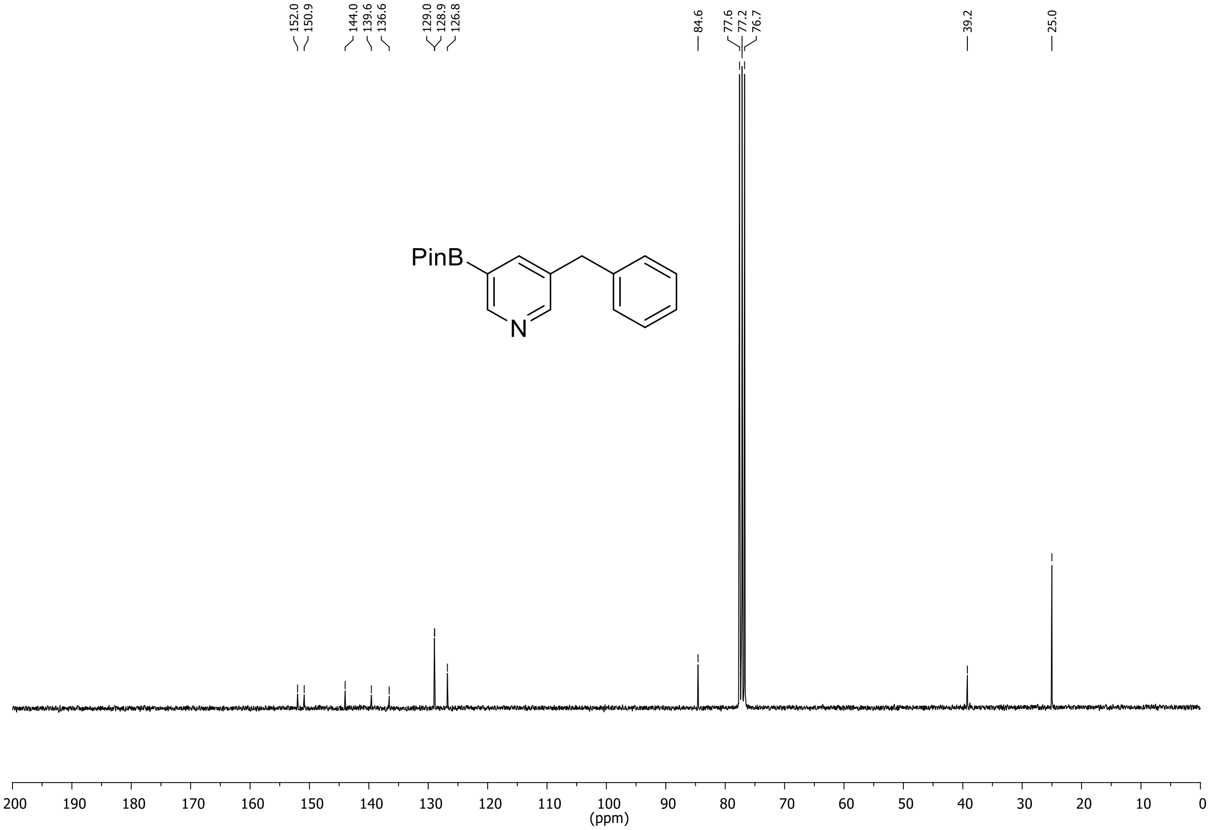

Methyl 2-(5-bromopyridin-3-yl)acetate (**10**)

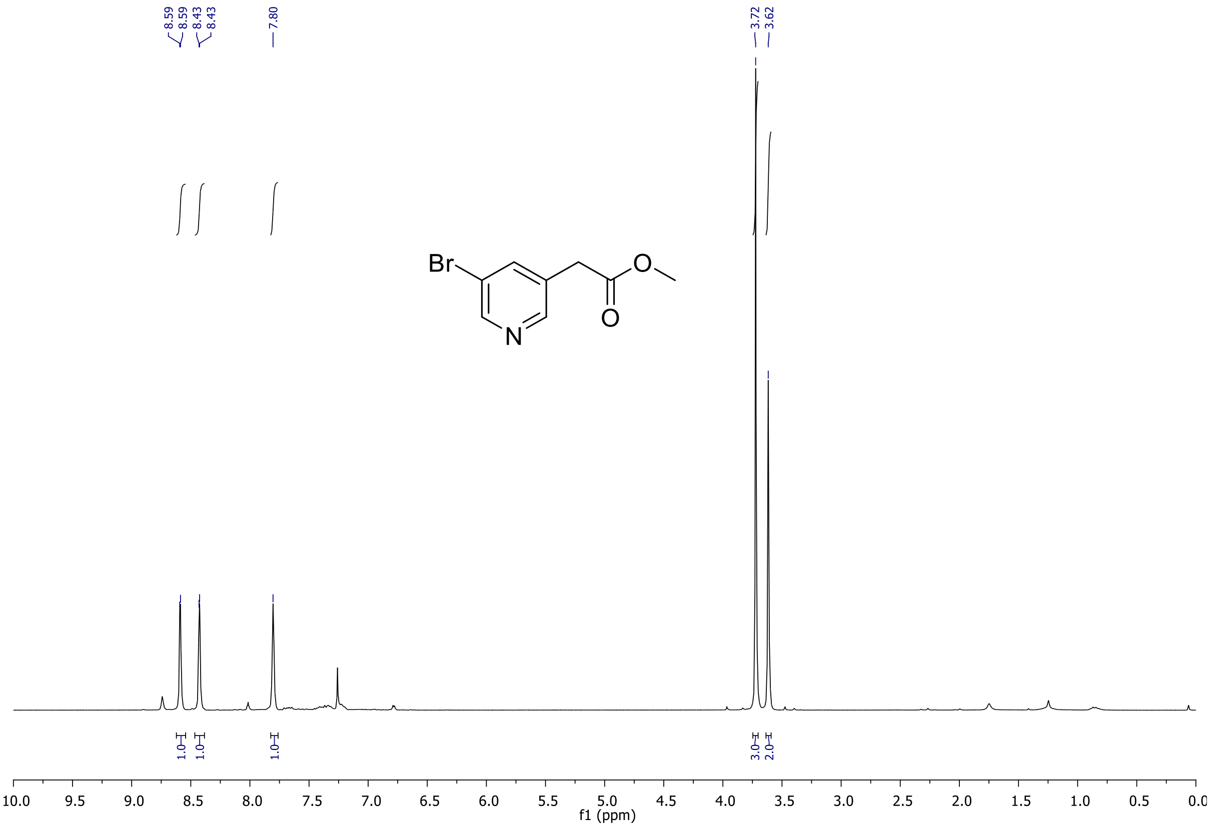

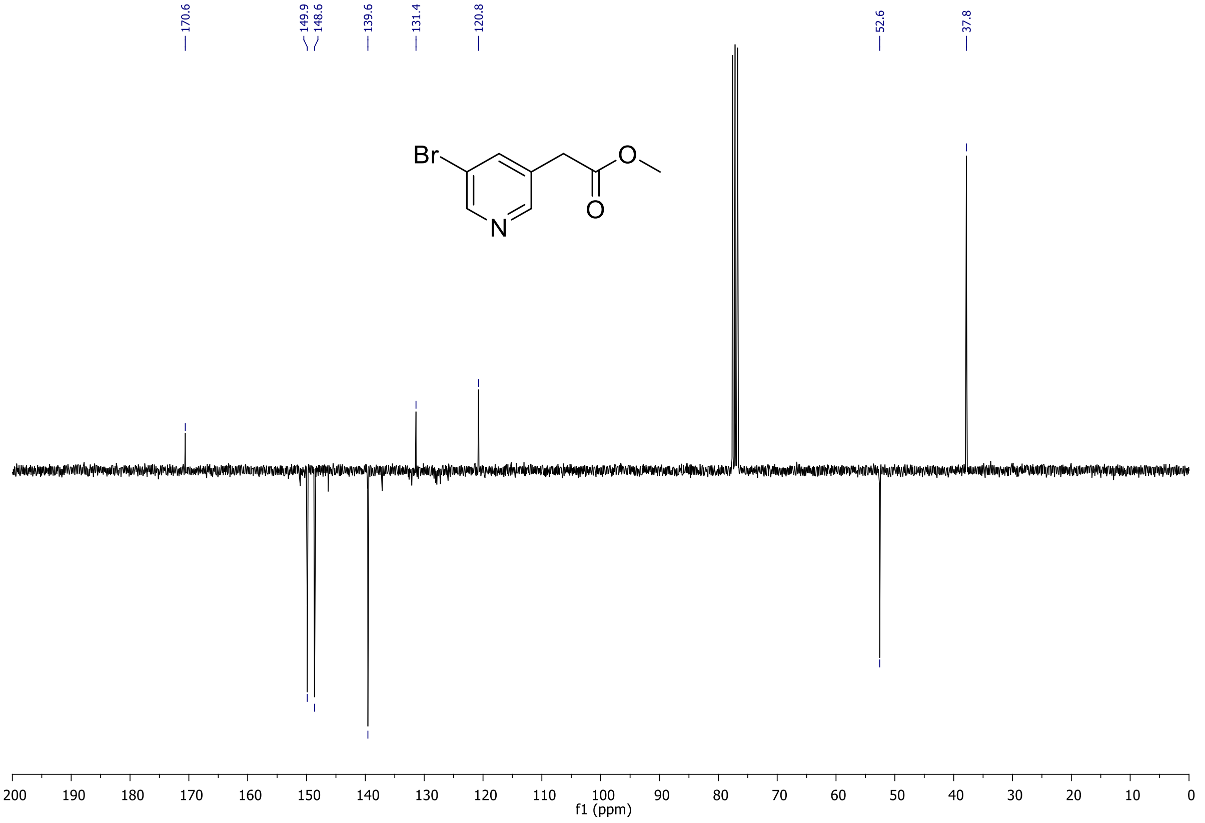

Methyl 2-(5-iodopyridin-3-yl)acetate (**11a**)

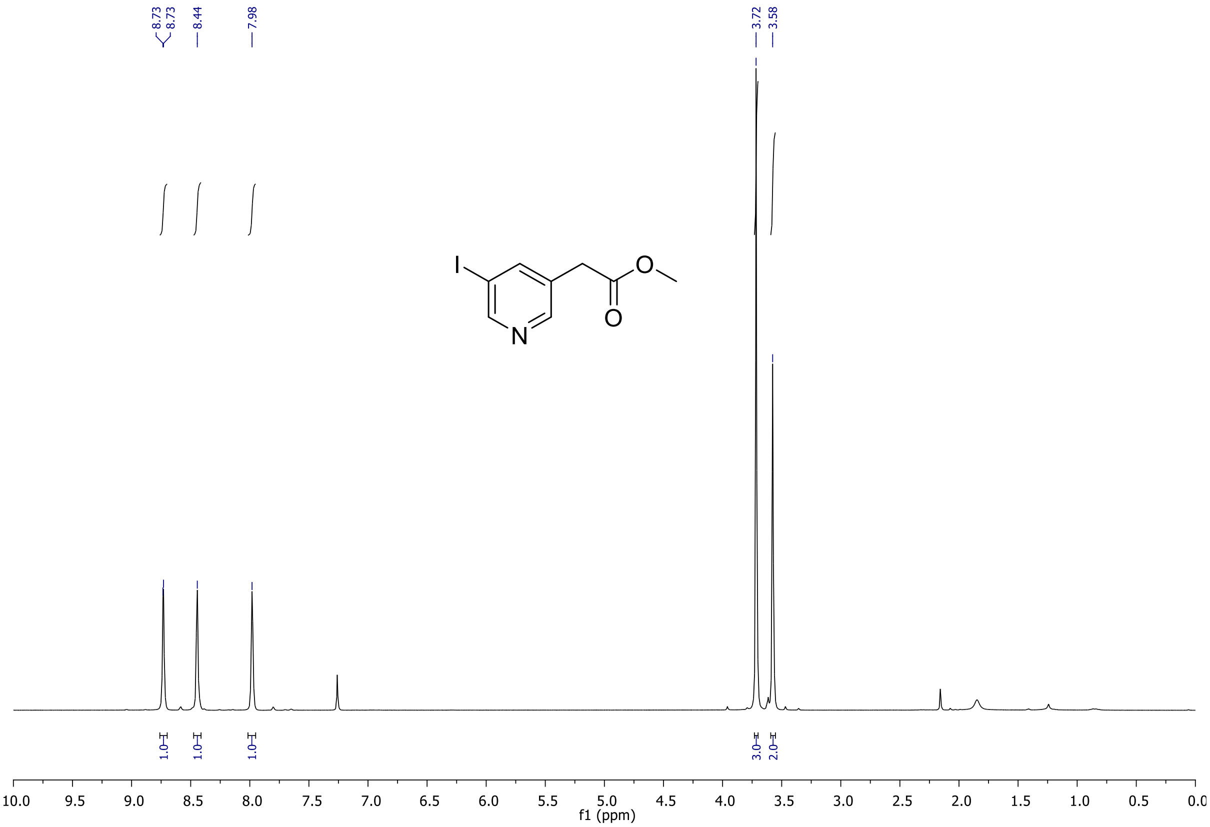

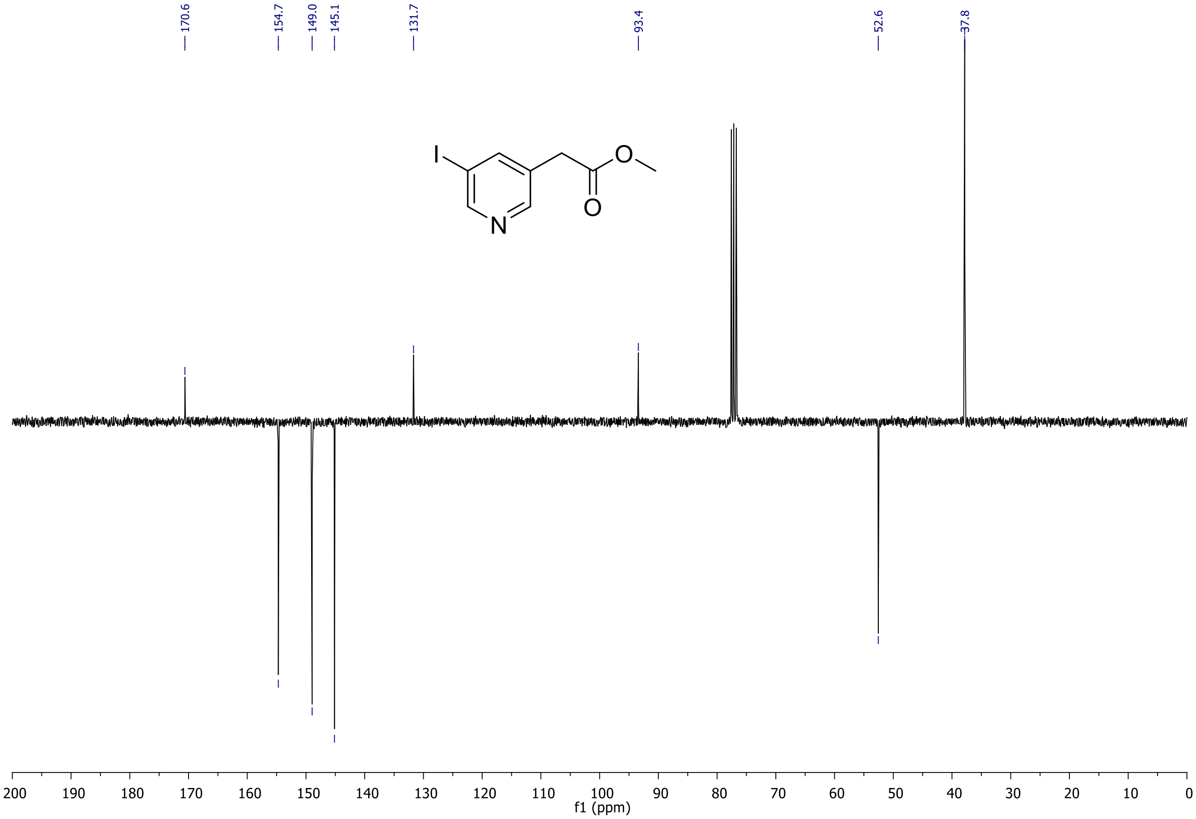

Methyl 2-(5-(4,4,5,5-tetramethyl-1,3,2-dioxaborolan-2-yl)pyridin-3-yl)acetate (**11**)

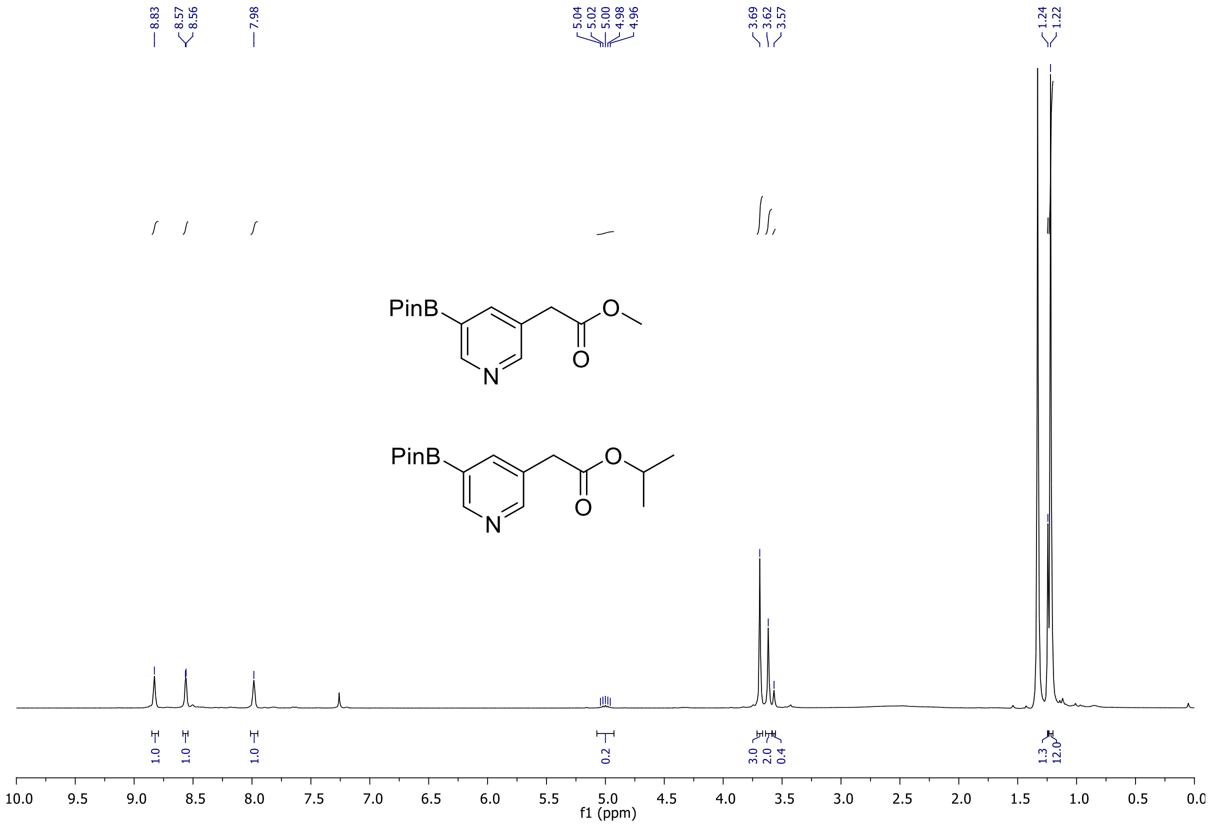

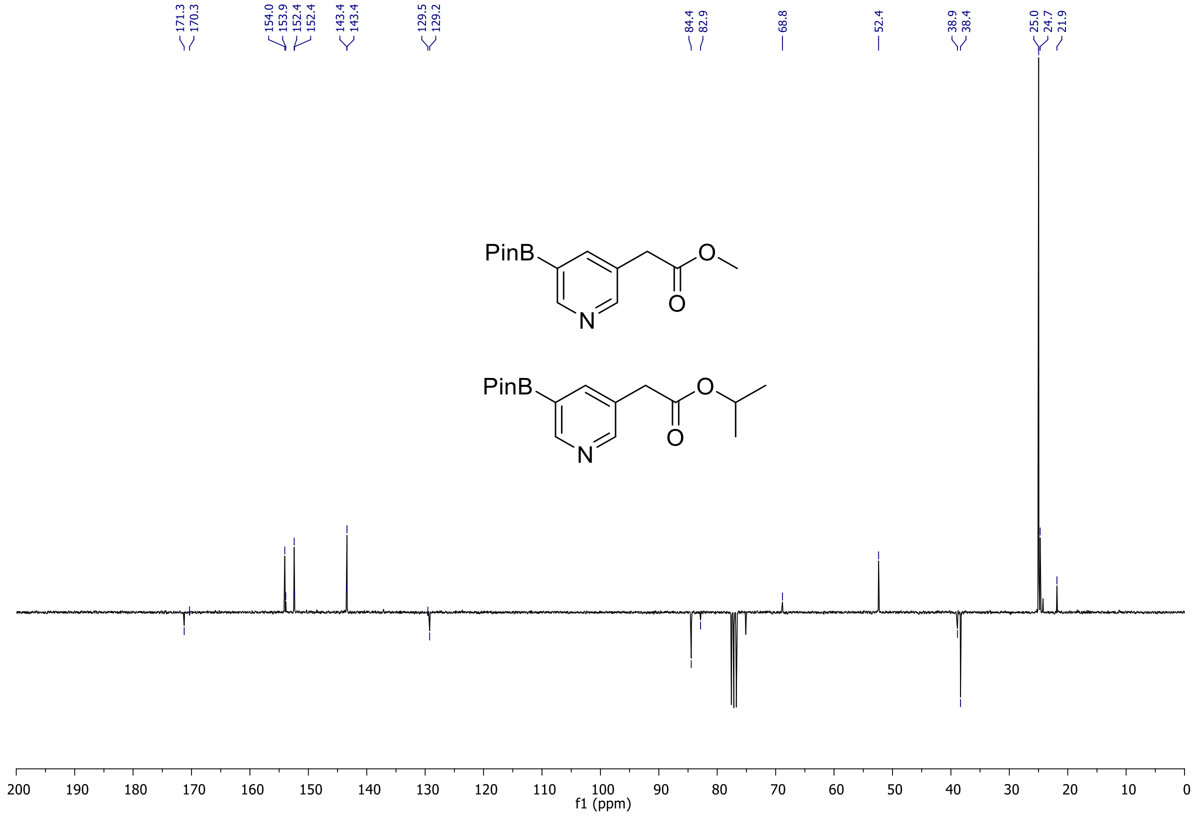

Methyl (*E*)-3-(5-bromopyridin-3-yl)acrylate (**12a**)

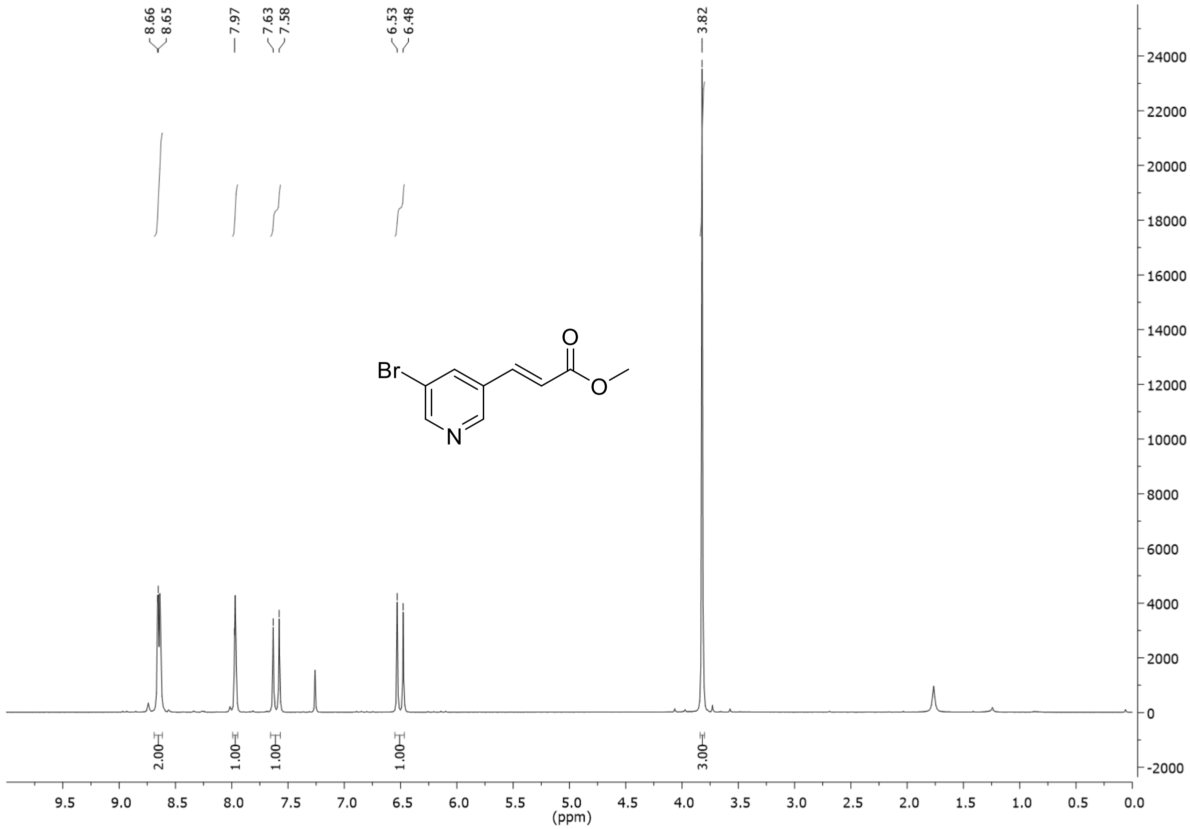

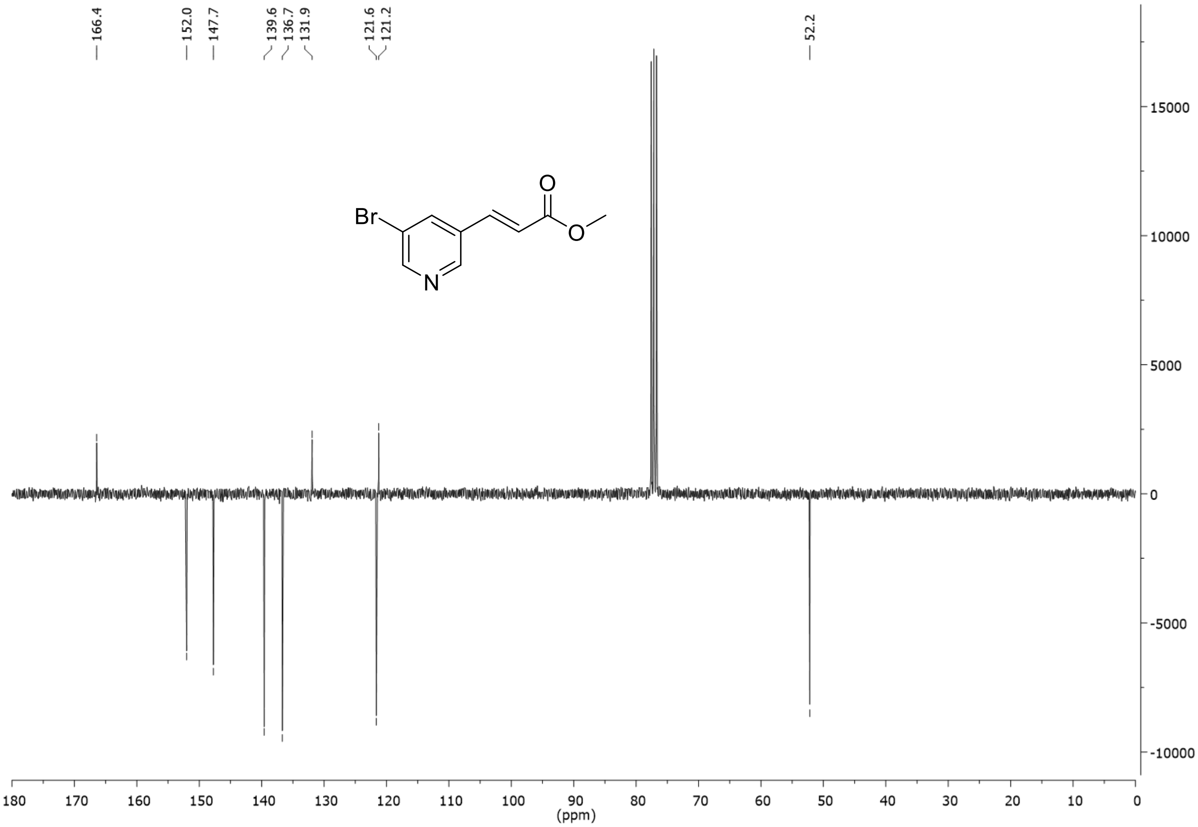

Methyl 3-(5-bromopyridin-3-yl)propanoate (**12**)

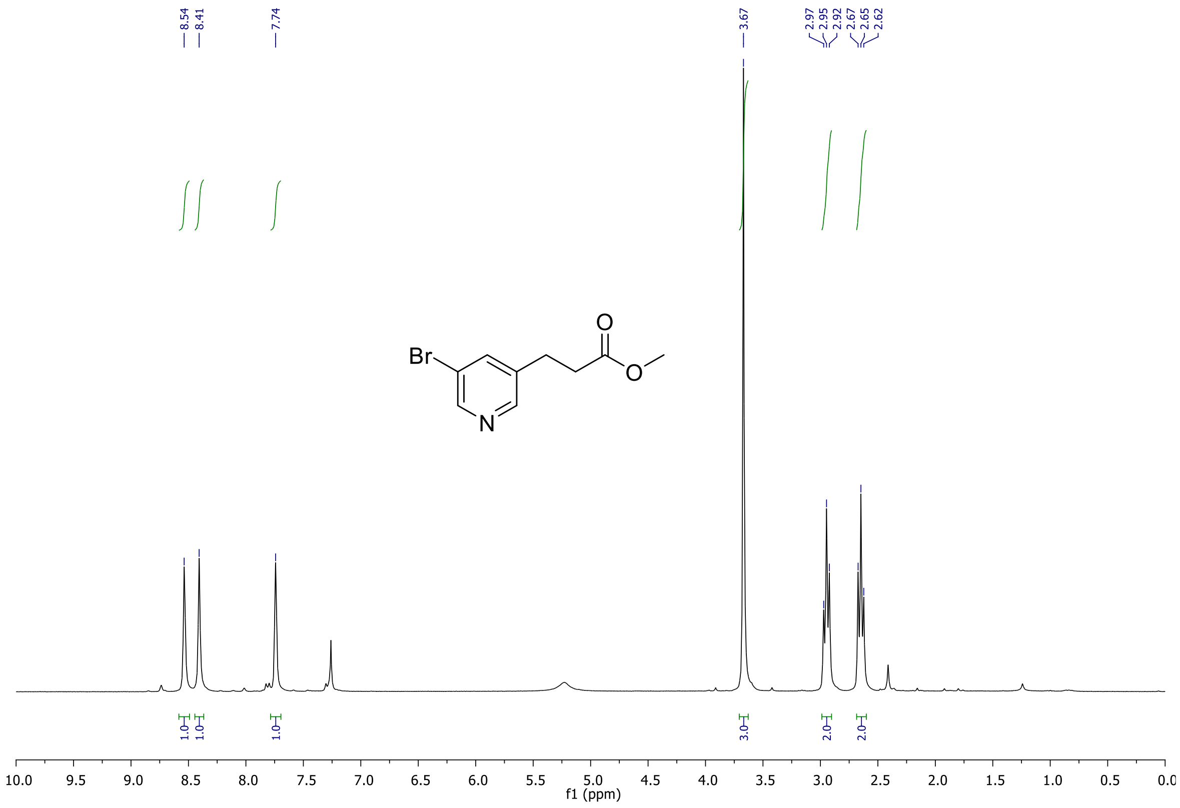

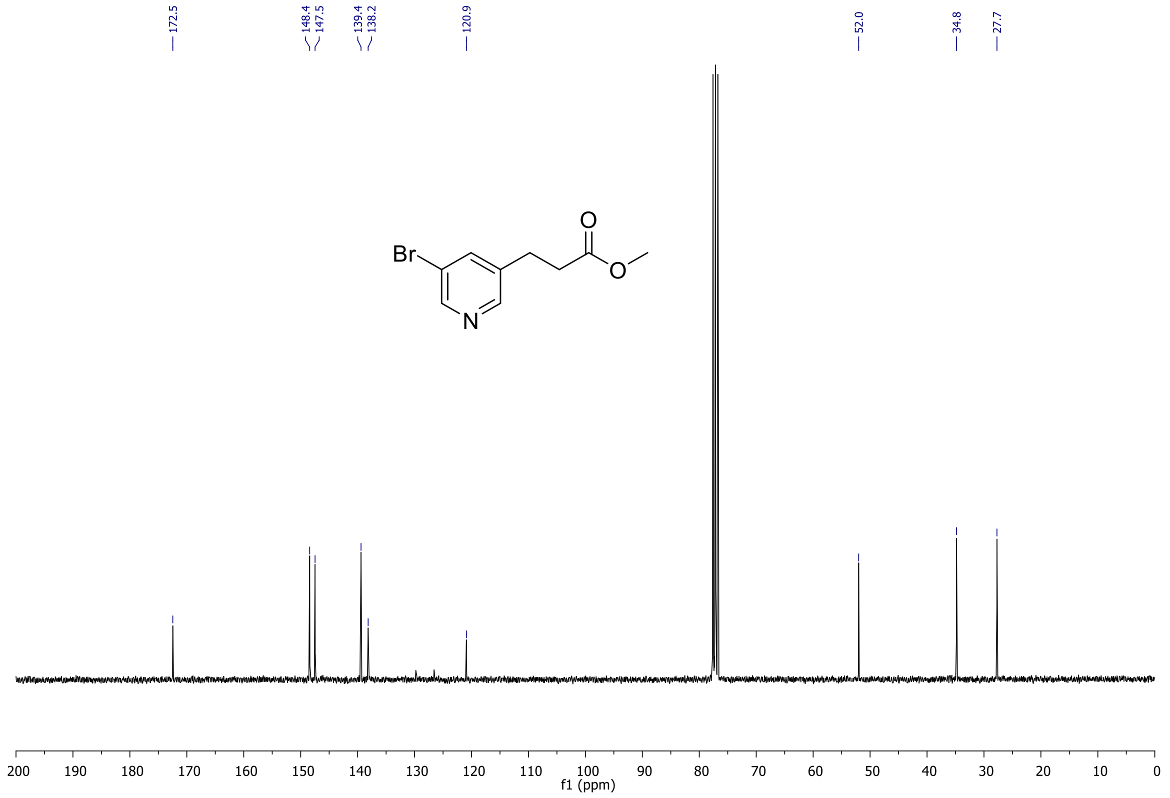

Methyl 3-(5-iodopyridin-3-yl)propanoate (**14a**)

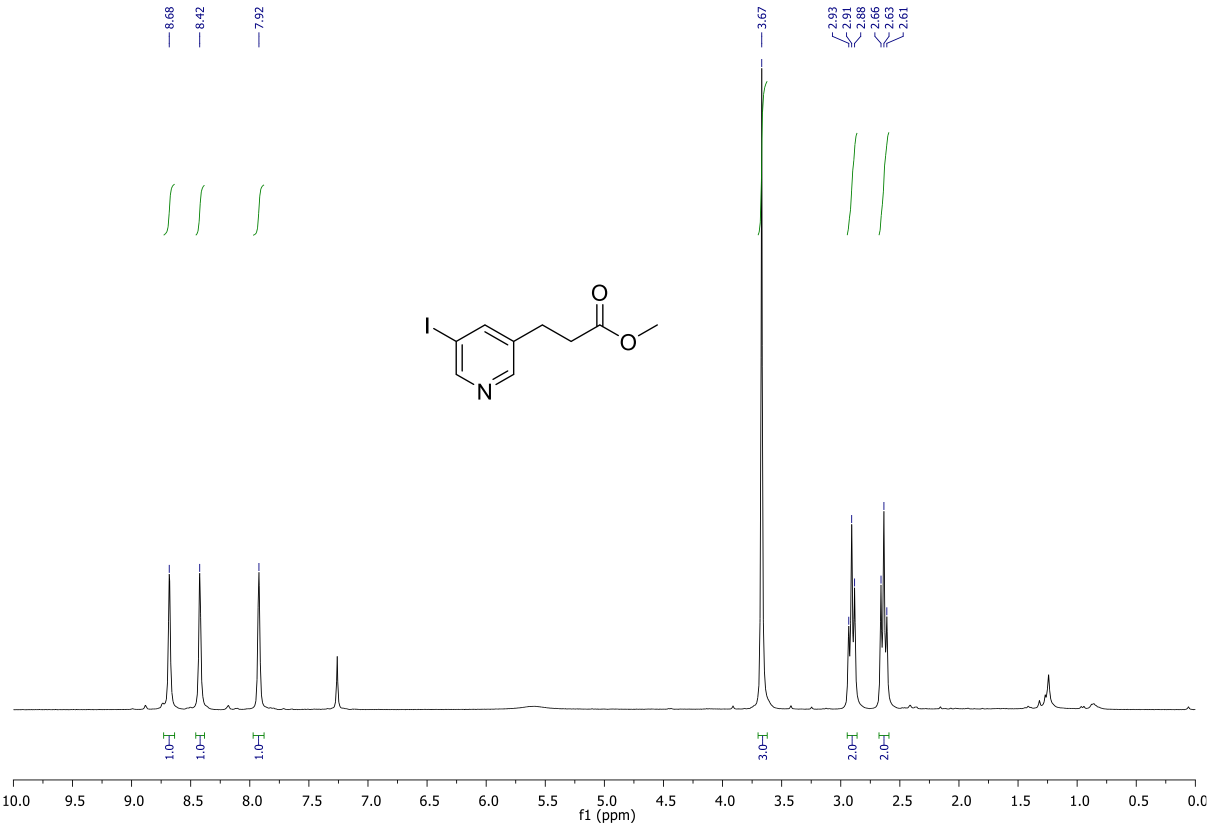

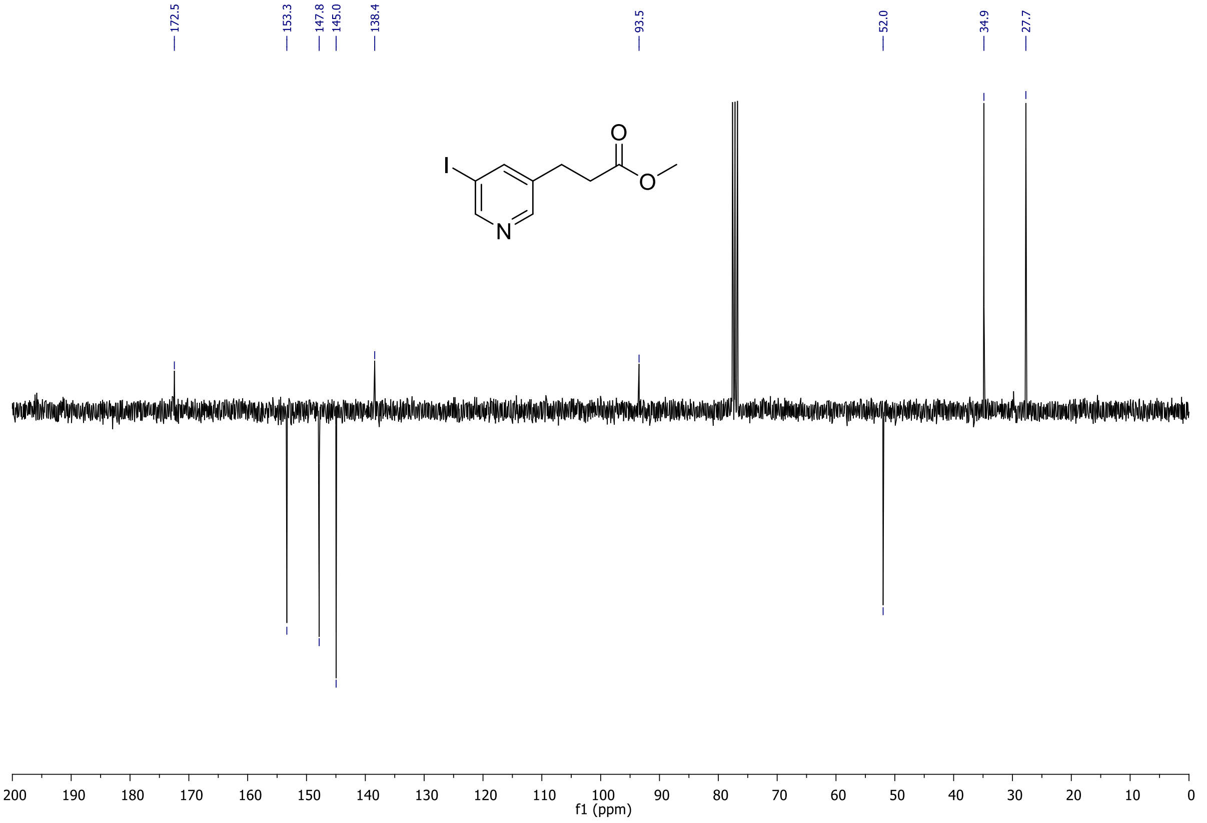

Methyl 3-(5-(4,4,5,5-tetramethyl-1,3,2-dioxaborolan-2-yl)pyridin-3 yl)propanoate (**14**)

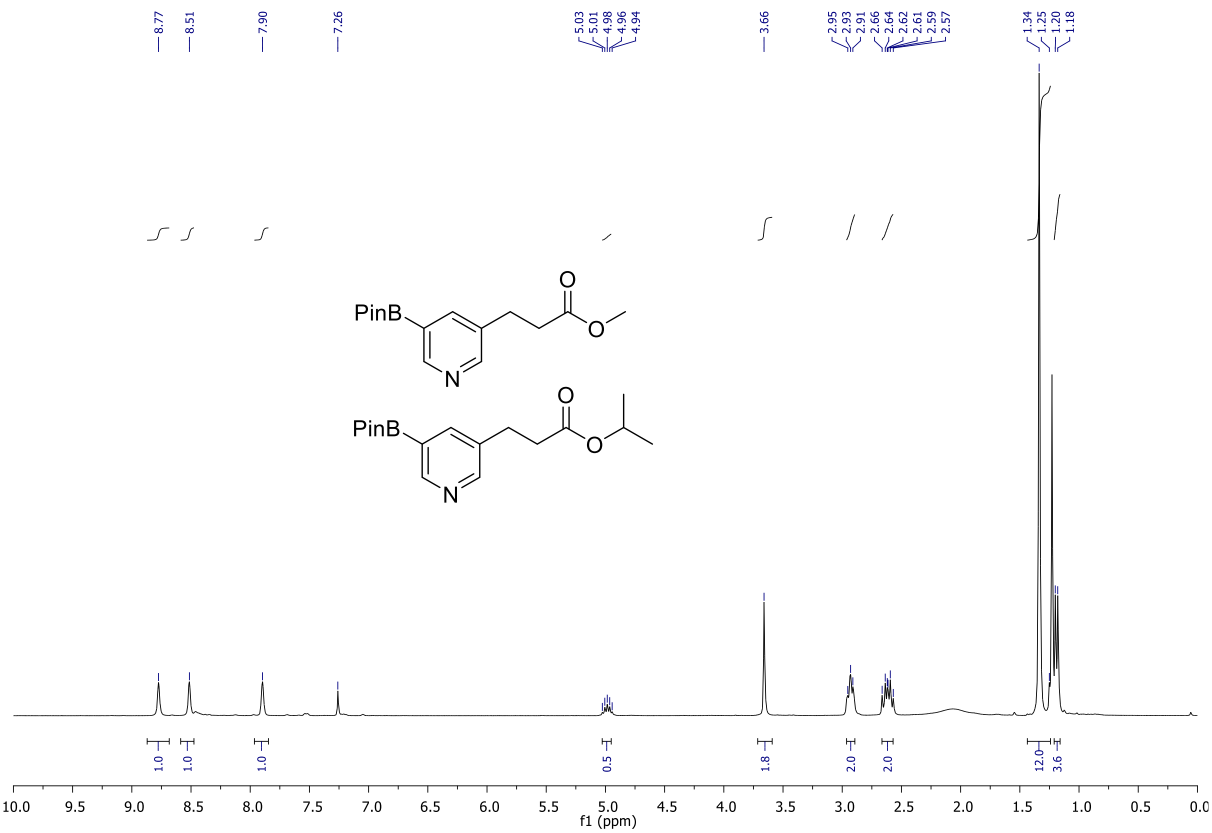

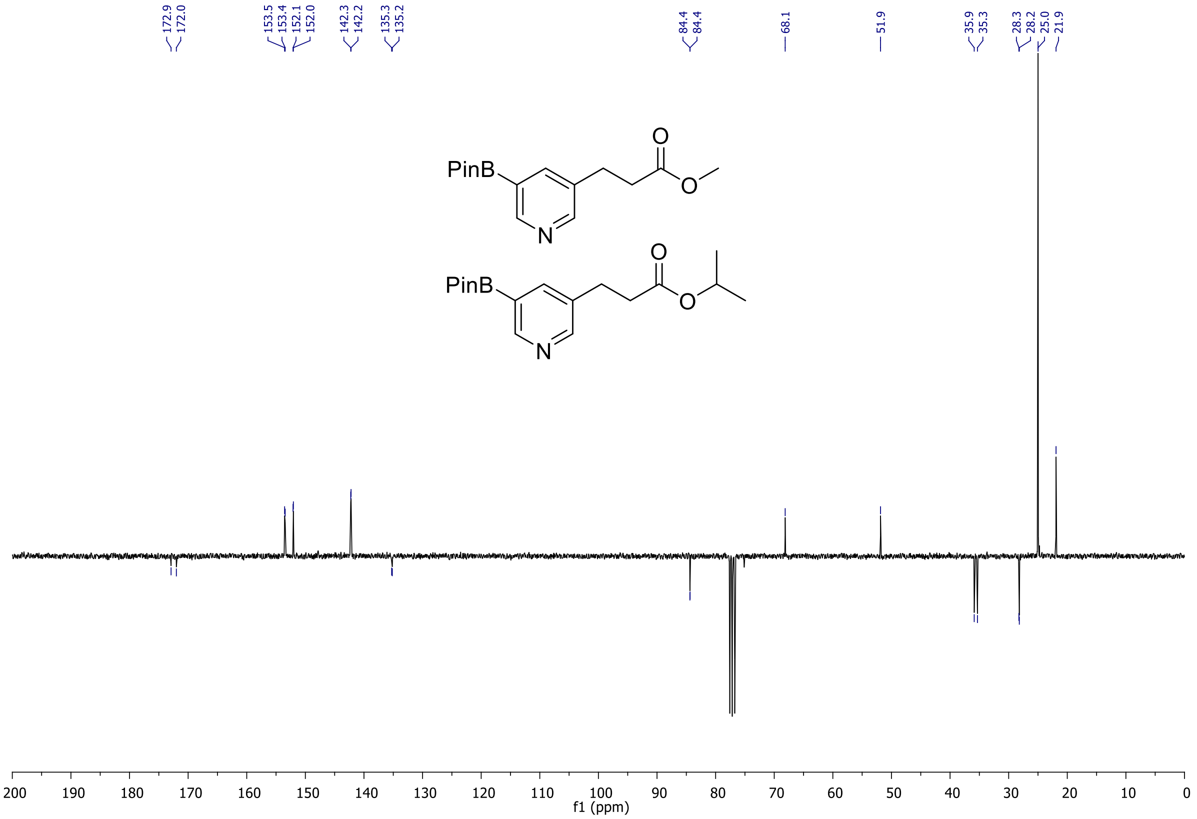

(*E*)-3-(5-Bromopyridin-3-yl)acrylonitrile (**13a**)

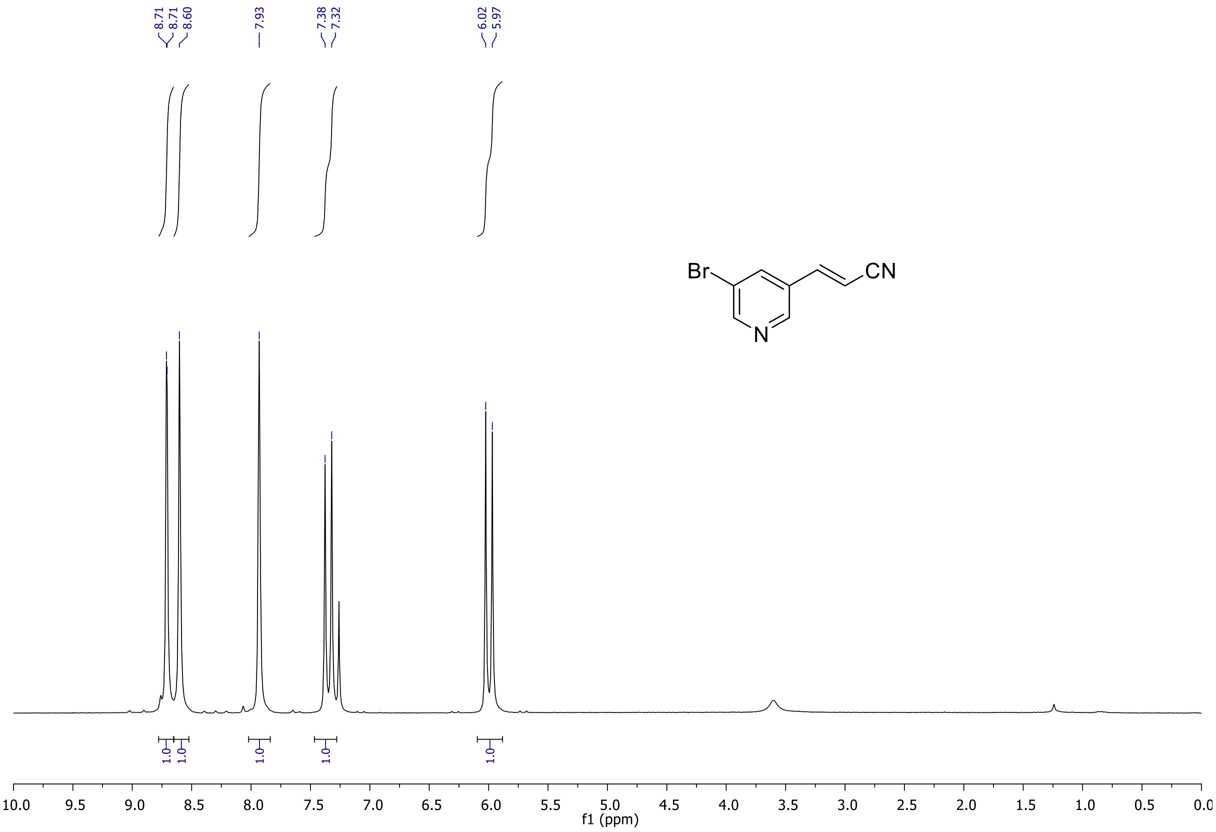

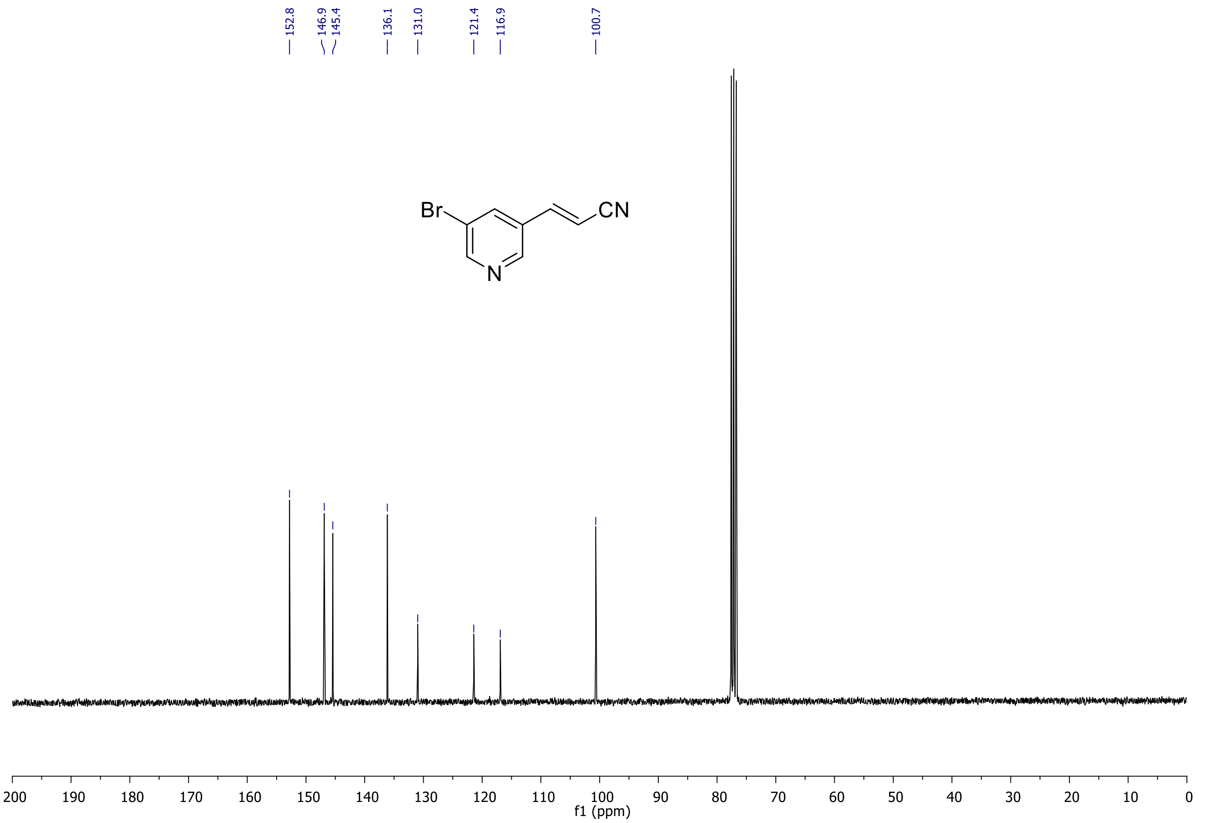

(*Z*)-3-(5-Bromopyridin-3-yl)acrylonitrile (**13a**)

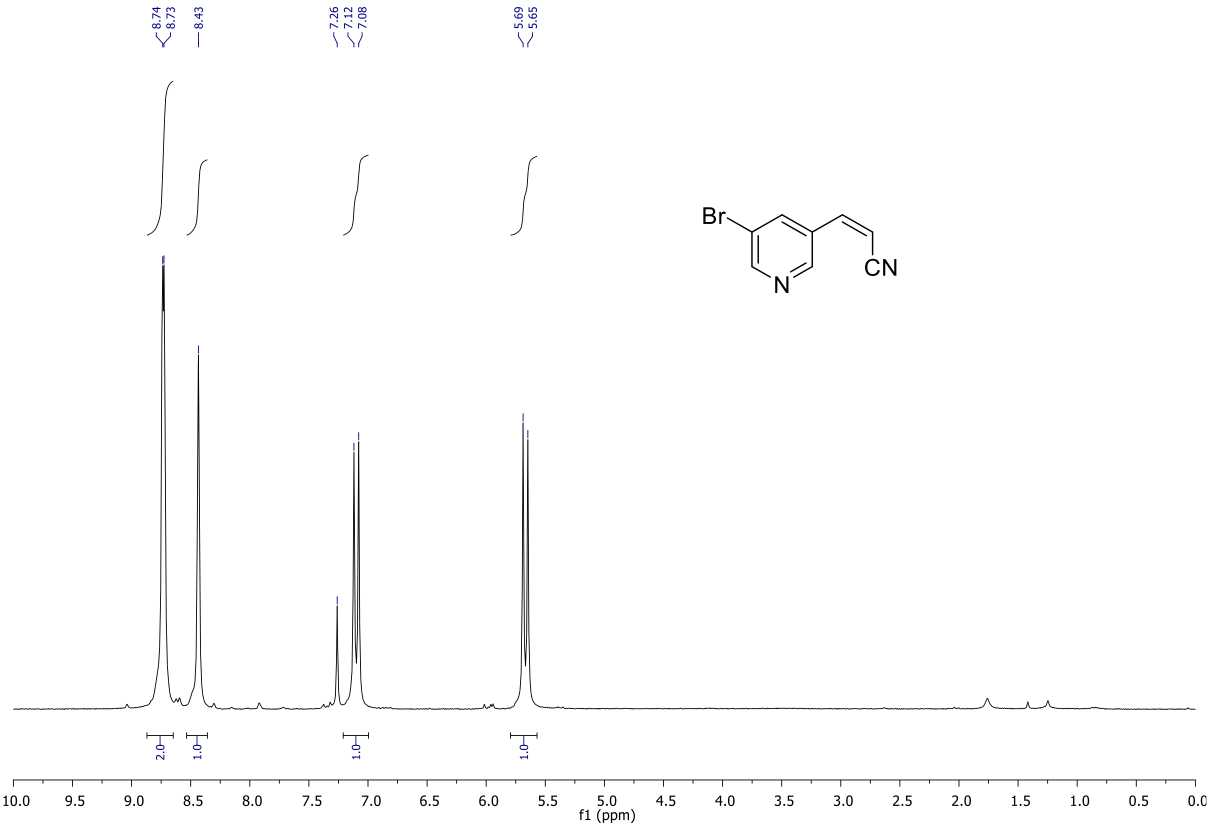

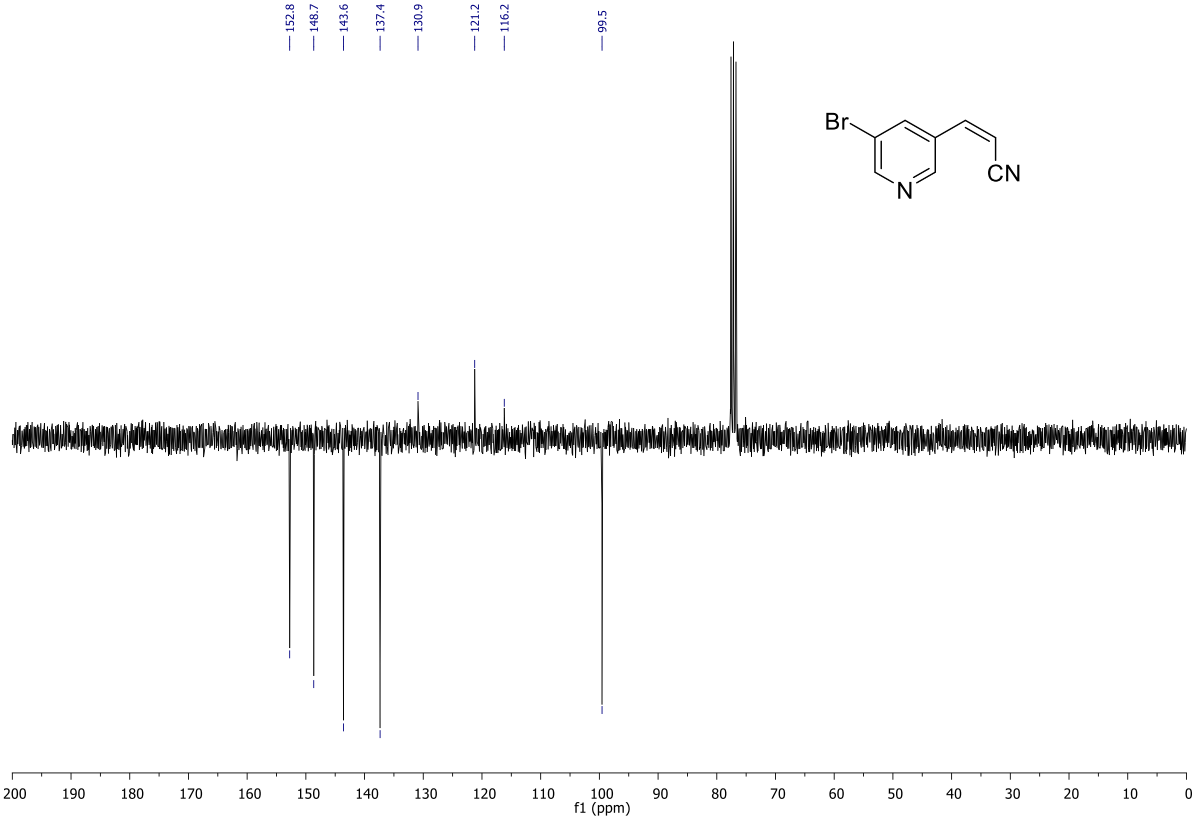

3-(5-Bromopyridin-3-yl)propanenitrile (**13**)

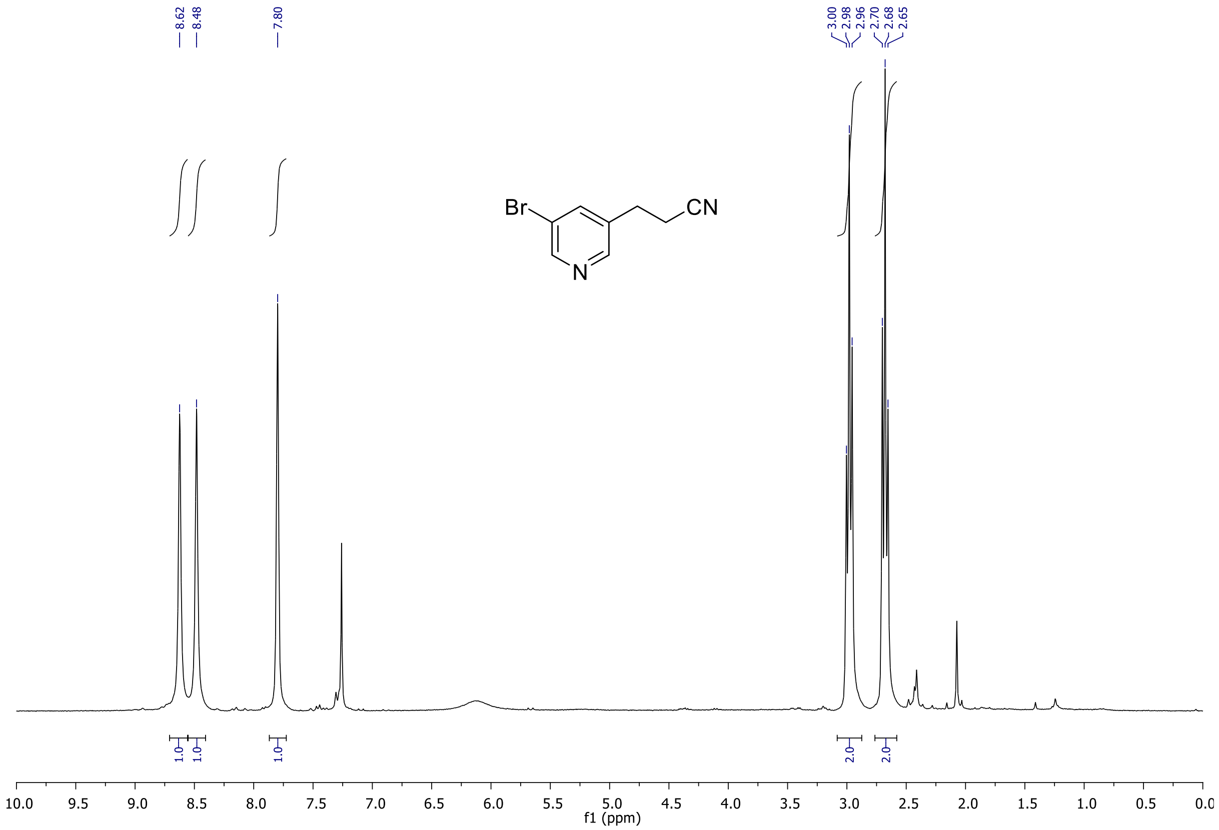

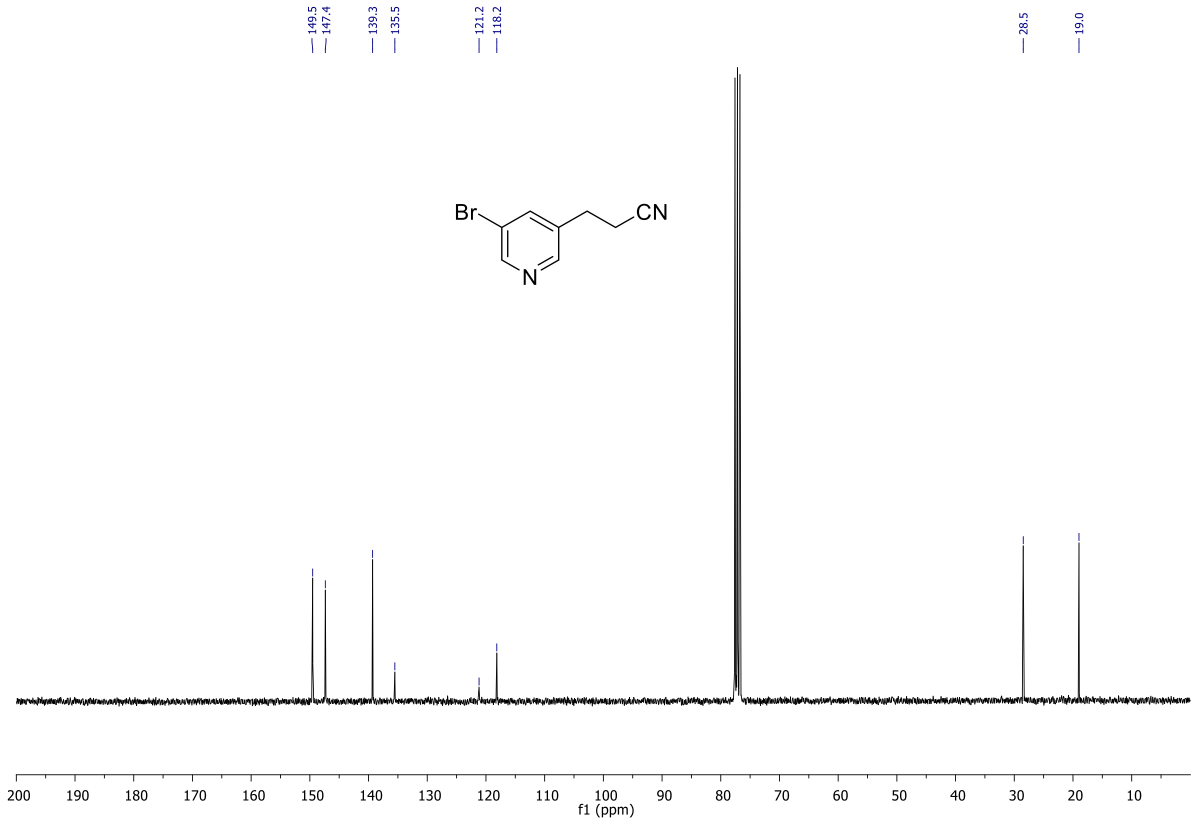

3-(5-Iodopyridin-3-yl)propanenitrile (**15a**)

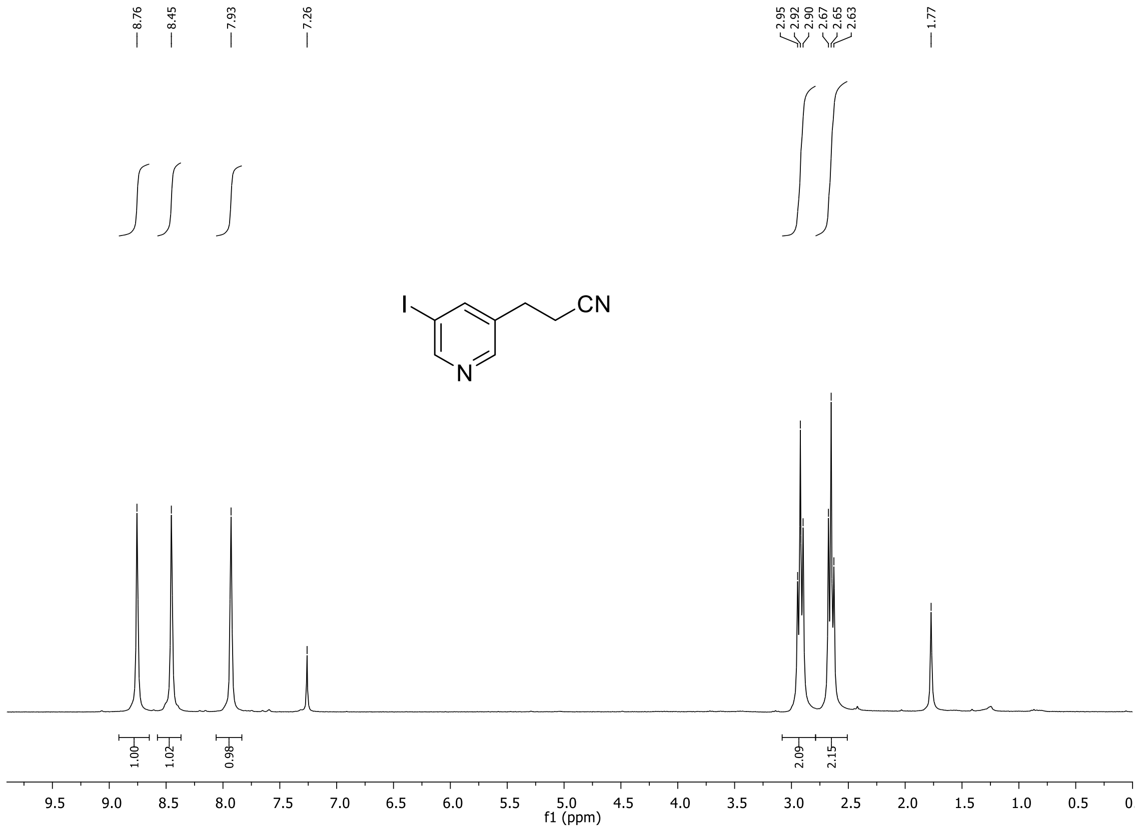

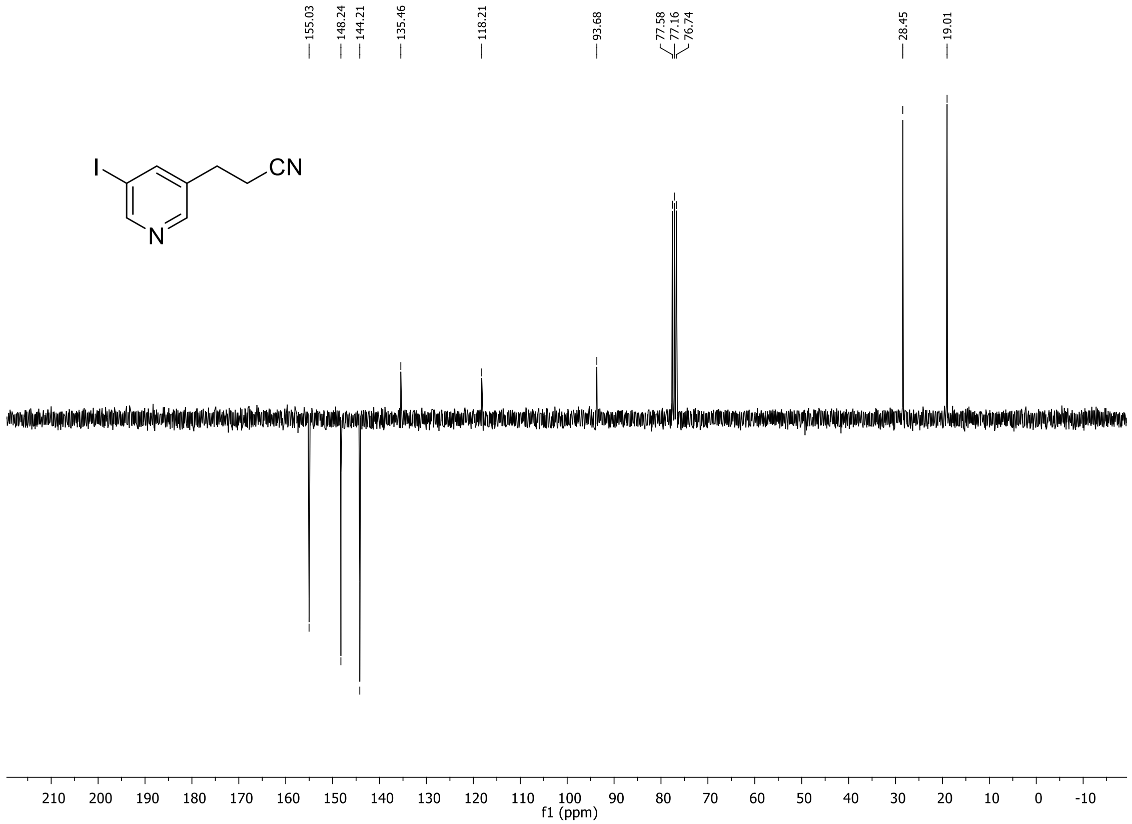

3-(5-(4,4,5,5-Tetramethyl-1,3,2-dioxaborolan-2-yl)pyridin-3-yl)propanenitrile (**15**)

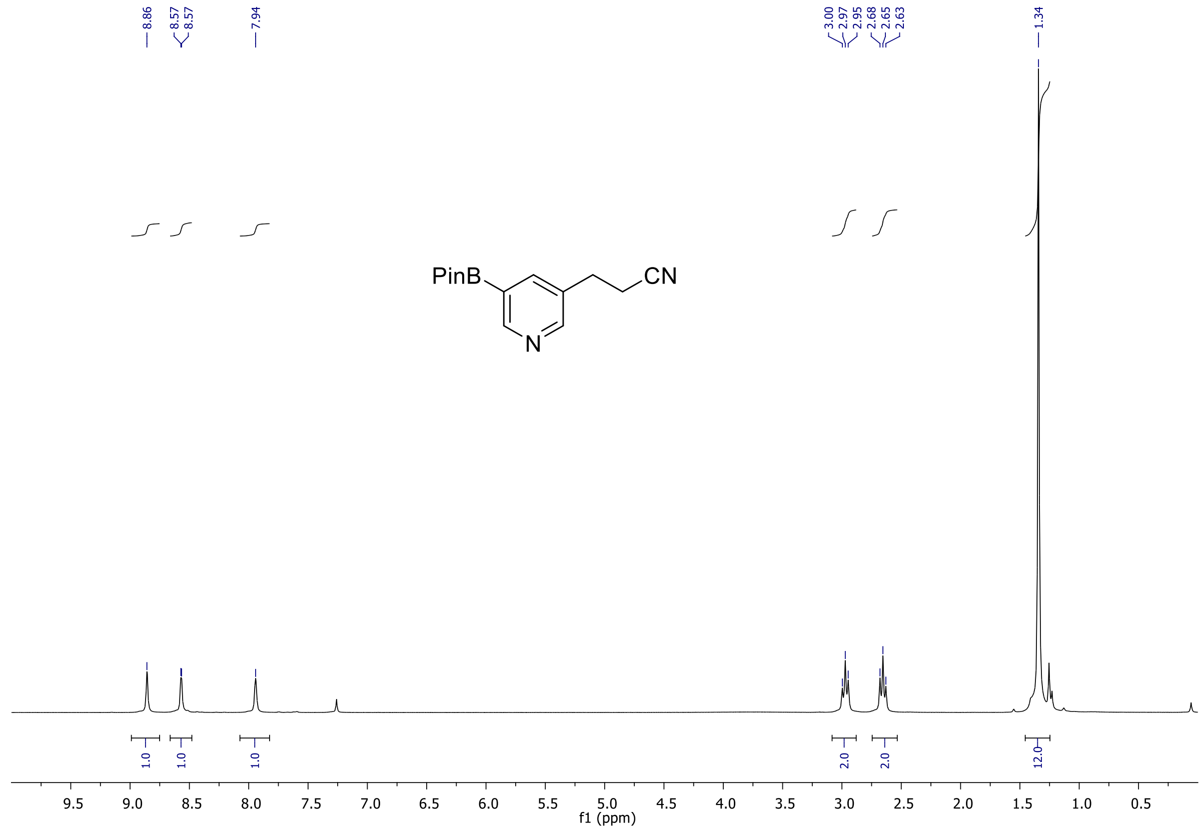

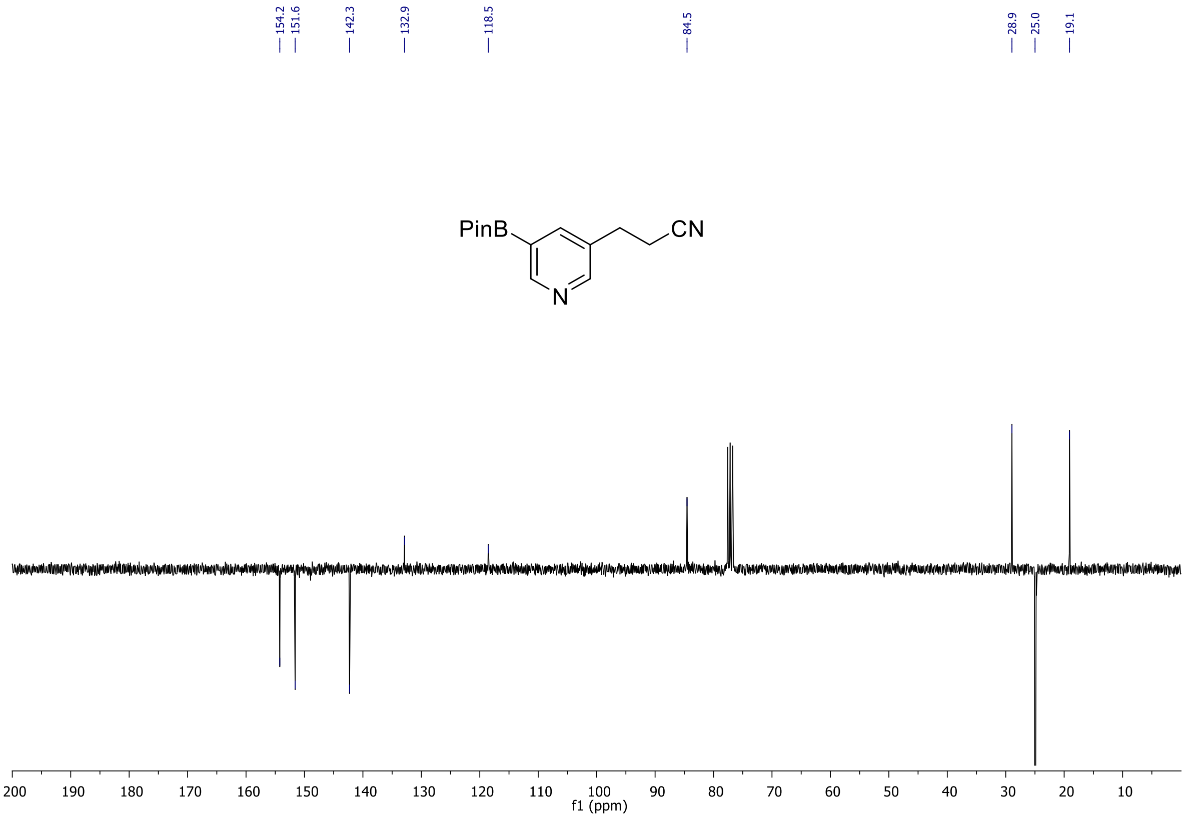

3-(2-(1,3-Dioxolan-2-yl)ethyl)-5-bromopyridine (**16a**)

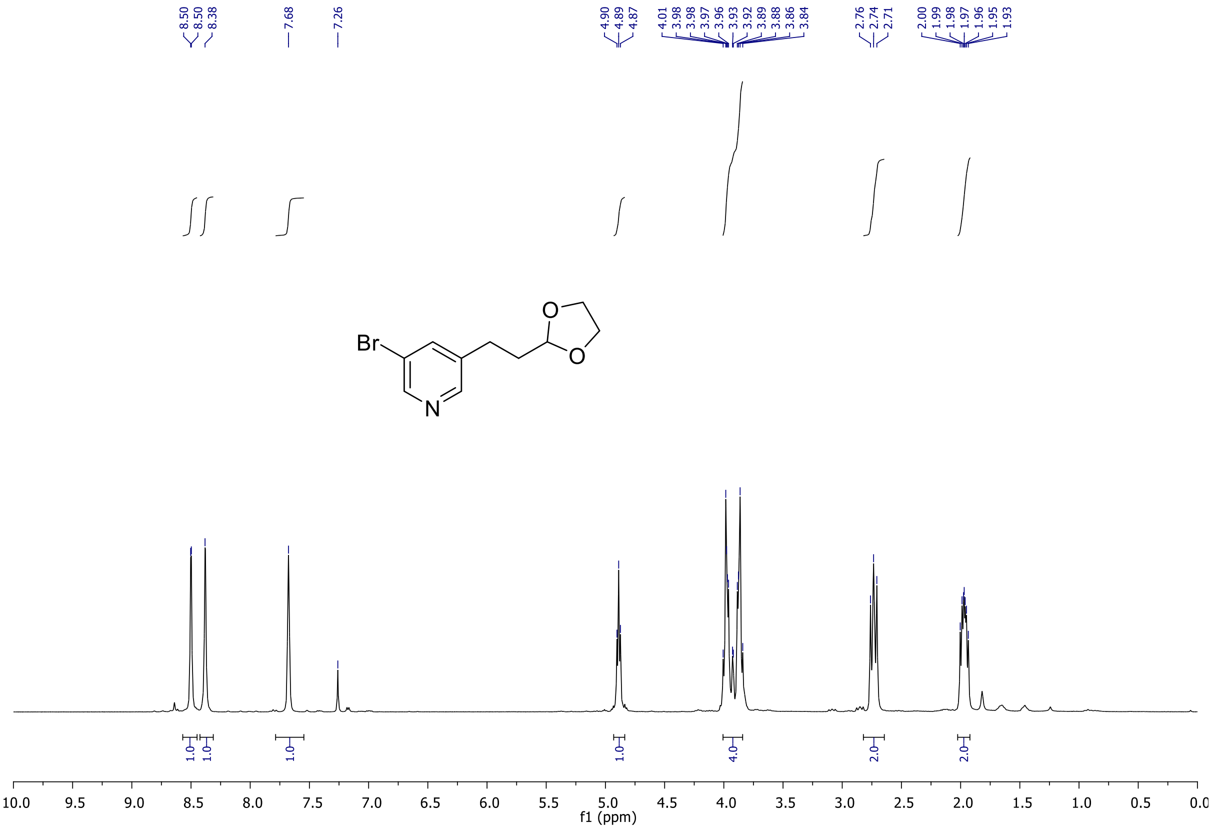

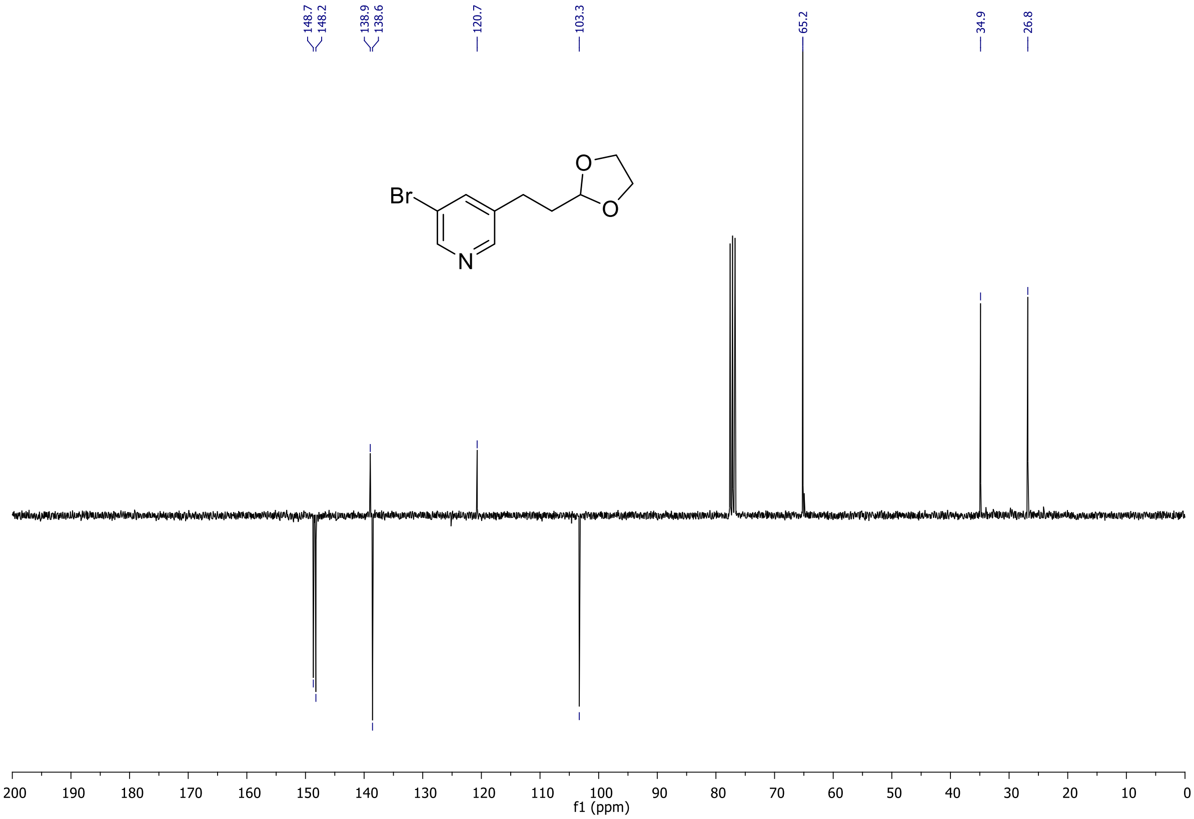

3-((5-Bromopyridin-3-yl)methyl)-1*H*-indole (**16**)

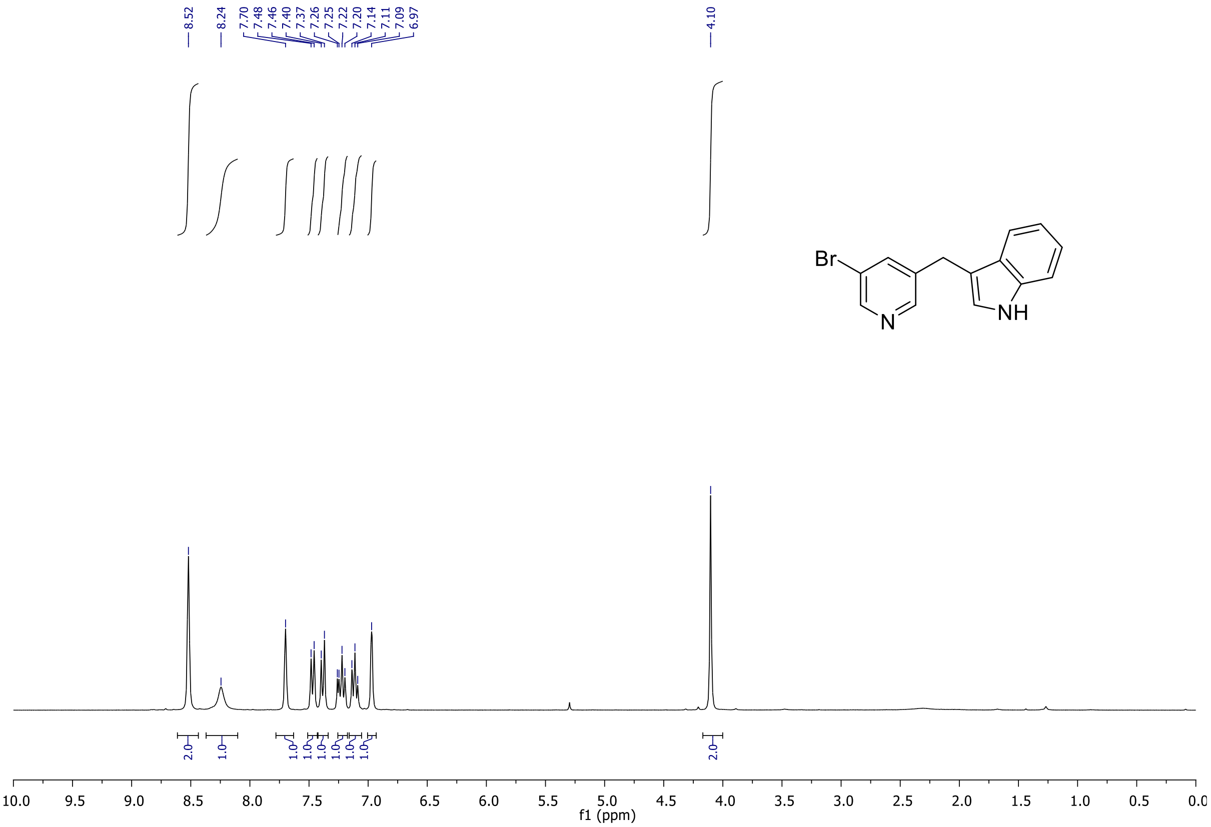

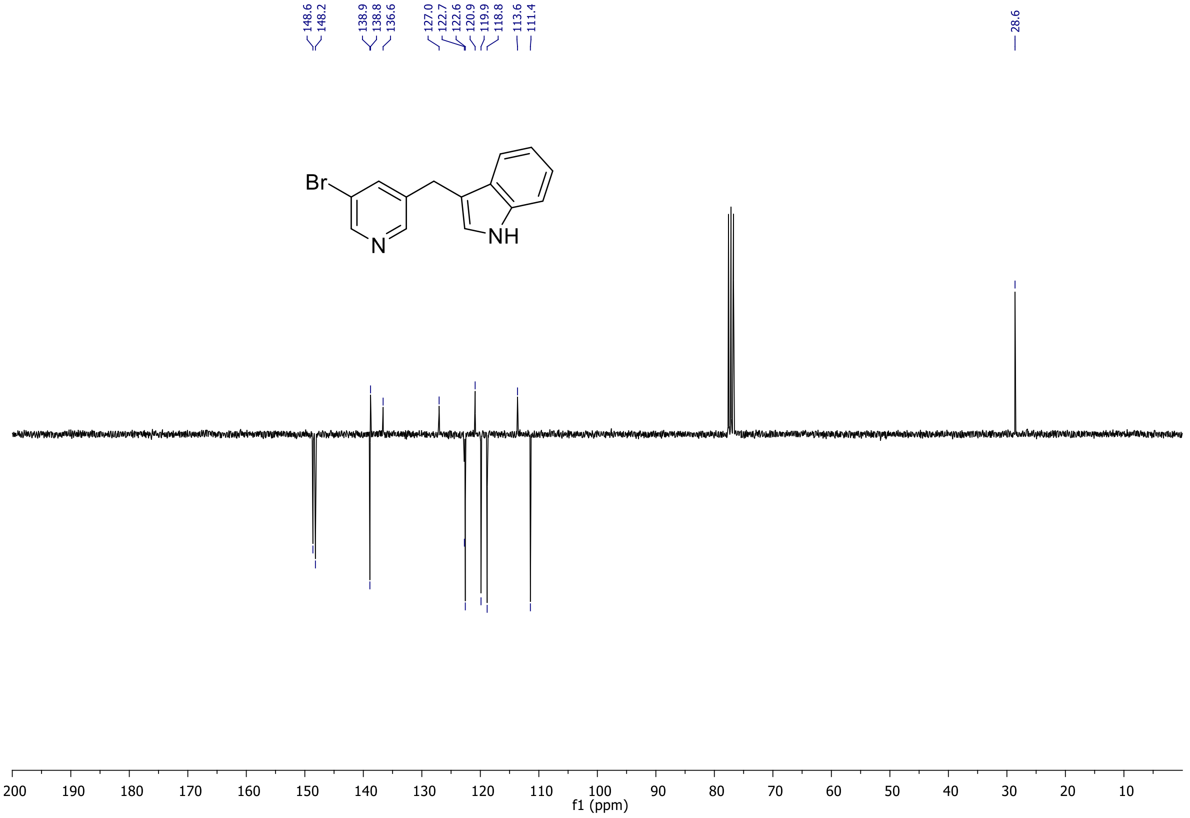

3-((5-(4,4,5,5-Tetramethyl-1,3,2-dioxaborolan-2-yl)pyridin-3-yl)methyl)-1*H*-indole (**17**)

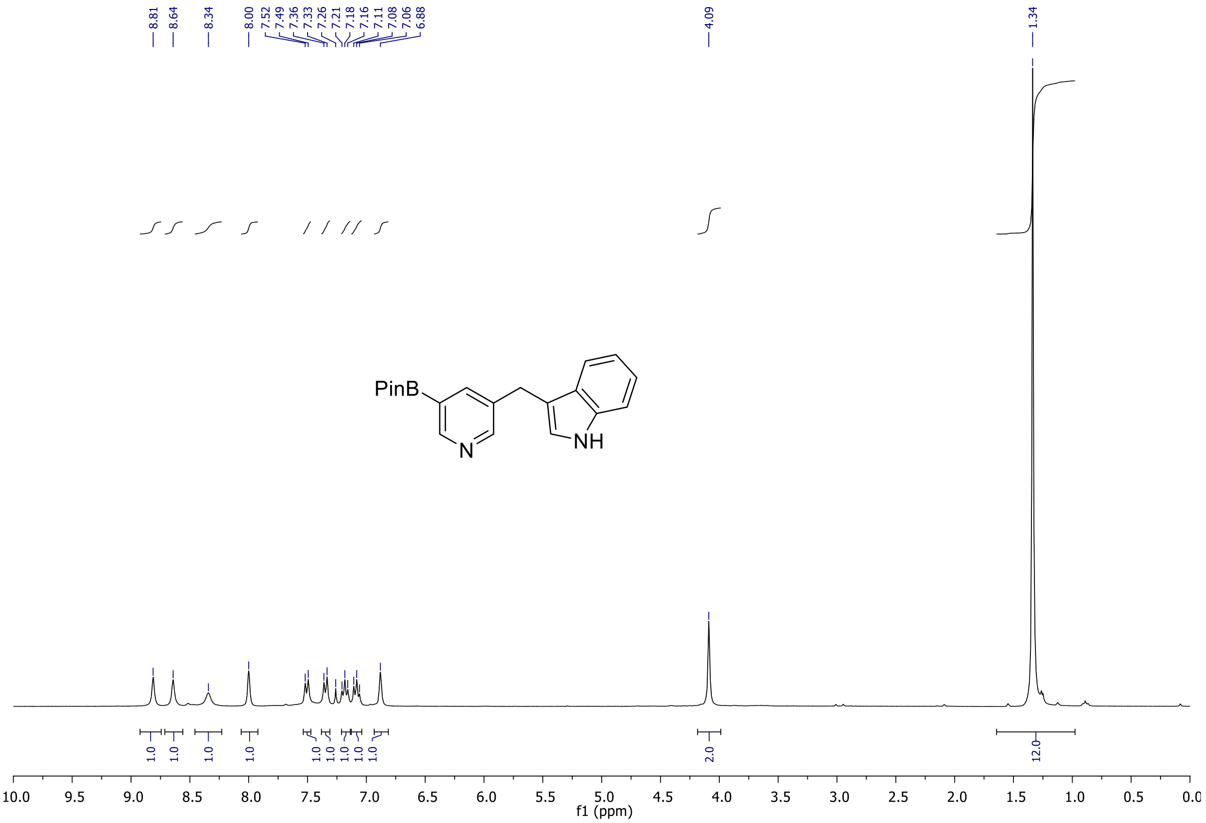

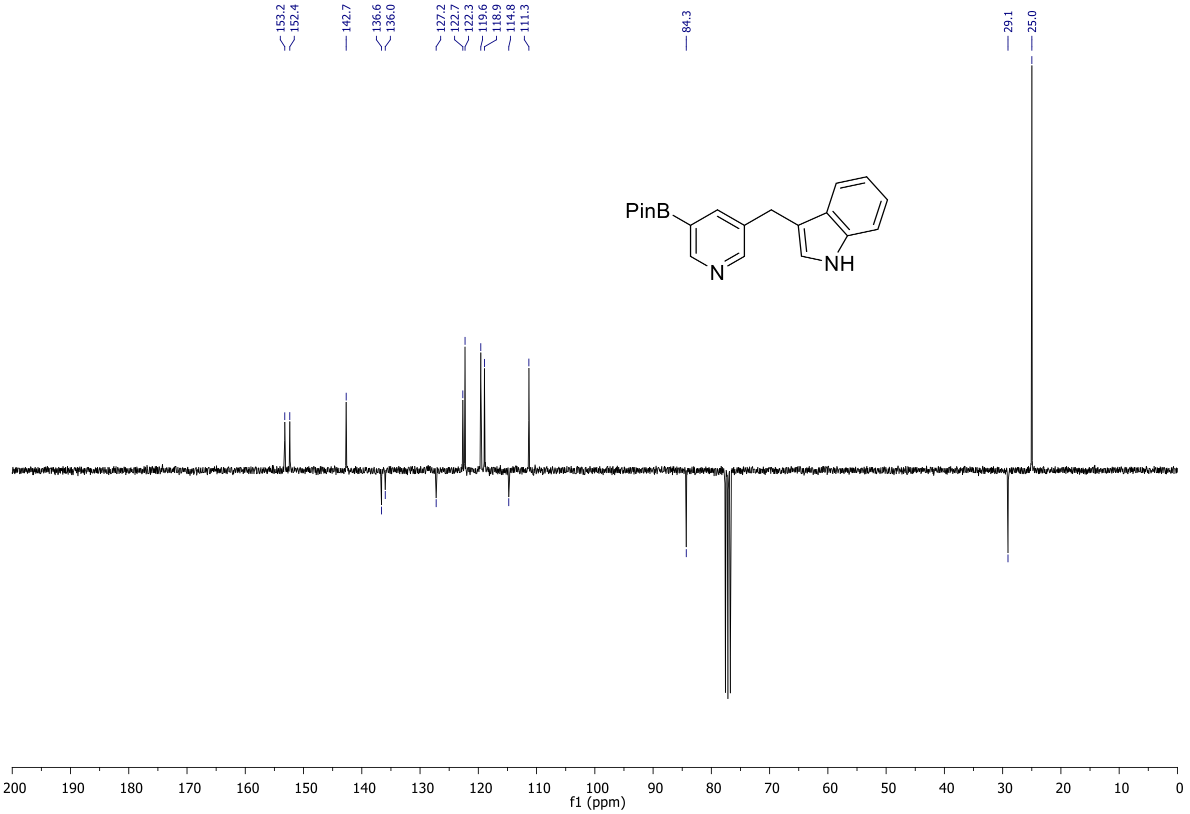

2-(5-(4,4,5,5-Tetramethyl-1,3,2-dioxaborolan-2-yl)pyridin-3-yl)acetamide (**18**)

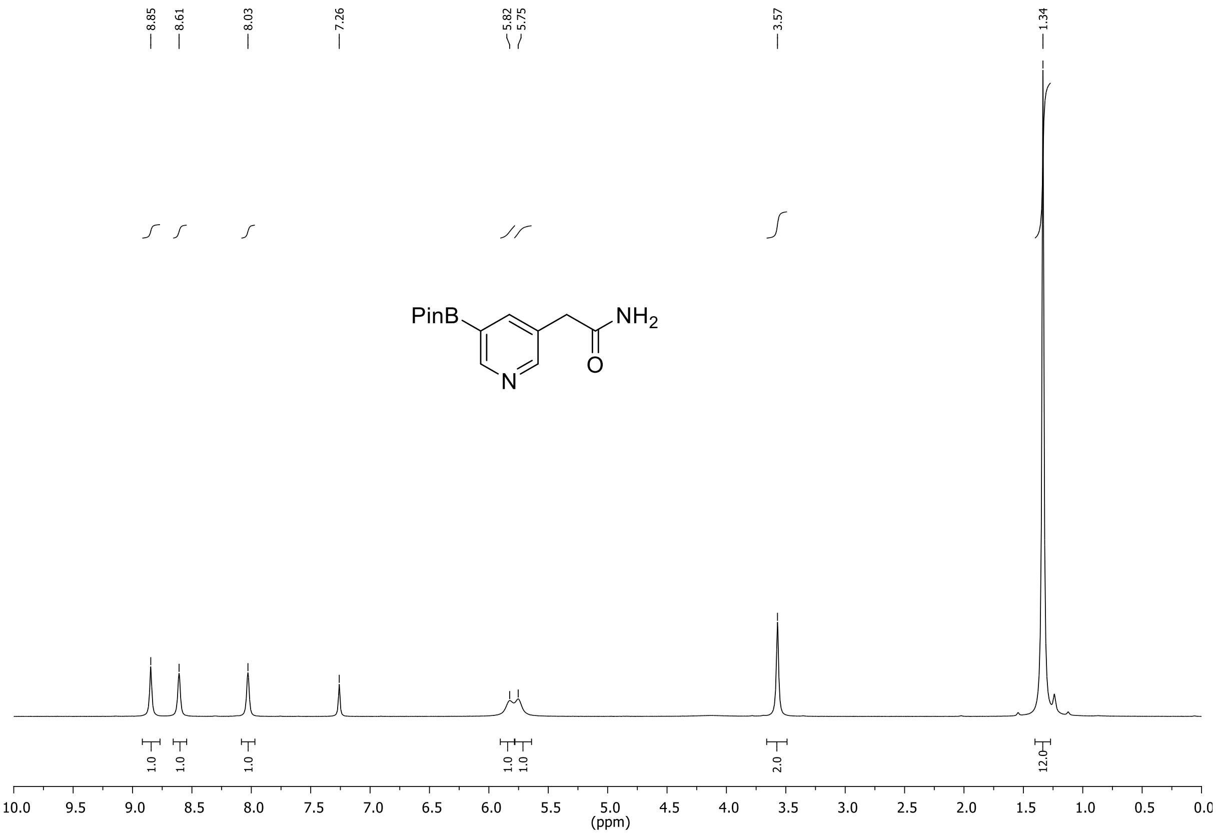

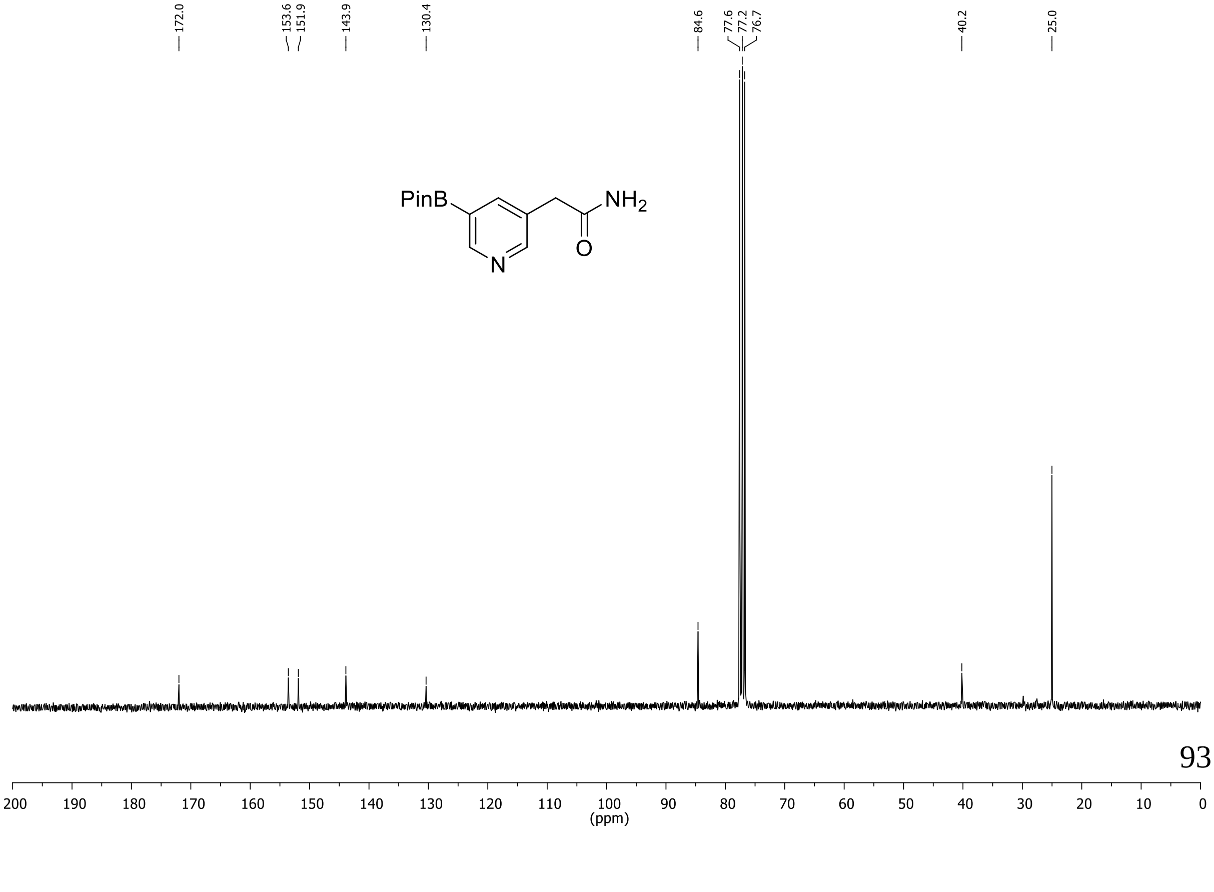

1-Trityl-1*H*-imidazole-4-carbaldehyde (**19**)

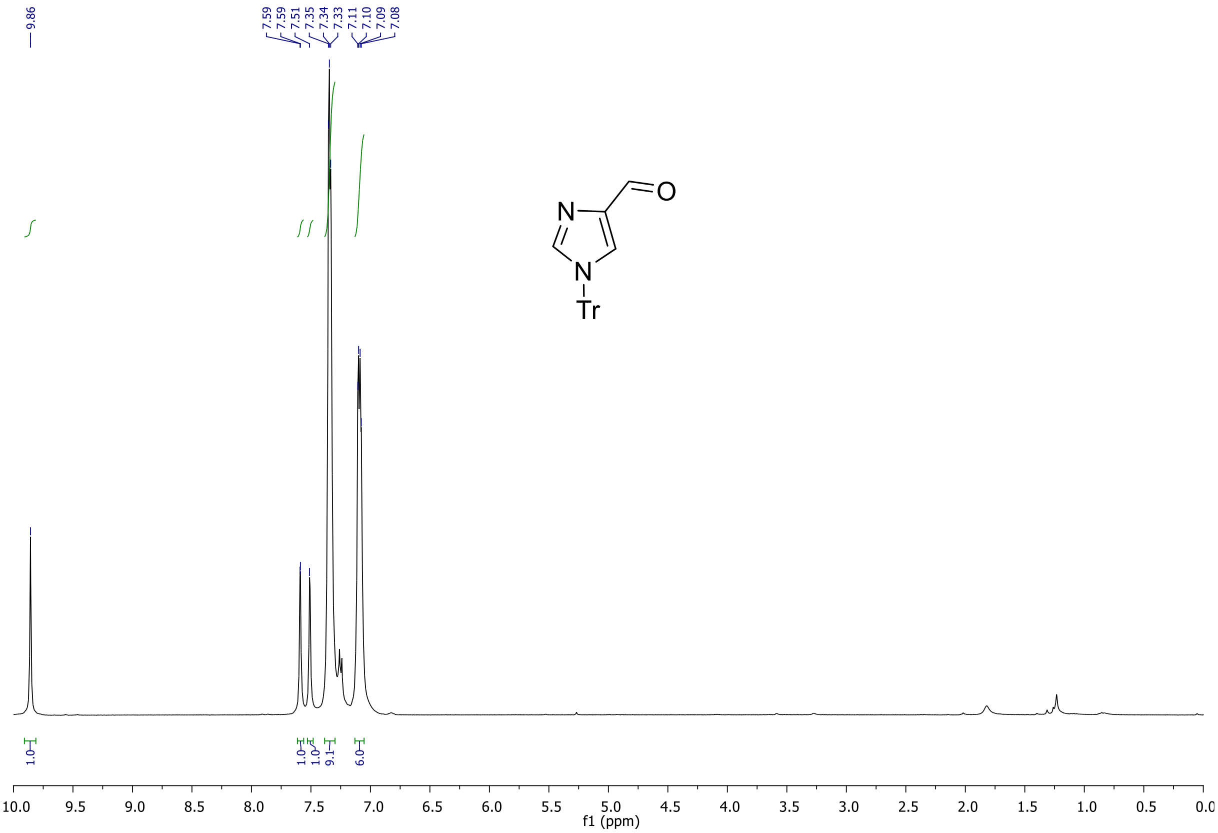

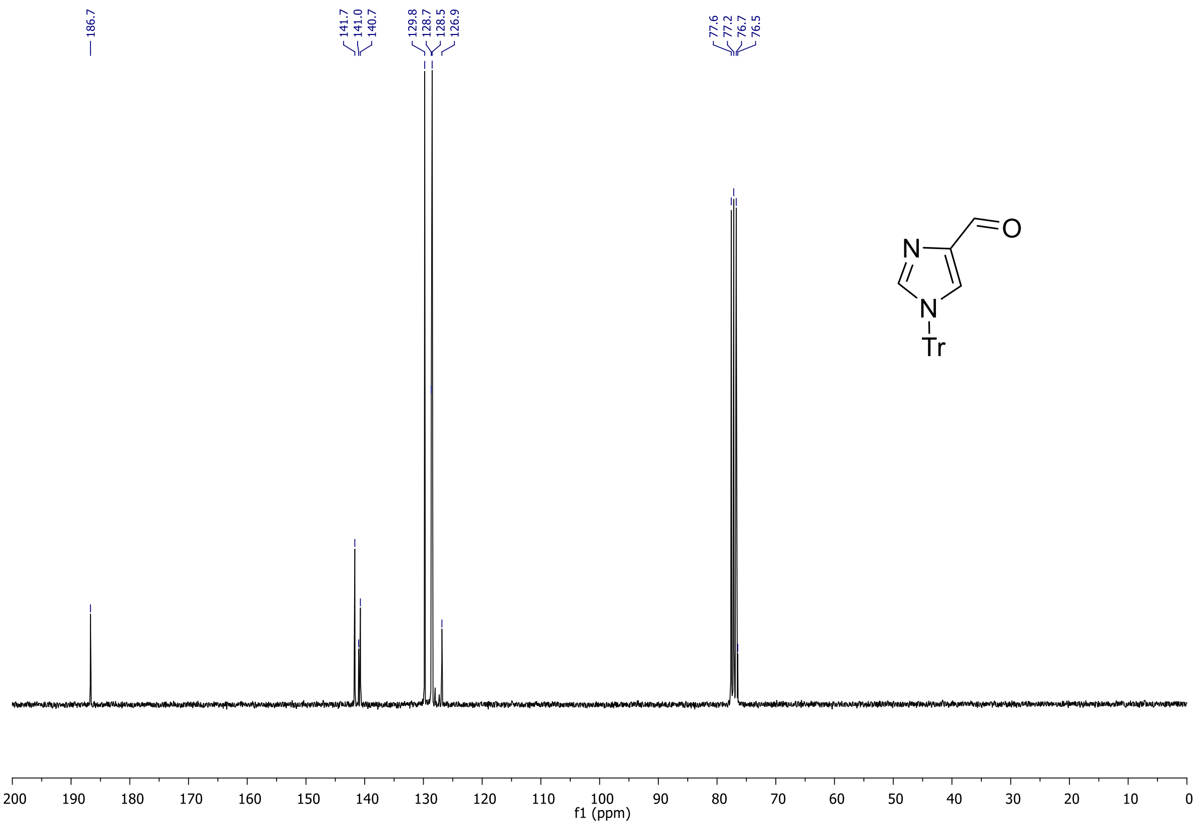

(5-Bromopyridin-3-yl)(1-trityl-1*H*-imidazol-4-yl)methanol (**20a**)

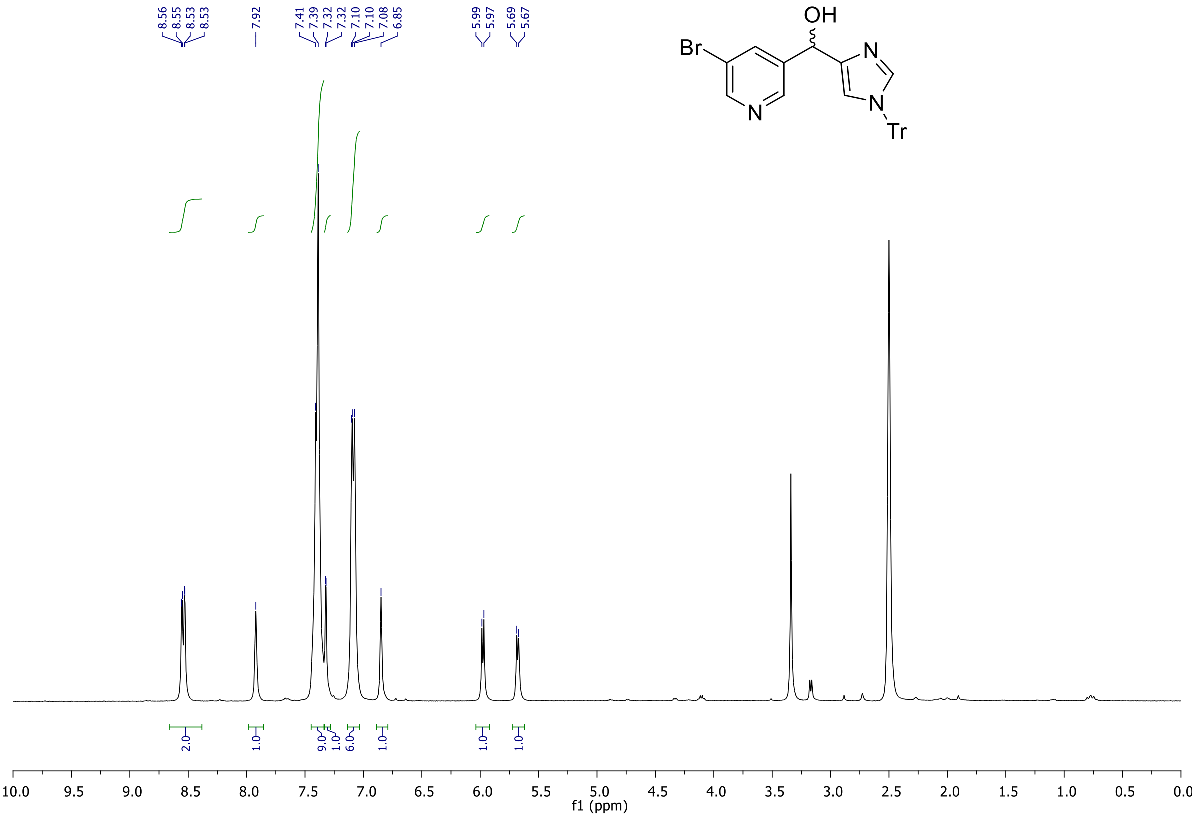

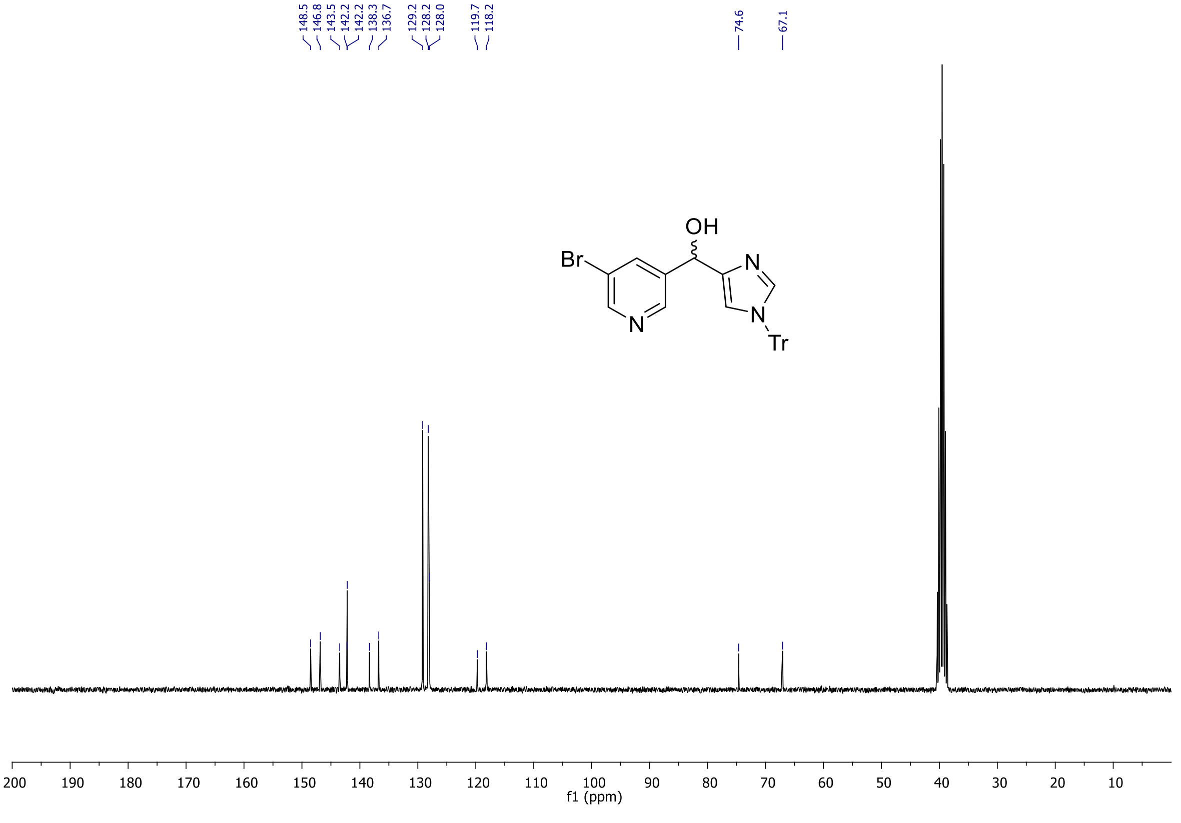

(5-Bromopyridin-3-yl)(1-trityl-1*H*-imidazol-4-yl)methyl acetate (**20**)

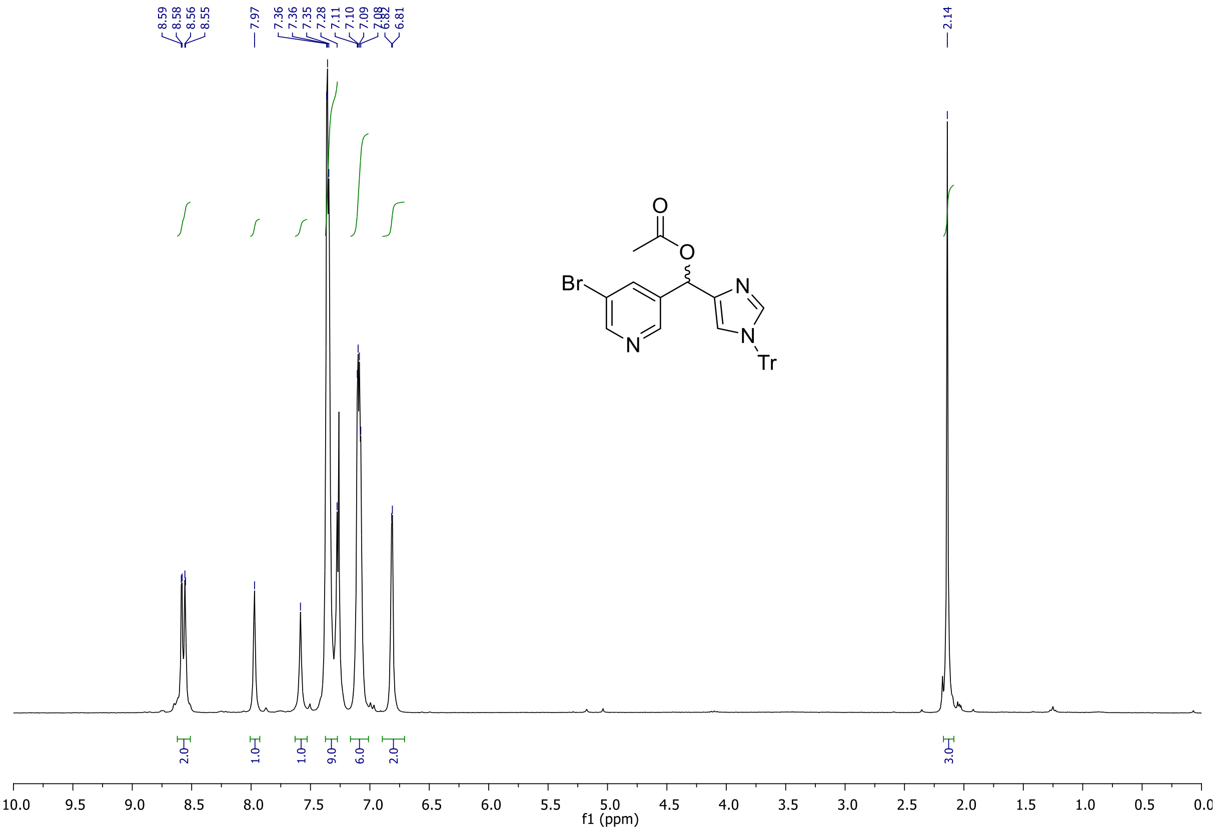

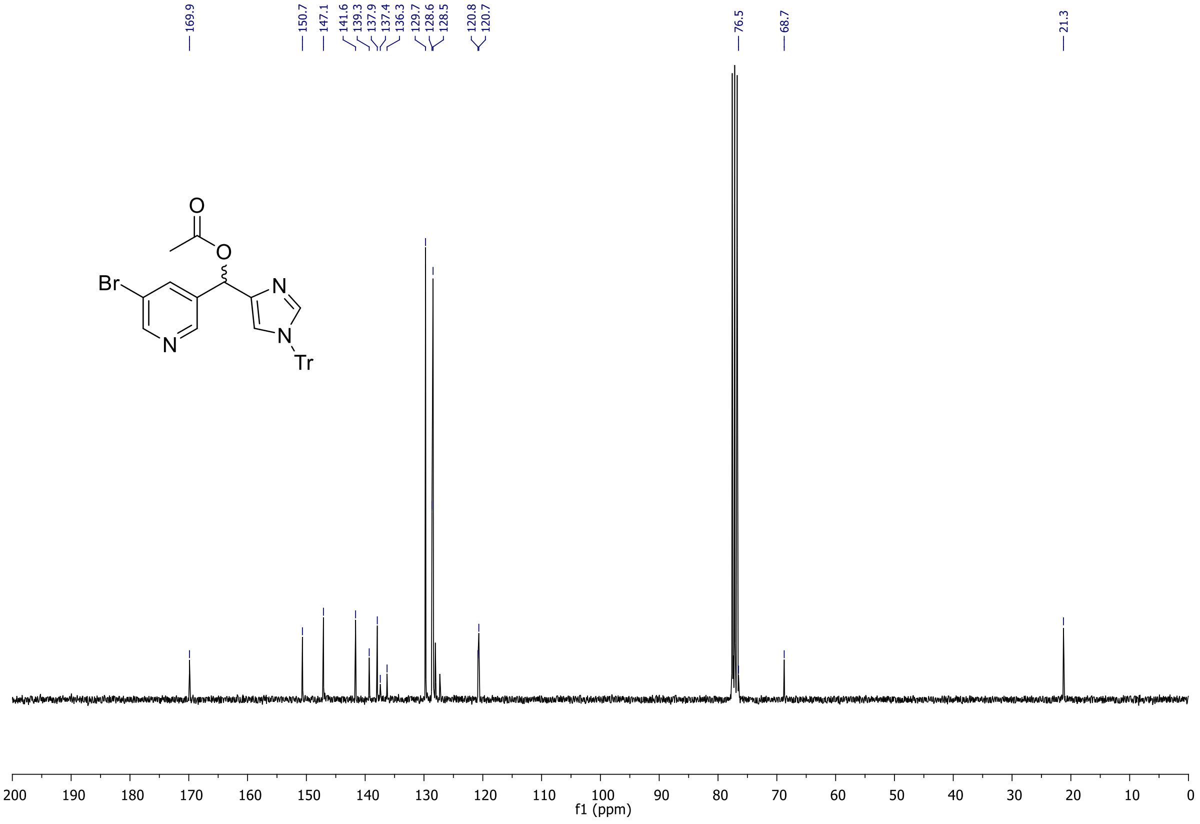

3-Bromo-5-((1-trityl-1*H*-imidazol-4-yl)methyl)pyridine (**21**)

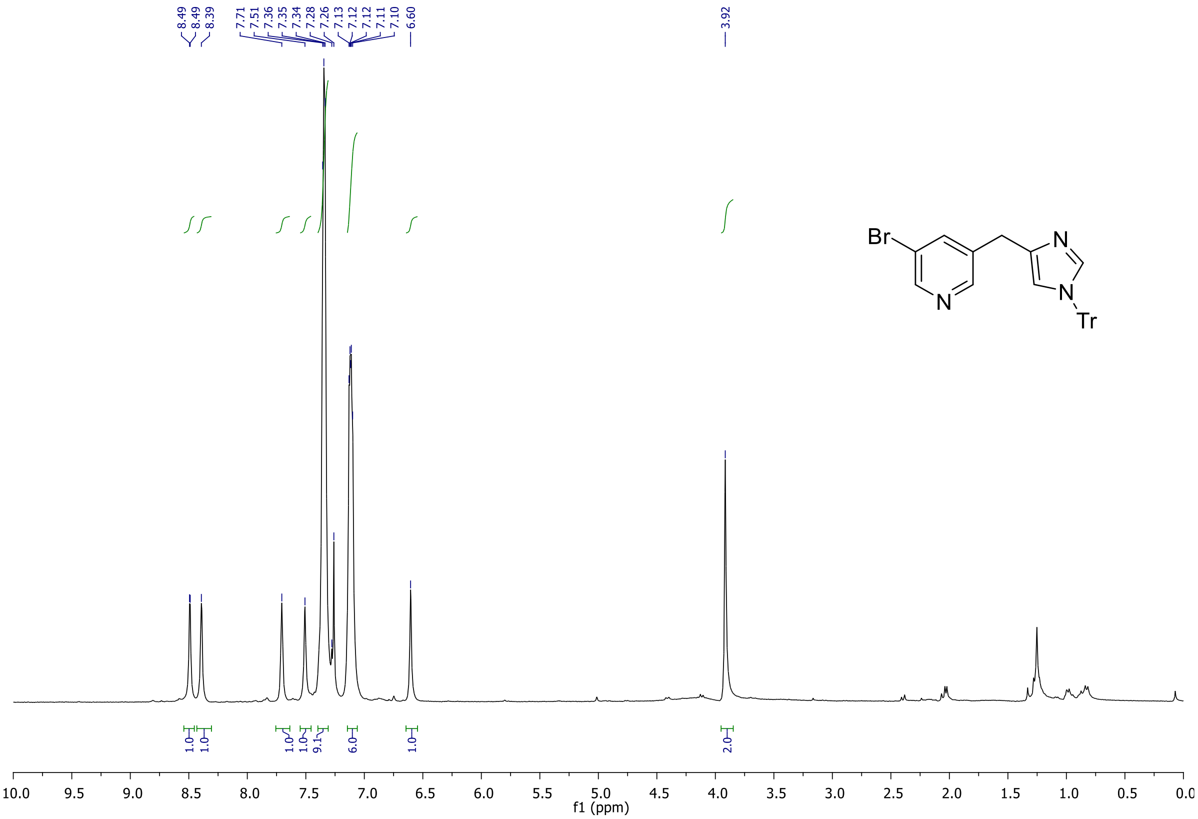

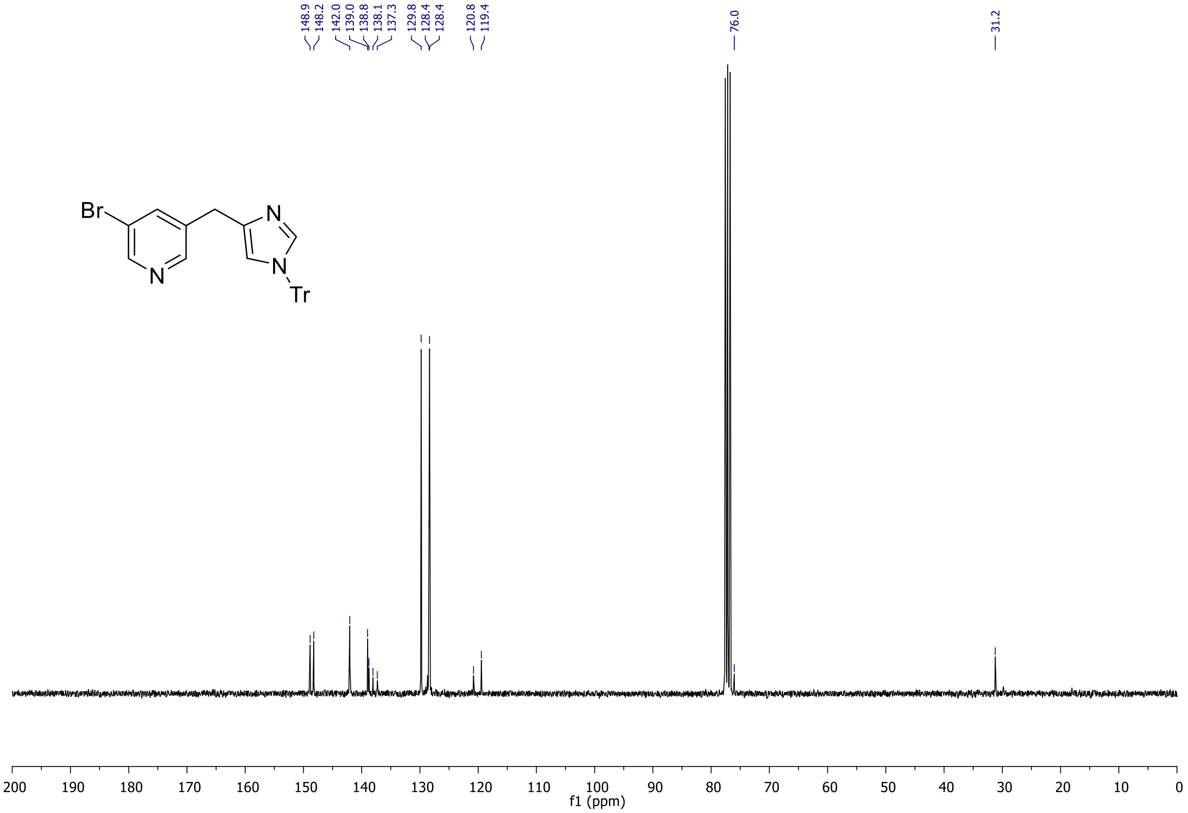

(5-(4,4,5,5-Tetramethyl-1,3,2-dioxaborolan-2-yl)pyridin-3-yl)(1-trityl-1*H*-imidazol-4-yl)methyl acetate (**22**)

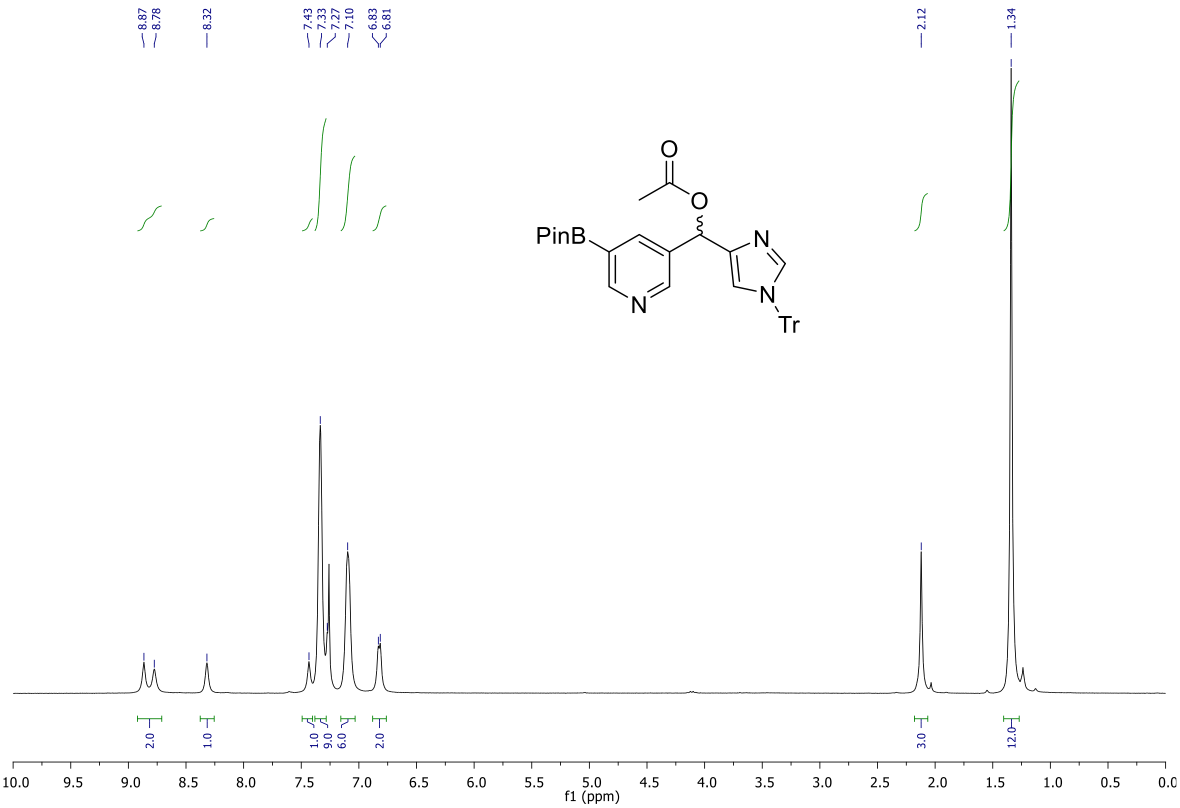

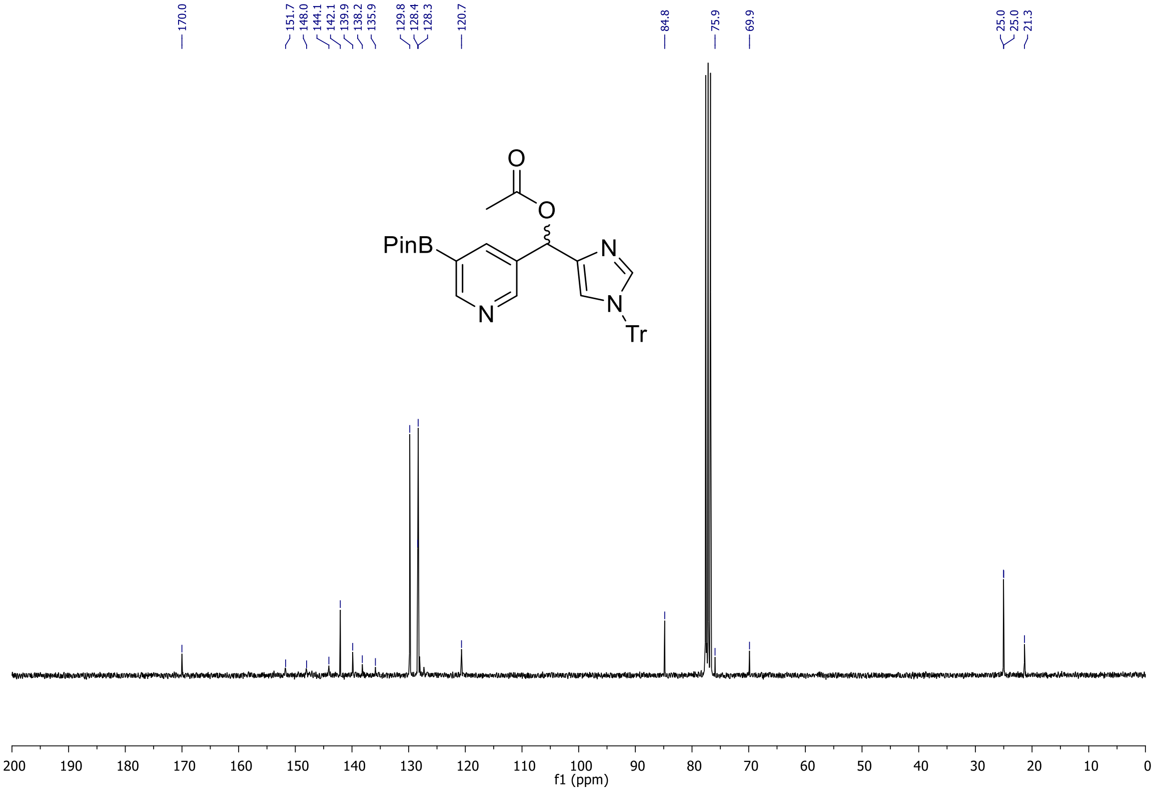

3-(4,4,5,5-Tetramethyl-1,3,2-dioxaborolan-2-yl)-5-((1-trityl-1*H*-imidazol-4-yl)methyl)pyridine (**23**)

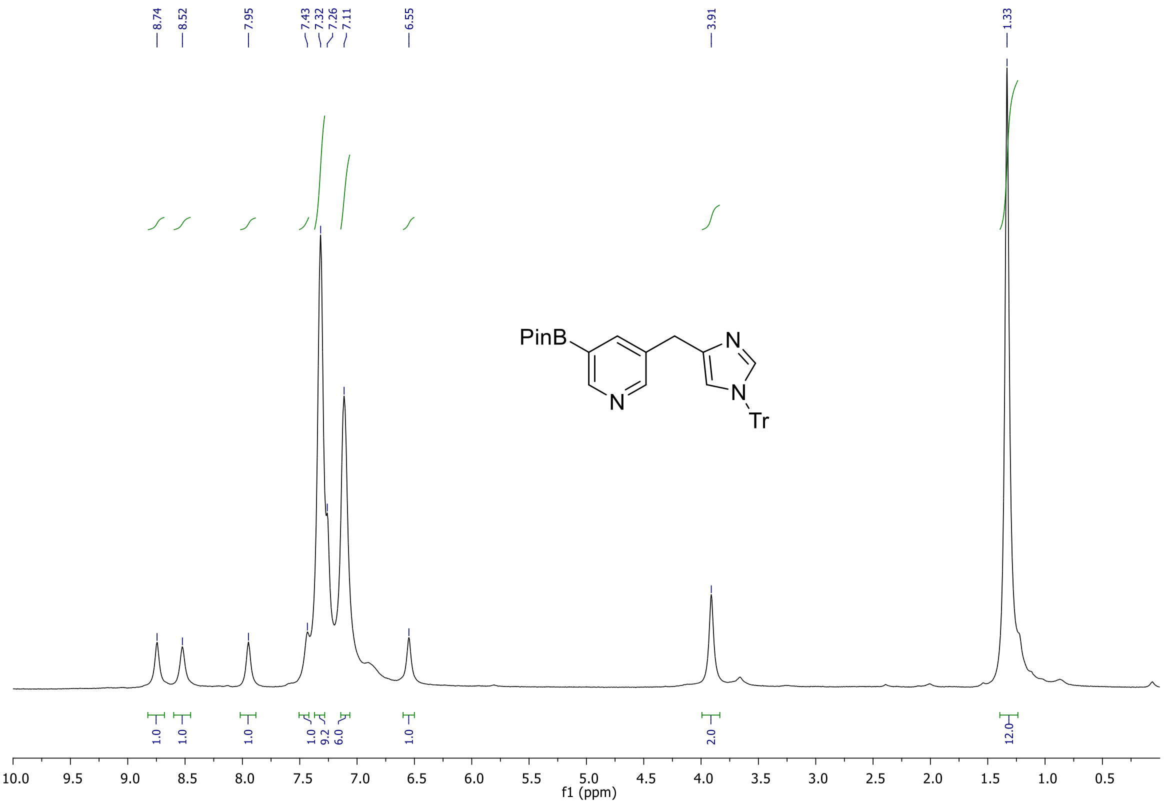

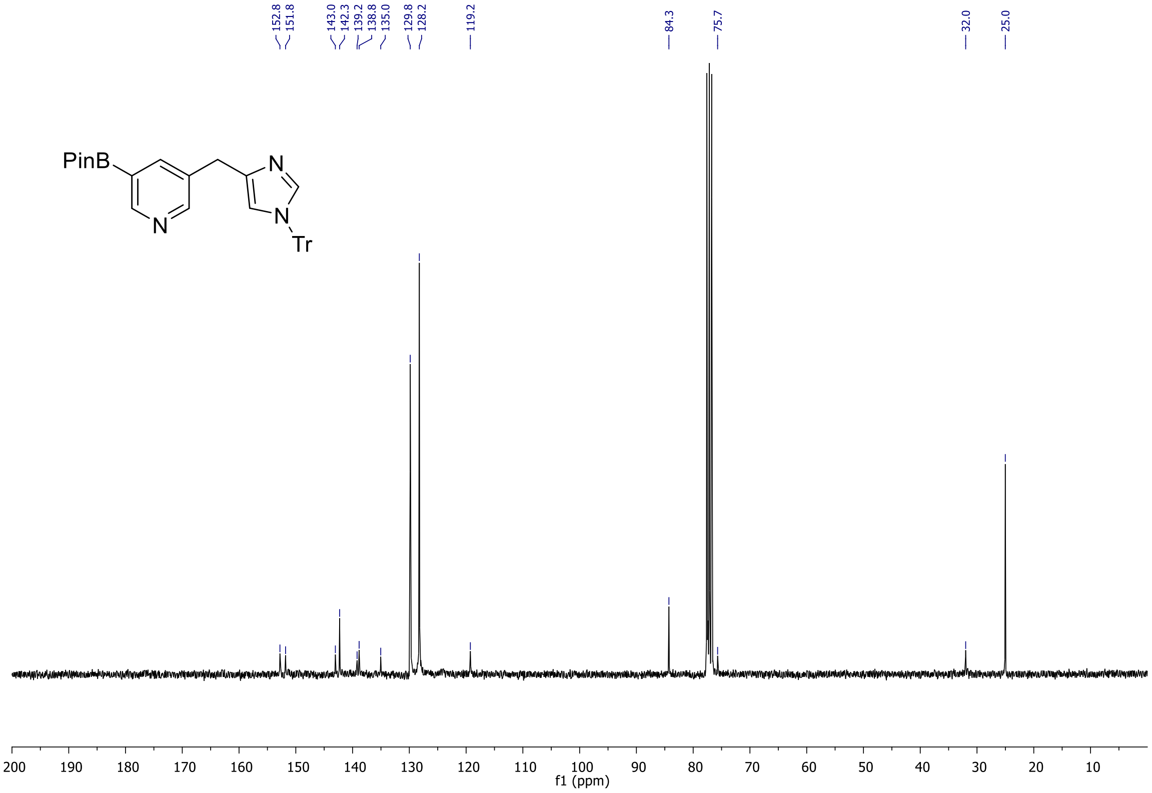

3,5-Diiodopyridine (**2**)

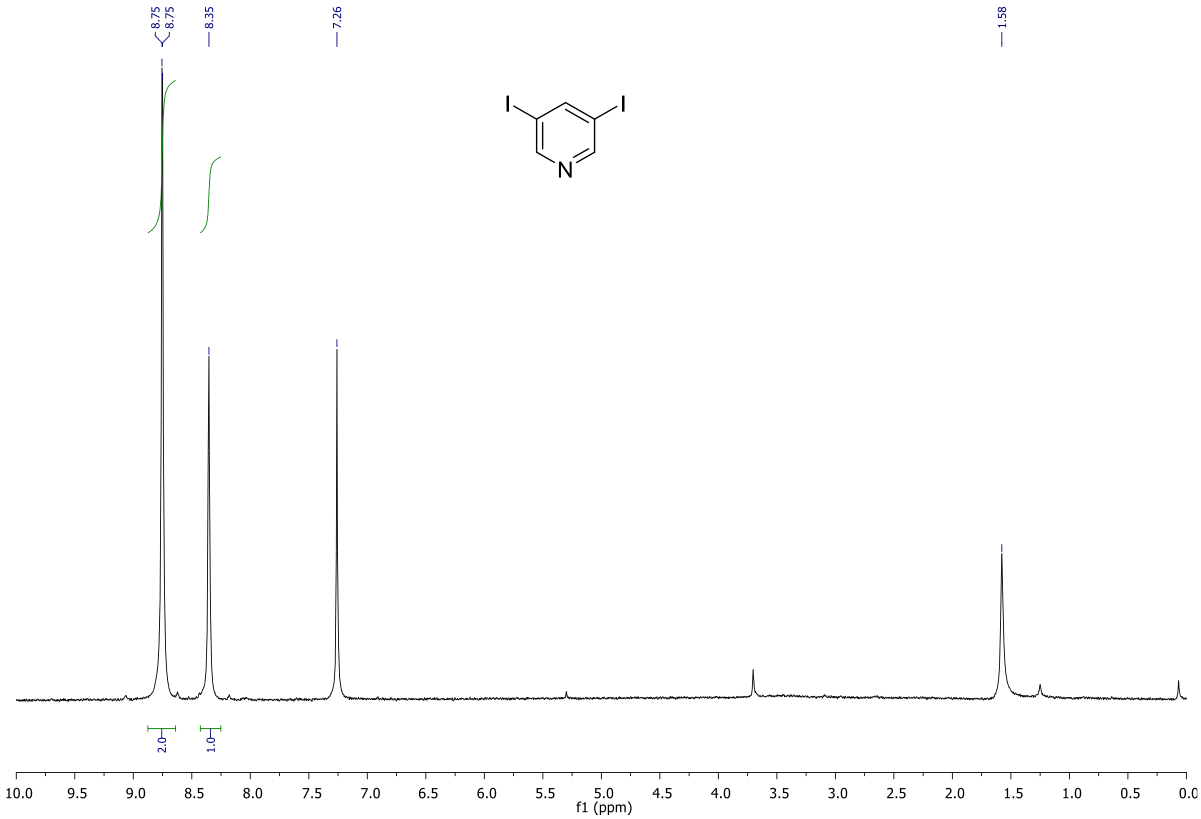

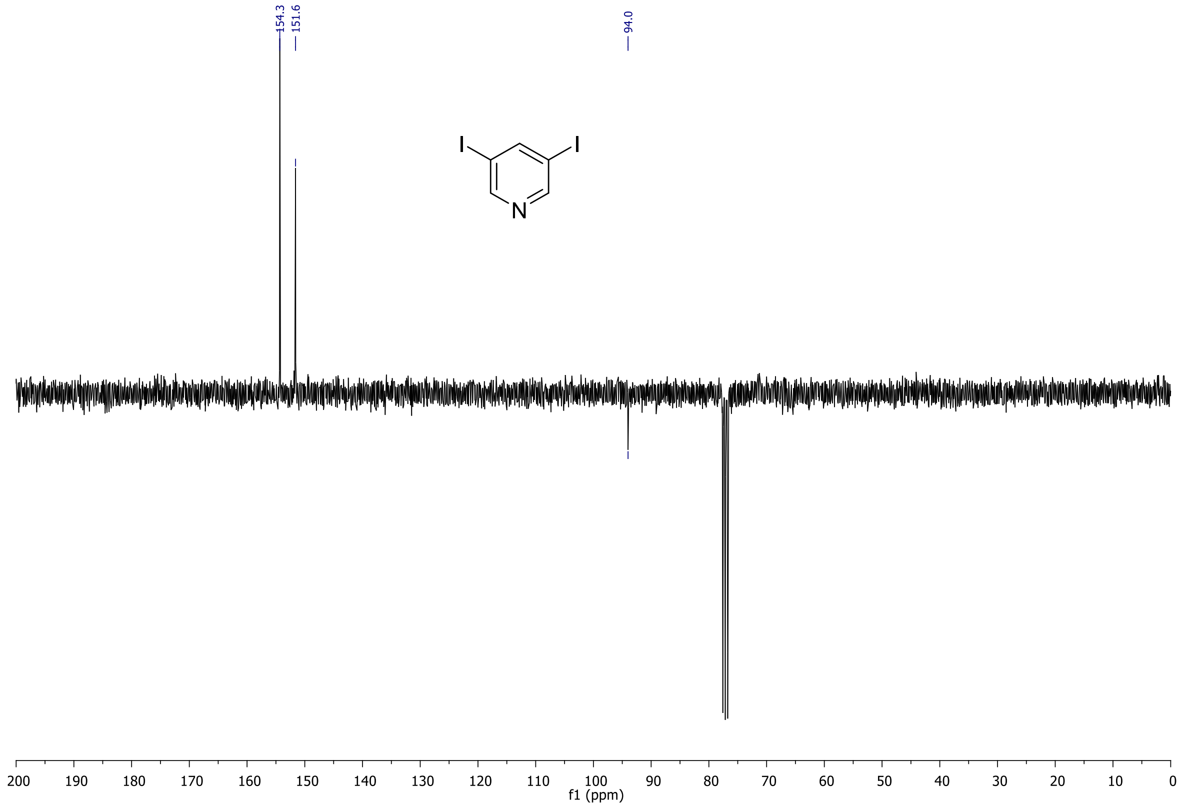

(4-((*tert*-Butyldimethylsilyl)oxy)phenyl)(5-iodopyridin-3-yl)methanol (**25a**)

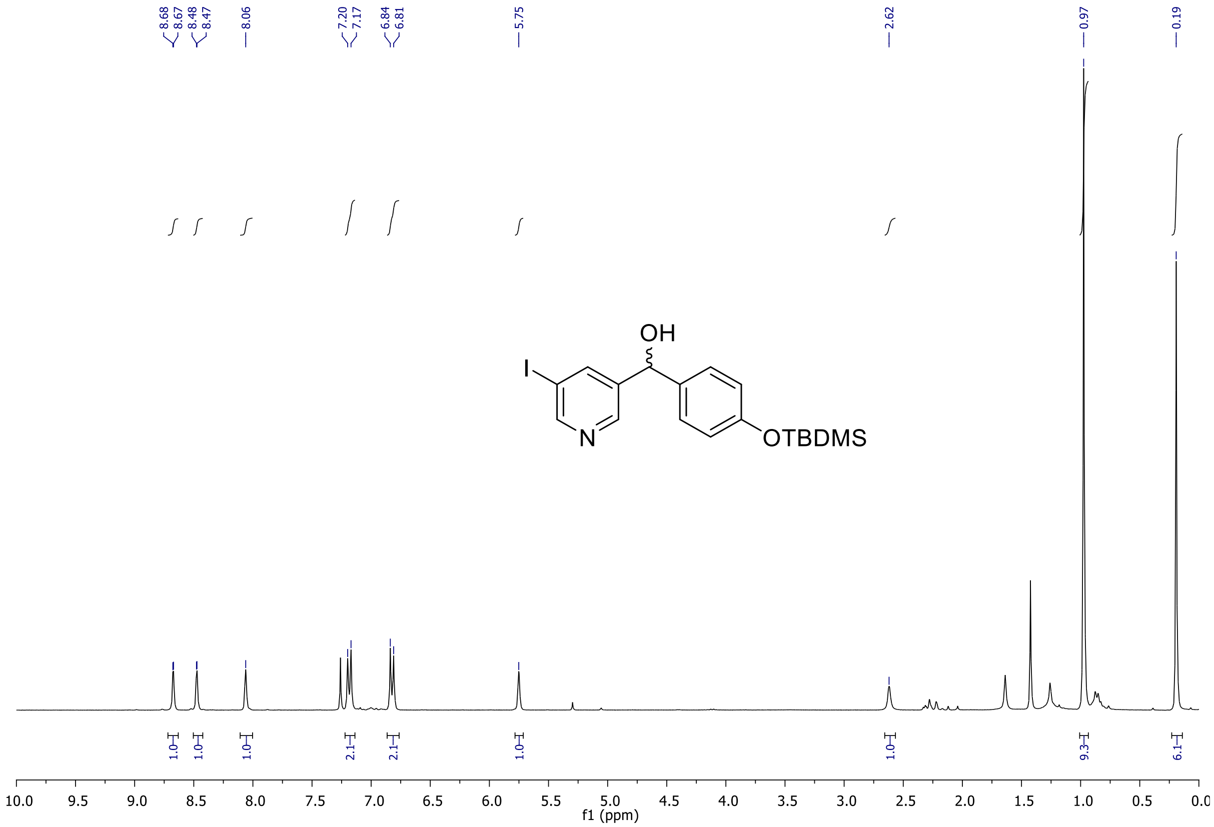

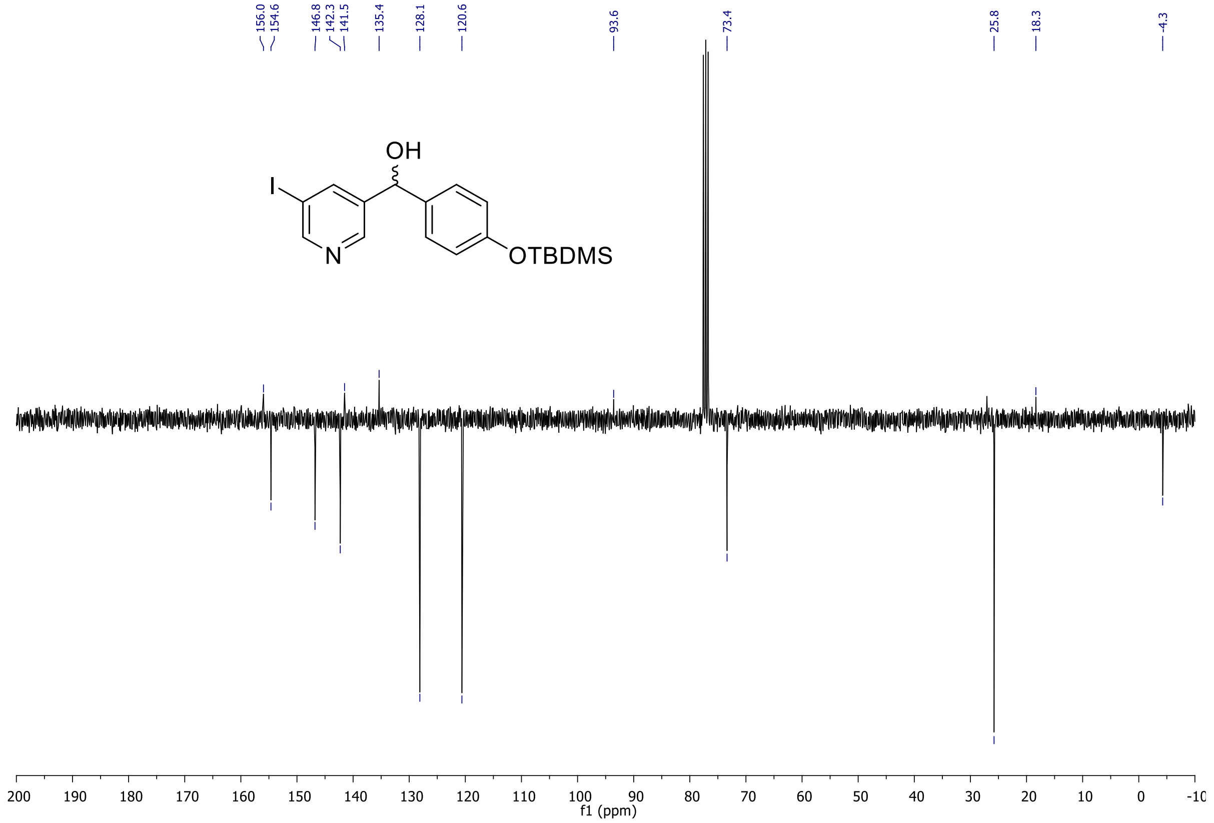

3-(4-((*tert*-Butyldimethylsilyl)oxy)benzyl)-5-iodopyridine (**25**)

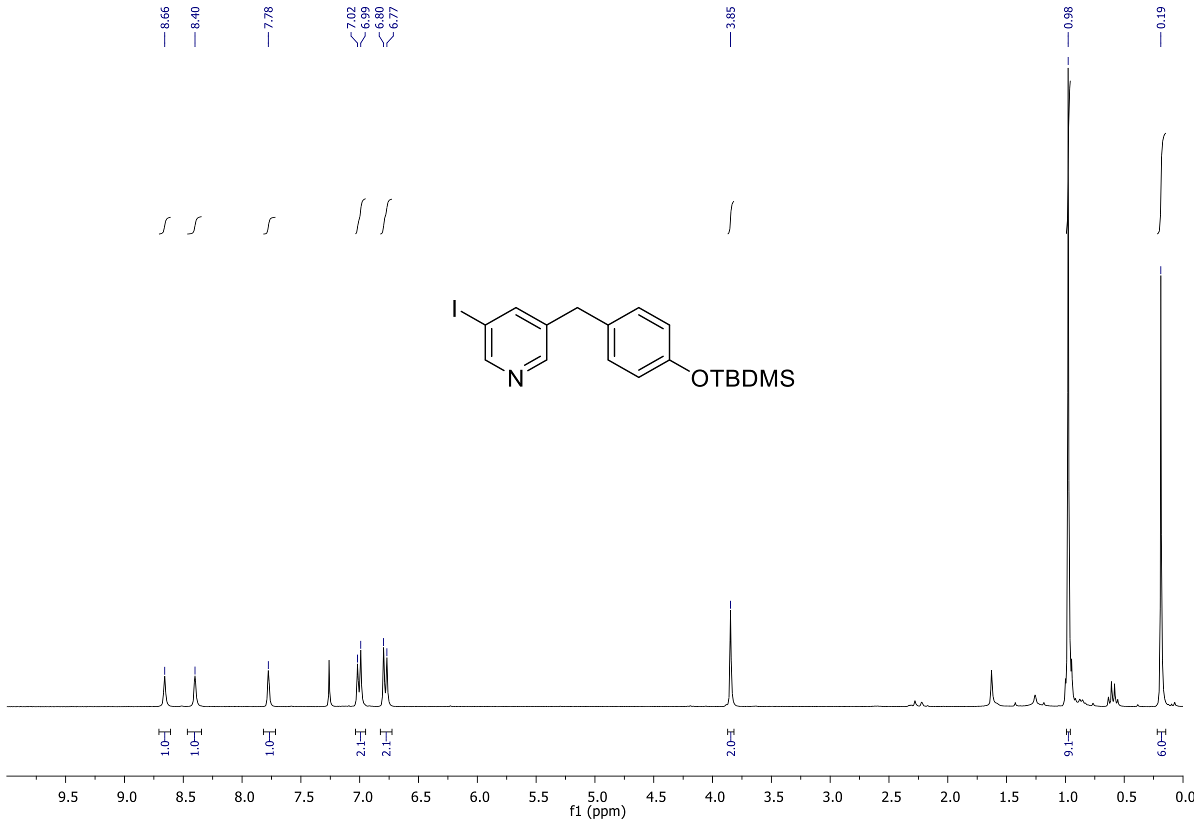

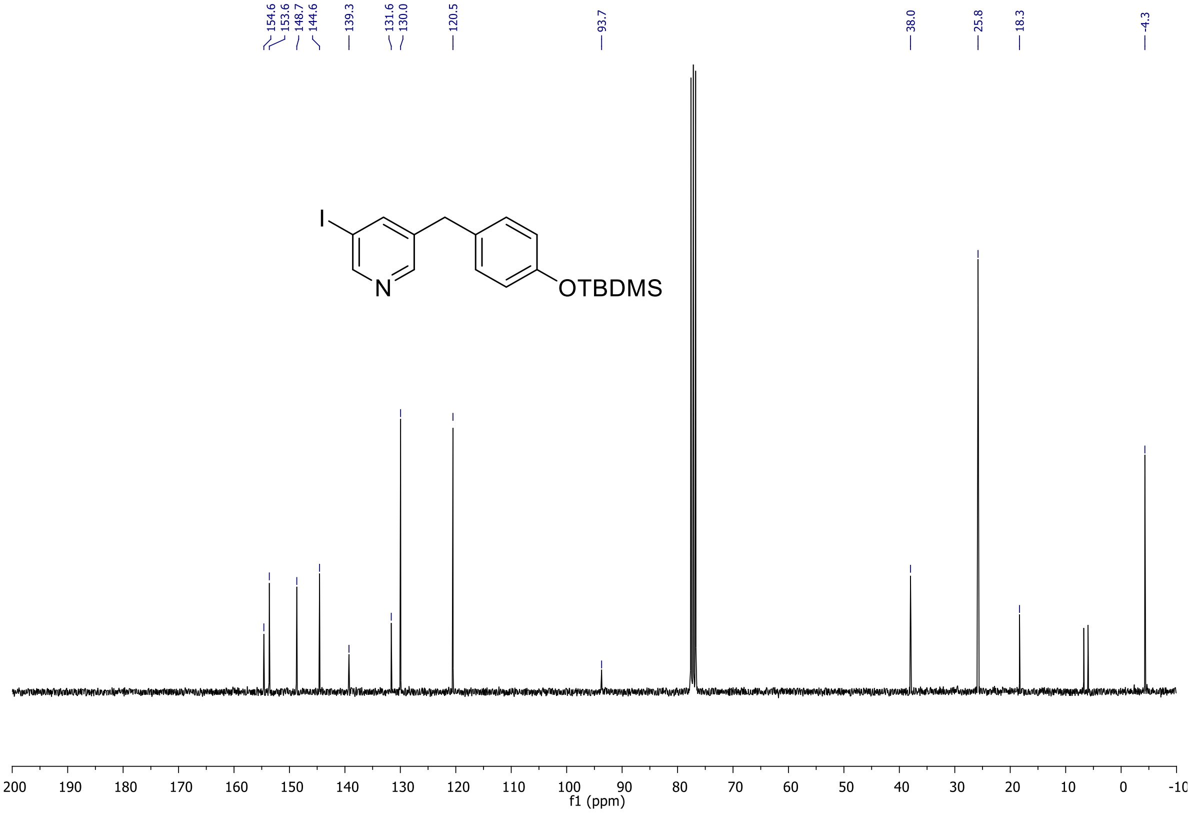

3-(4-((*tert*-Butyldimethylsilyl)oxy)benzyl)-5-(4,4,5,5-tetramethyl-1,3,2-dioxaborolan-2-yl)pyridine (**26**)

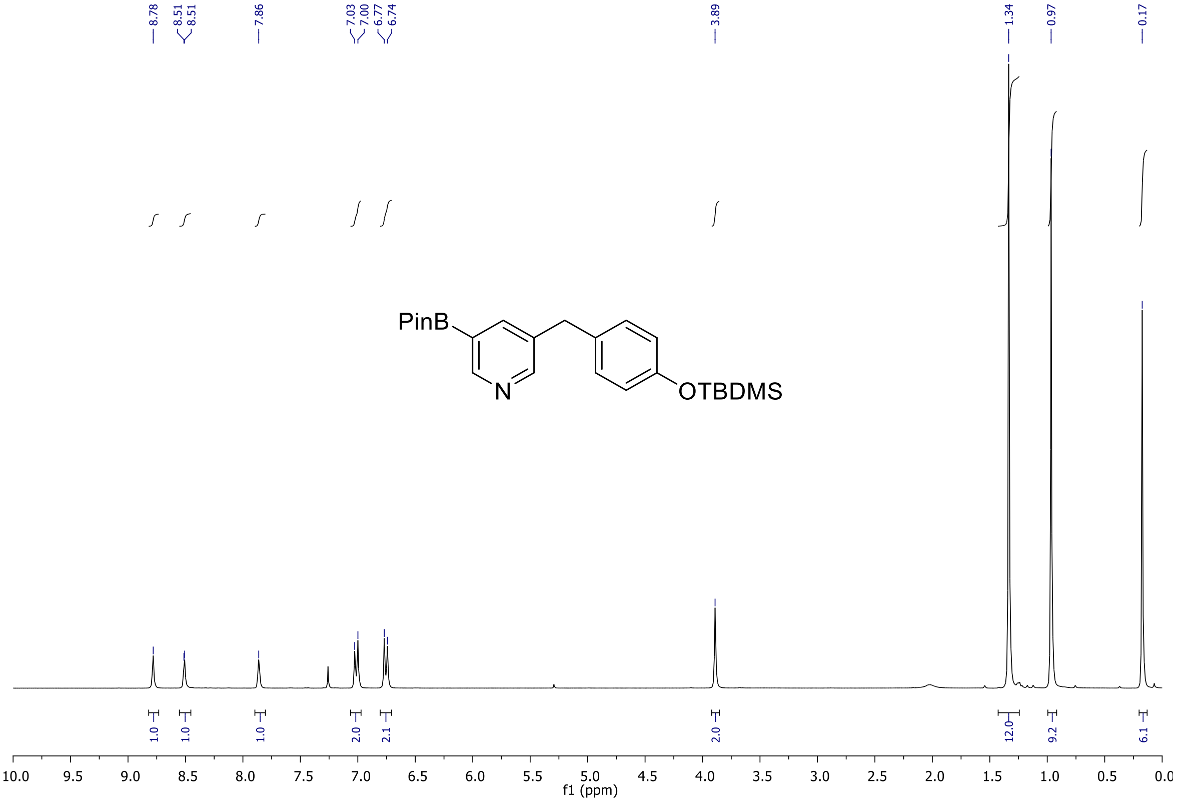

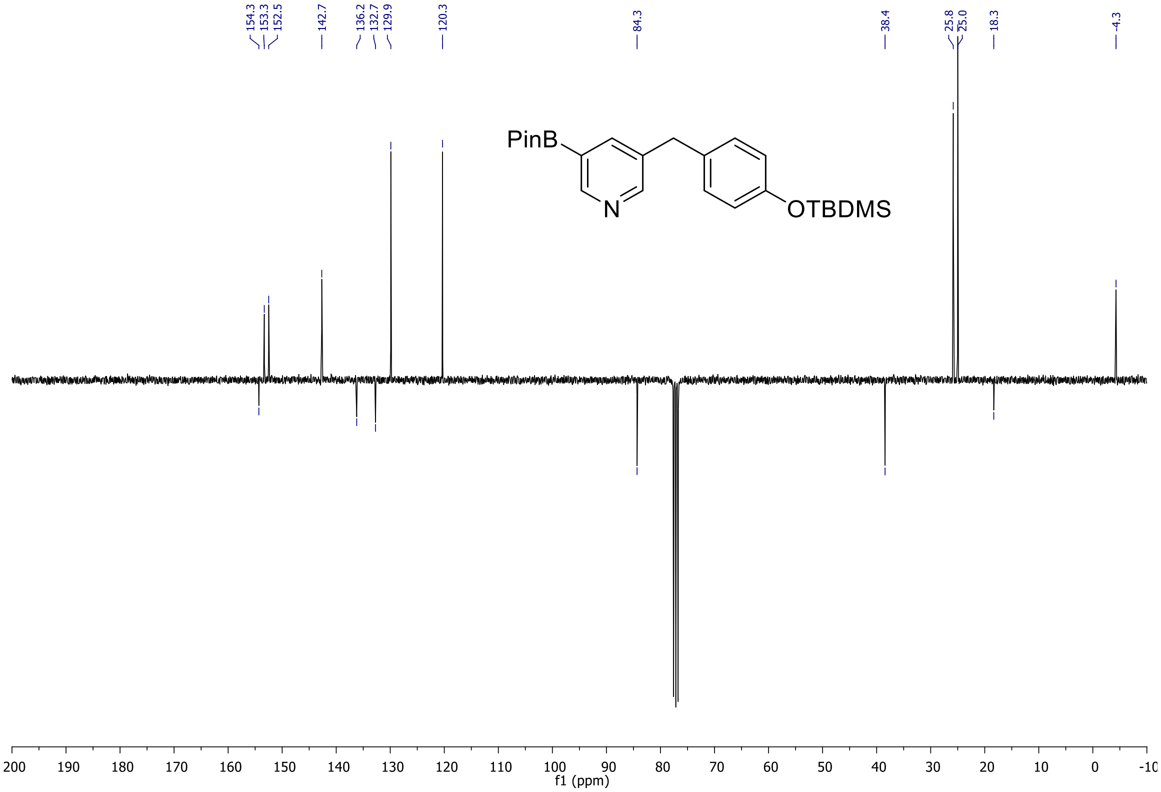

4-Hydroxy-4-(5-iodopyridin-3-yl)butanenitrile (**28a**)

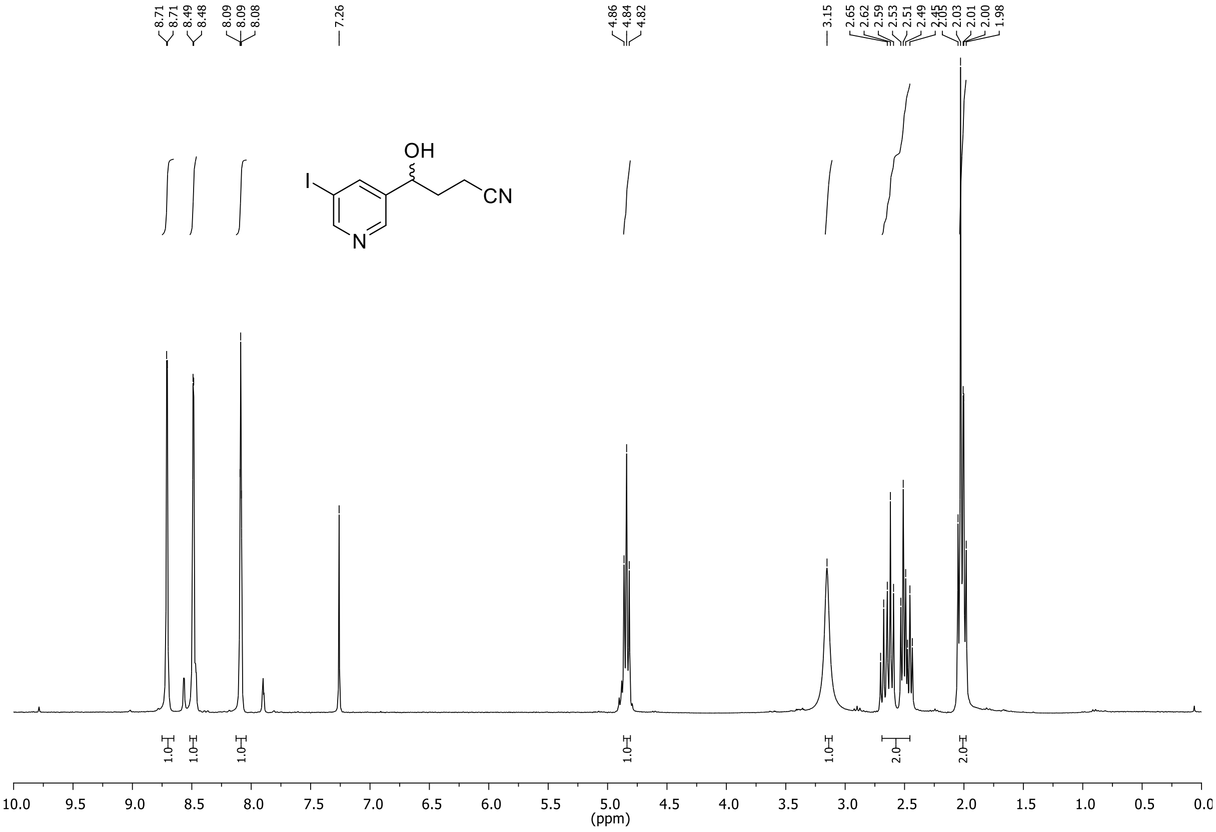

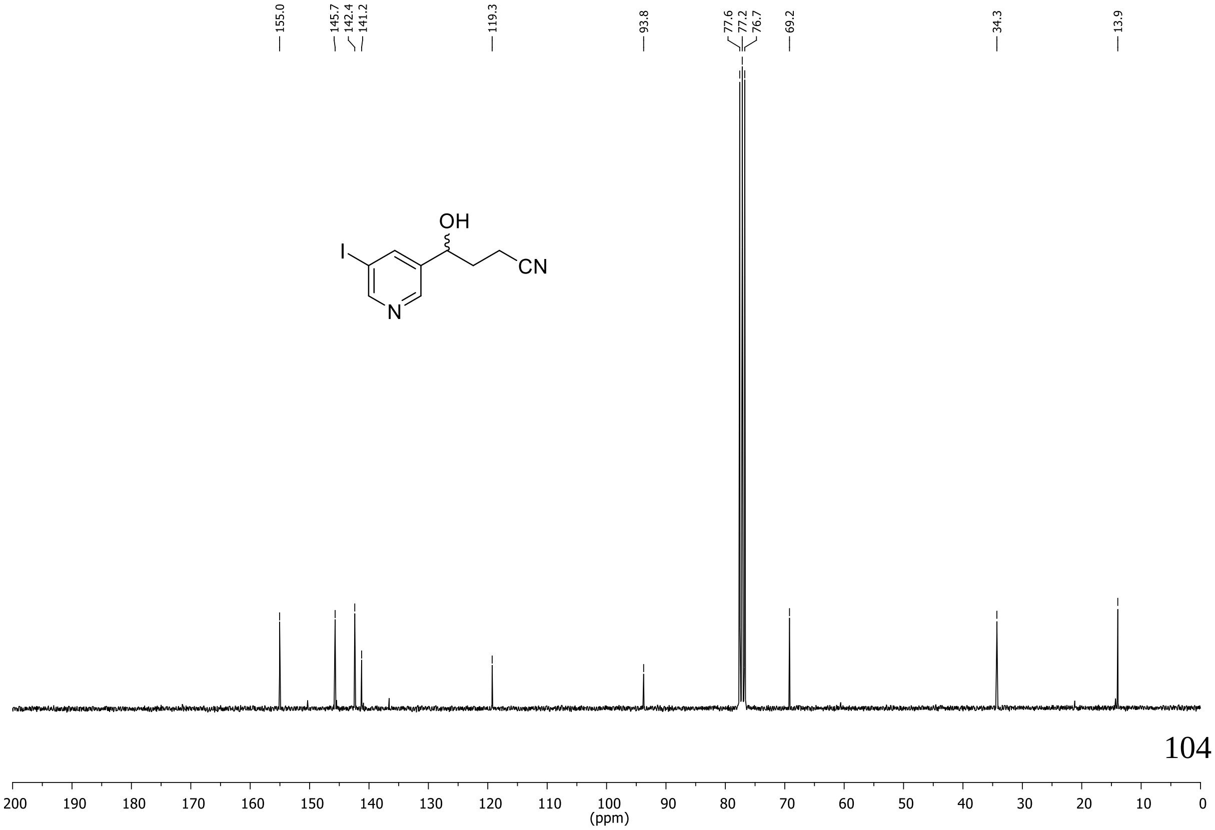

4-Chloro-4-(5-(4,4,5,5-tetramethyl-1,3,2-dioxaborolan-2-yl)pyridin-3-yl)butanenitrile **(28)**

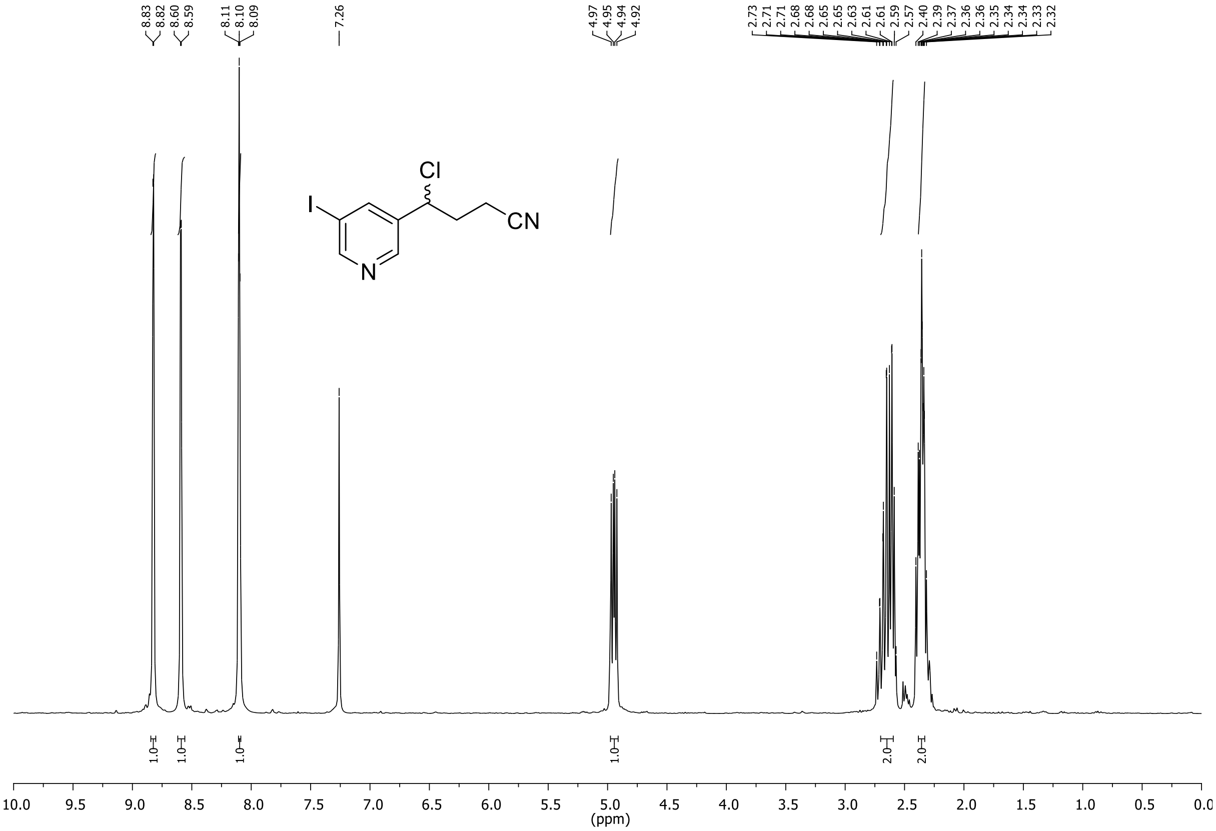

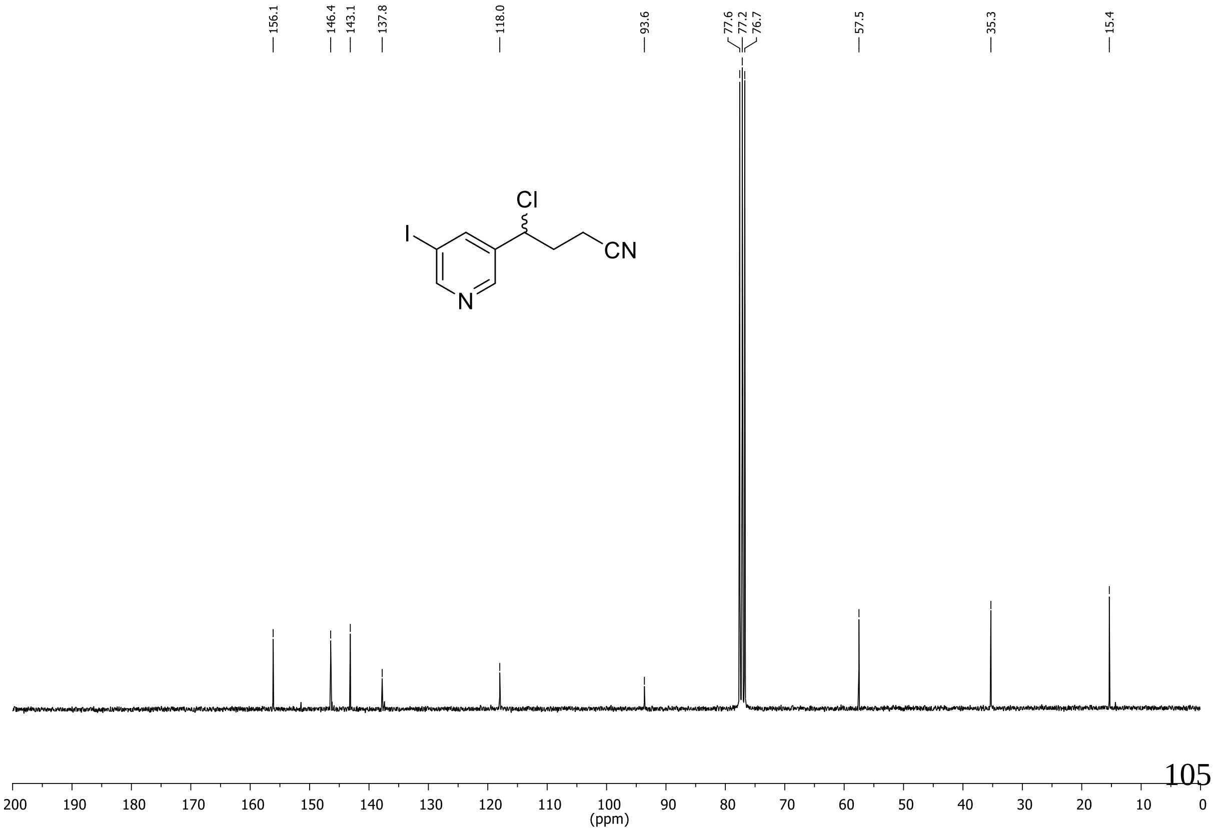

4-(5-(4,4,5,5-Tetramethyl-1,3,2-dioxaborolan-2-yl)pyridin-3-yl)butanenitrile (**29**)

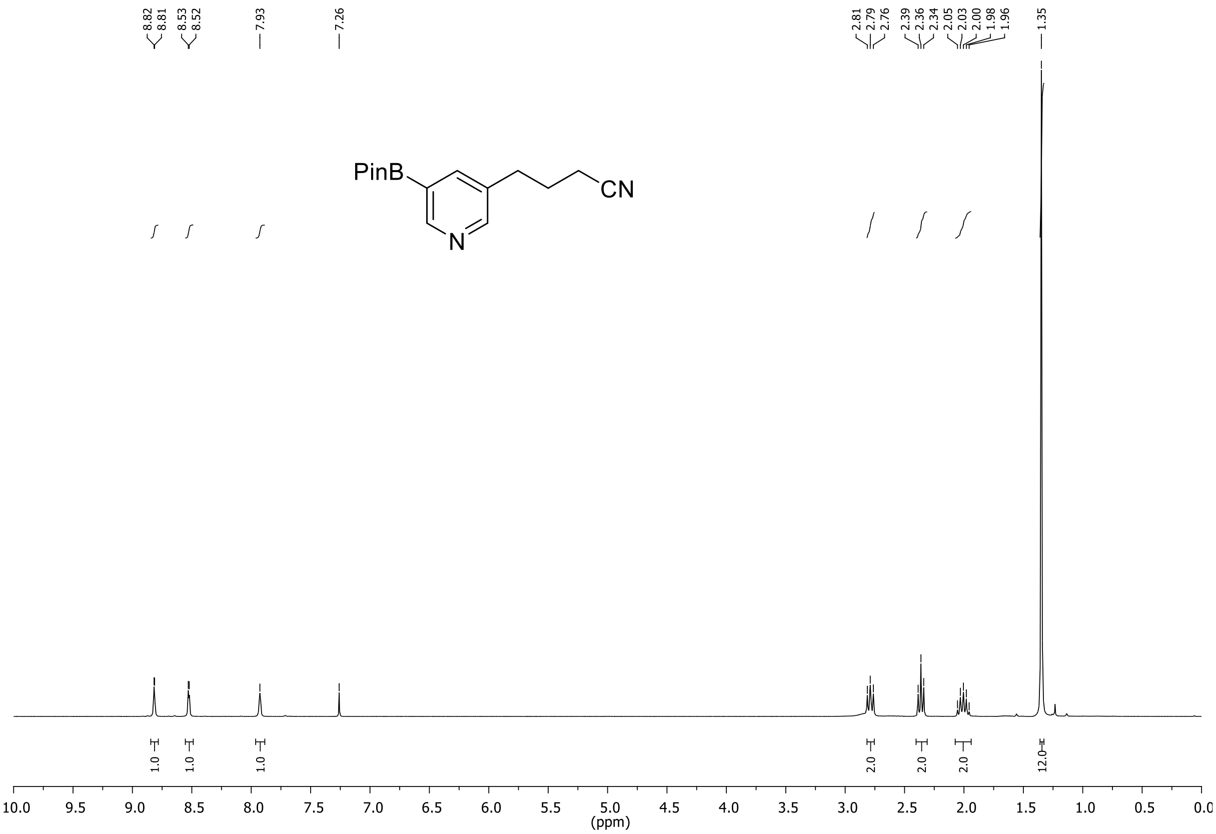

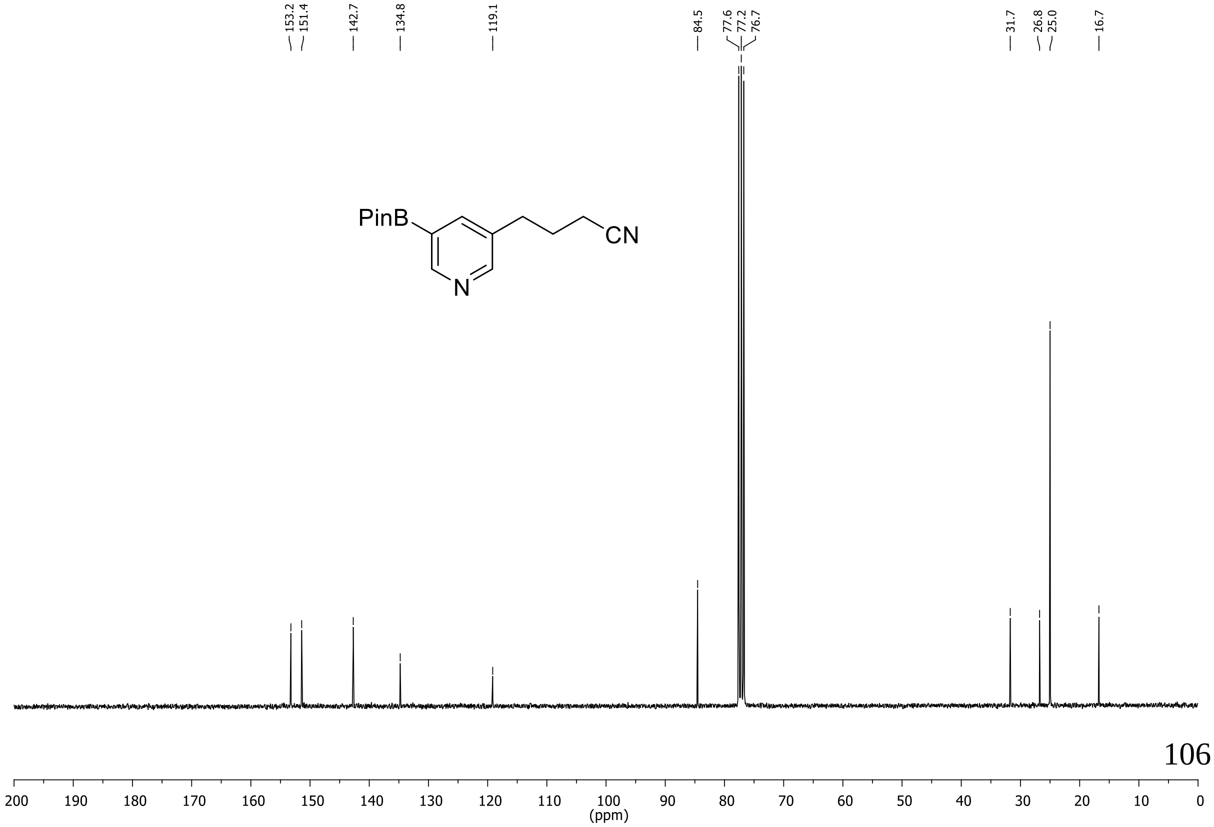

5-Iodonicotinaldehyde **(3)**

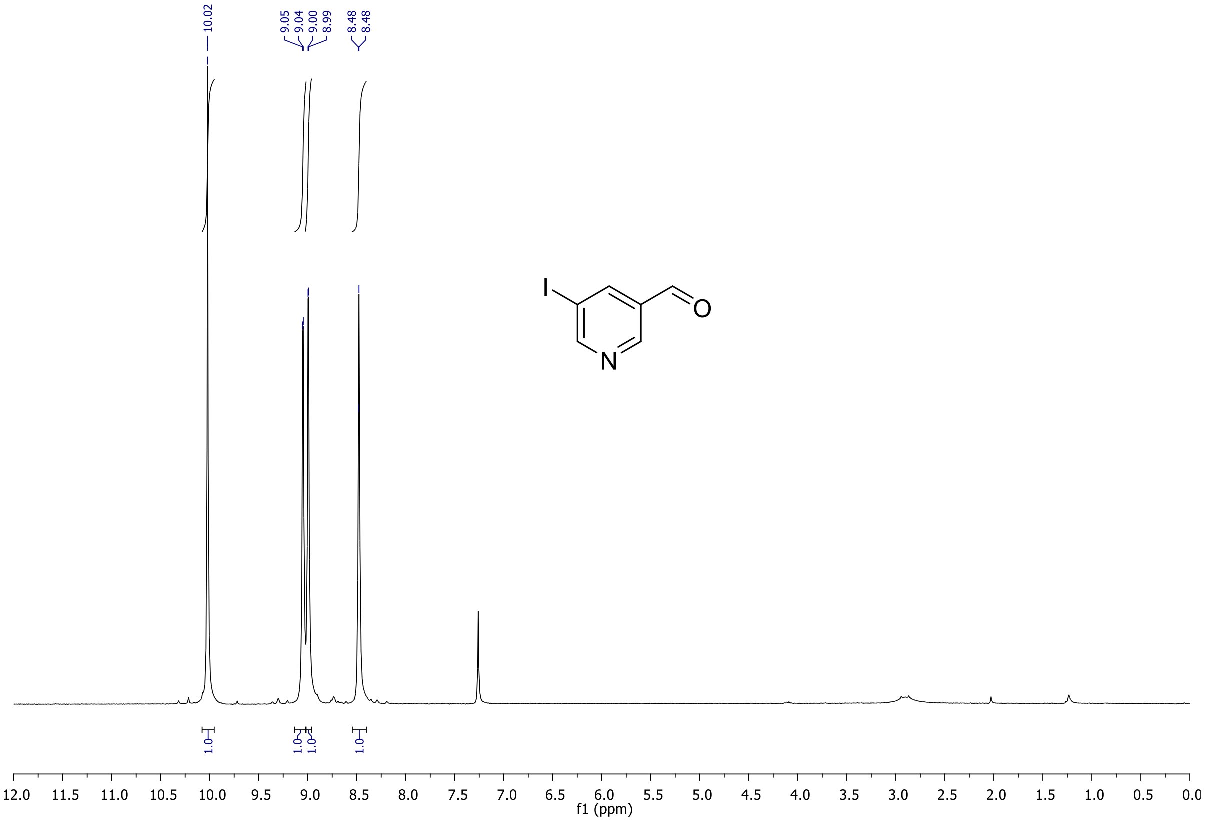

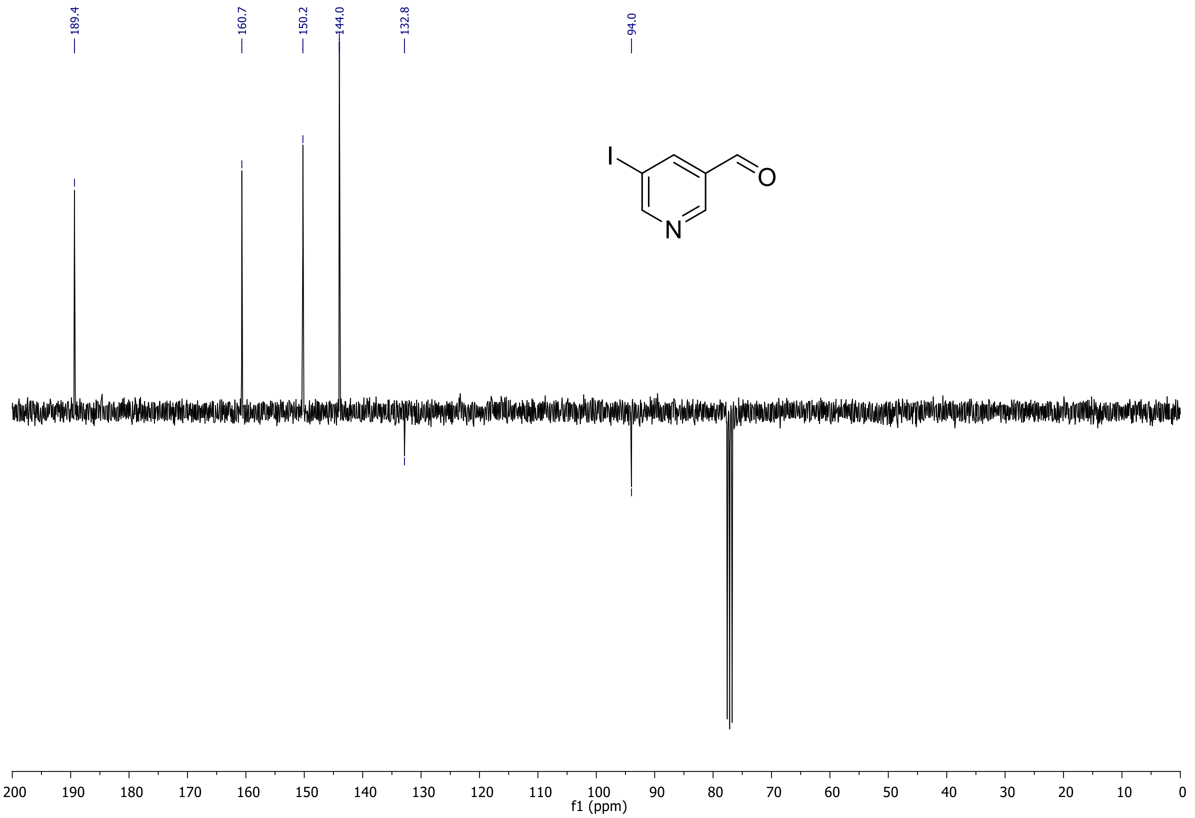

1-(5-Iodopyridin-3-yl)ethan-1-ol (**30**)

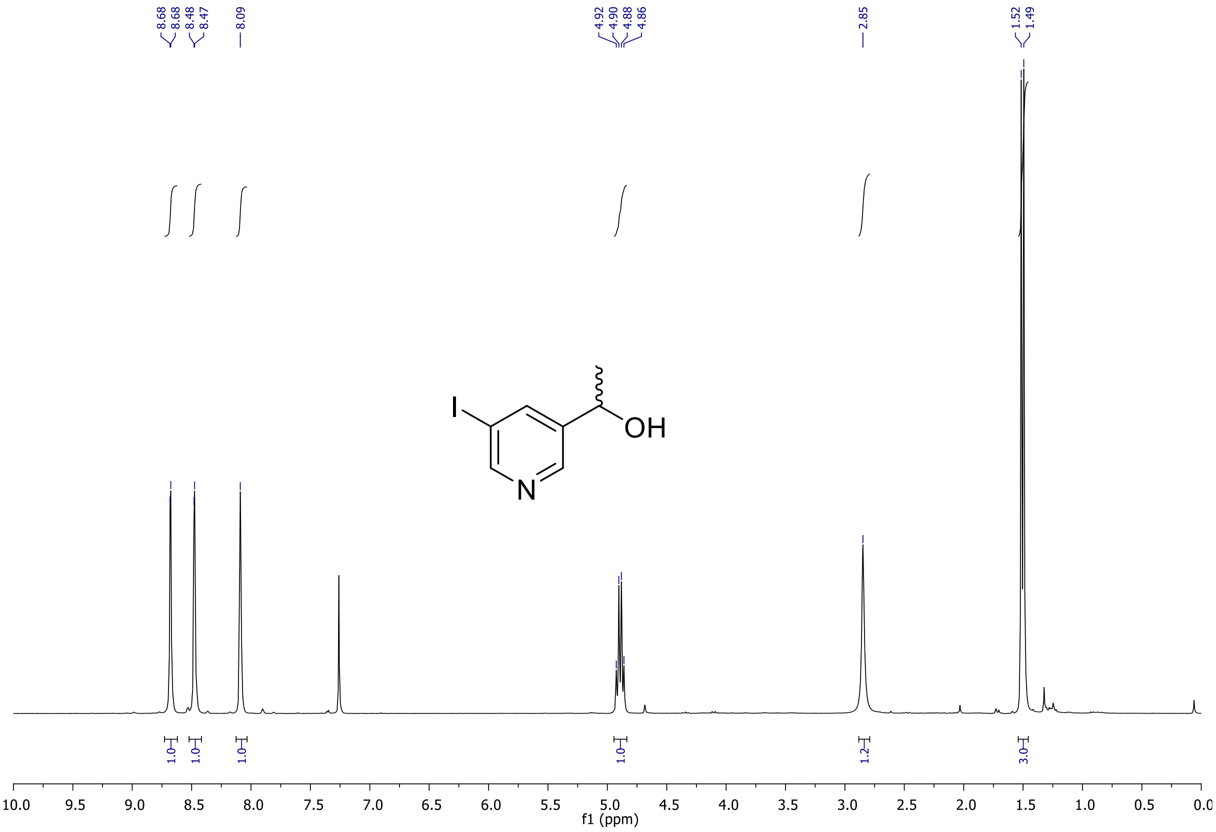

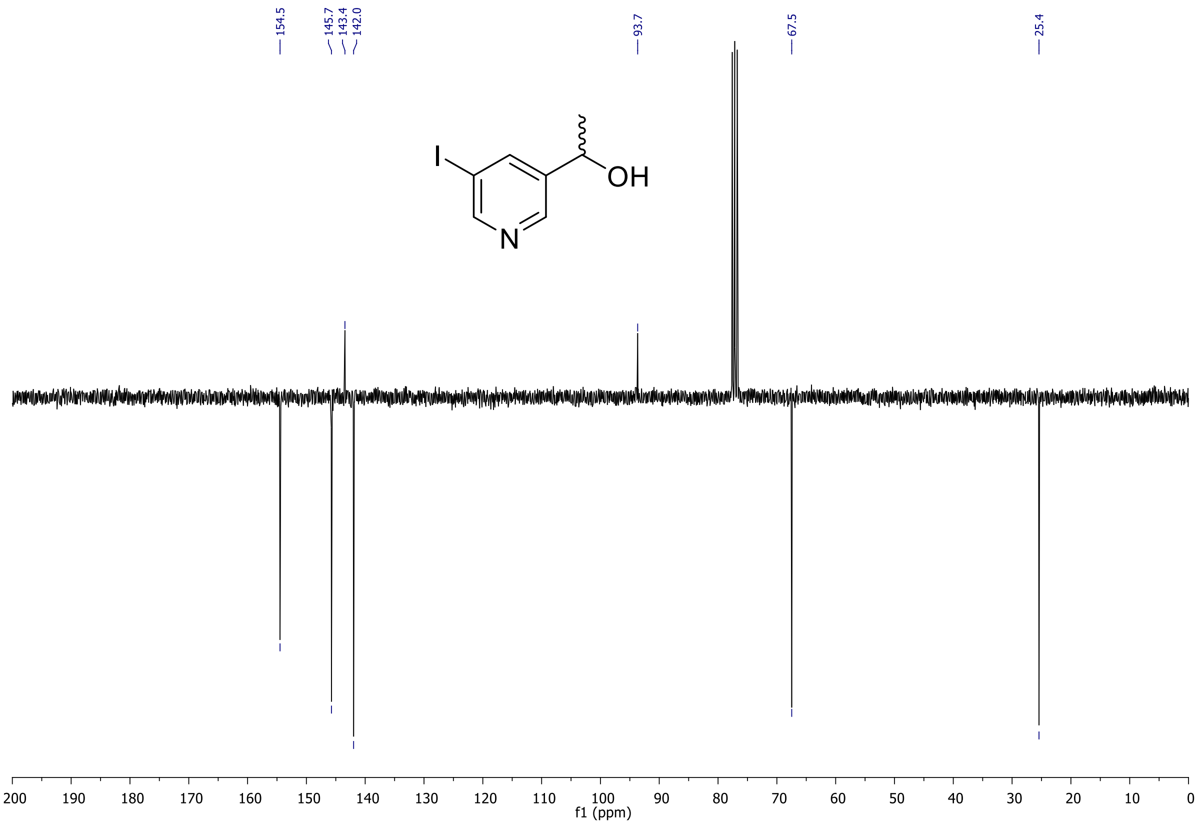

3-(1-((*tert*-Butyldiphenylsilyl)oxy)ethyl)-5-iodopyridine **(31a)**

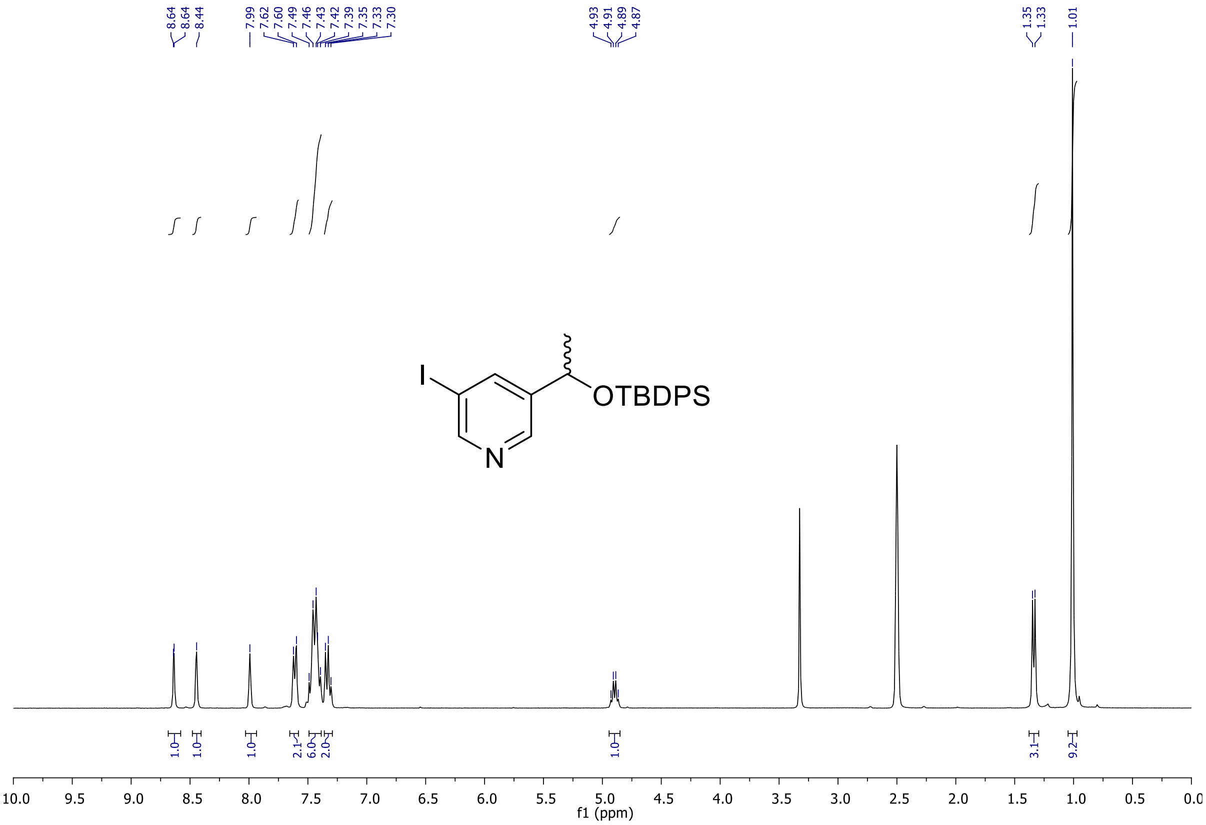

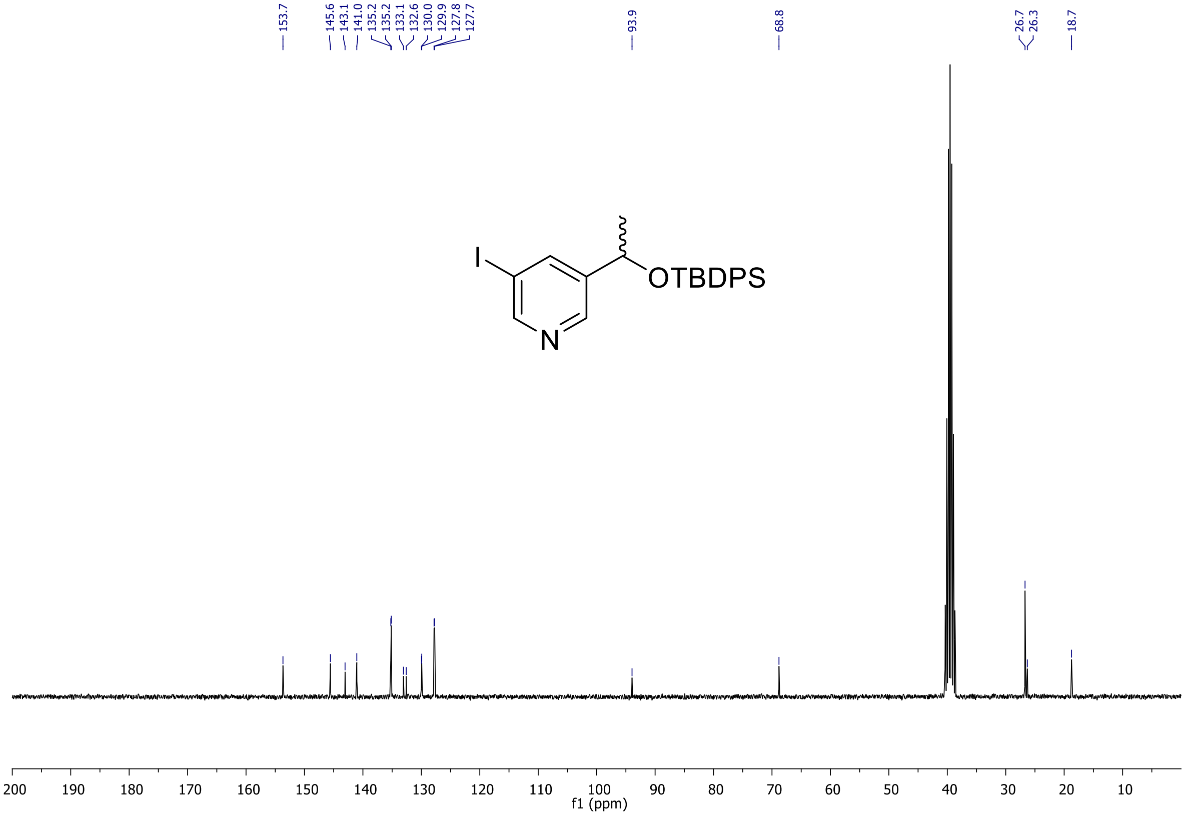

3-(1-((*tert*-Butyldiphenylsilyl)oxy)ethyl)-5-(4,4,5,5-tetramethyl-1,3,2-dioxaborolan-2-yl)pyridine (**31**)

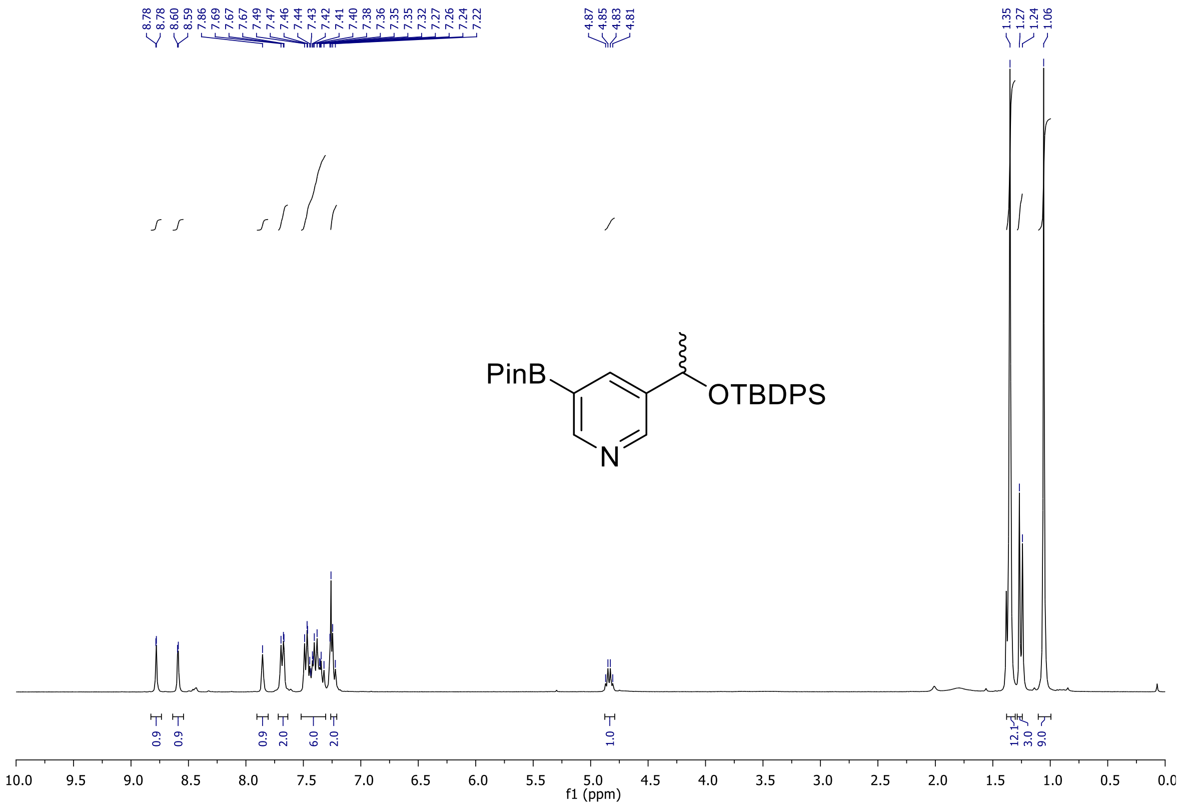

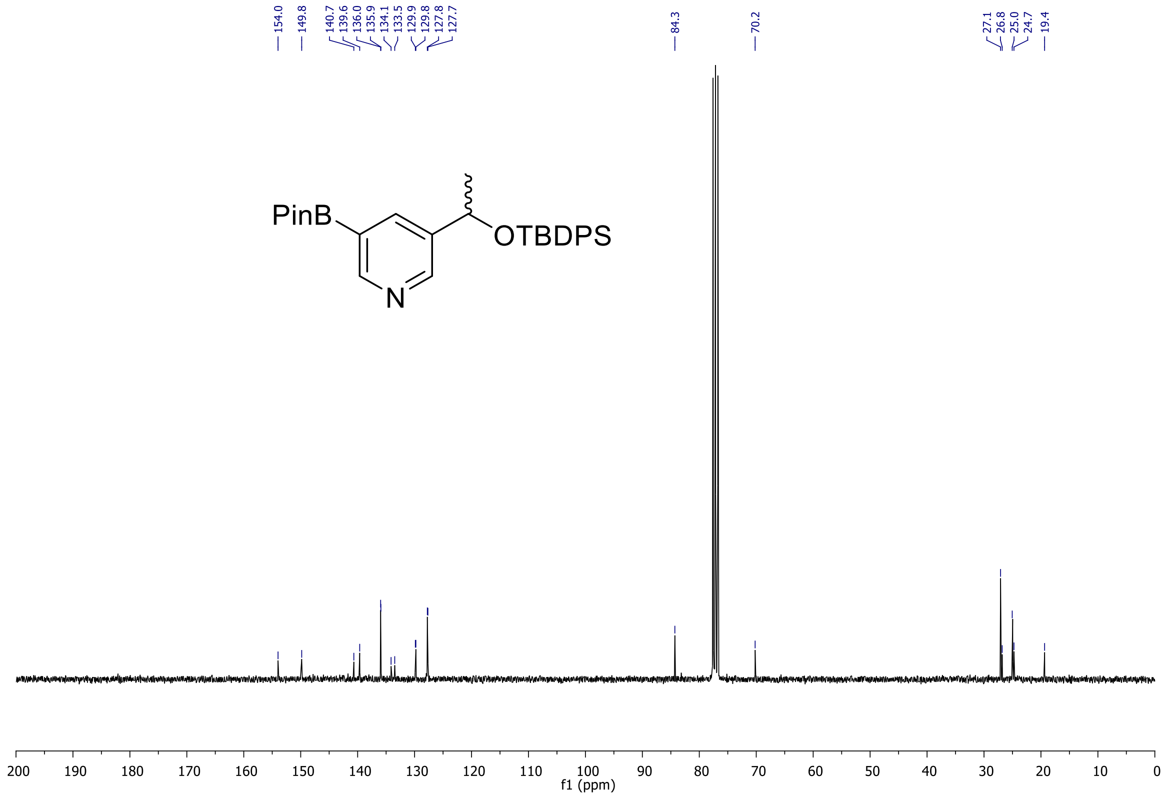

((Methylthio)methyl)triphenylphosphonium chloride (**32**)

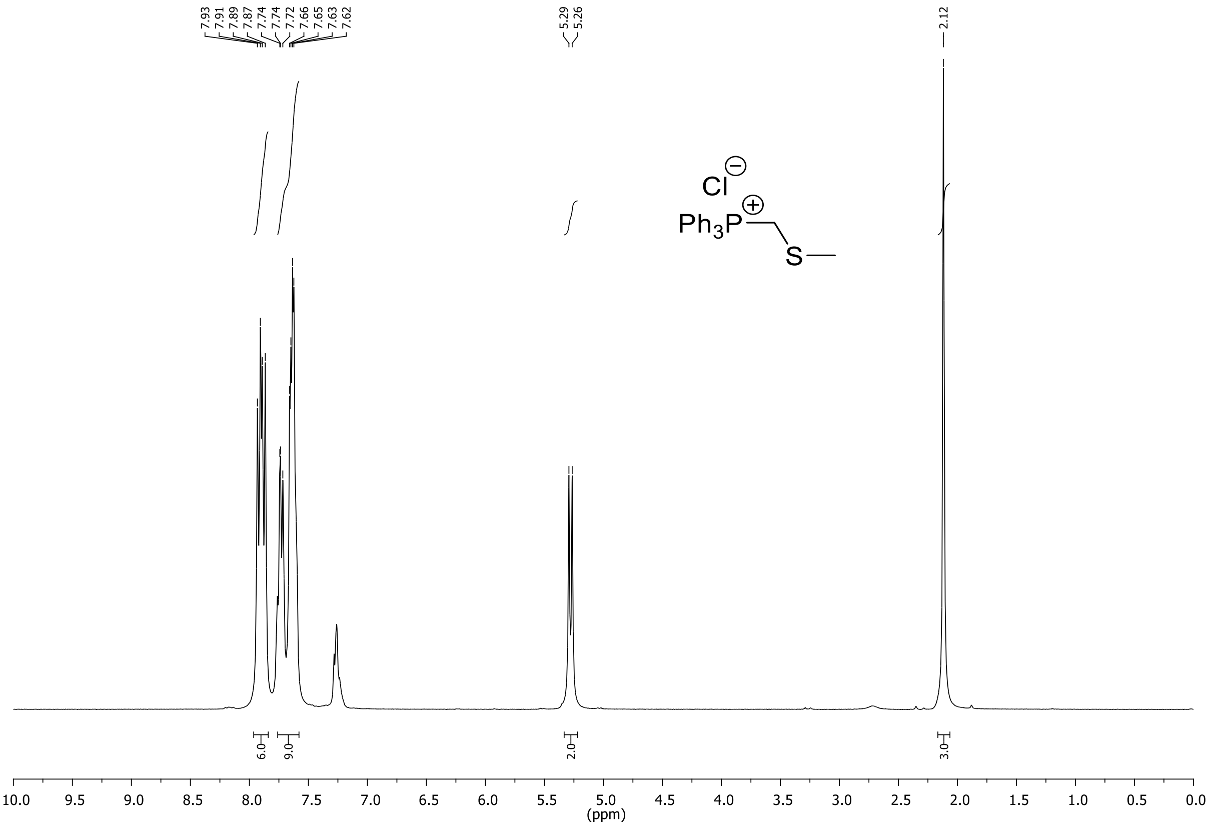

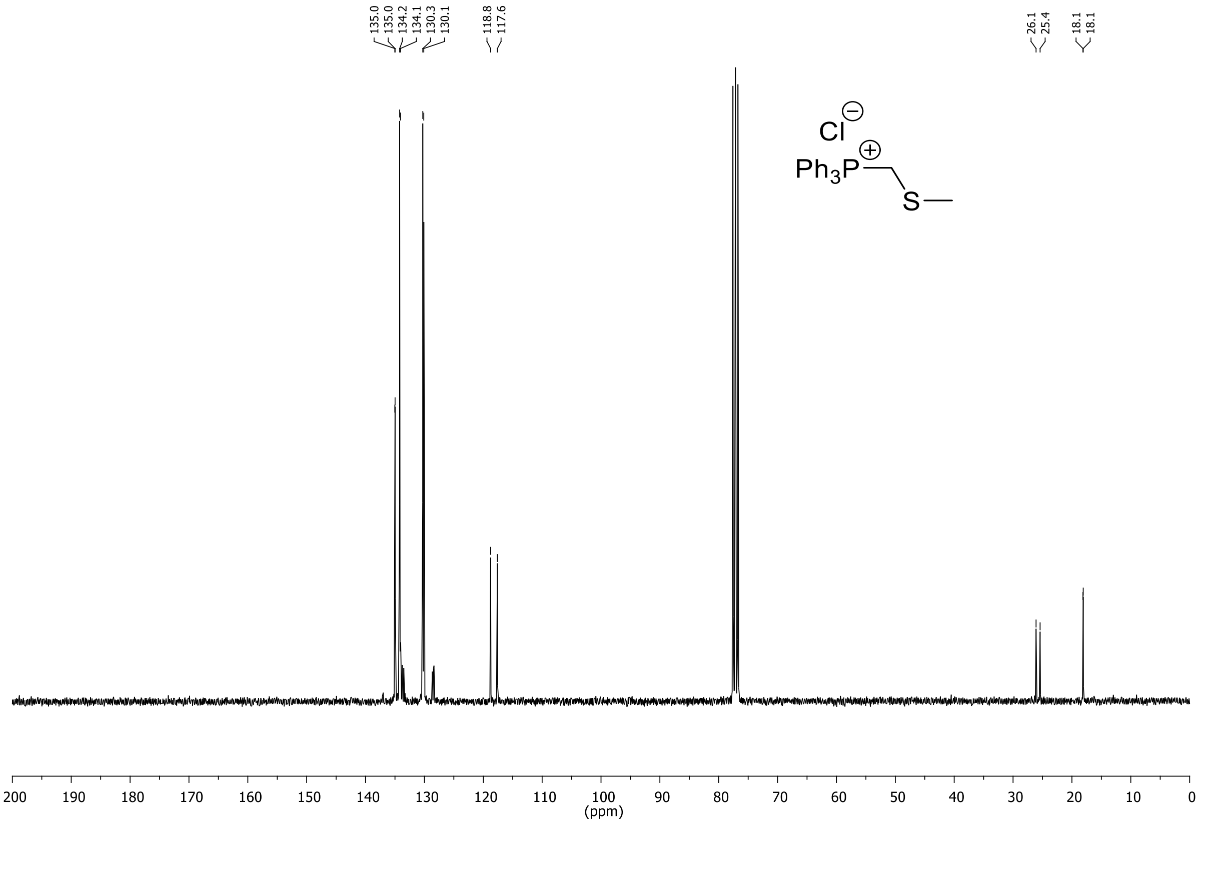

(E,Z)-3-Iodo-5-(2-(methylthio)vinyl)pyridine **(33)**

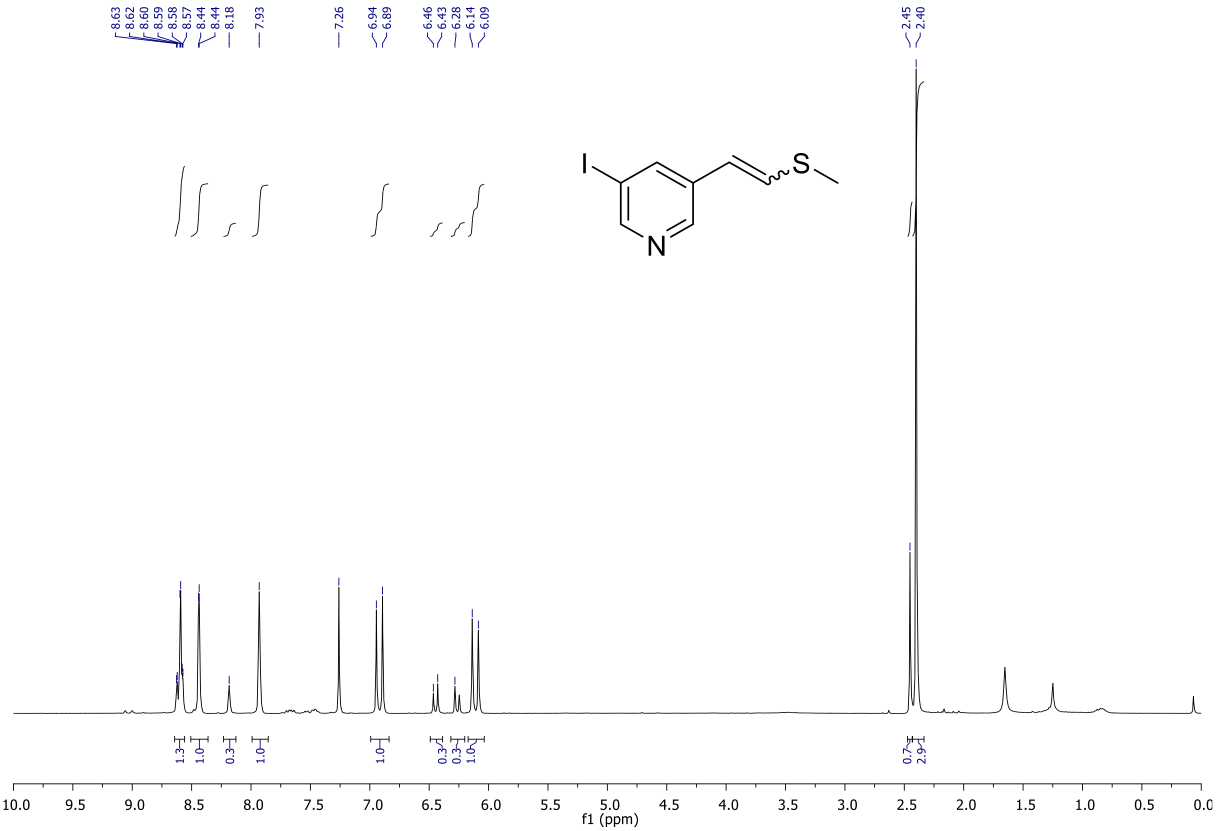

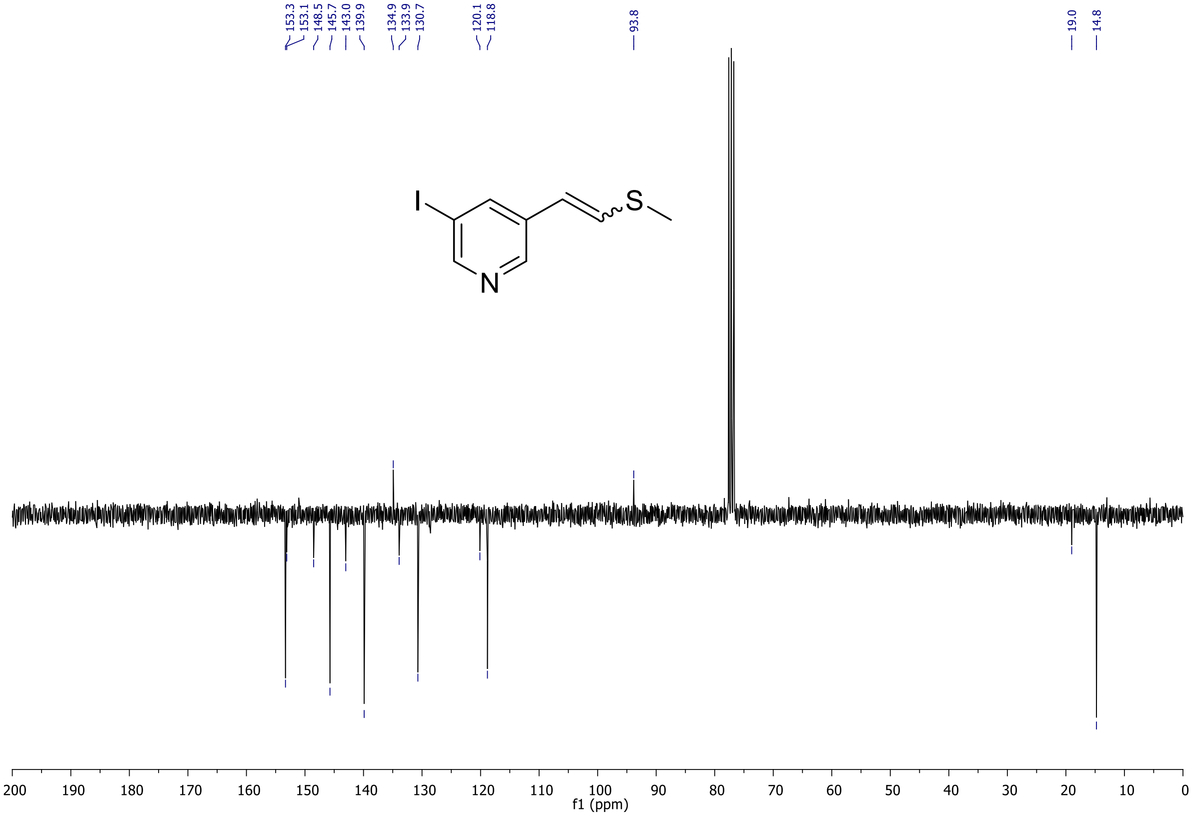

3-Iodo-5-(2-(methylthio)ethyl)pyridine (**34a**)

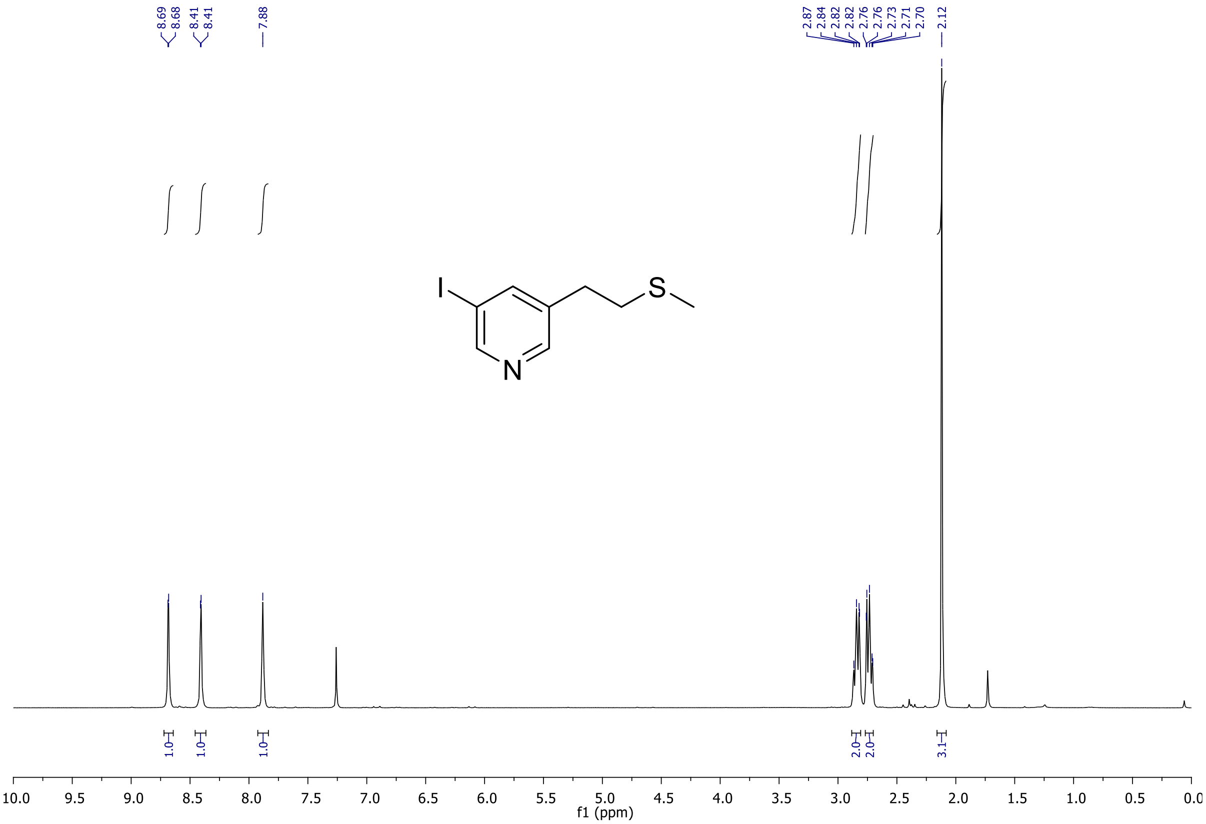

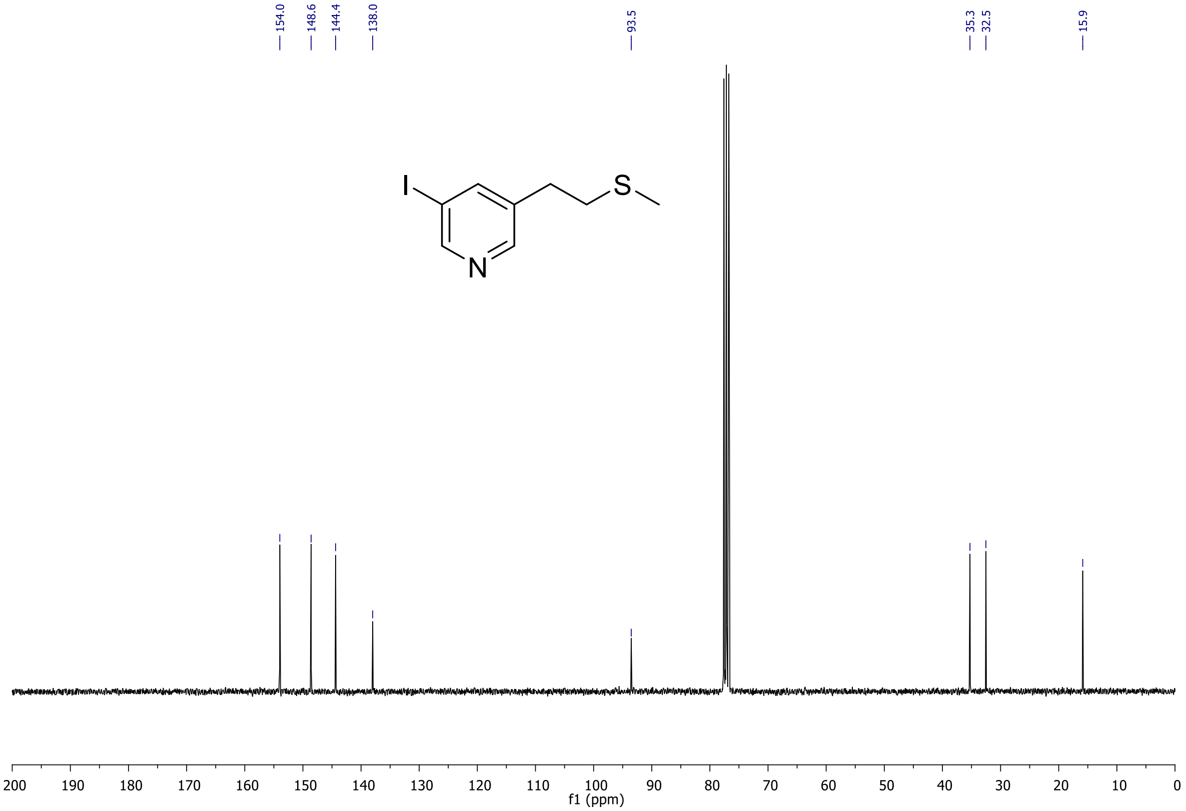

3-(2-(Methylthio)ethyl)-5-(4,4,5,5-tetramethyl-1,3,2-dioxaborolan-2-yl)pyridine (**34**)

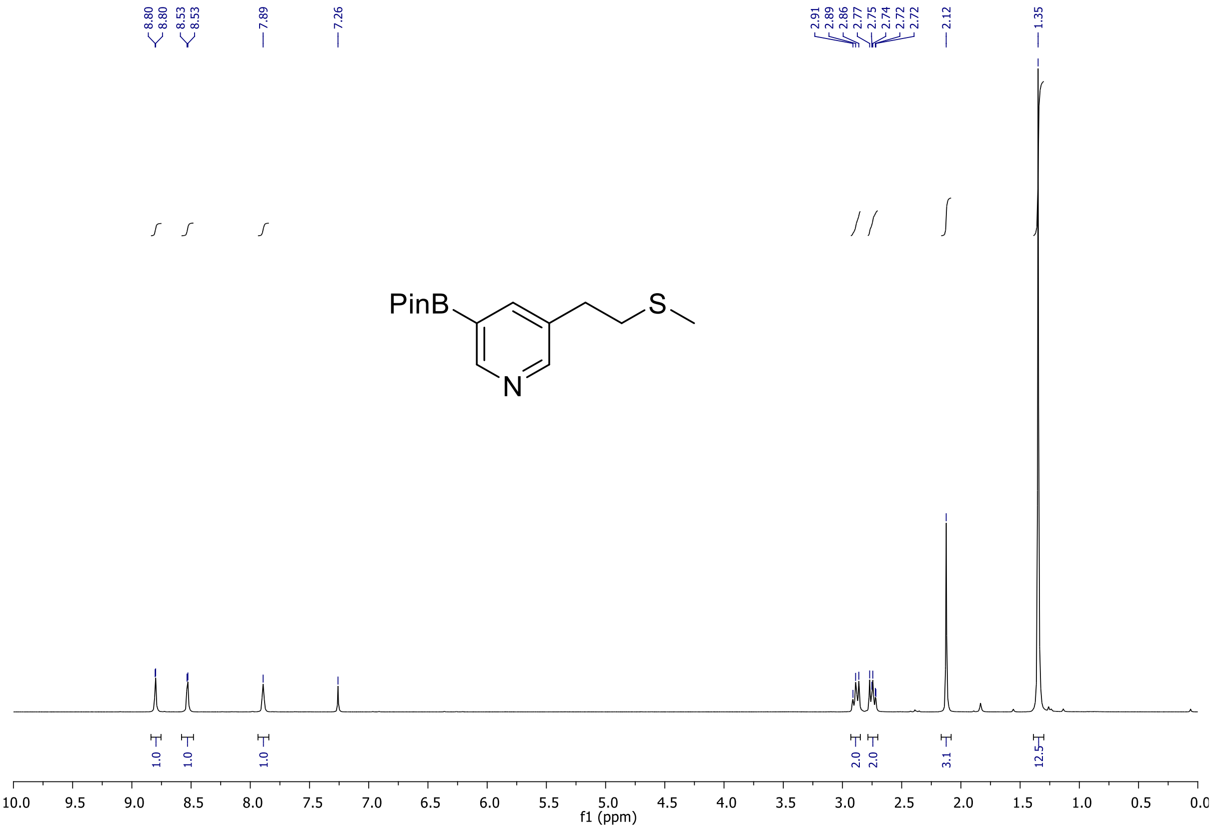

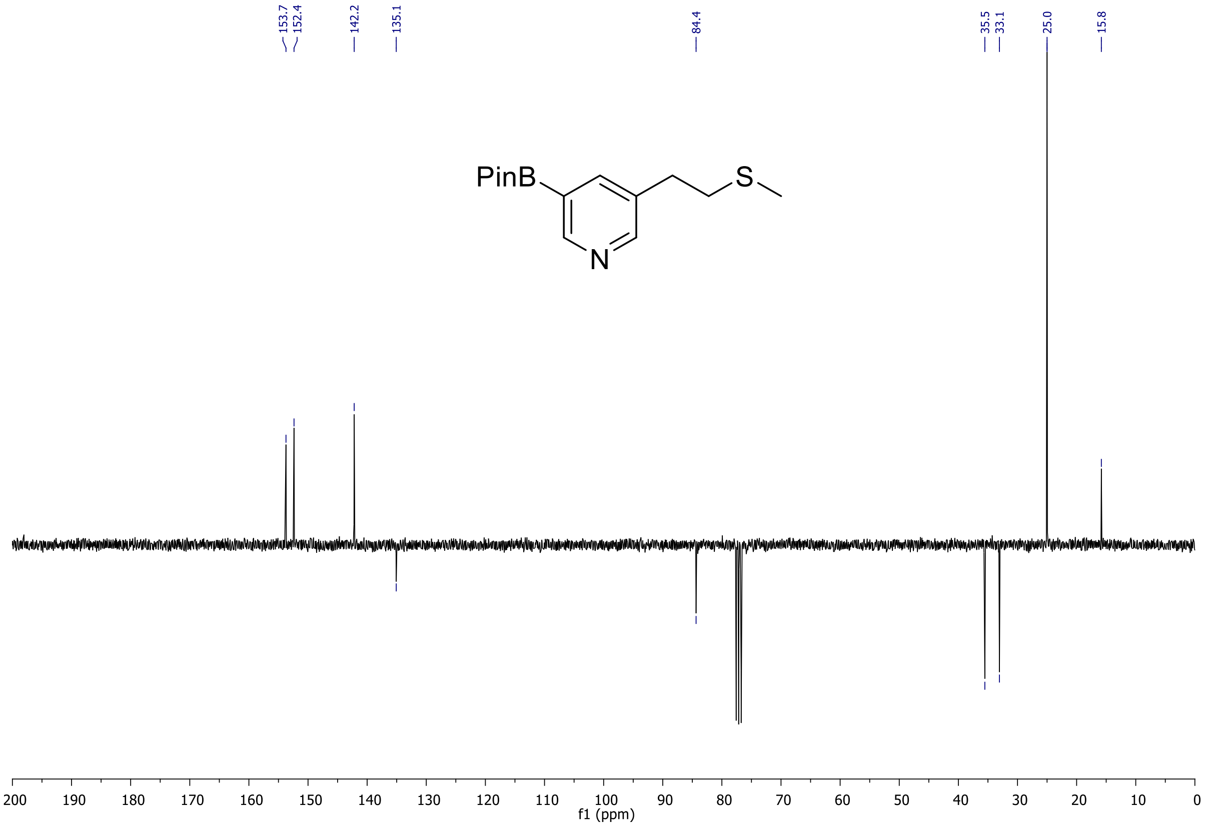

3-Methyl-5-(4,4,5,5-tetramethyl-1,3,2-dioxaborolan-2-yl)pyridine (**36**)

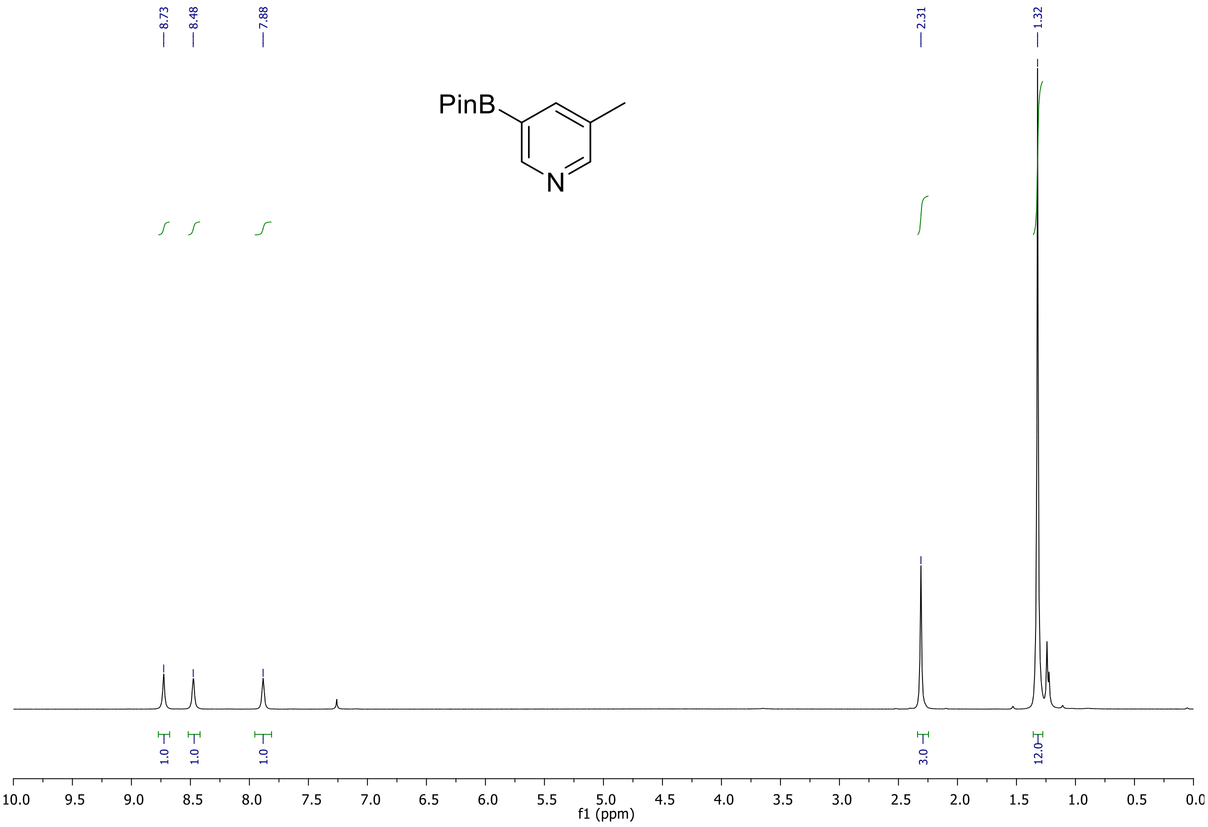

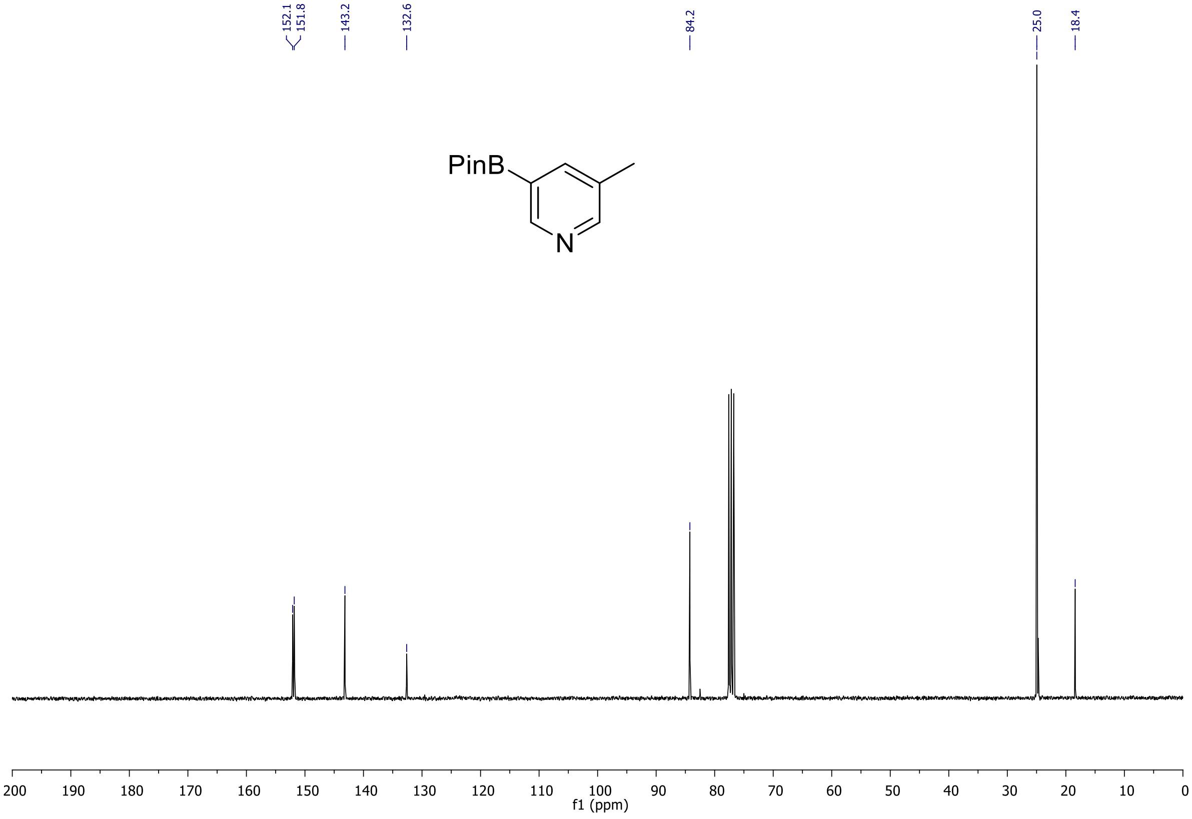

3-Chloro-5-isobutylpyridine (**38**)

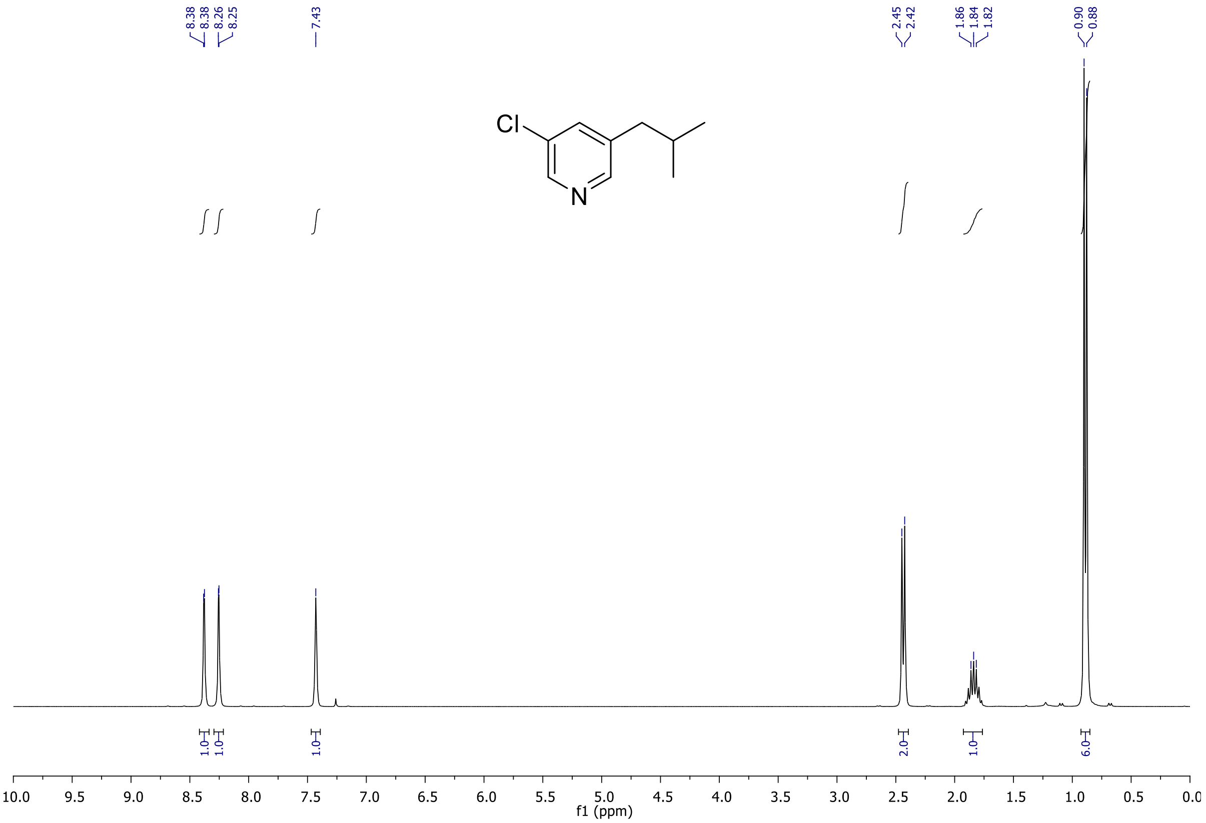

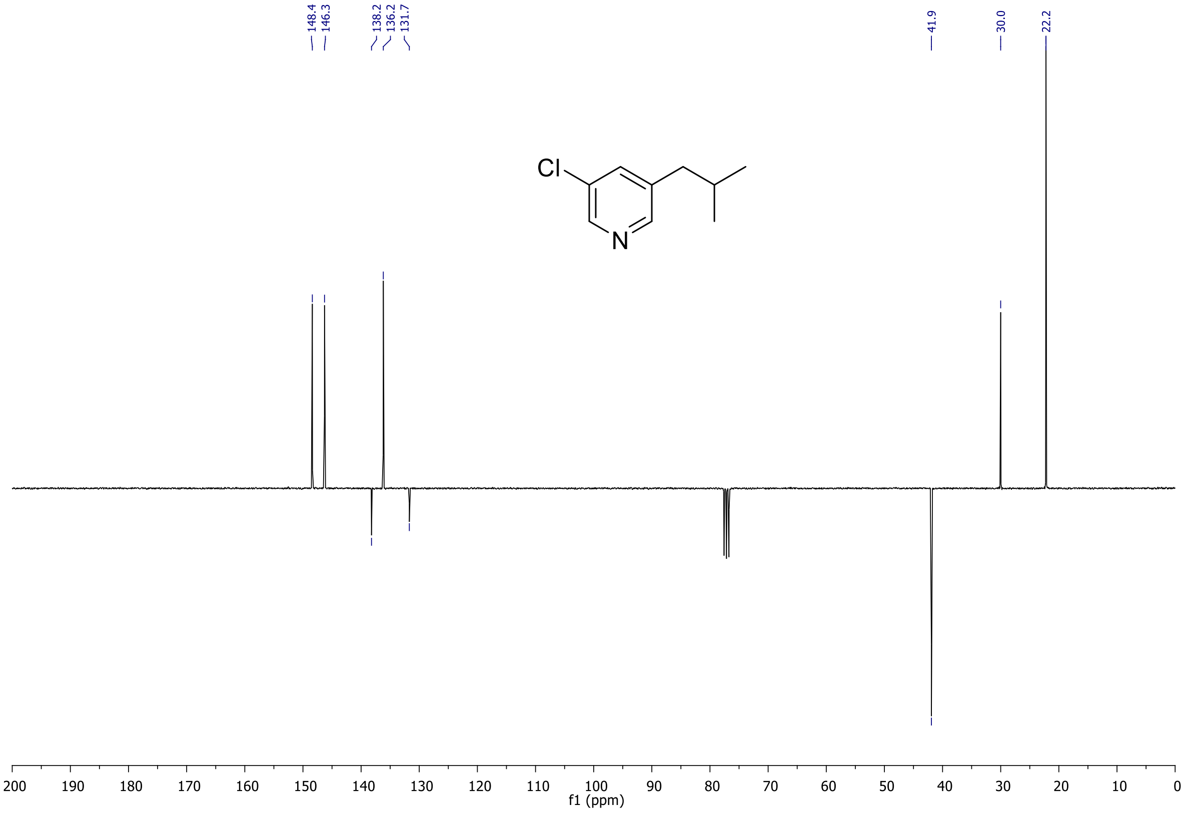

3-Isobutyl-5-(4,4,5,5-tetramethyl-1,3,2-dioxaborolan-2-yl)pyridine (**39**)

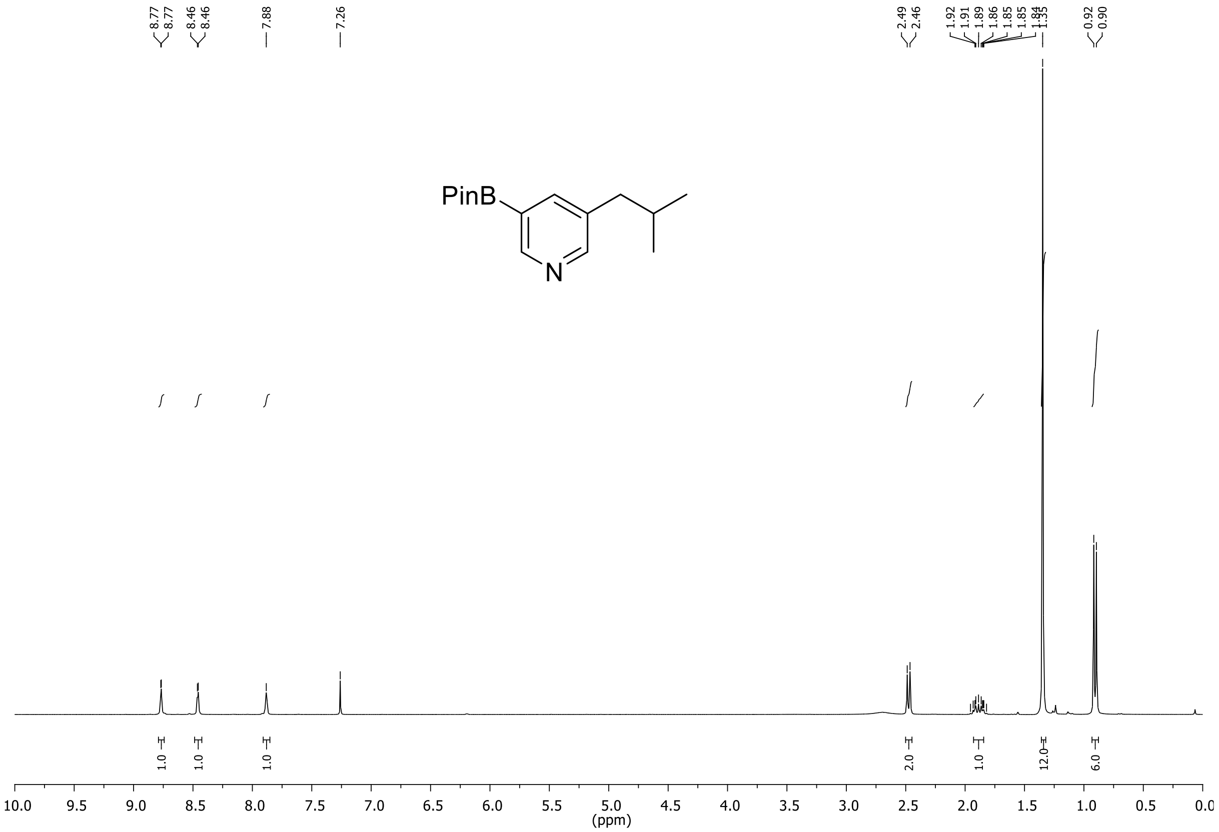

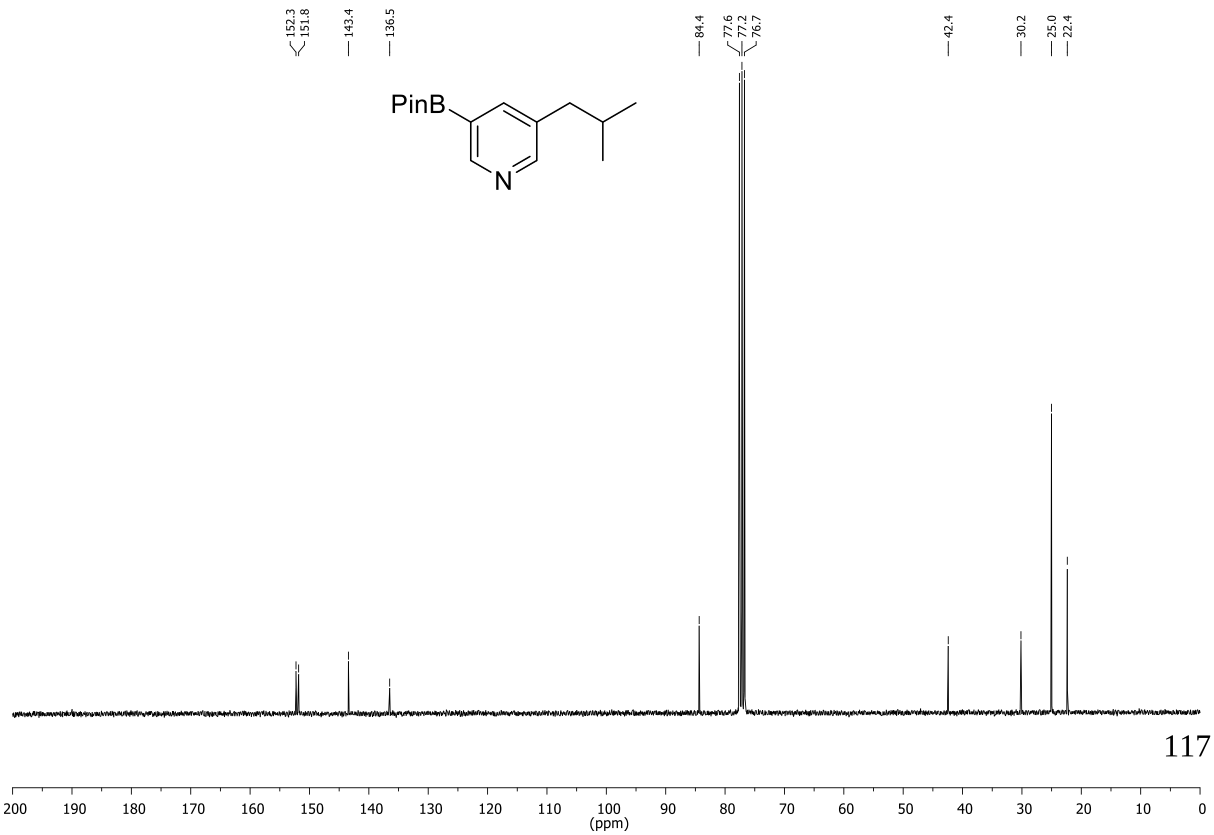

(5-Bromopyridin-3-yl)methanol (**41a**)

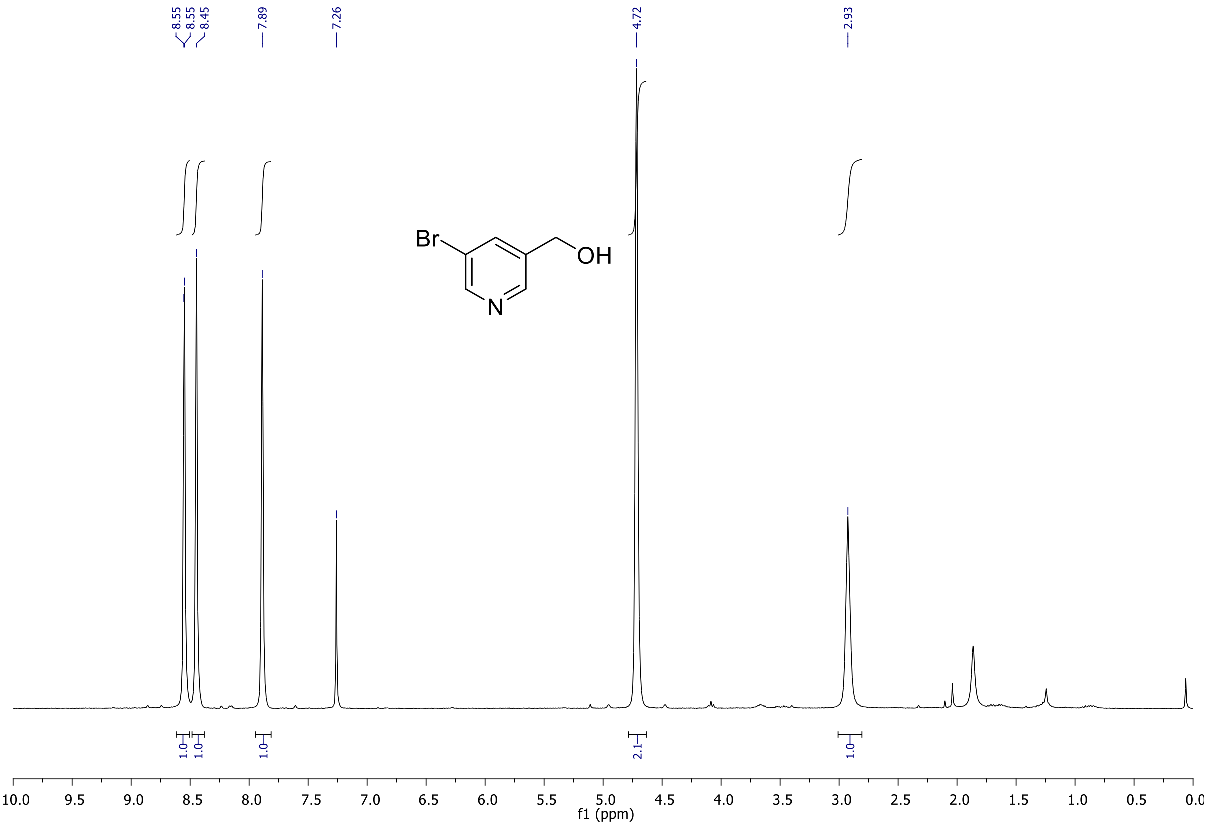

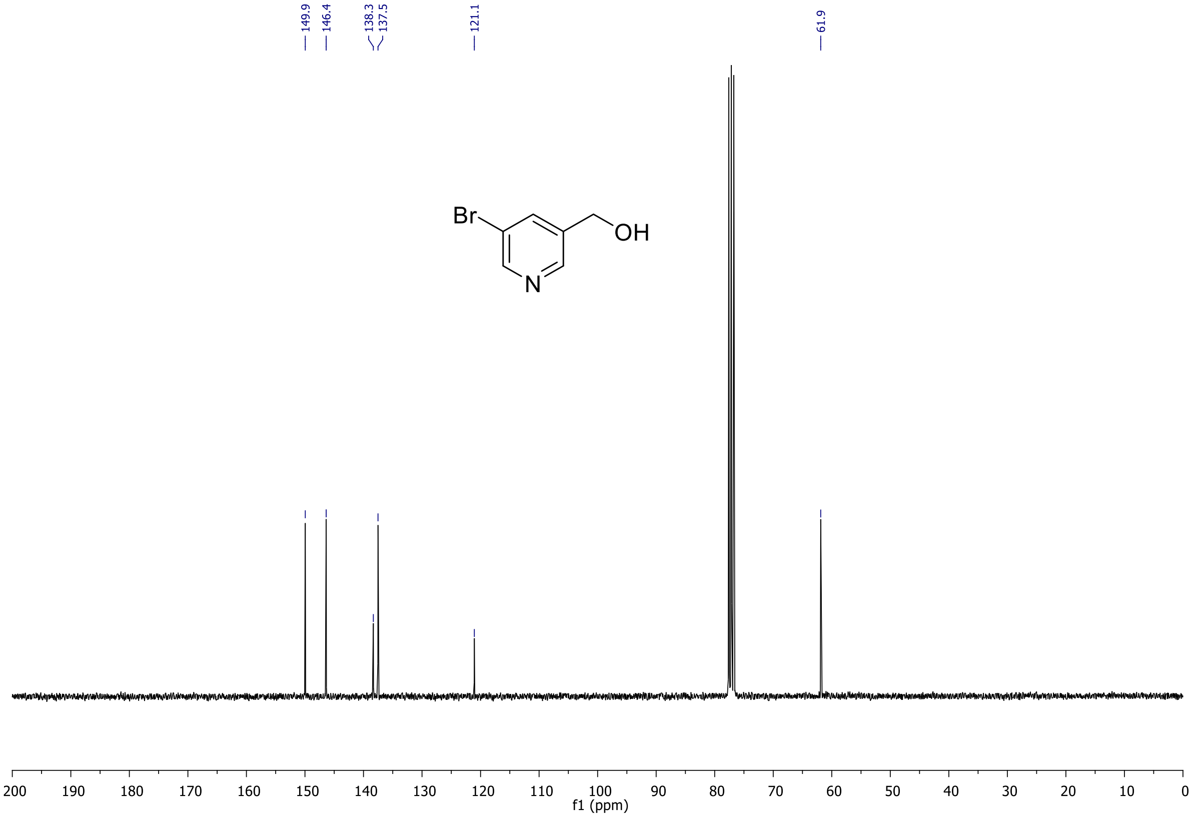

(5-Iodopyridin-3-yl)methanol (**41**)

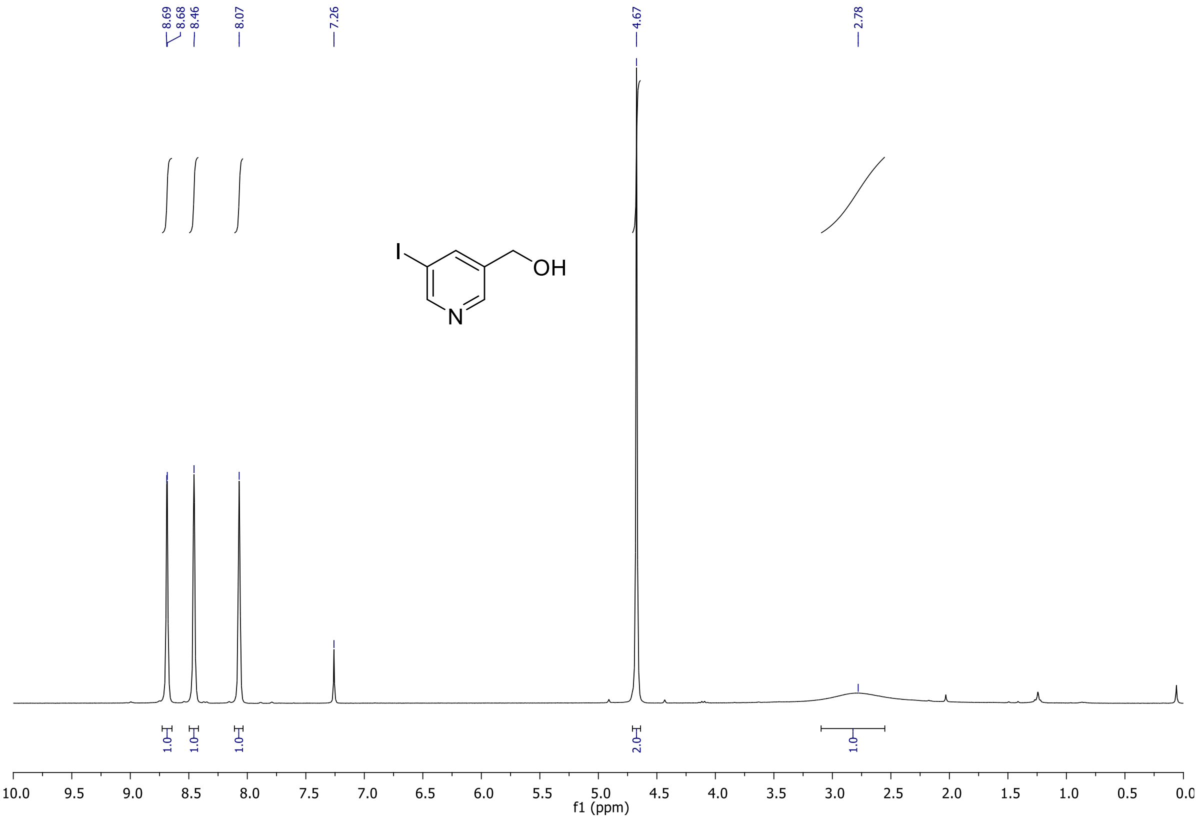

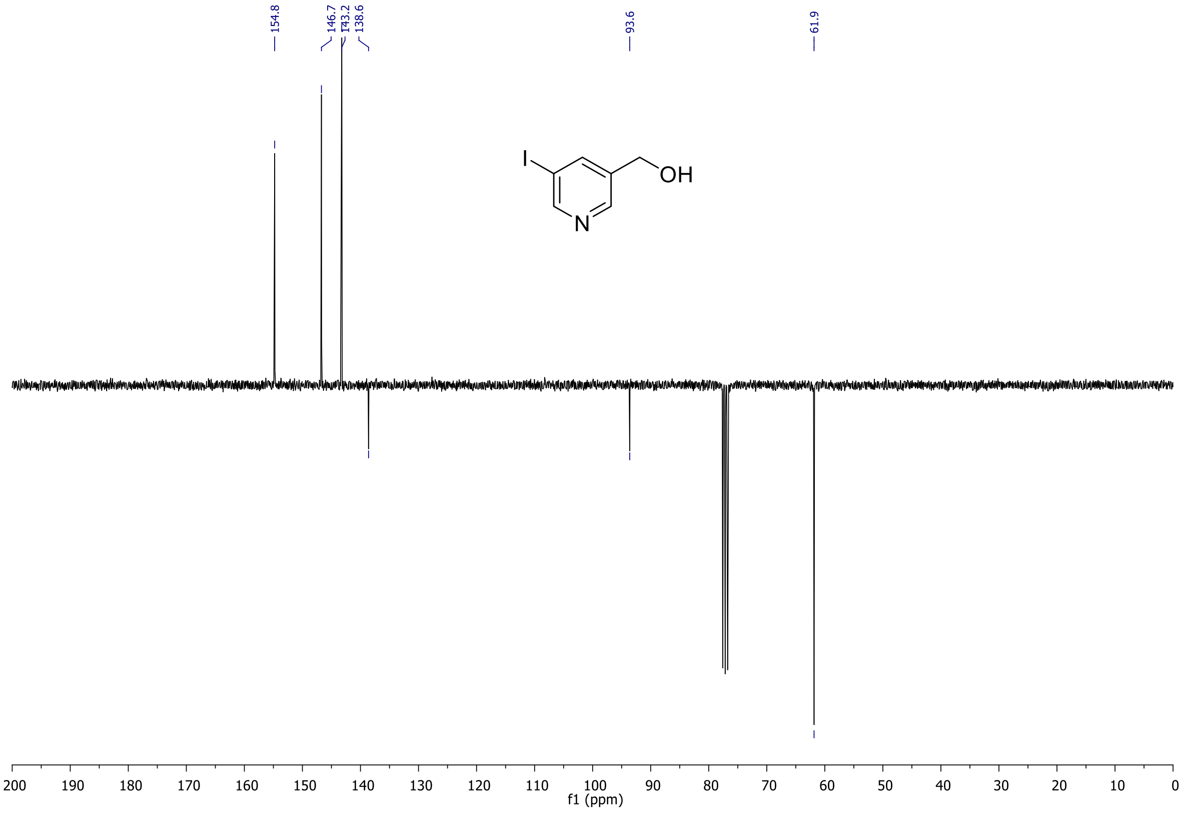

3-(Chloromethyl)-5-iodopyridine (**42**)

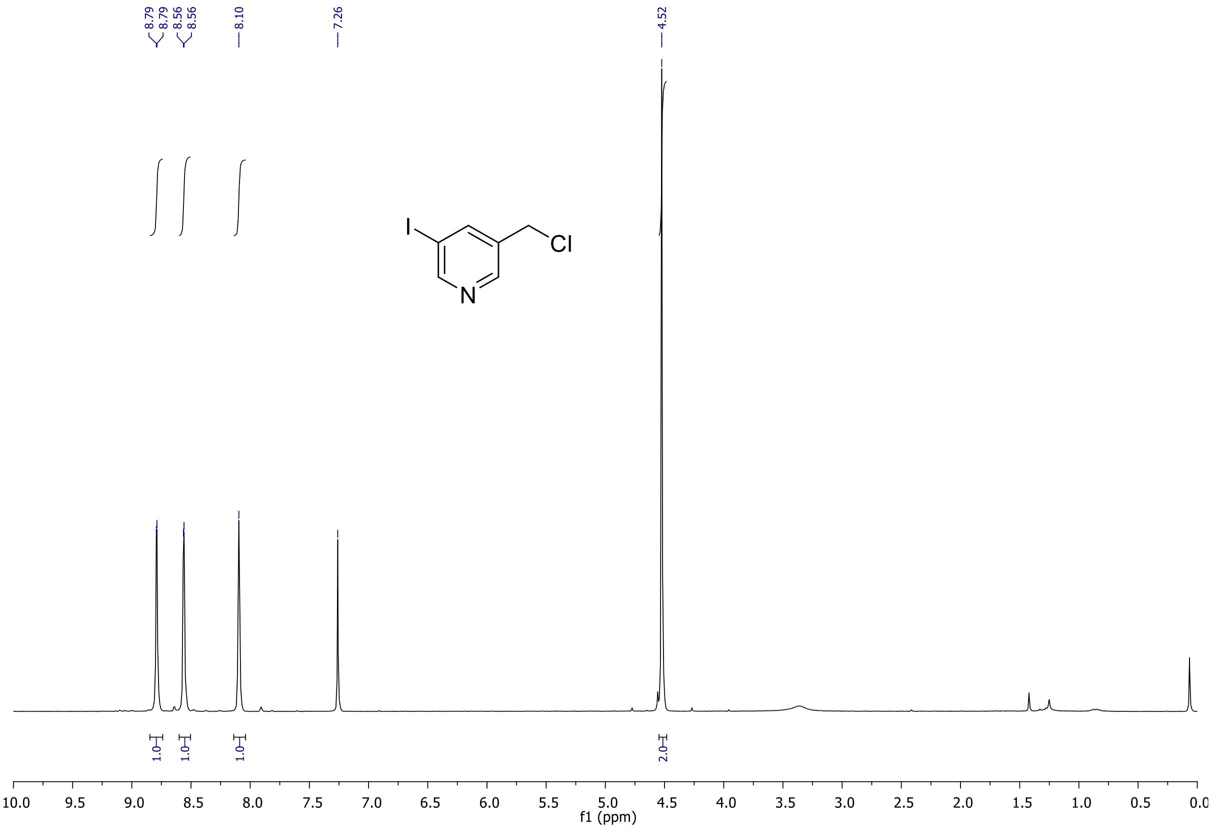

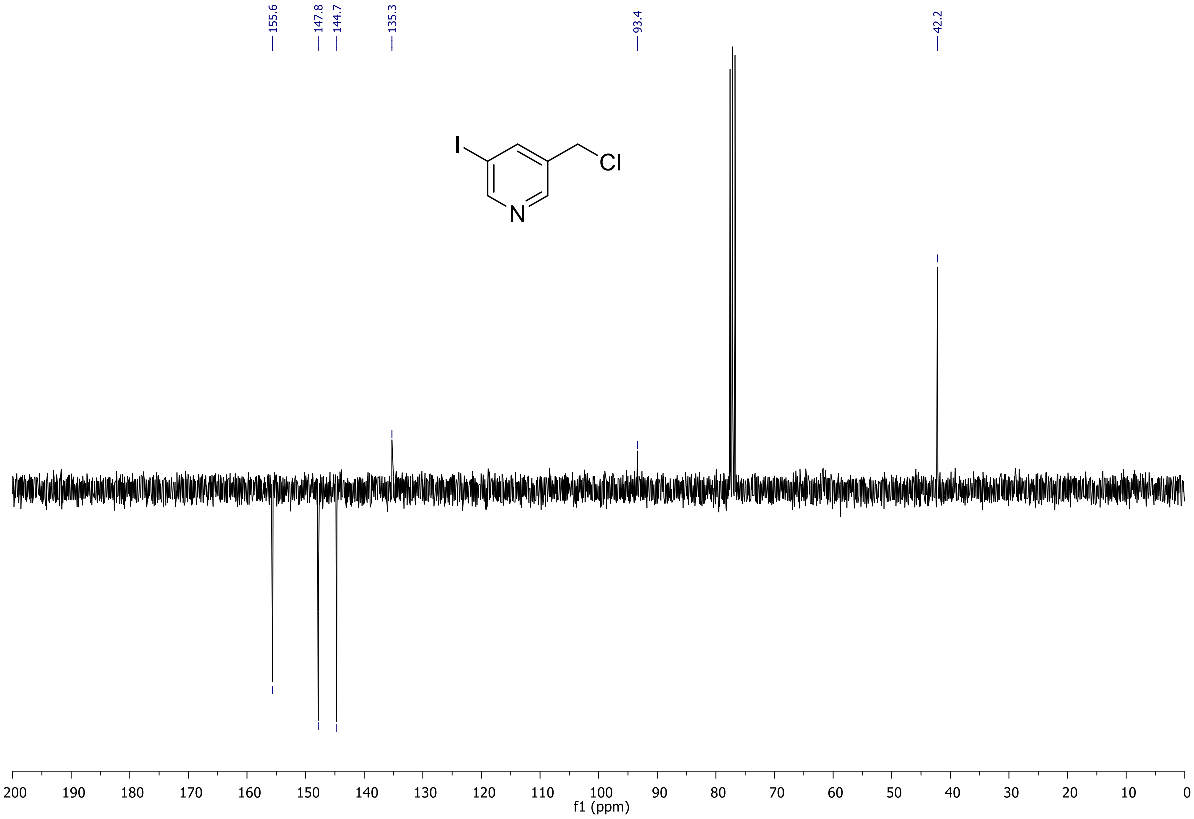

2-Methoxy-2-methylpropanoic acid (**43a**)

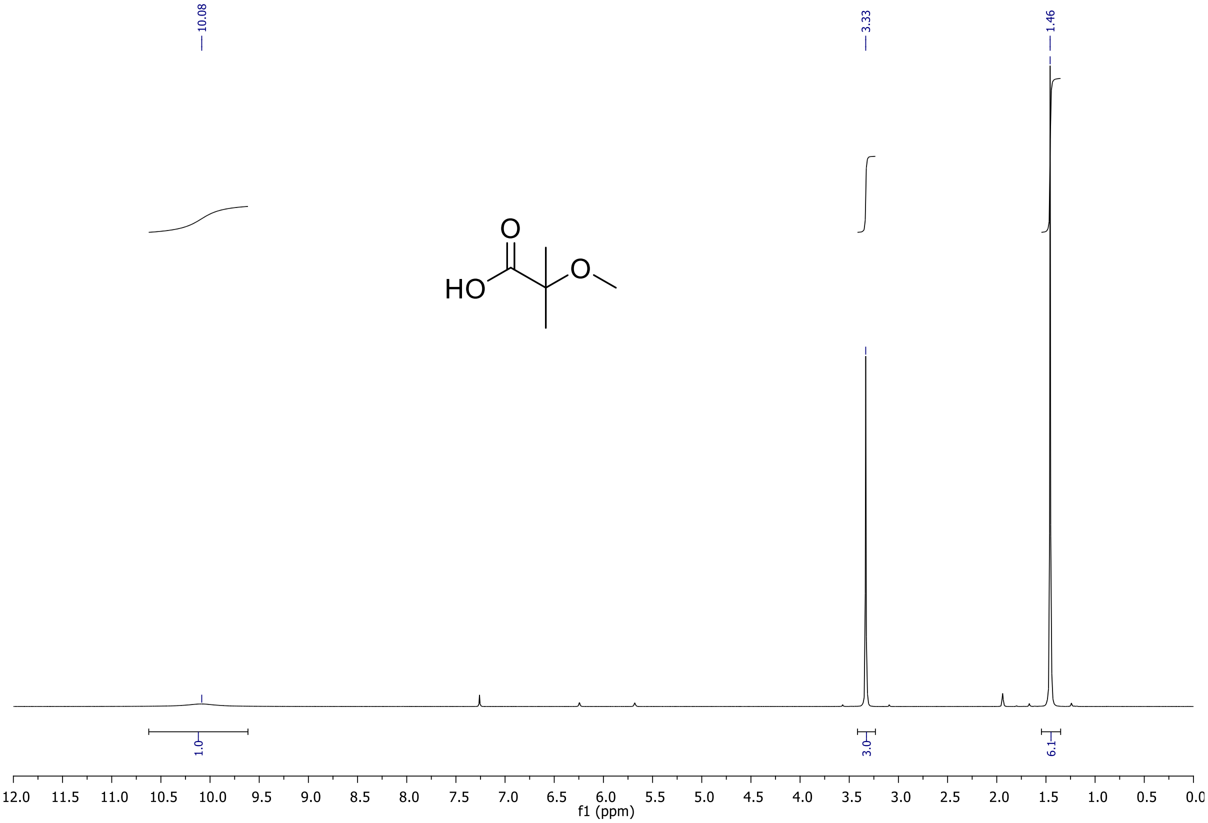

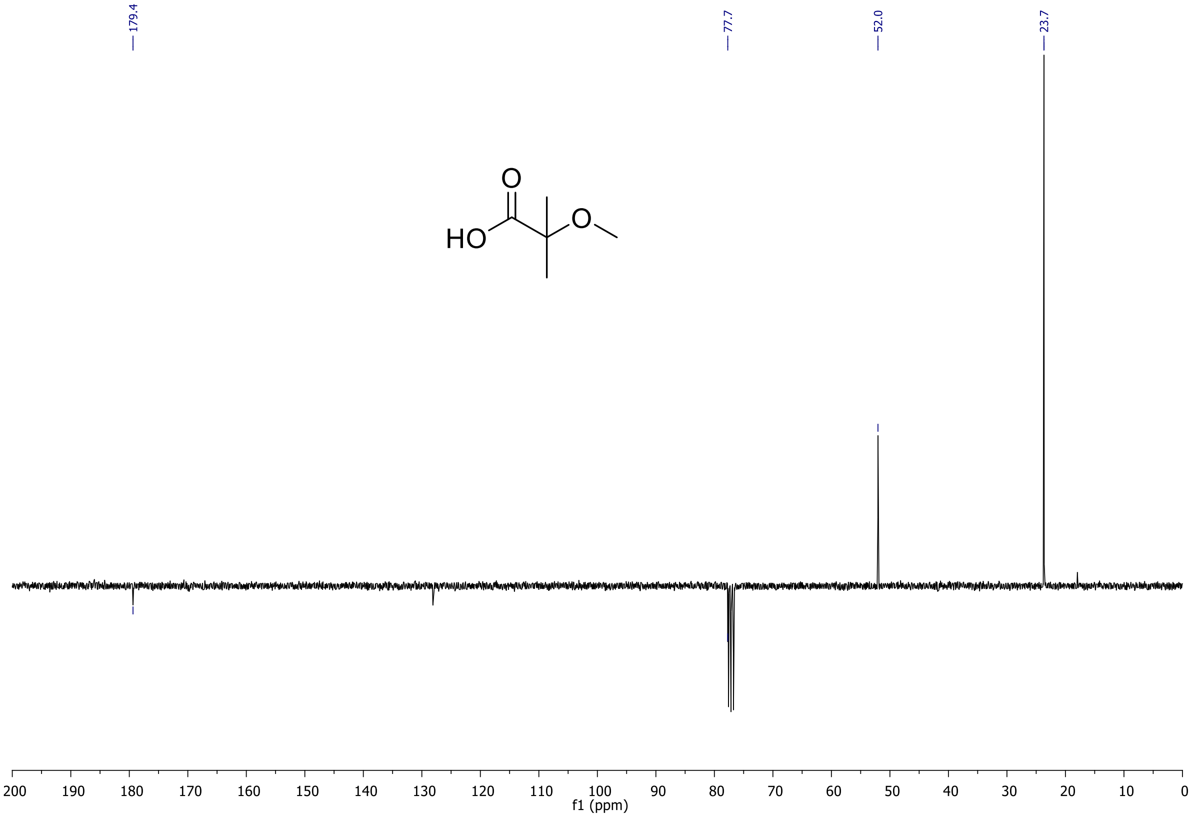

2-Methoxy-2-methylpropanethioic *S*-acid (**43**)

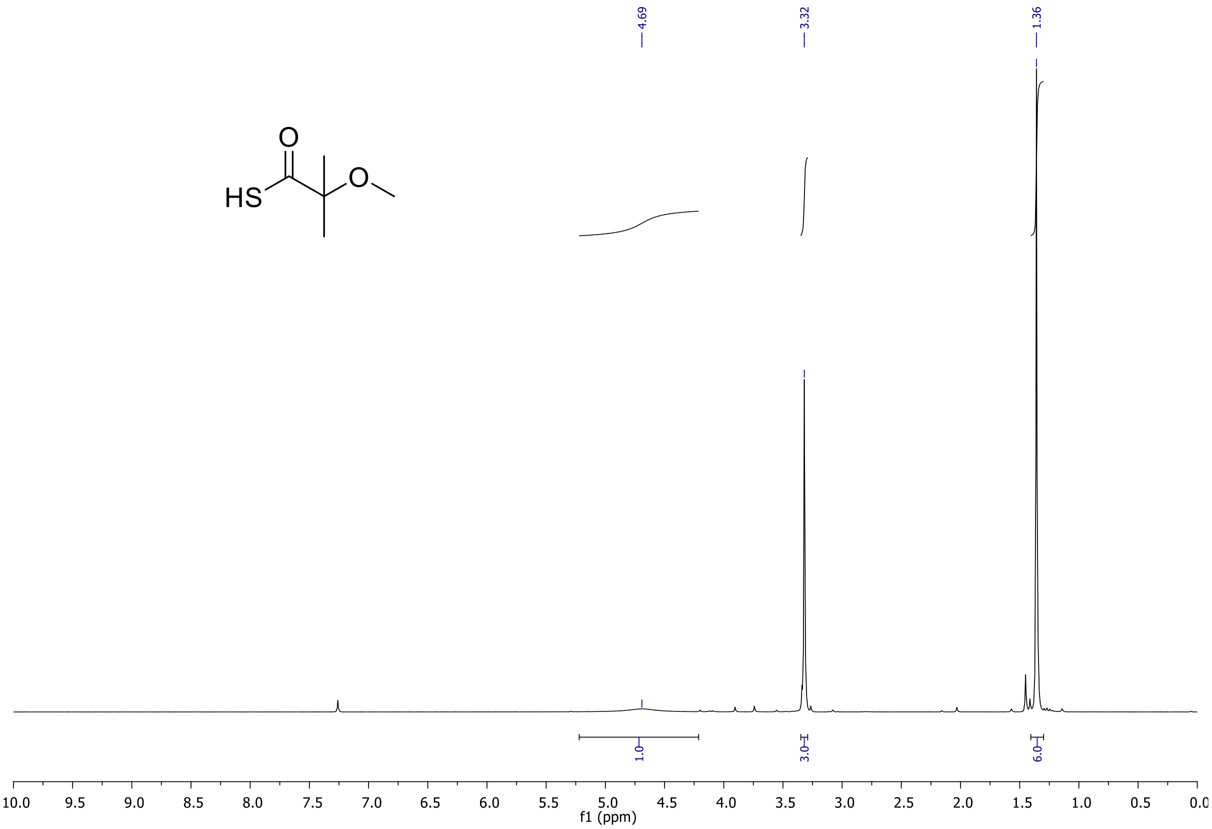

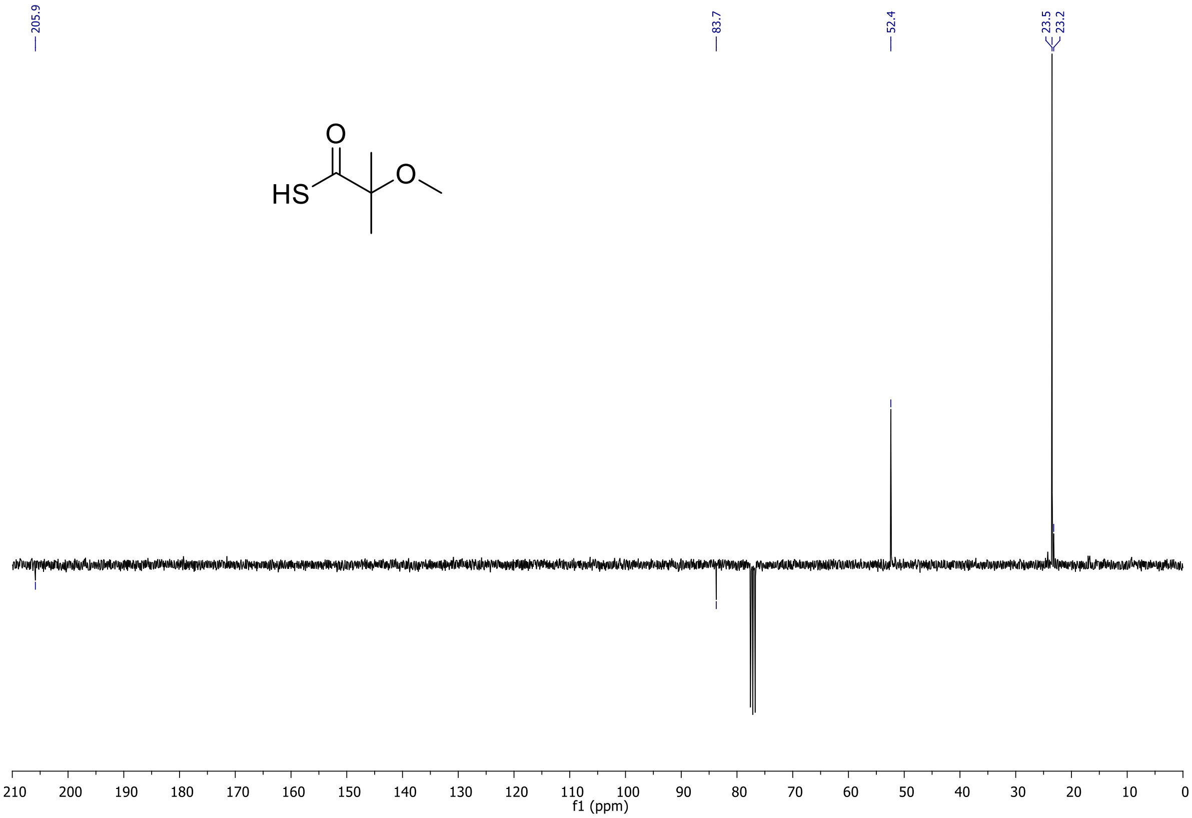

S-((5-Iodopyridin-3-yl)methyl) 2-methoxy-2-methylpropanethioate (**44a**)

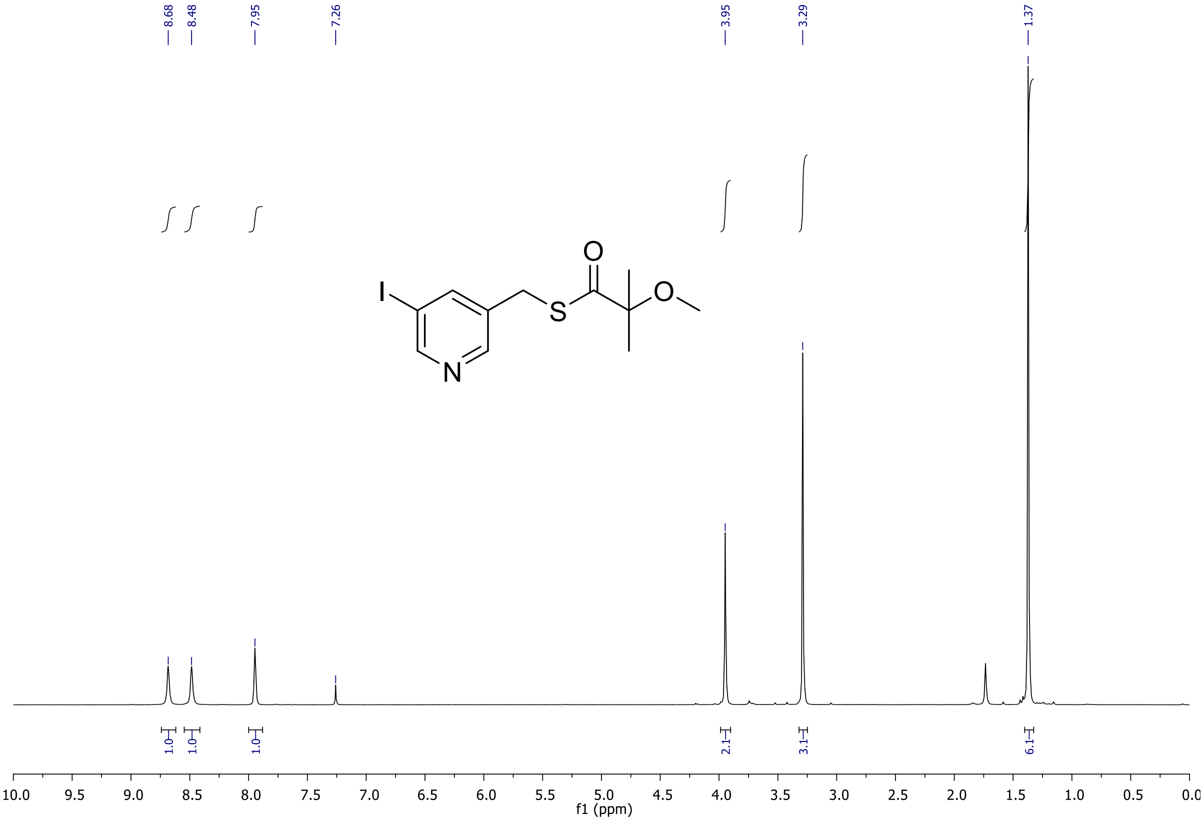

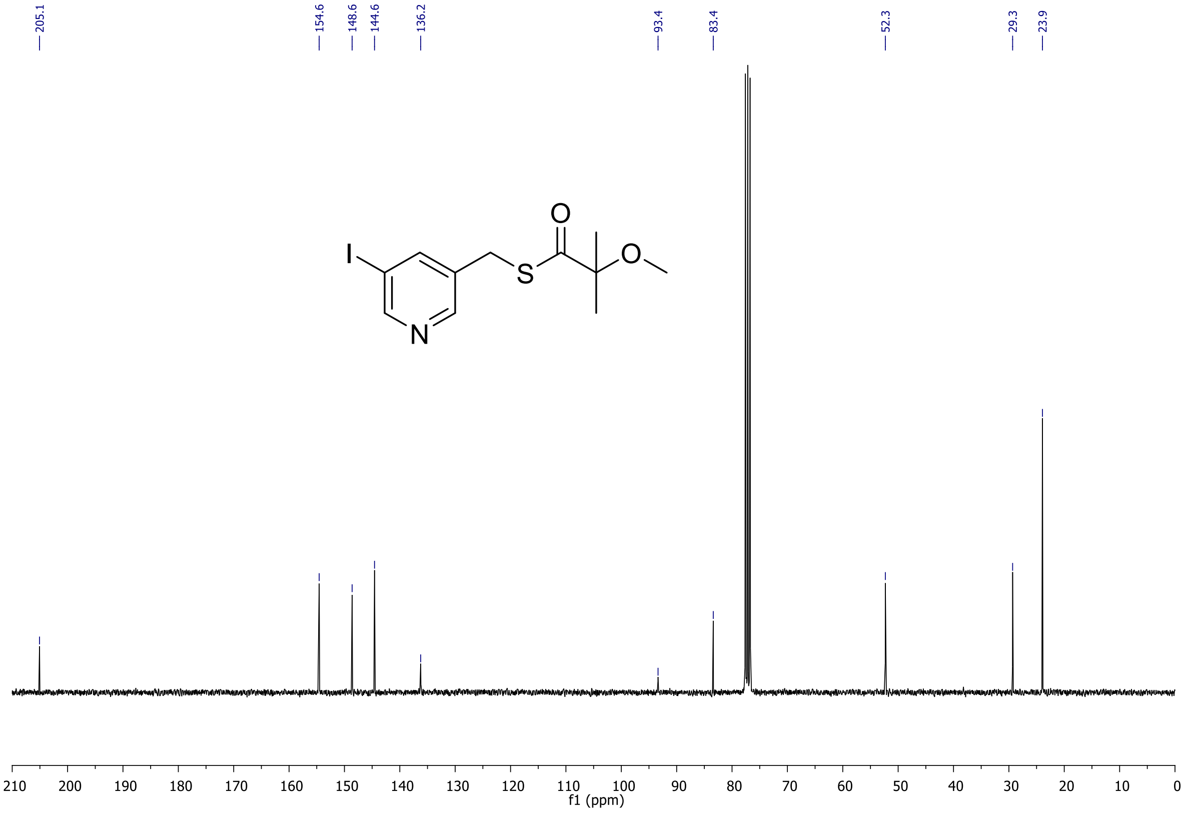

S-((5-(6-methyl-4,8-dioxo-1,3,6,2-dioxazaborocan-2-yl)pyridin-3-yl)methyl) 2-methoxy-2-methylpropanethioate (**44**)

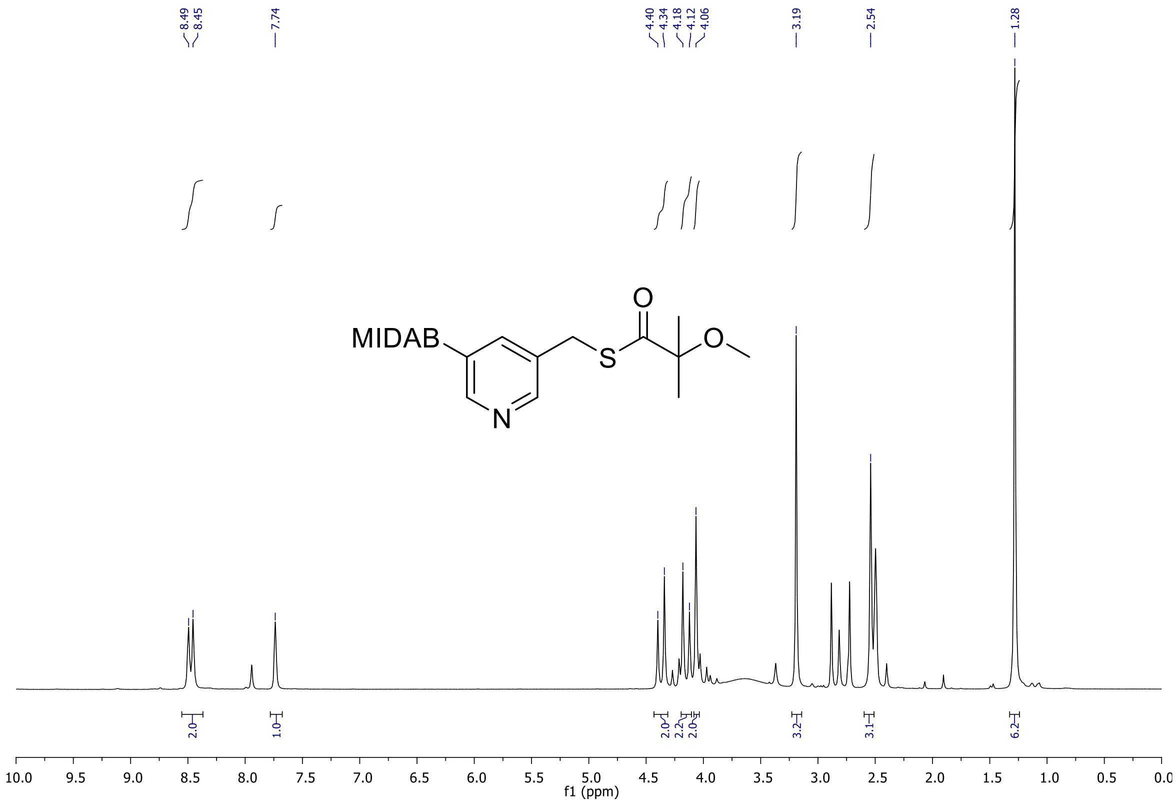

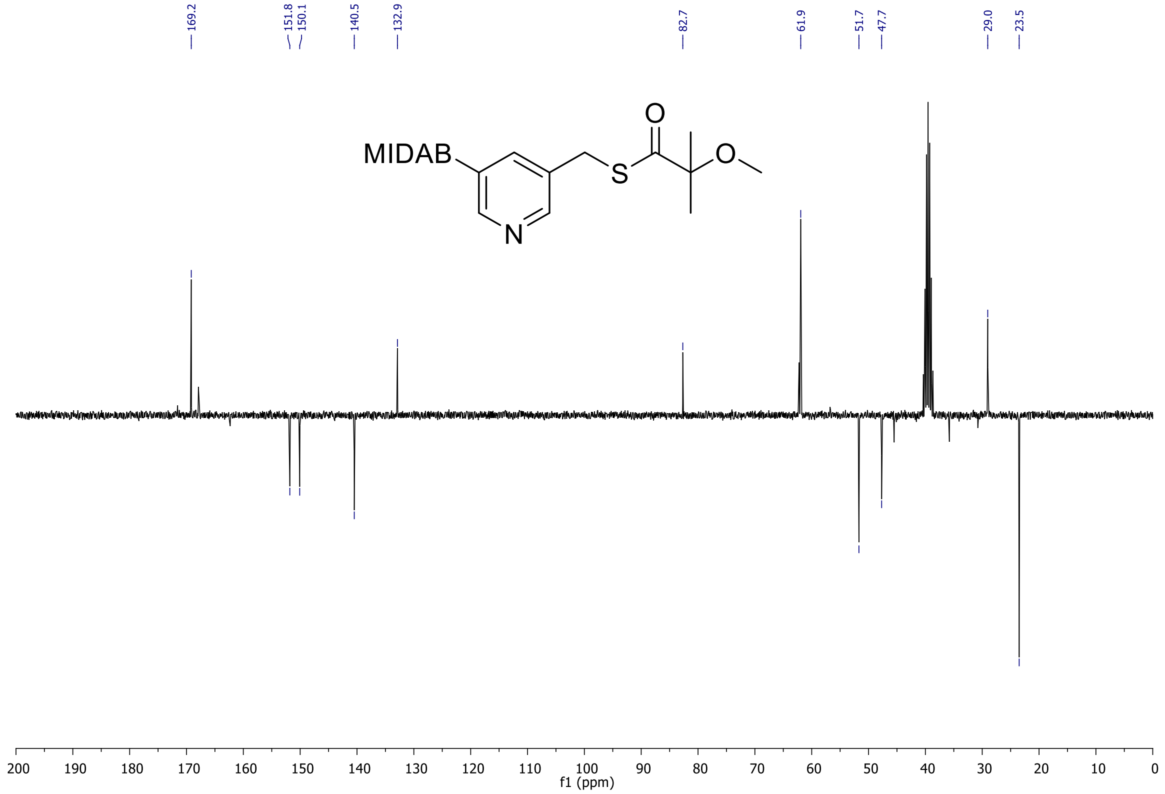

3-(((*tert*-Butyldiphenylsilyl)oxy)methyl)-5-iodopyridine (**45a**)

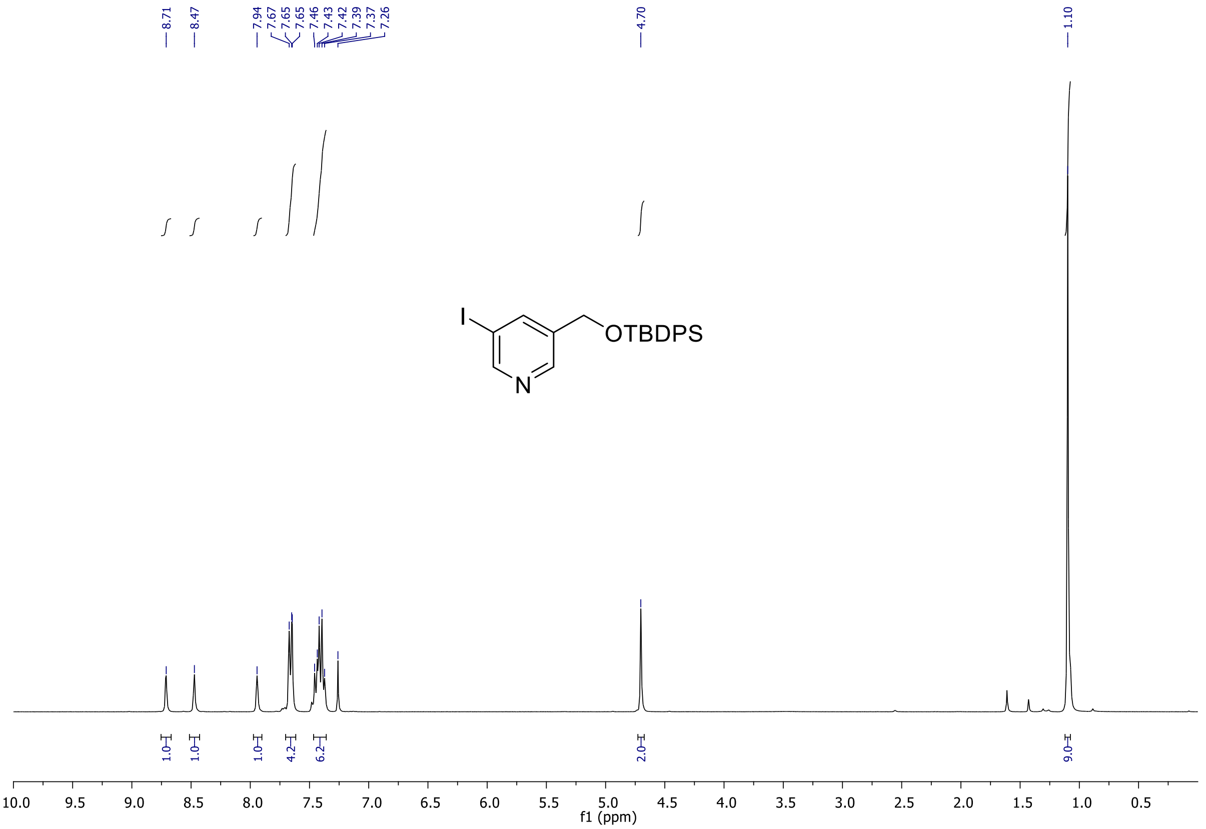

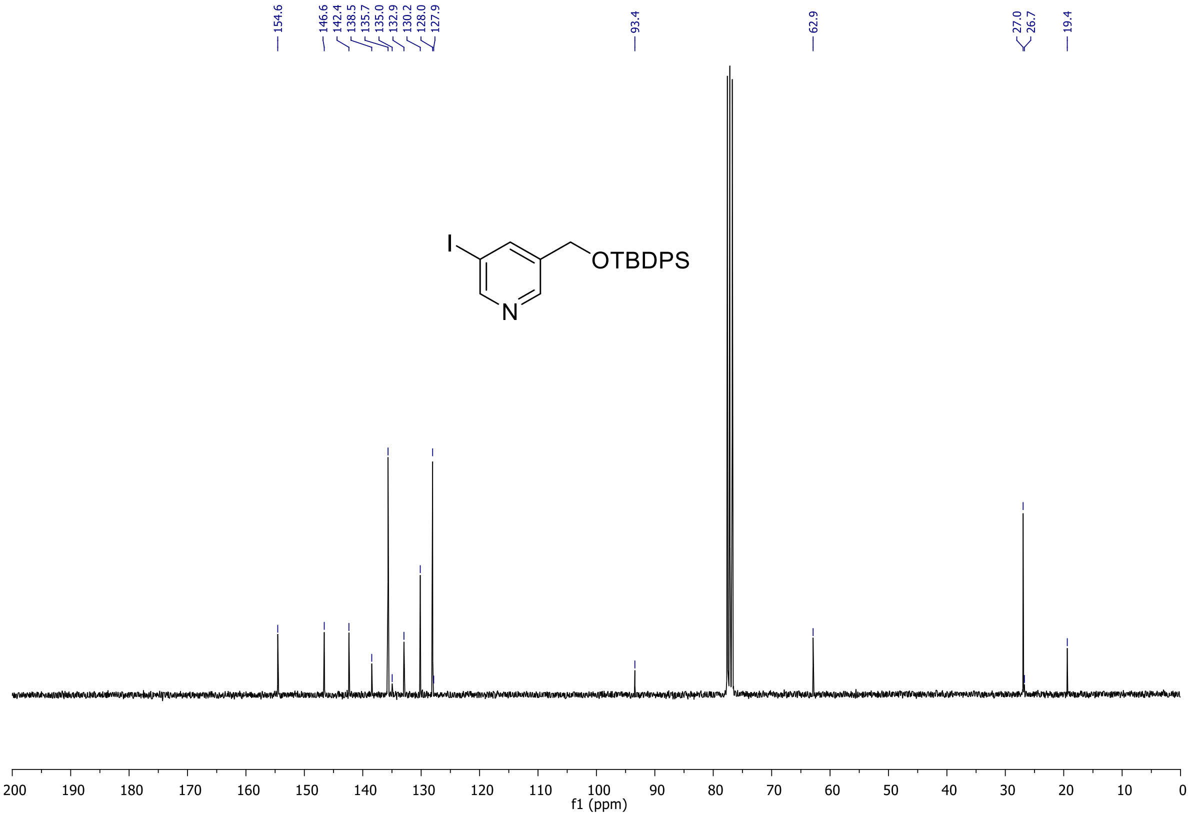

3-(((*tert*-Butyldiphenylsilyl)oxy)methyl)-5-(4,4,5,5-tetramethyl-1,3,2-dioxaborolan-2-yl)pyridine (**45**)

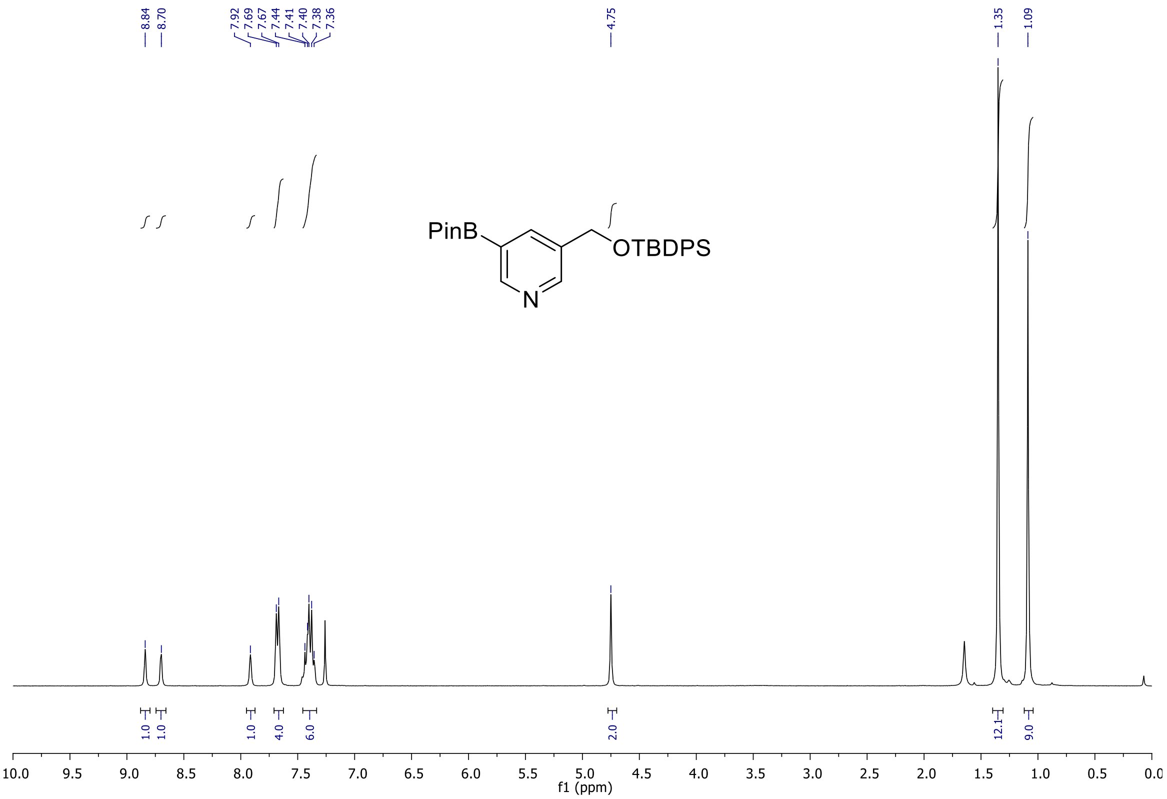

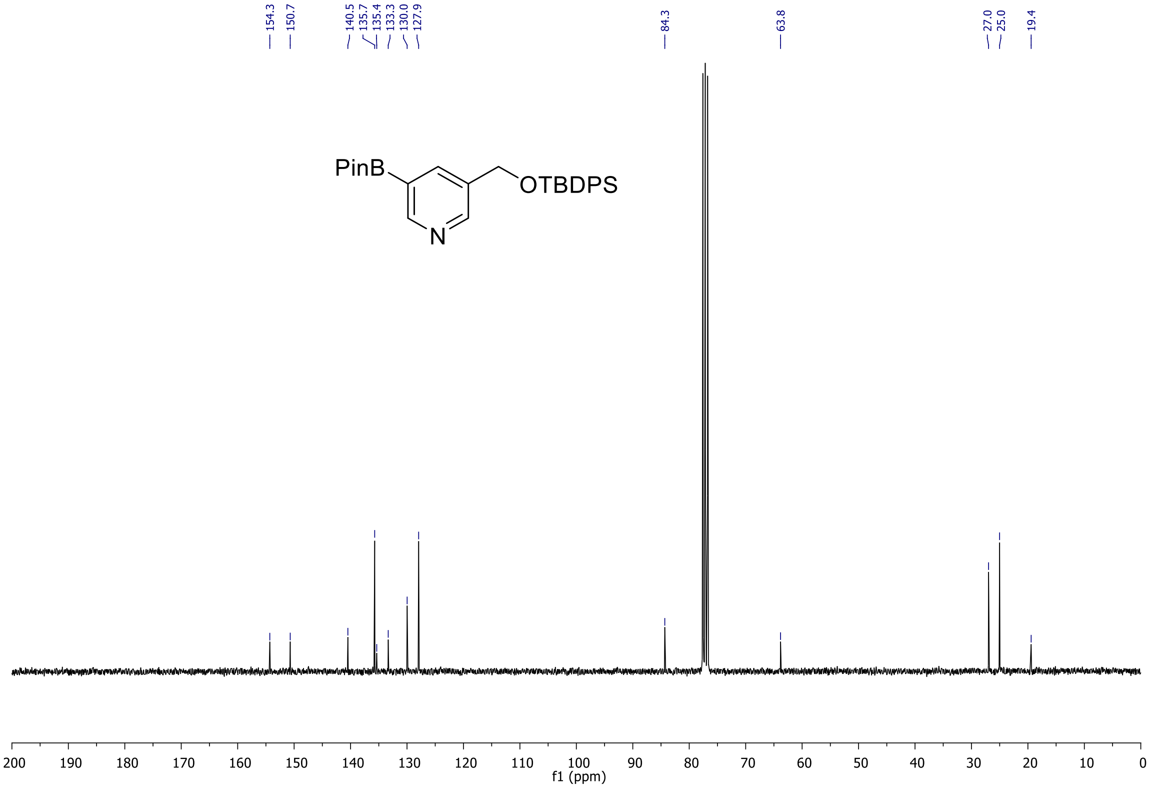

Methyl 2-(5-(4-(5-((1*H*-indol-3-yl)methyl)pyridin-3-yl)-2-isopropylphenyl)pyridin-3-yl)acetate (**46a**)

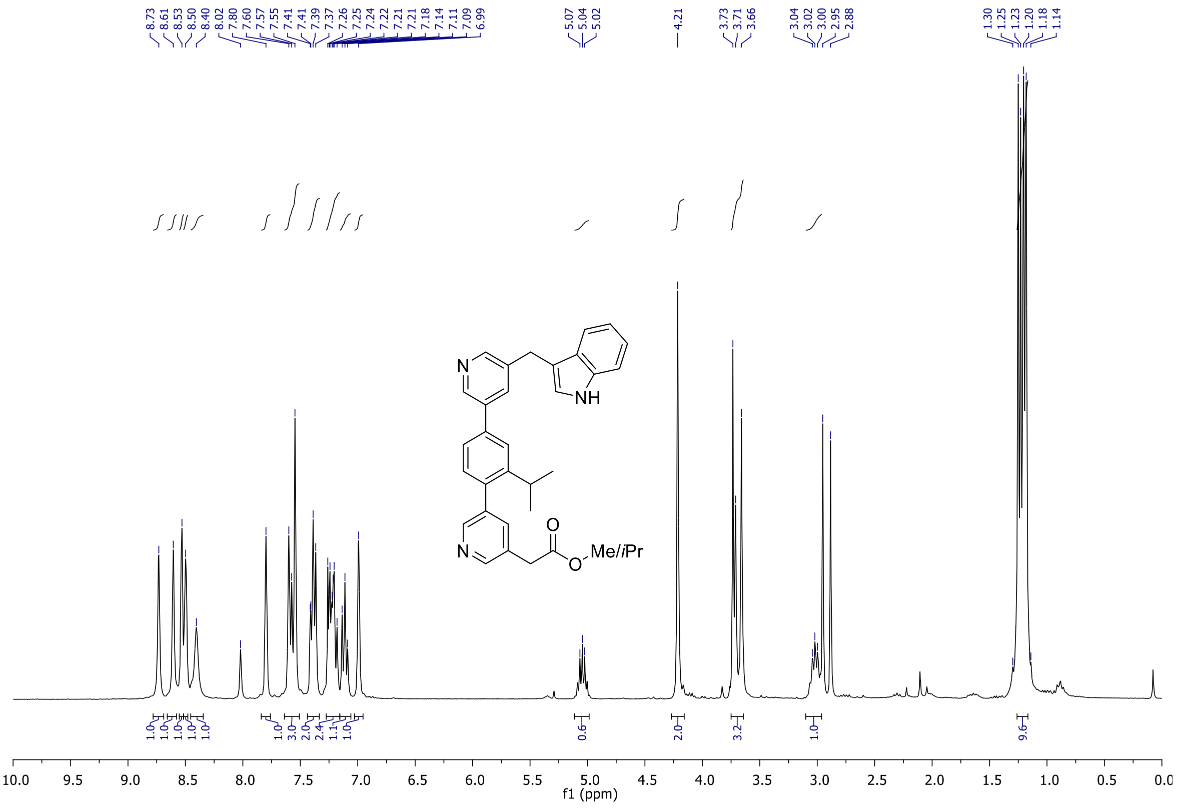

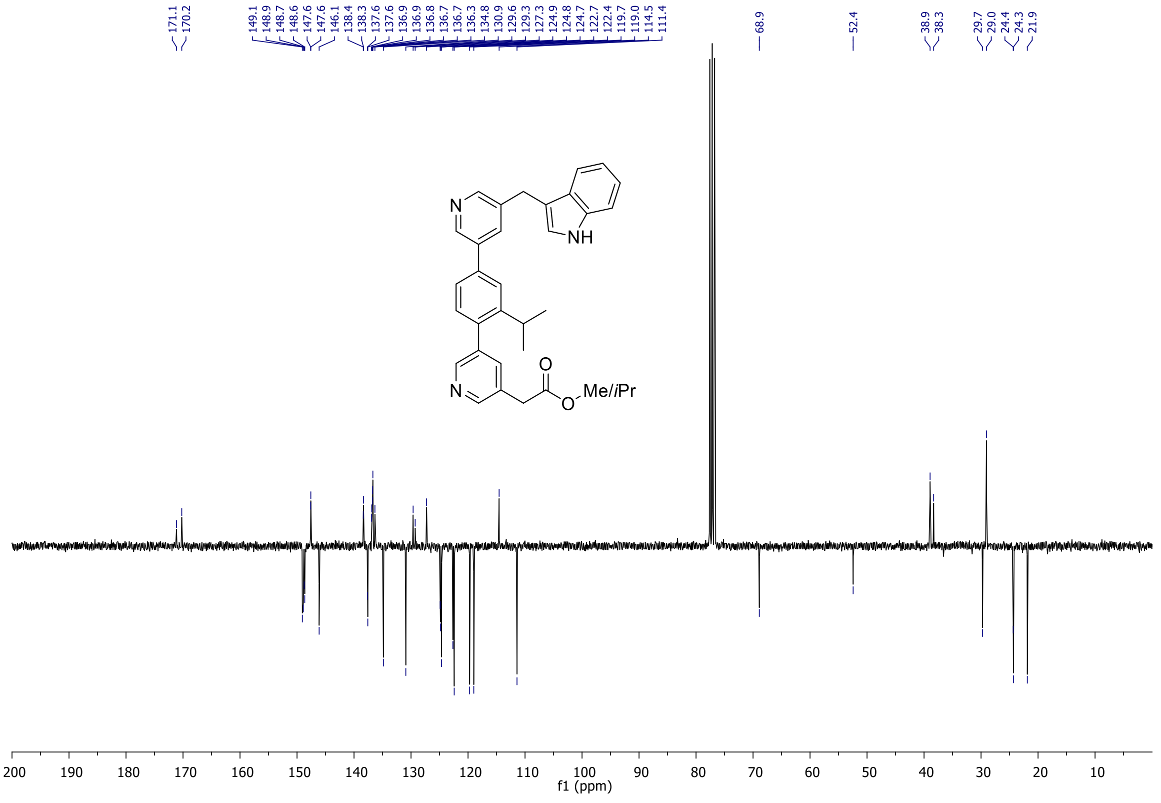

Trp-Val-Asp (**46**)

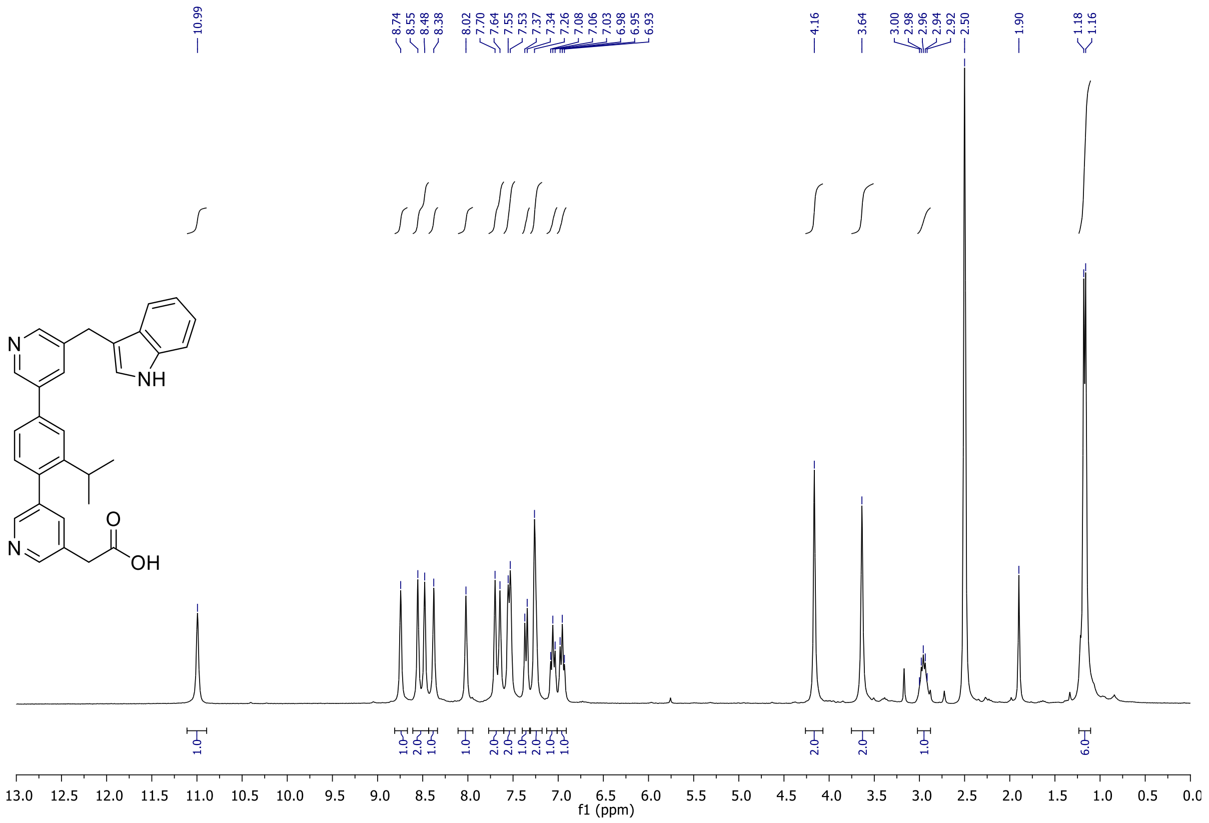

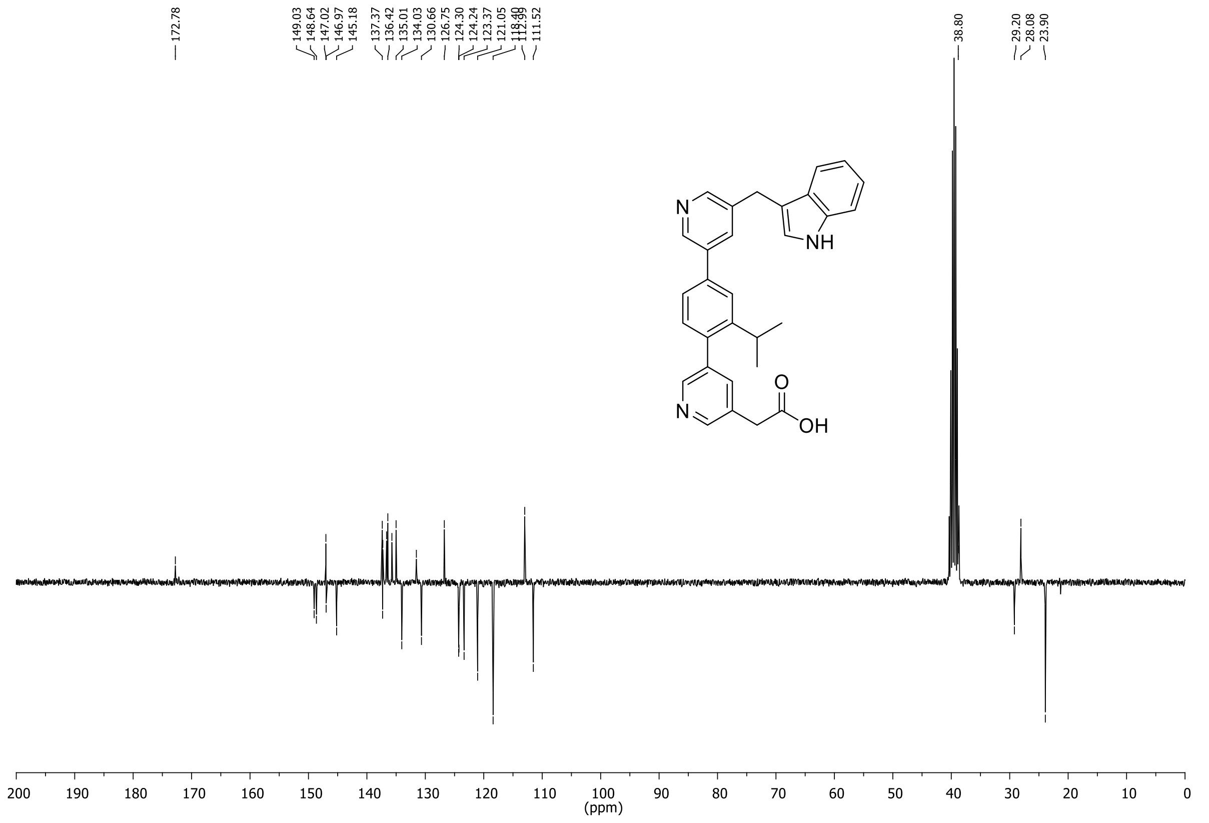

4-(5-(4-(5-Benzylpyridin-3-yl)-2-methylphenyl)pyridin-3-yl)butanenitrile (**47a**)

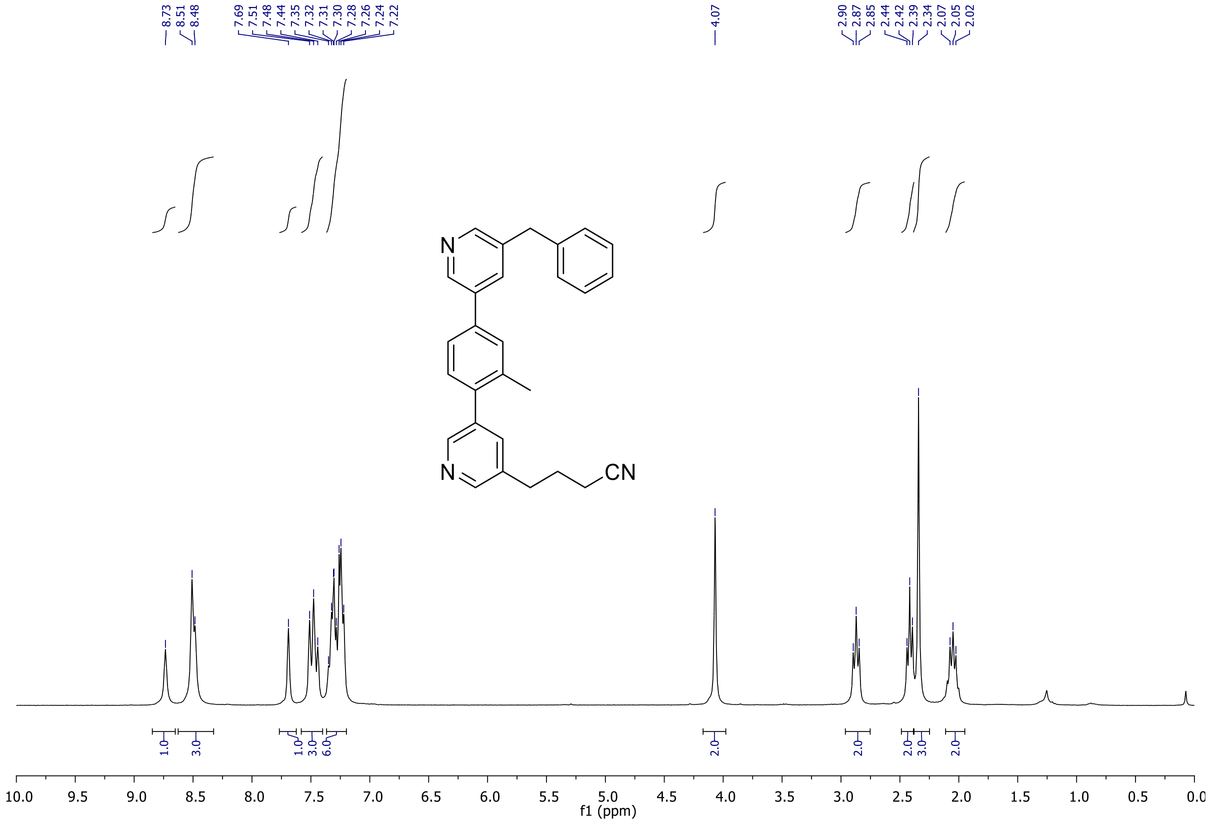

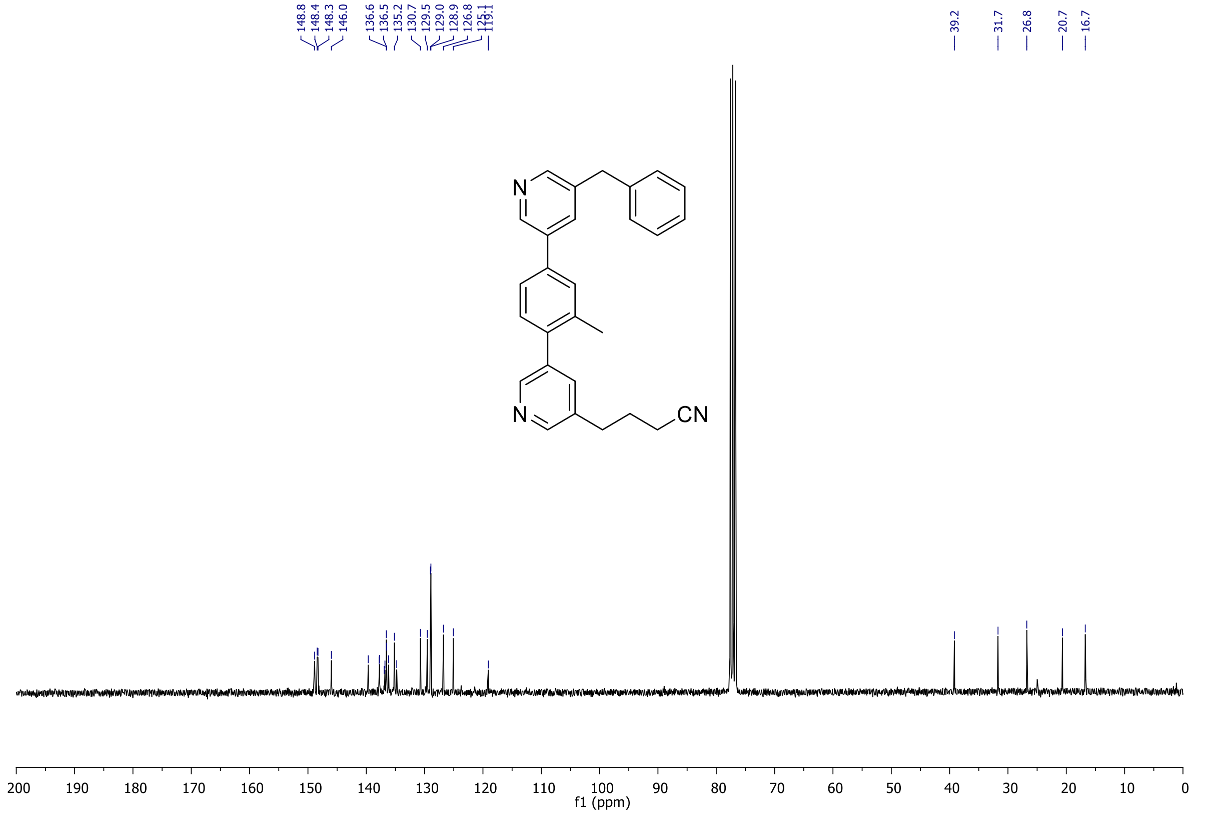

Phe-Ala-Lys (**47**)

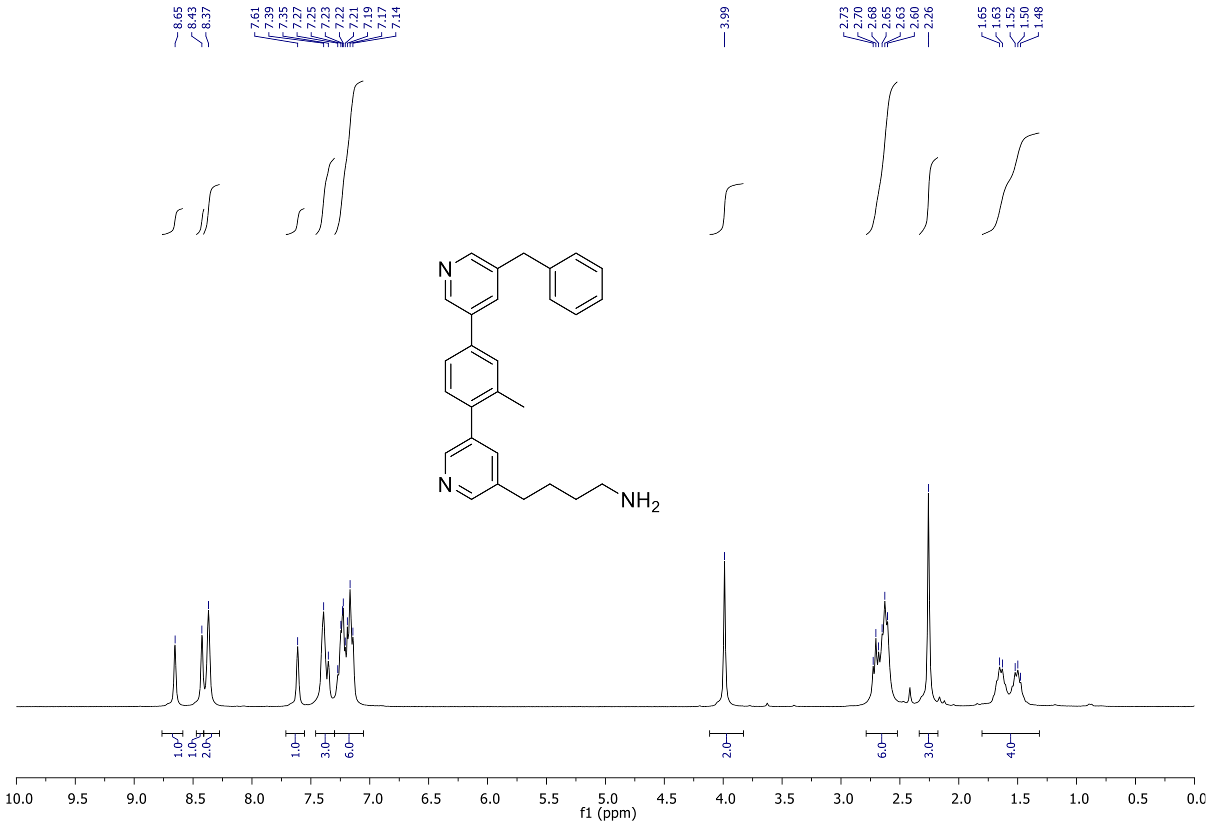

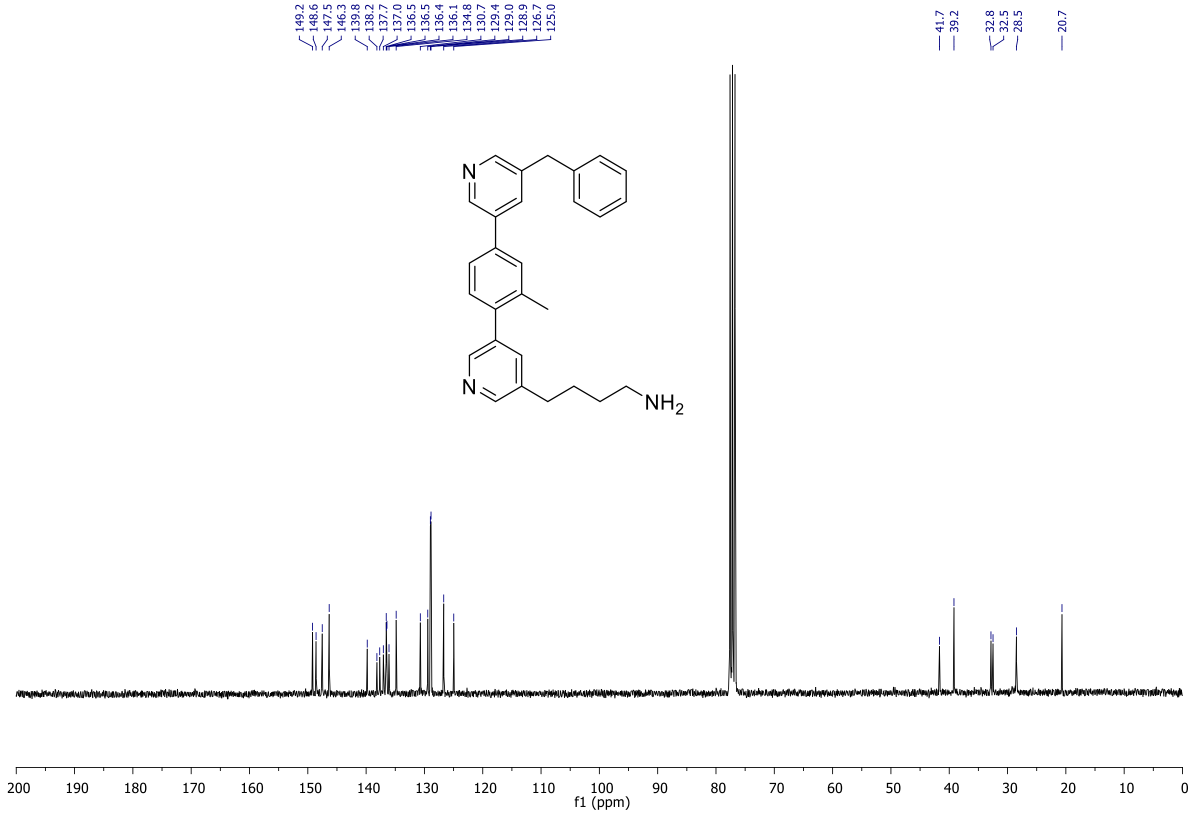

Methyl 3-(2-(5-(hydroxymethyl)pyridin-3-yl)-5-(5-methylpyridin-3-yl)phenyl)propanoate (**48a**)

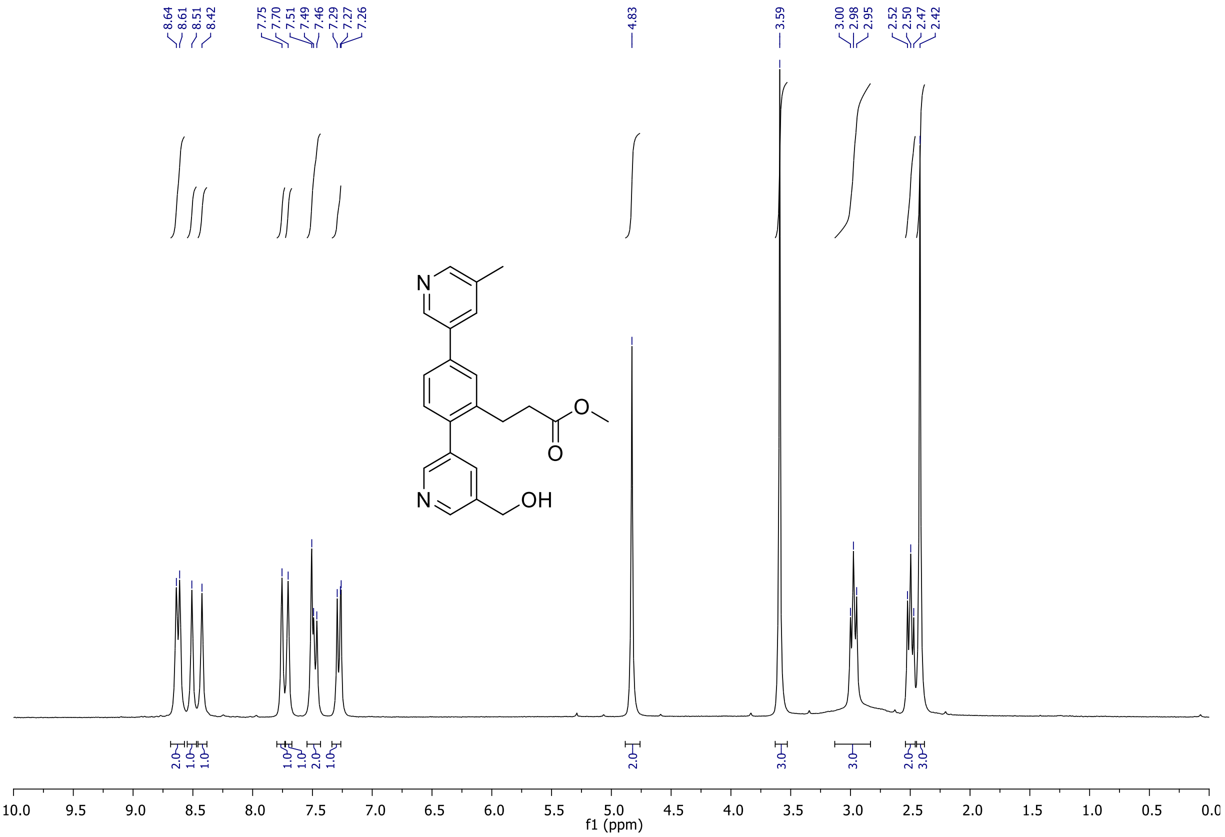

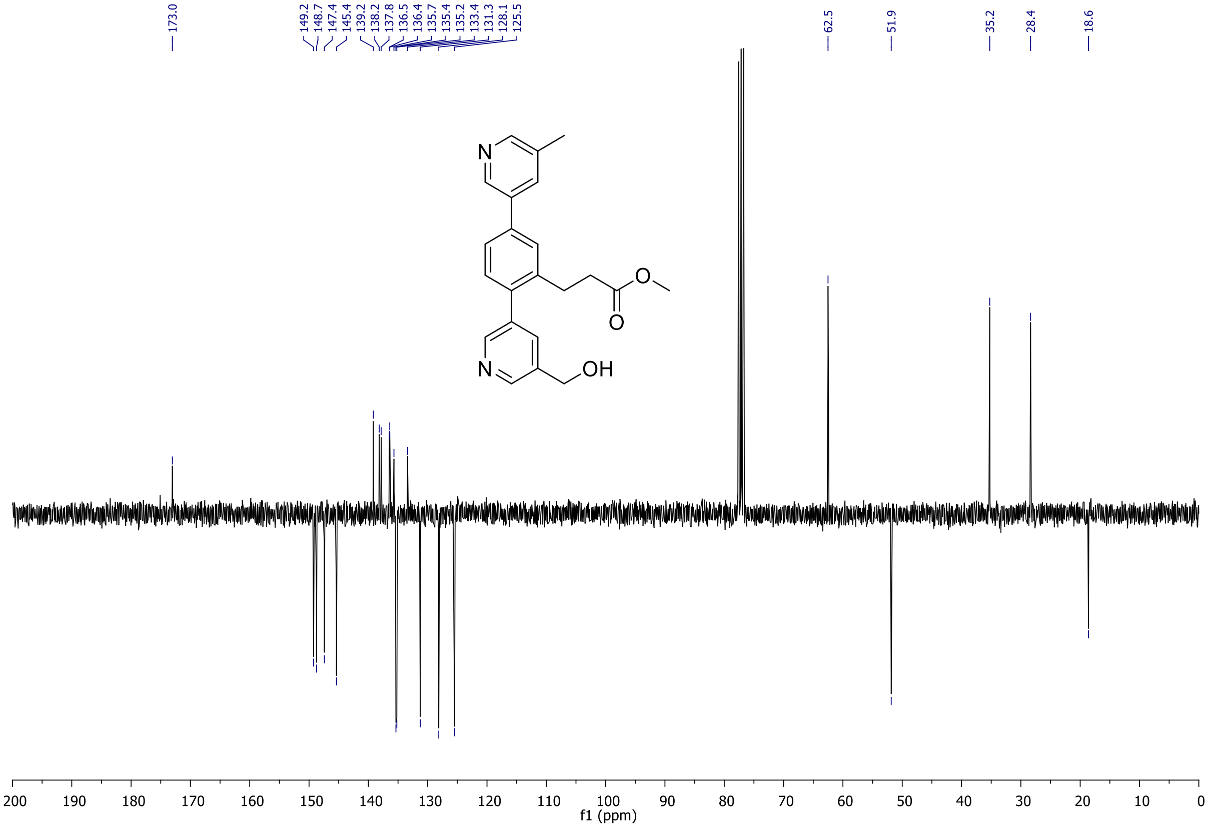

Ala-Glu-Ser (**48**)

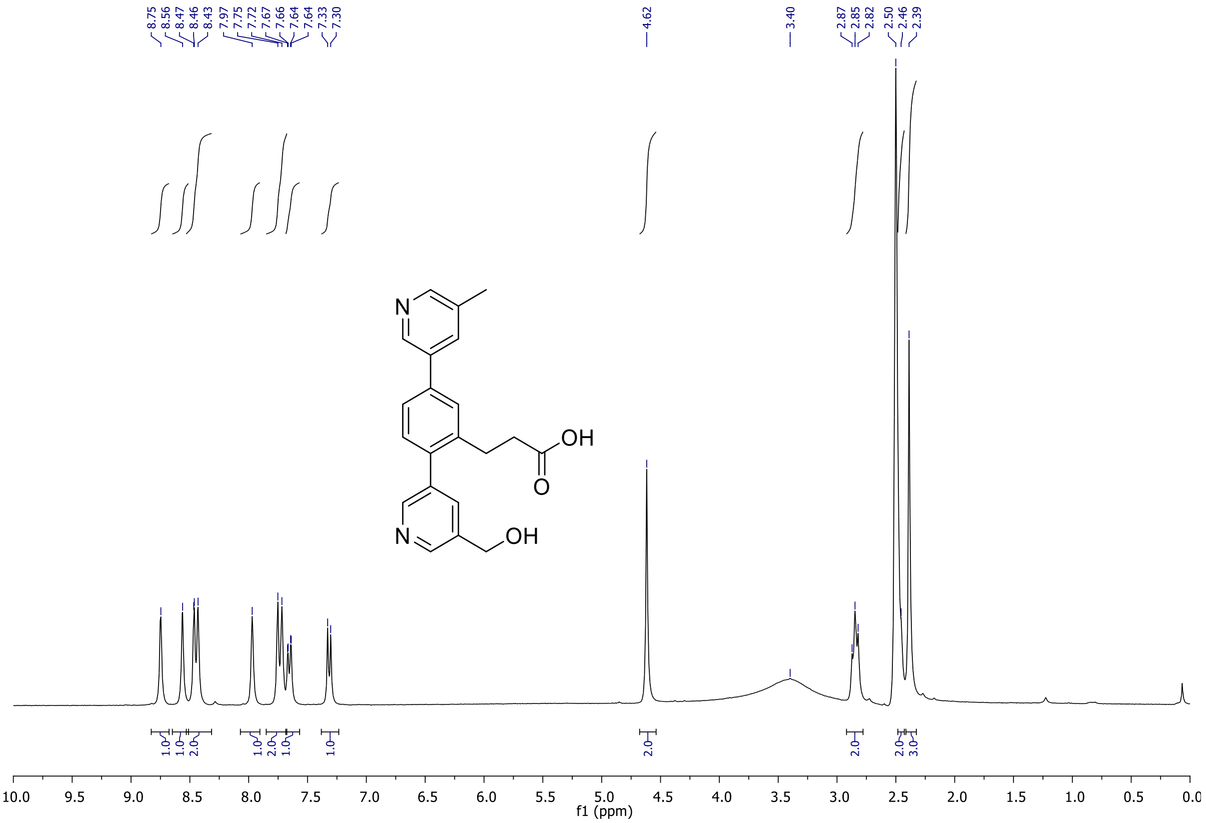

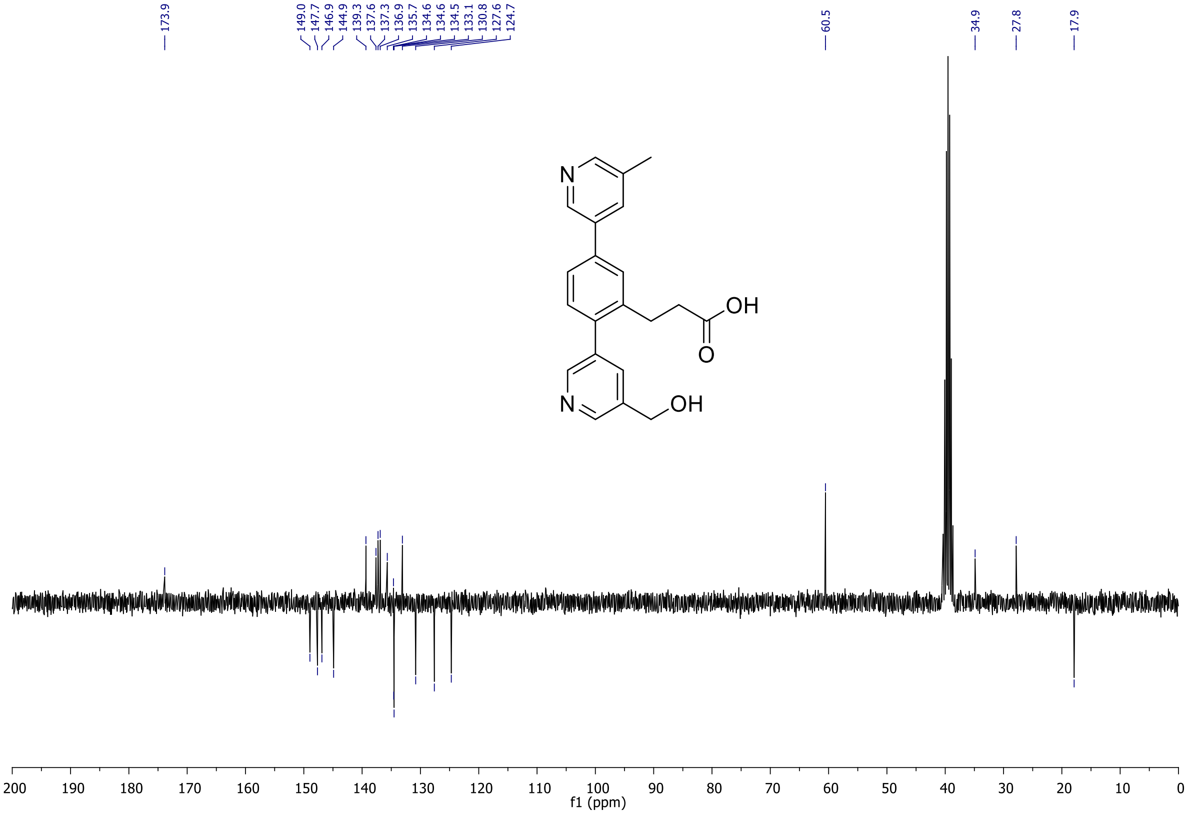

3-(5-(4-(5-Isobutylpyridin-3-yl)-2-methylphenyl)pyridin-3-yl)propanenitrile (**49**)

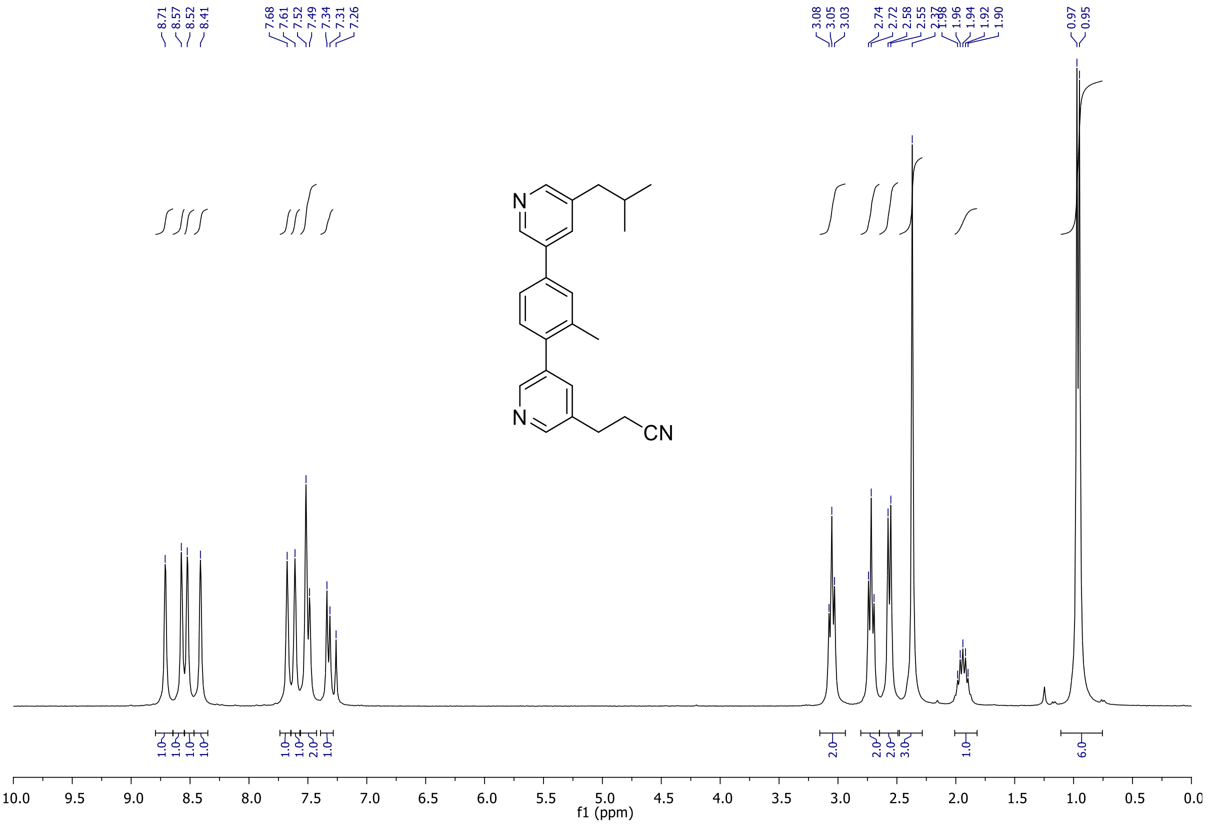

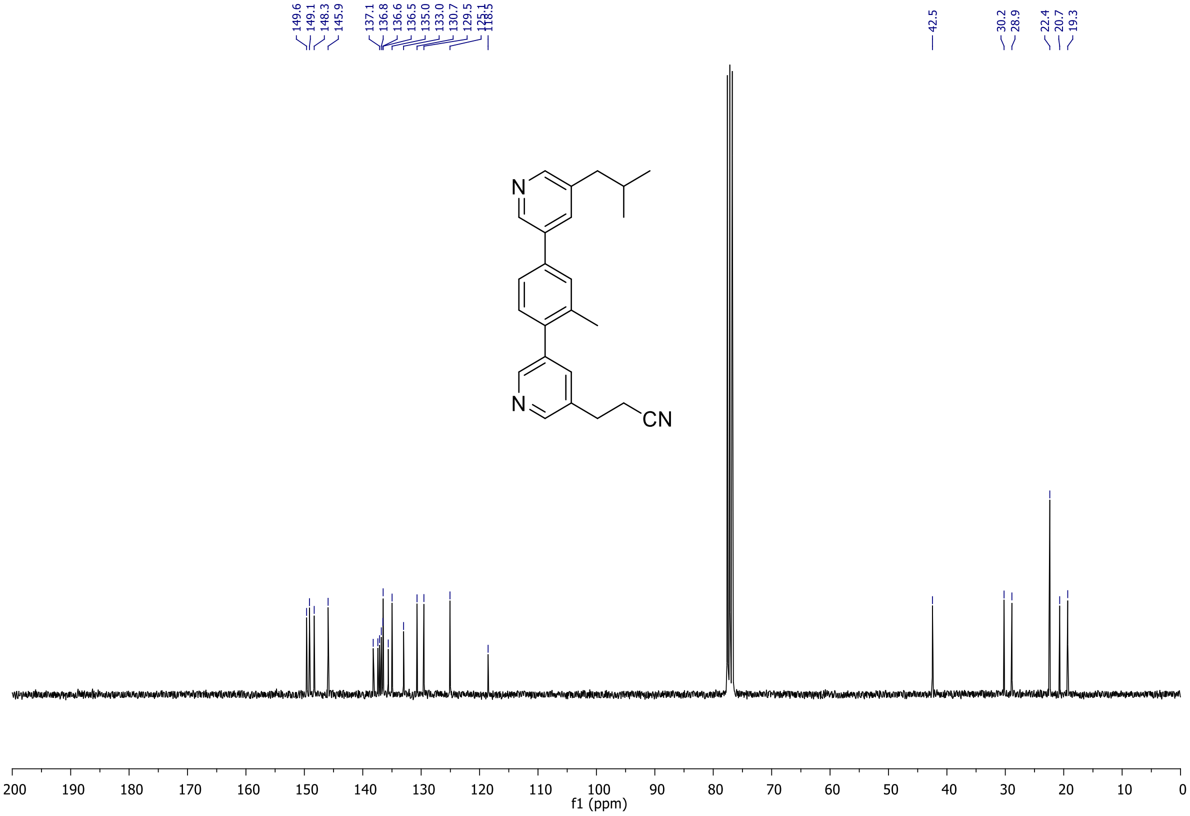

Leu-Ala-Arg (**52**)

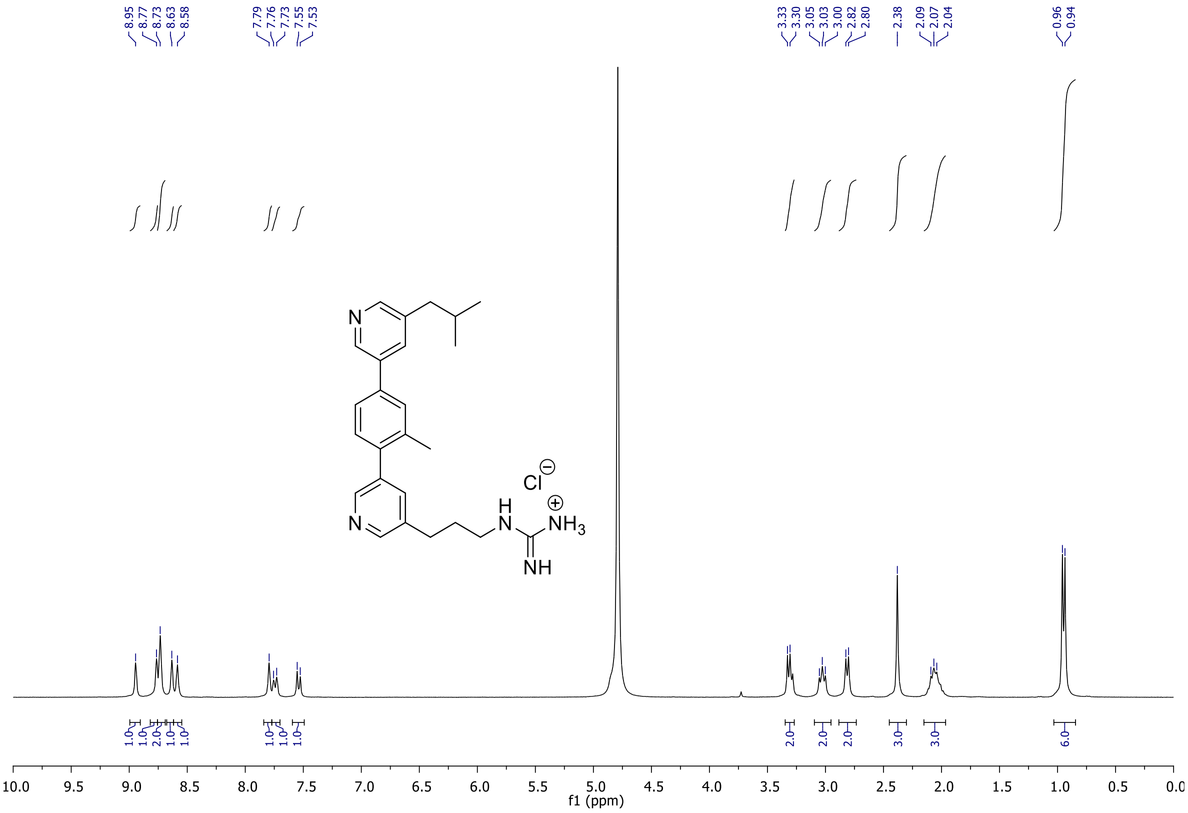

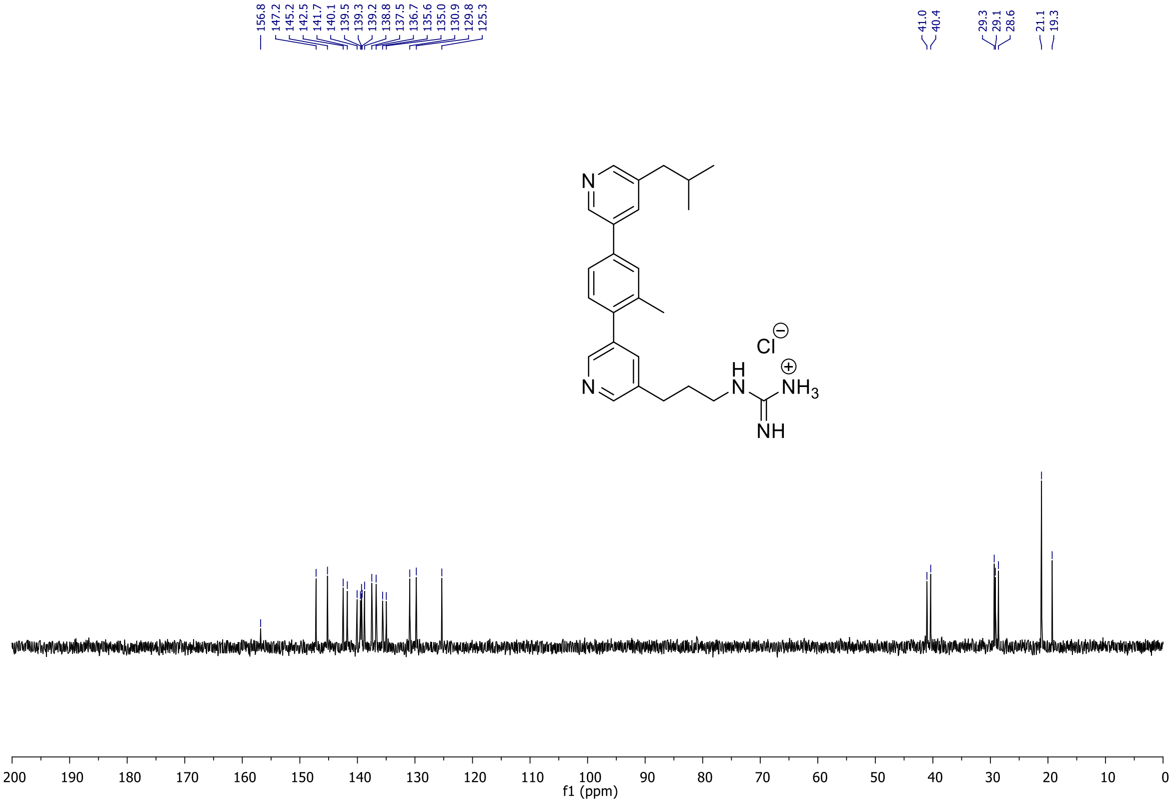

3-(5-(4-(5-(2-Cyanoethyl)pyridin-3-yl)-2-methylphenyl)pyridin-3-yl)propanamide (**53**)

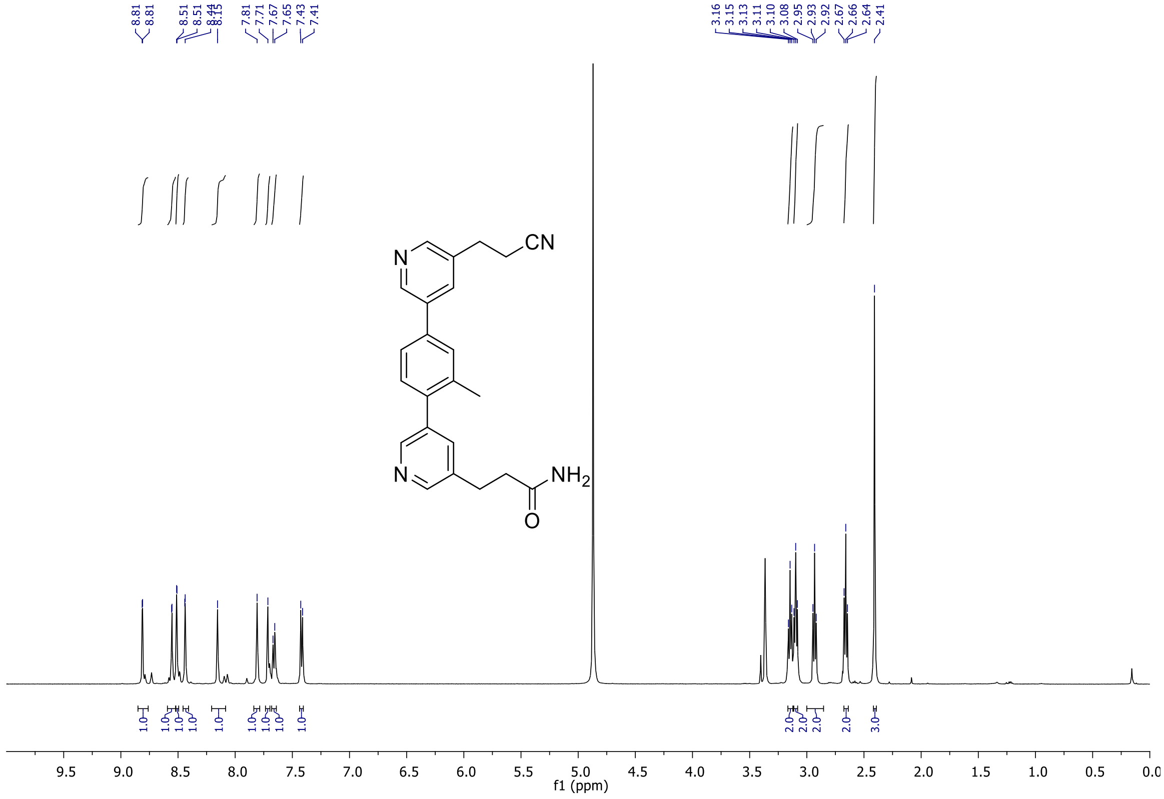

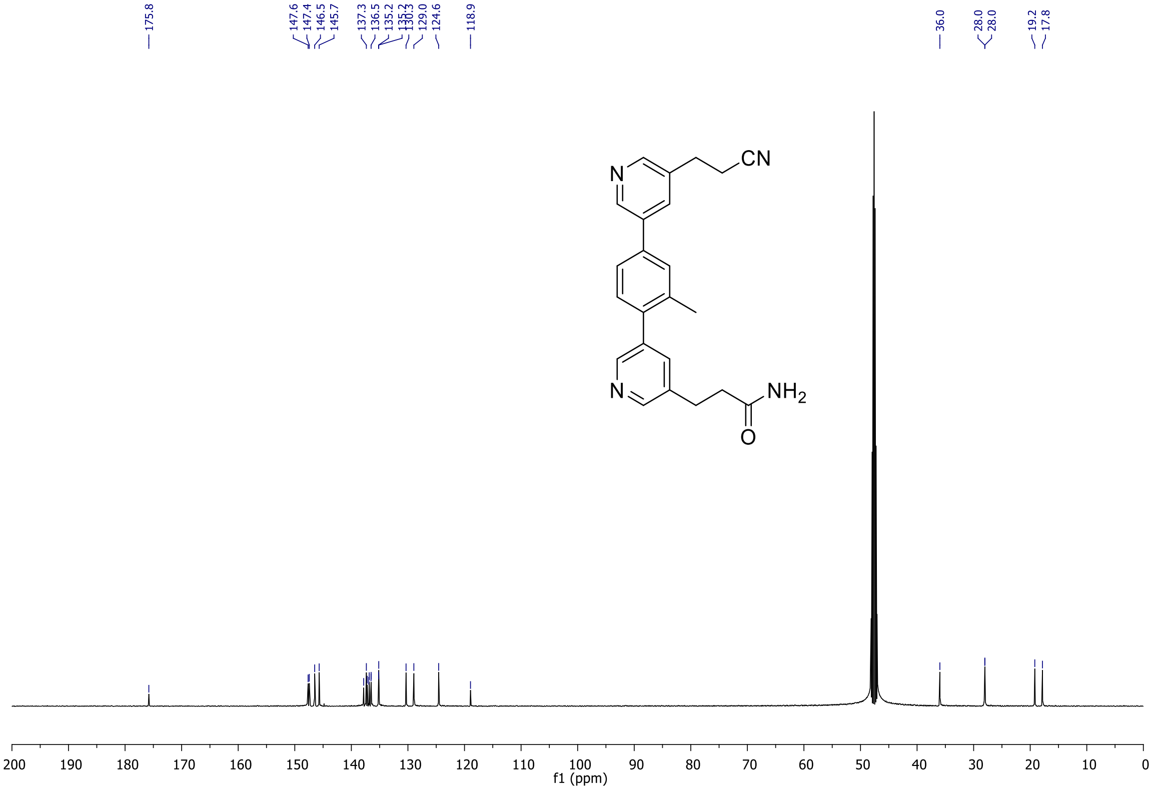

Gln-Ala-Glu (**54**)

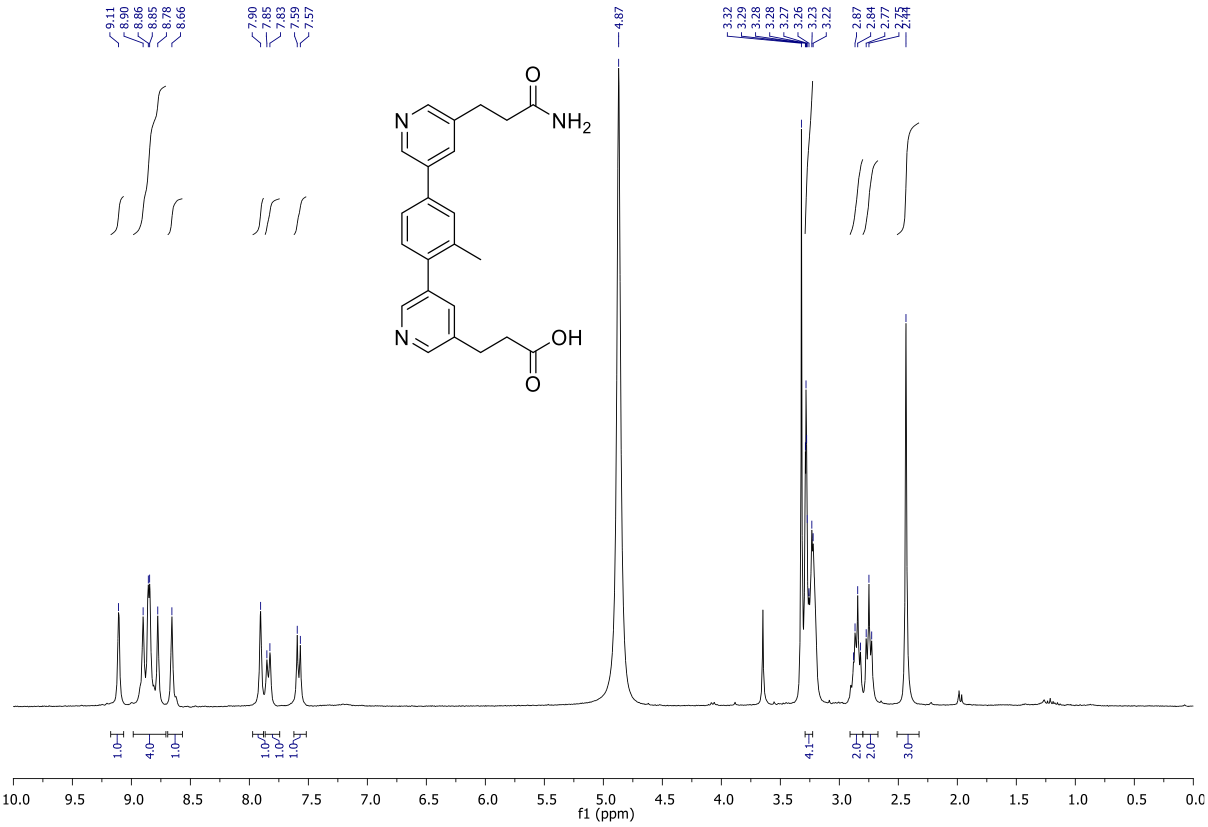

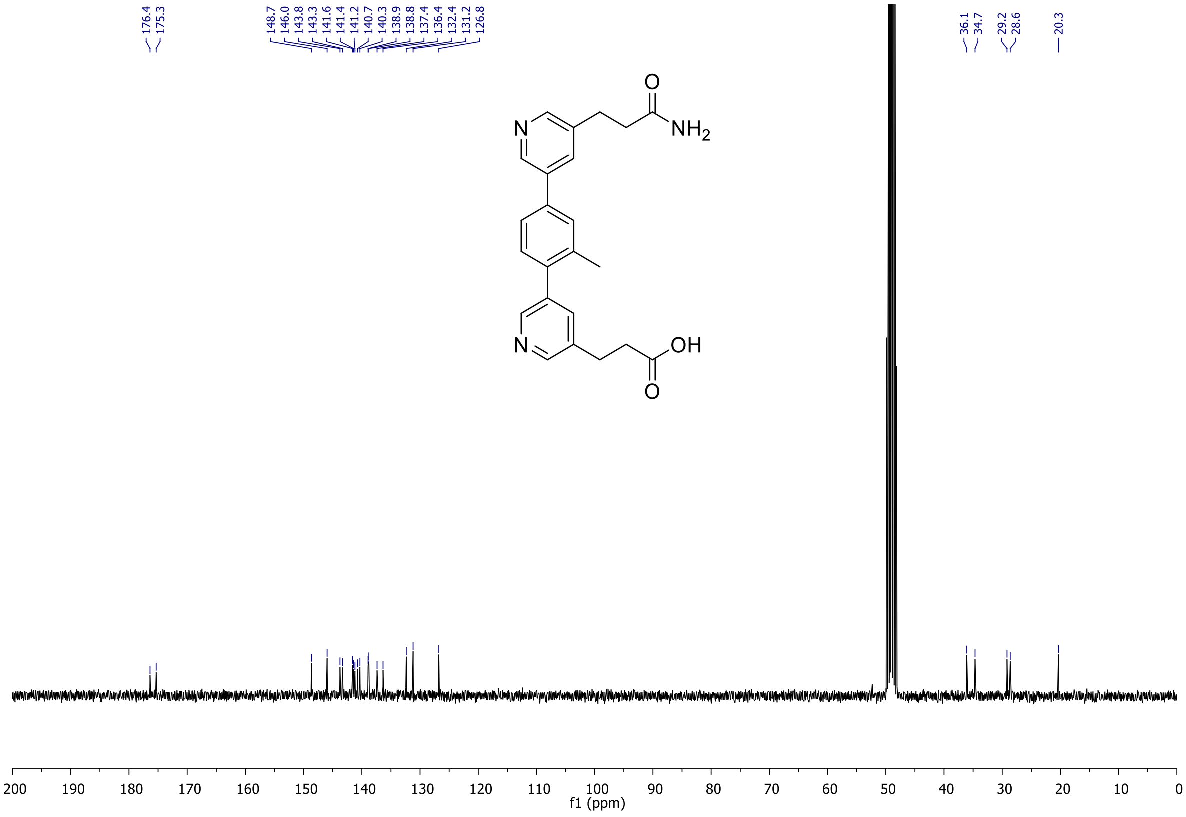

2'-Methyl-1,1':4',1''-terphenyl (**55**)

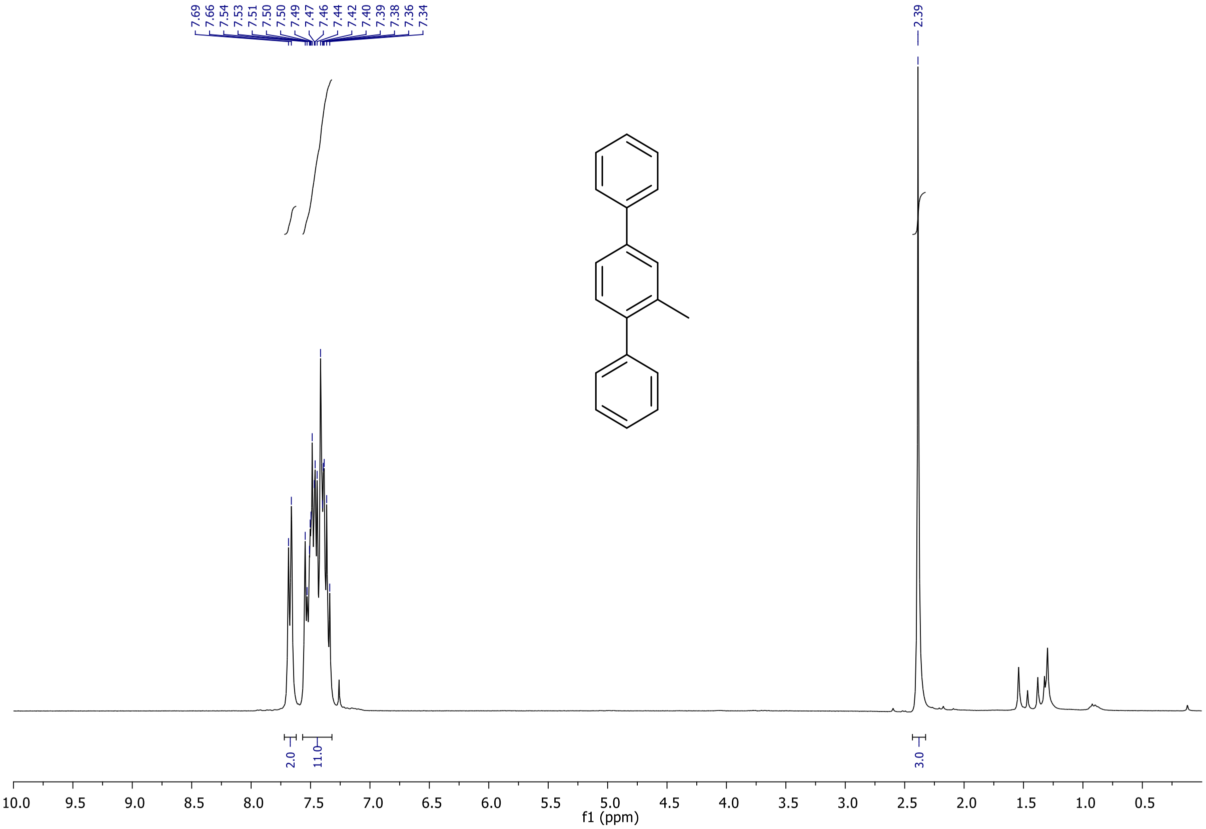

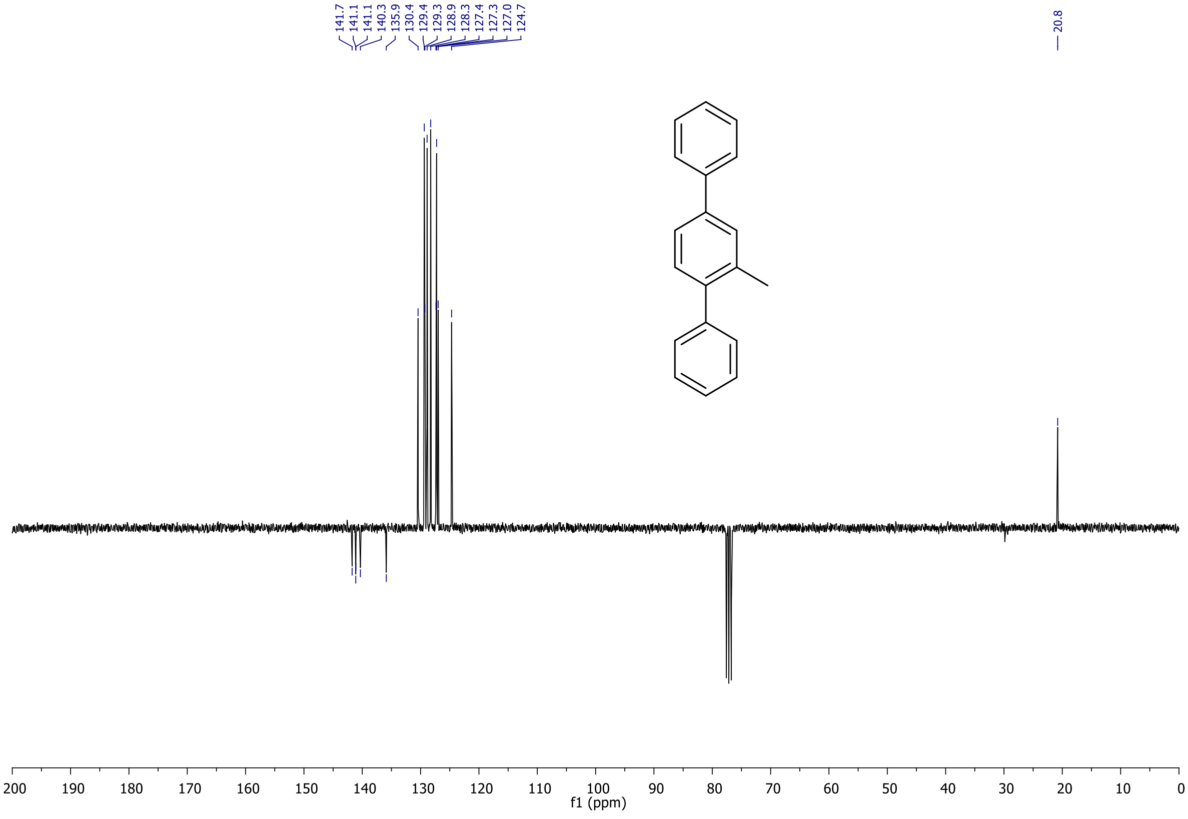

3-(2-Methyl-[1,1'-biphenyl]-4-yl)pyridine (**56**)

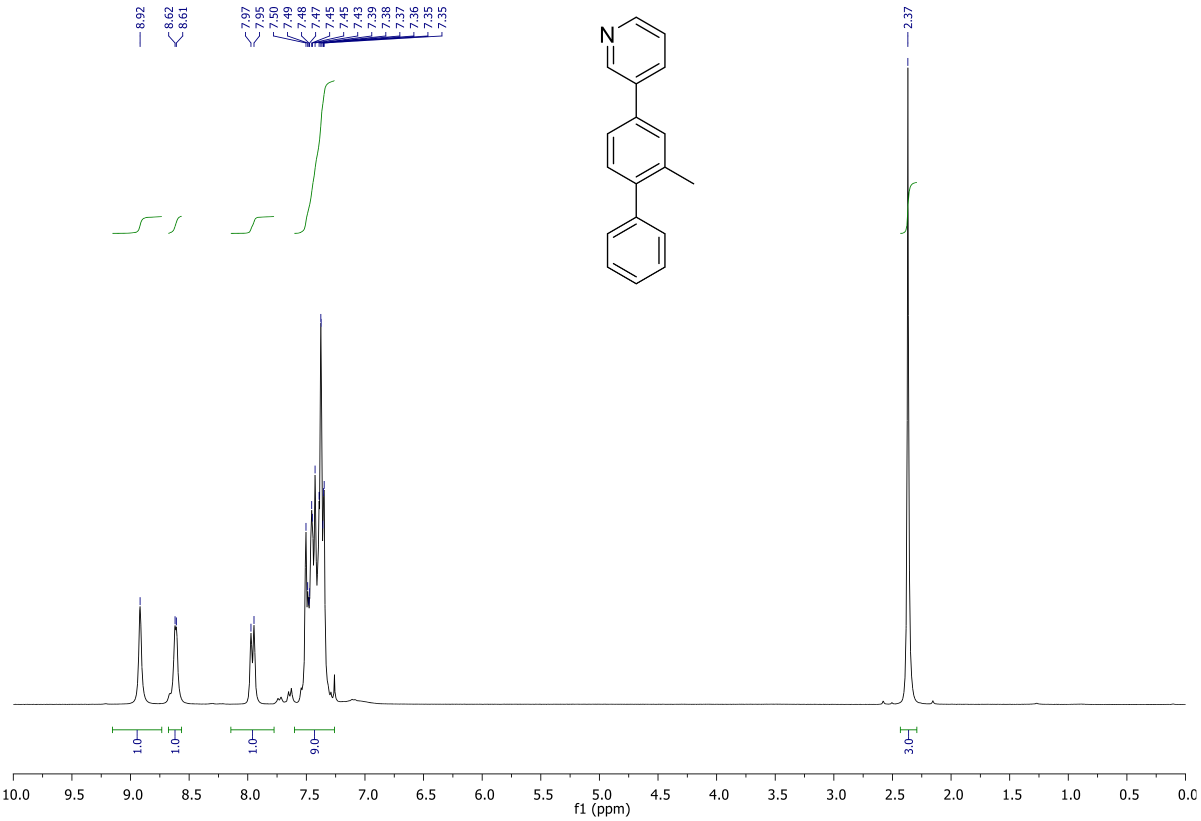

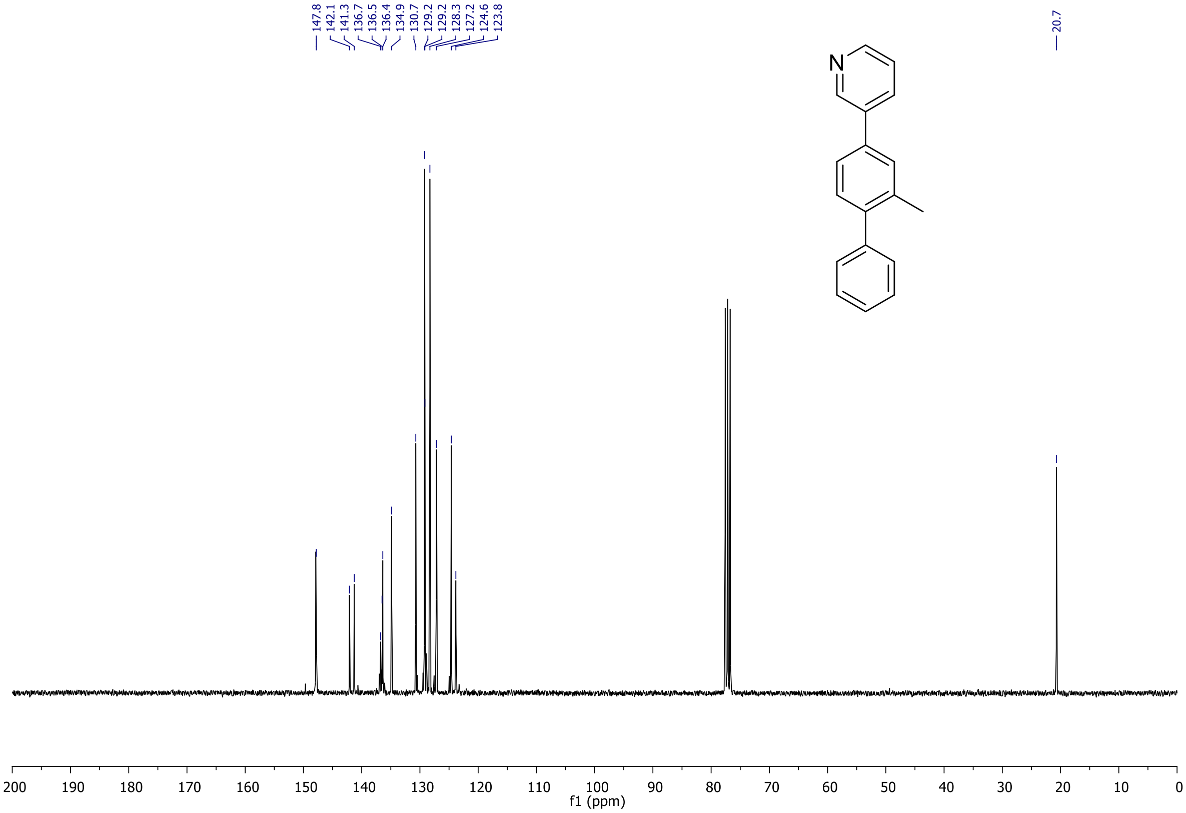

Gly-Ala-Gly (**57**)

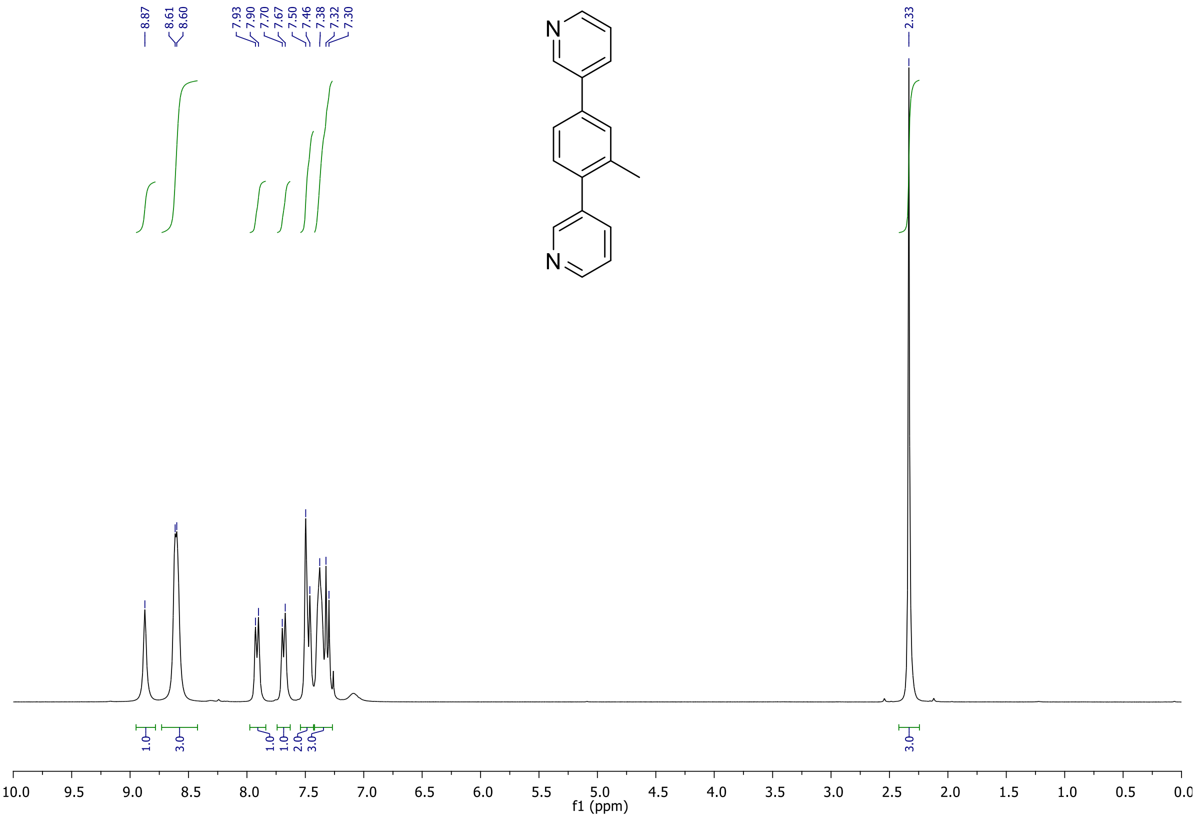

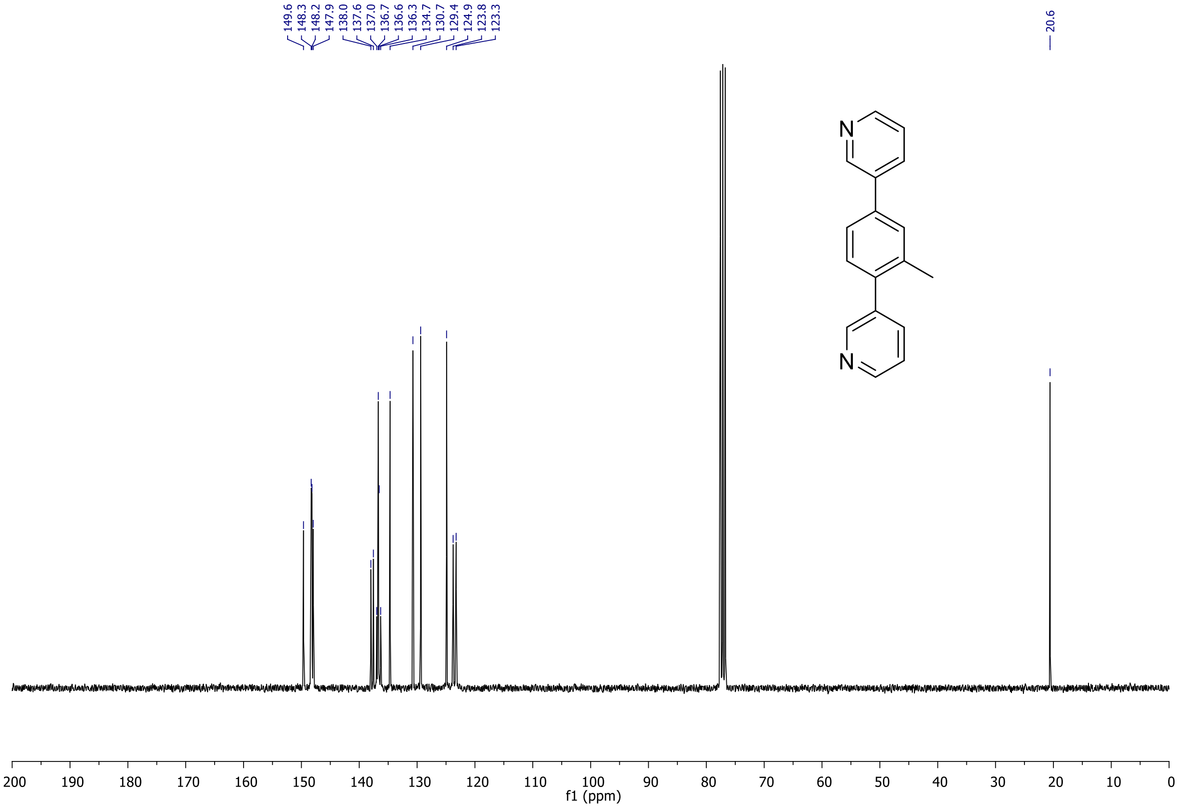

2-Bromo-5-iodobenzaldehyde (**58a**)

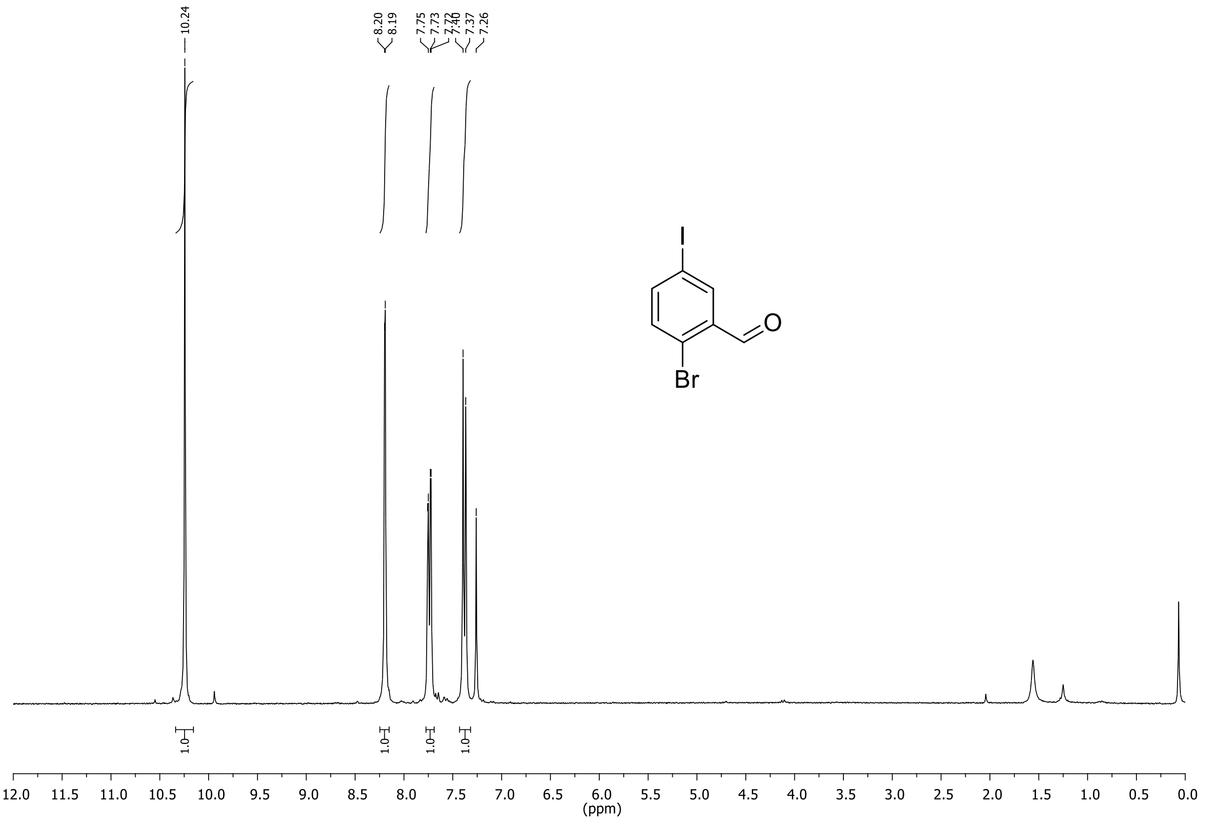

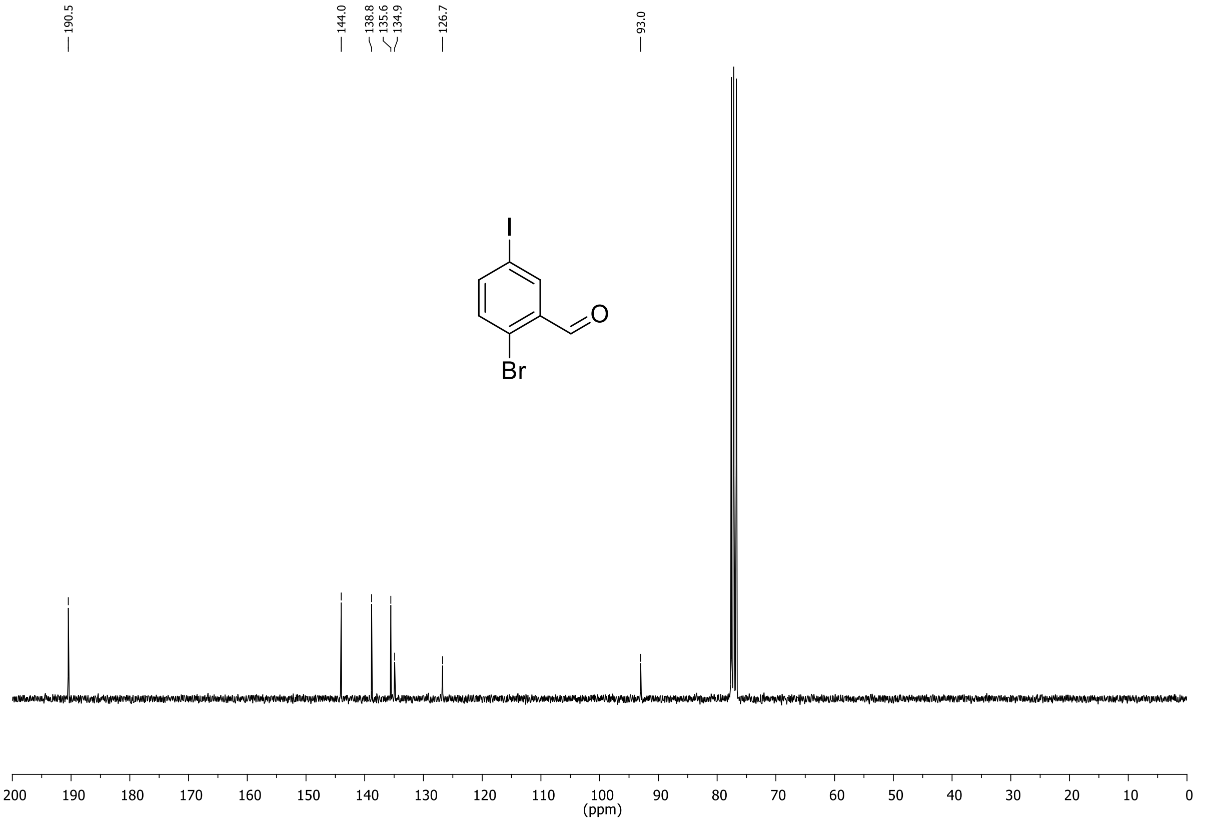

Ethyl 3-(2-bromo-5-iodophenyl)acrylate (**58b**)

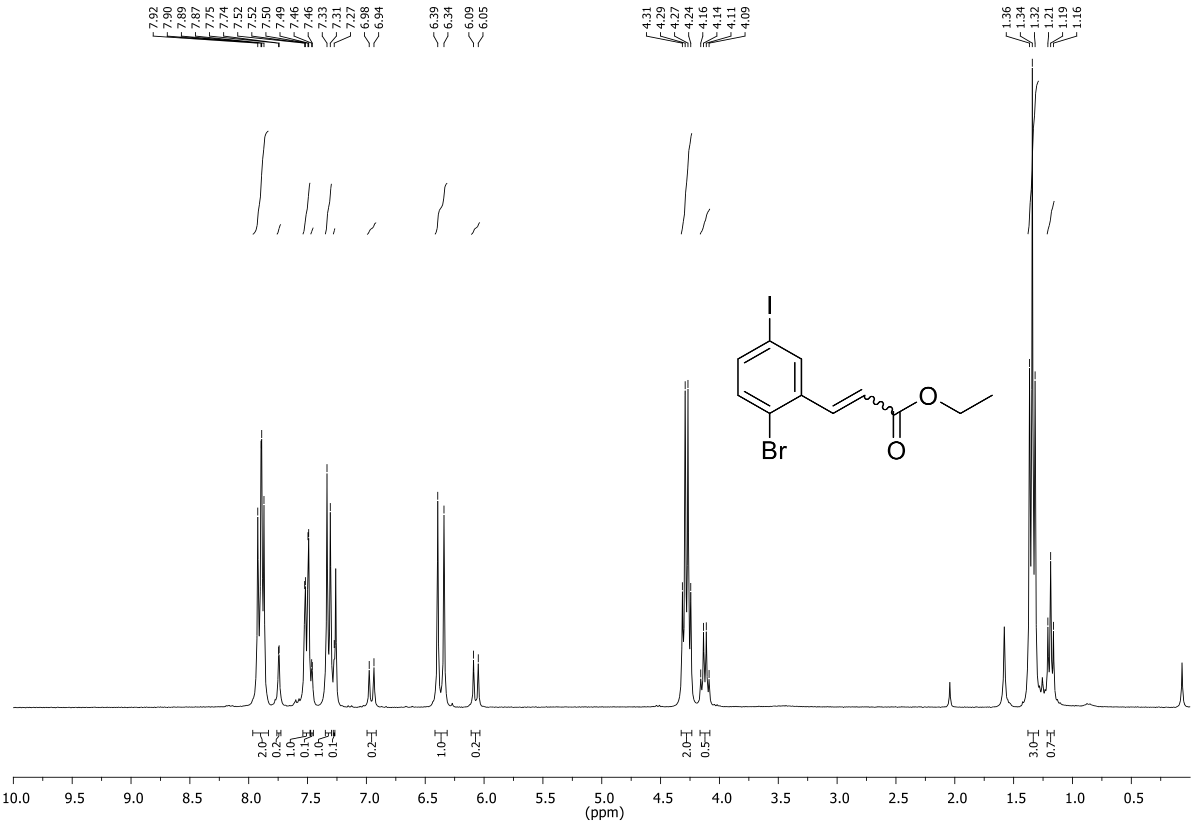

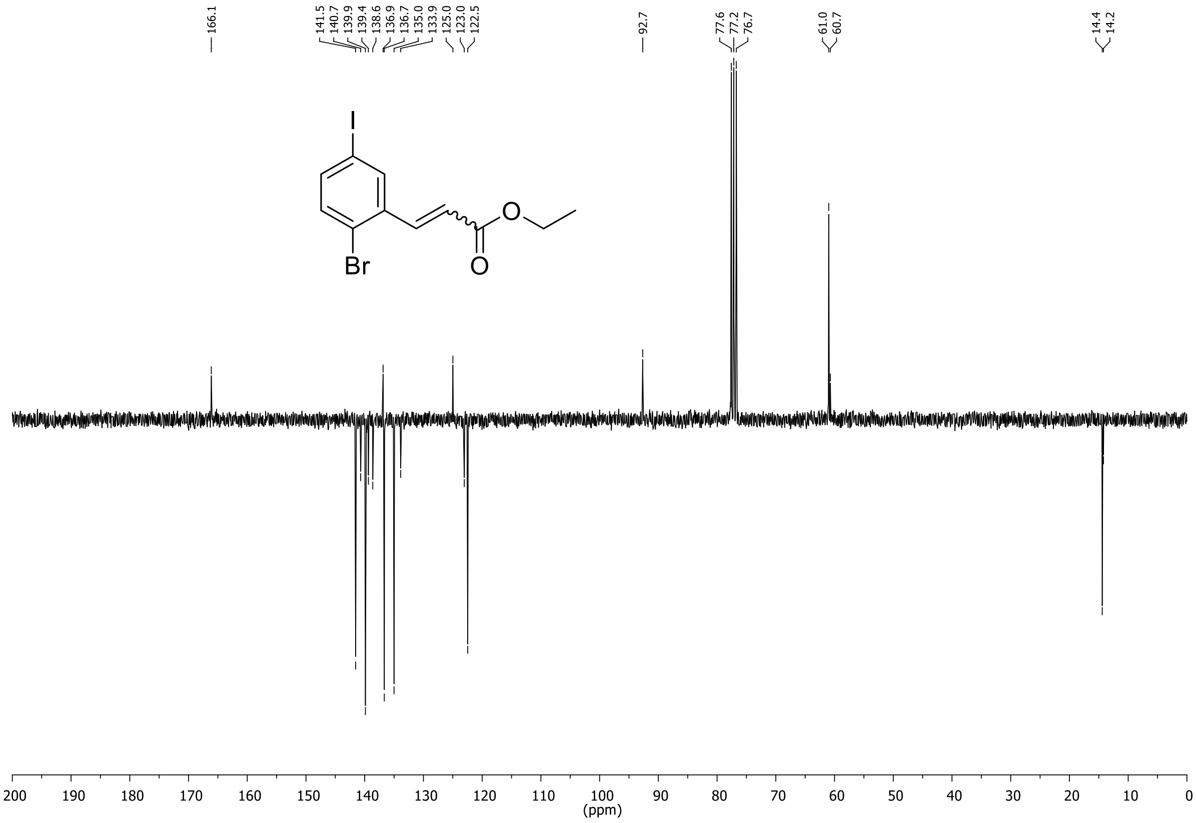

Ethyl 3-(2-bromo-5-iodophenyl)propanoate (**58c**)

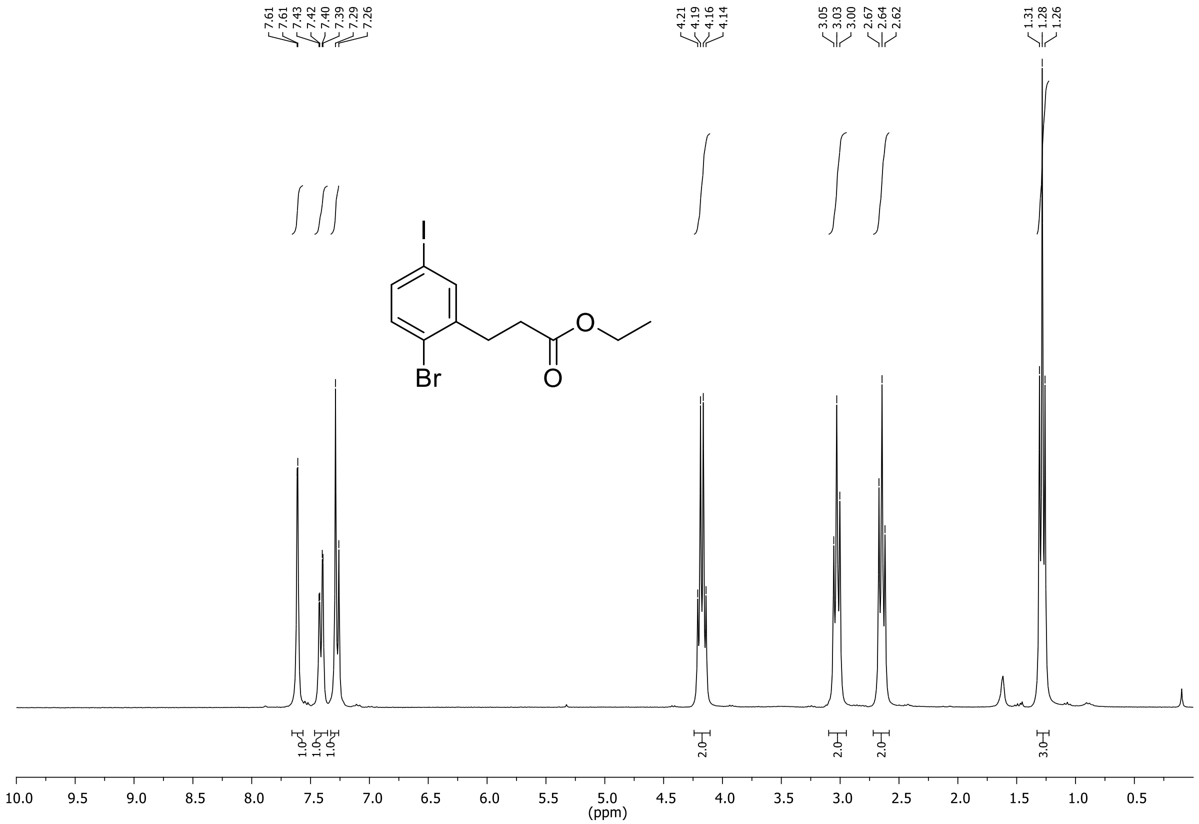

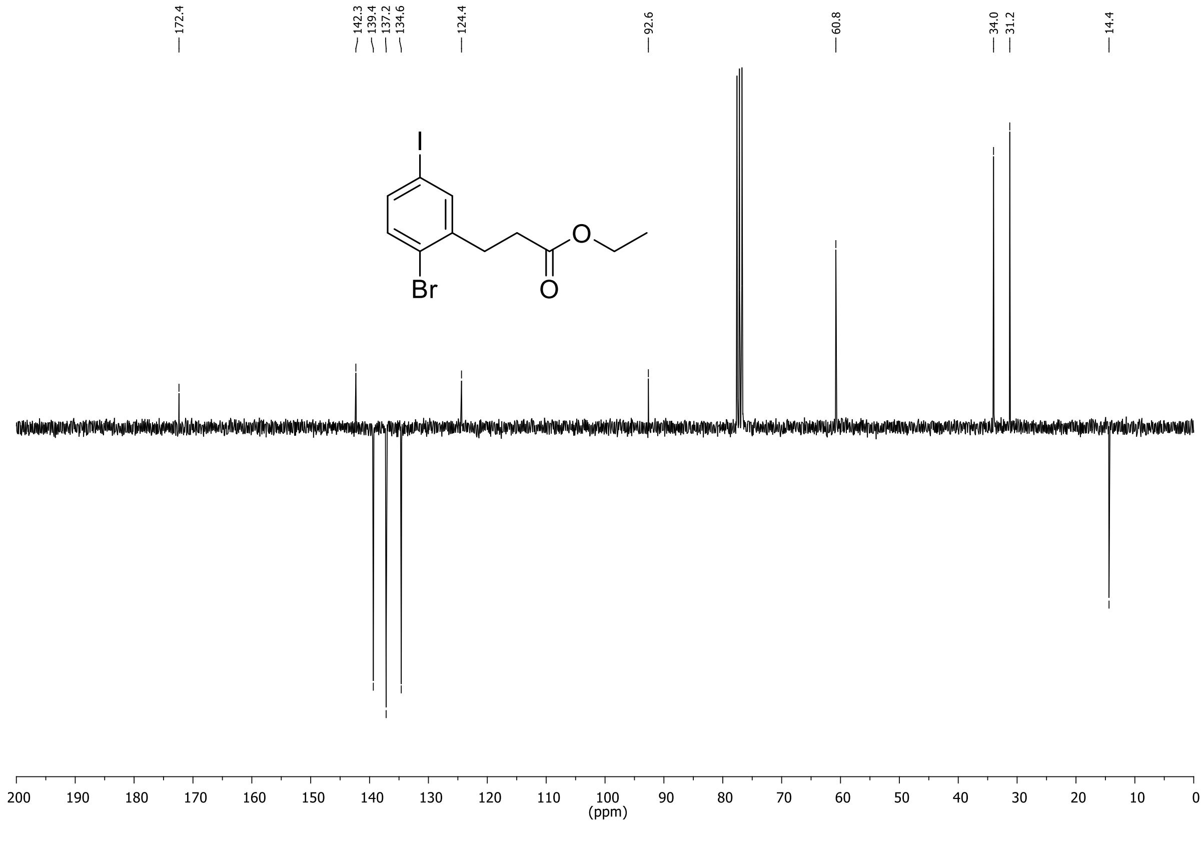

3-(3,3''-Diisobutyl-[1,1':4',1''-terphenyl]-2'-yl)propanoic acid (**58**)

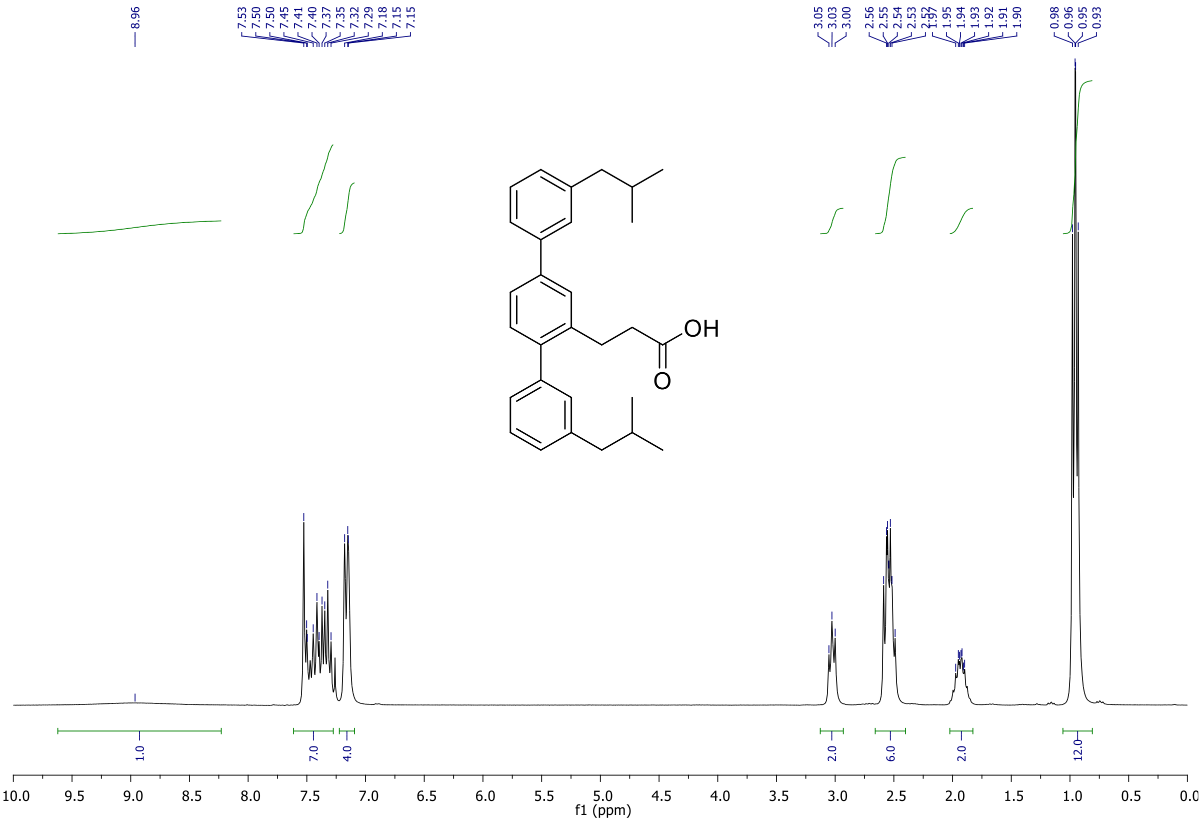

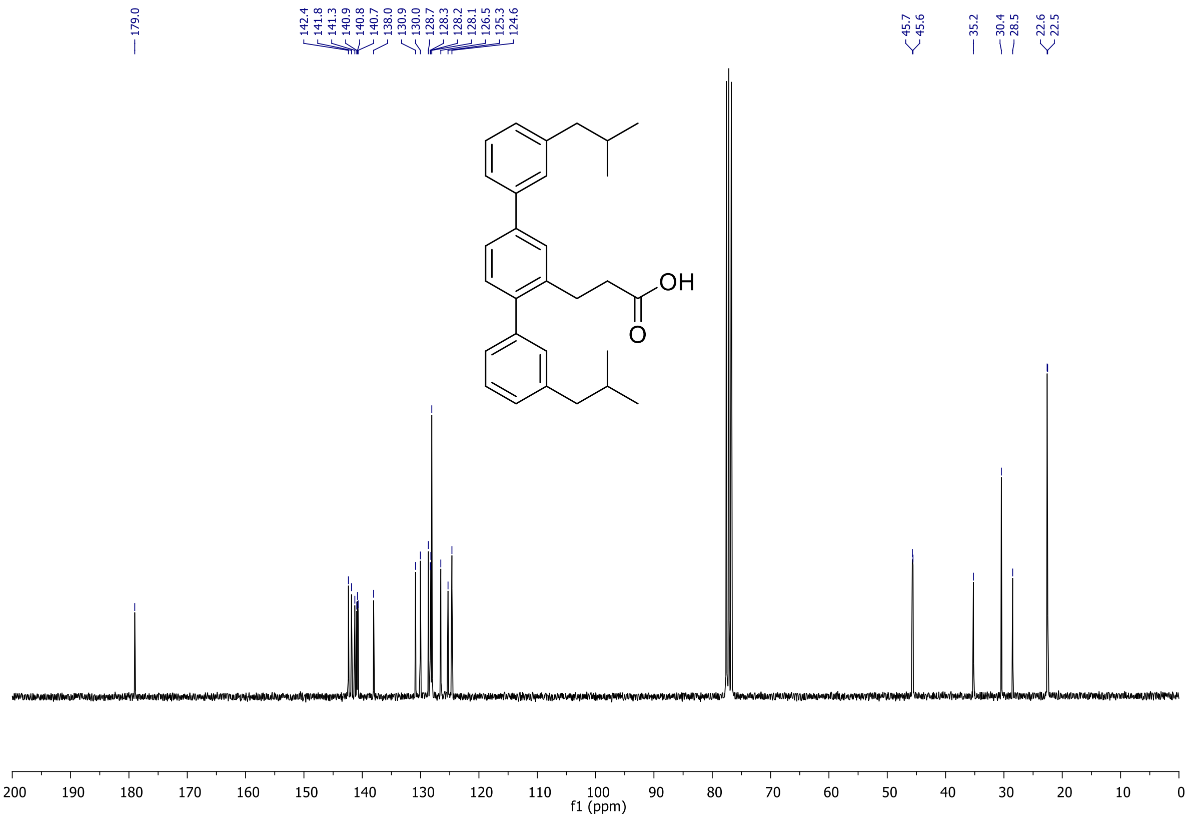

3-(3'-Isobutyl-4-(5-isobutylpyridin-3-yl)-[1,1'-biphenyl]-2-yl)propanoic acid (**59**)

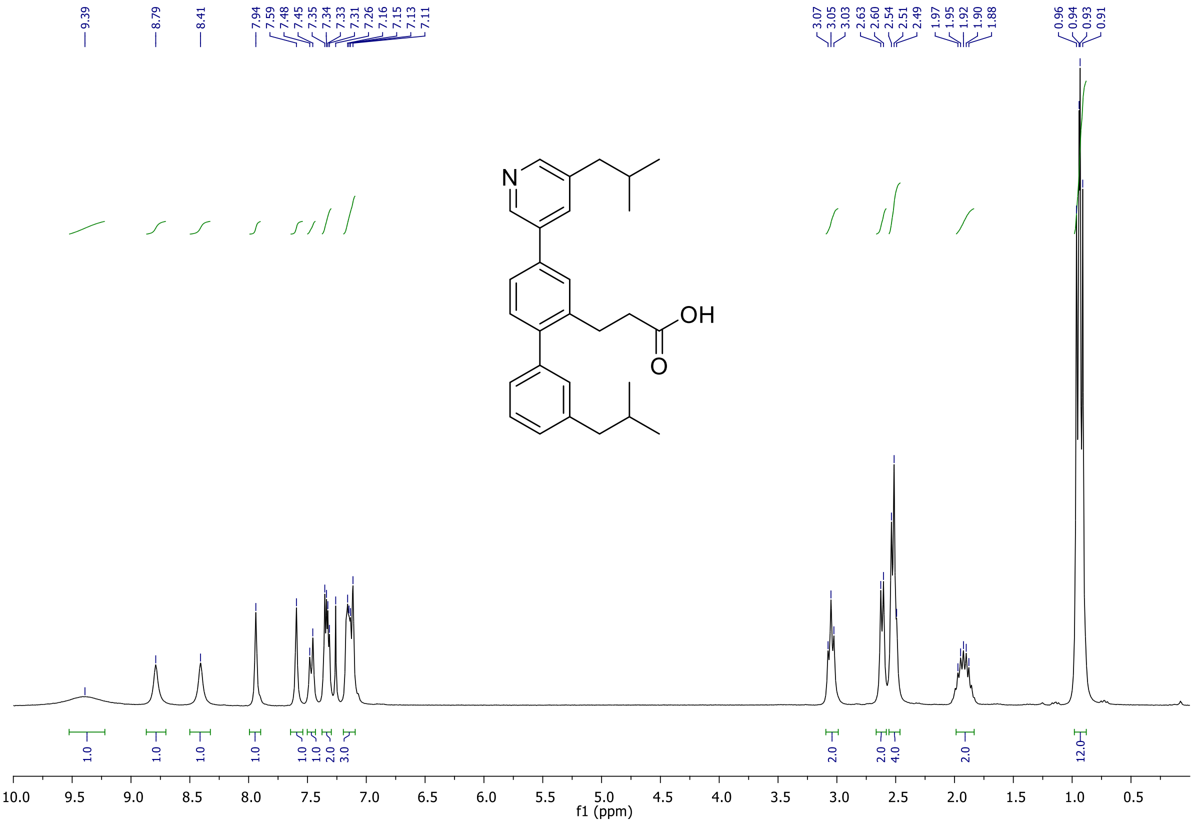

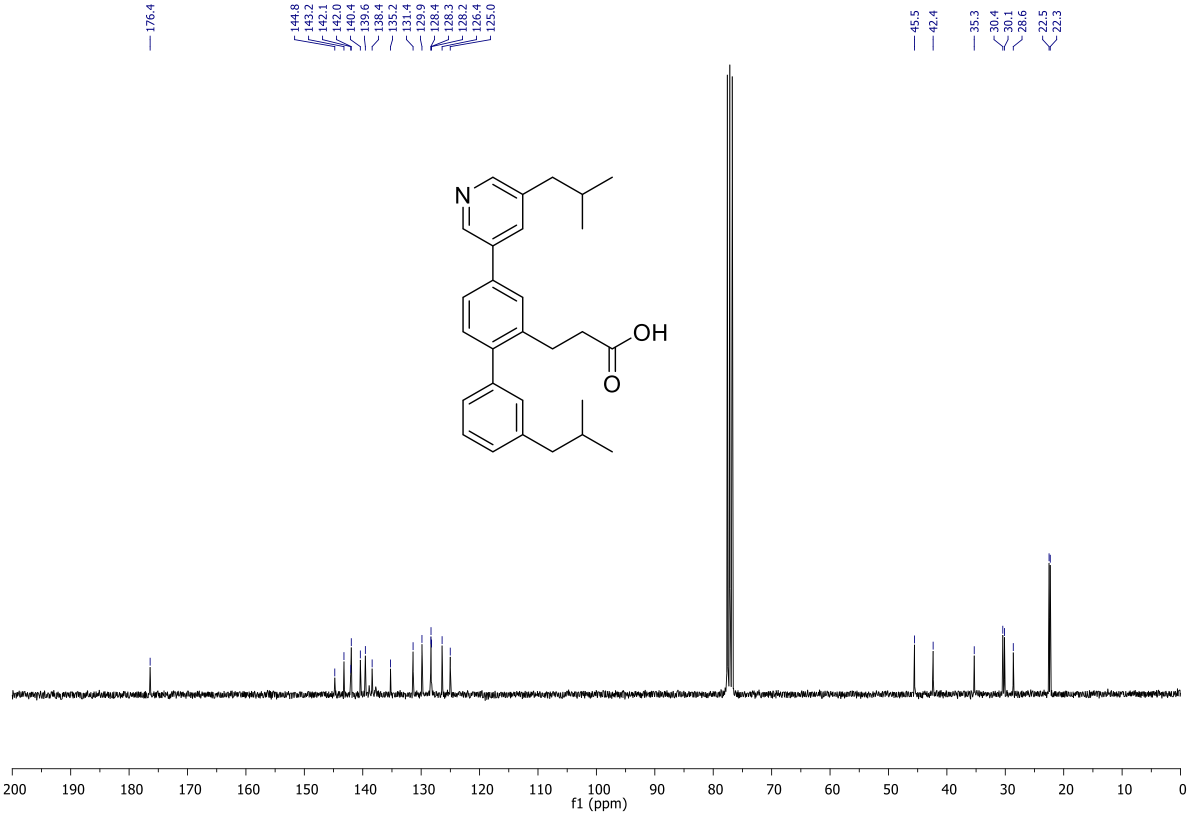

Leu-Glu-Leu (**60**)

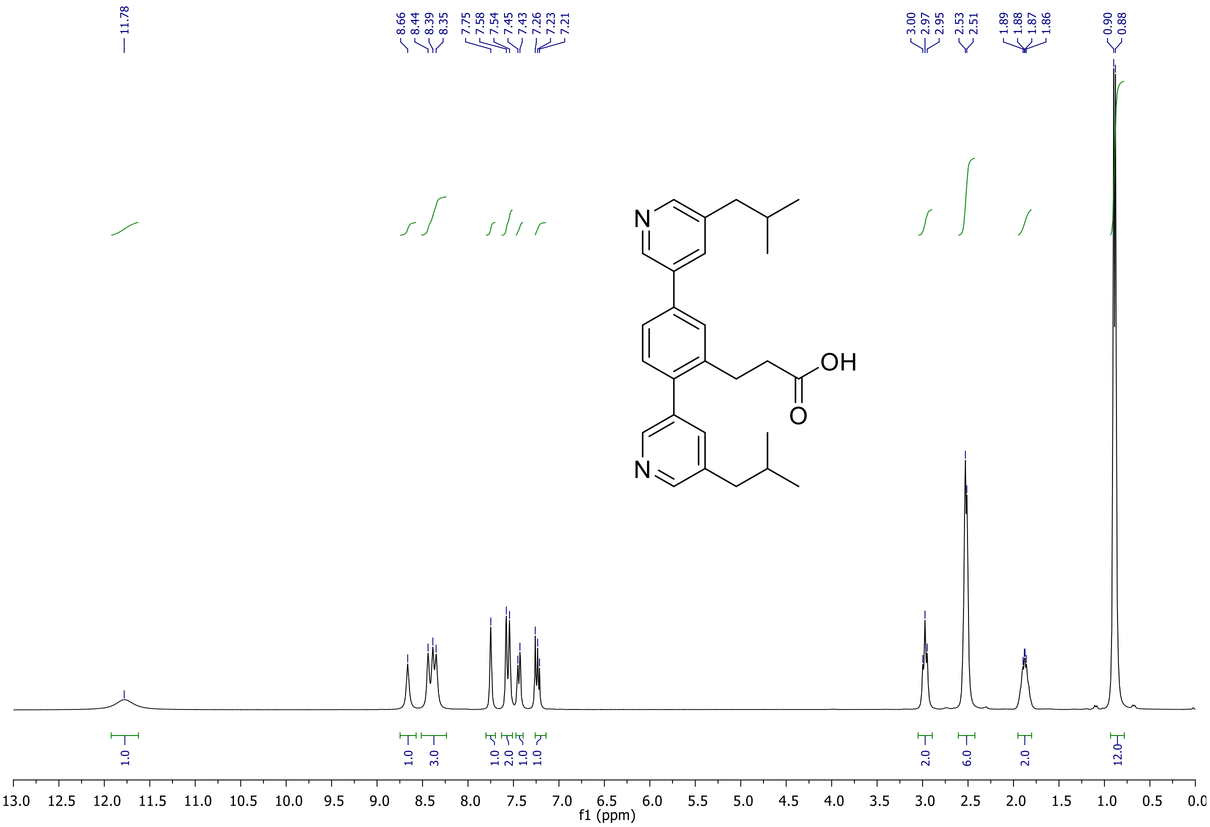

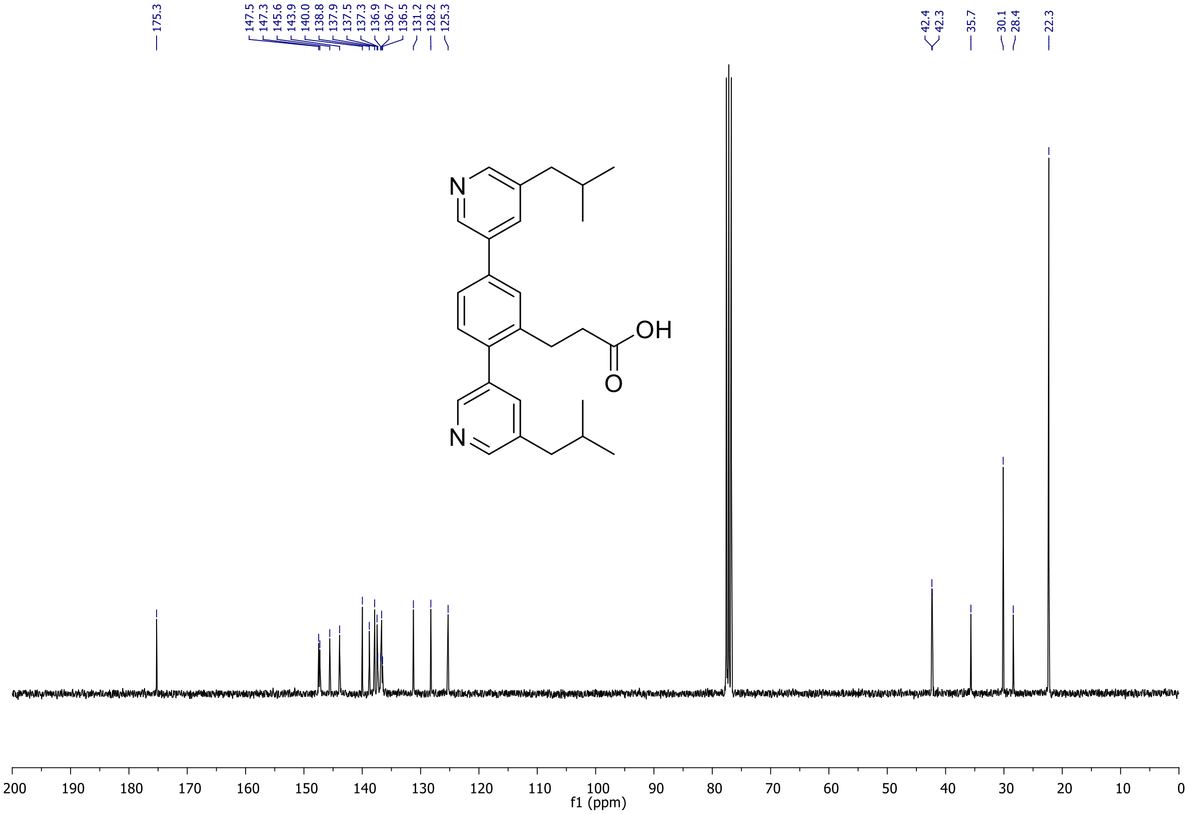

Supplement: Supplementary file 1 — Supporting Information [file EJOC-2022-0-s001.pdf]
